# Directed copper-catalyzed C–H functionalization of unactivated olefins with azodicarbonamide compounds†

**DOI:** 10.1039/d4ra04113e

**Published:** 2024-08-29

**Authors:** Jing Cui, Xiaoya Wang, Runsheng Zeng

**Affiliations:** a Key Laboratory of Organic Synthesis of Jiangsu Province, College of Chemistry, Chemical Engineering and Materials Science, Soochow University Suzhou 215123 P. R. China zengrunsheng@suda.edu.cn

## Abstract

The copper-catalyzed strategy employing the 8-aminoquinoline directing group has proven to be a highly advantageous approach for functionalizing C–H bonds. In this study, we present the successful application of this strategy to accomplish Heck-type coupling reactions and construct β-lactam skeletons, simultaneously introducing a unique cyano functional group. The resulting Heck-type coupling products demonstrate good stereo- and region-selectivity. Initial mechanistic investigations indicate that the reaction proceeds *via* a radical coupling mechanism, exhibiting a wide substrate scope and delivering good yields.

## Abstract 2

The copper-catalyzed strategy employing directing group has proven to be a advantageous approach. In this study, we present the successful application of this strategy to accomplish Heck-type coupling reactions and construct β-lactam skeletons.

## Graphical abstract

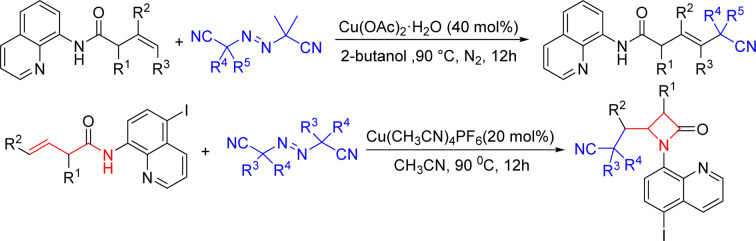

## Introduction

Transition metal-catalyzed direct C–H functionalization reactions represent a highly efficient method for the construction of complex molecules in organic synthesis, offering advantages such as atom economy and step economy.^1^ This method enables the rapid introduction of C–C and C–X bonds. Although alkenes are commonly used in C–H activation due to their availability, affordability, and unique reactive sites,^2^ studies on C–H functionalization of unactivated alkenes remain limited.^3^ This is due to their lower reactivity and challenging control of regioselectivity. However, the development of directing groups has proven effective in addressing these issues.^4^ Especially, 8-aminoquinoline has shown distinct advantages in C–H functionalization of unactivated alkenes.^5^

**Scheme 1:**
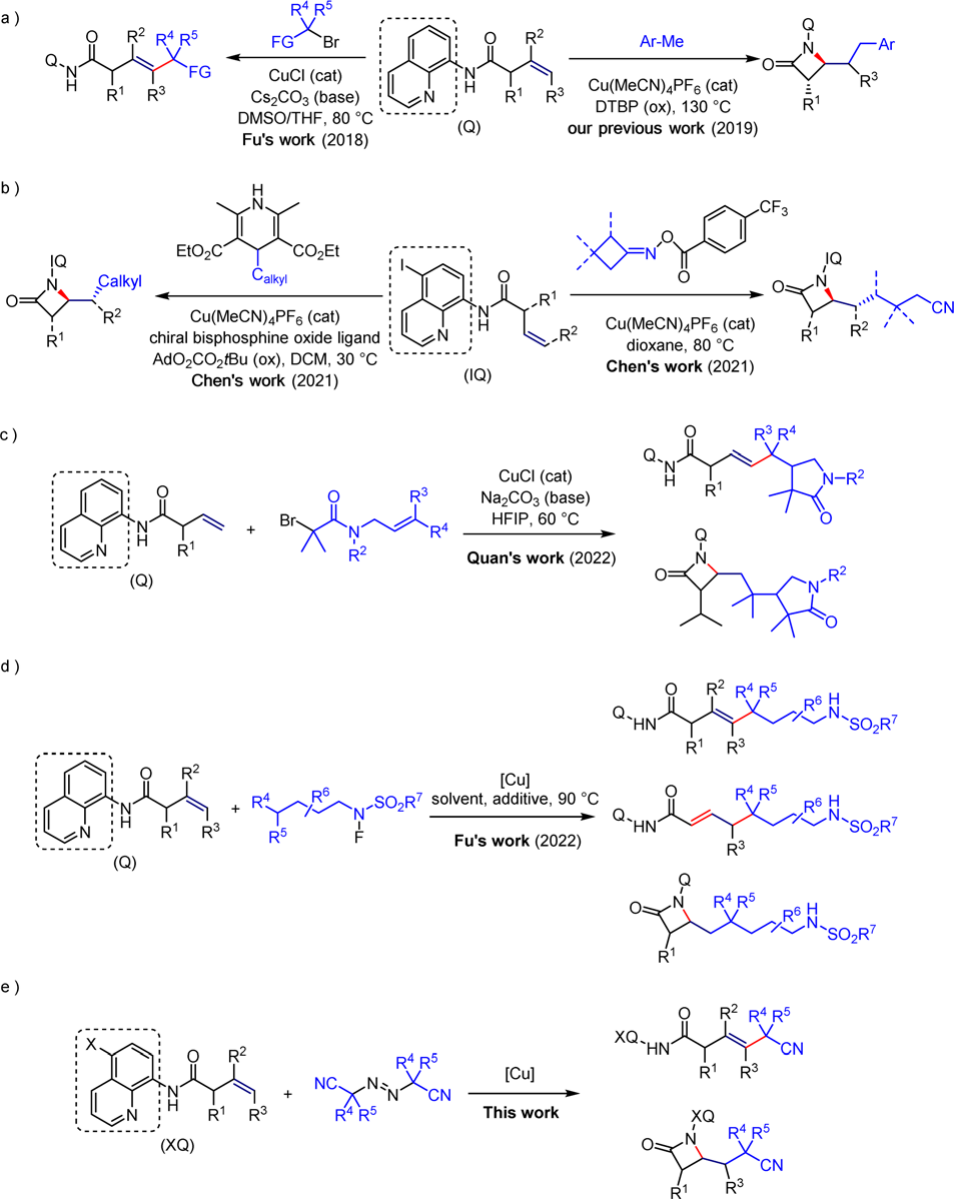
Previous work and this study.

In this field, various research groups, with Engle's group as a representative, have accomplished C–H functionalization reactions of unactivated olefins through the use of palladium-catalyzed,^6^ nickel-catalyzed,^7^ and cobalt-catalyzed^8^ methods. Simultaneously, significant progress has been made in the copper mediated functionalization of olefins. In 2018, Fu's group achieved a copper-catalyzed Heck-type coupling reaction of unactivated olefins and alkyl halides for the first time under the influence of directing groups,^9^ revealing the special interaction between copper and these directing groups (Scheme 1a). Additionally, in 2019, our group accomplished a copper-catalyzed carboamination of unactivated olefins with a carbon-amine group to construct β-lactam compounds (Scheme 1a).^10^ In 2021, Chen's group successfully achieved enantioselective synthesis of β-lactams, providing a favorable method for constructing chiral molecules (Scheme 1b).^11^ Subsequently, the Quan's group and Fu's group respectively conducted research on the directed copper-catalyzed cascade radical cyclization reaction of alkyl bromides^12^ and the different regioselective Heck-type coupling reactions of unactivated olefins with *N*-fluorosulfonamide derivatives (Scheme 1c and d).^13^ The achievements of both studies have revealed the modulation of Heck-type coupling reactions and β-lactam skeleton construction reactions under suitable reaction conditions.

Cyanide, as a unique functional group, is widely present in various natural products and pharmaceutical molecules. It possesses the capacity to effectively modulate the physicochemical properties of drugs, modify their pharmacokinetic characteristics, and enhance their bioavailability.^14^ Meanwhile, cyanide also displays a comparatively small molecular size and functions as an outstanding hydrogen bond acceptor. Additionally, it readily transforms into other functional groups, such as carboxylic acids, amides, and esters.^15^ Based on previous advances in the directed copper-catalyzed C–H functionalization of unactivated alkenes using 8-aminoquinoline as the directing group, the use of halides as radical precursors inevitably leads to the generation of halide waste, which is also detrimental to the environment.^16^ Therefore, we hypothesize whether it is possible to search for greener radical sources and, at the same time, introduce the cyanide into the molecule to achieve effective modulation of Heck-type coupling reactions or β-lactam skeleton construction reactions under some simple reaction conditions changes. This work is worth exploring and carries a certain degree of challenge.

8-Aminoquinoline serves as a nitrogen-containing directing group due to the presence of lone pair electrons on its nitrogen atom.^17^ This allows it to readily undergo precoordination with the metal copper. This coordination effectively overcomes the inherent spatial steric hindrance and electronic effects of the substrates, thereby facilitating the activation of C–H bonds and the addition of alkyl radicals to double bonds. Furthermore, through metal-catalyzed cyclization, 8-aminoquinoline forms a stable five-membered ring intermediate with neighboring C–H bonds^18^ and the copper catalyst, then the intermediates take the subsequent reactions under different conditions, leading to the synthesis of diverse target products.

## Results and discussion

**Scheme 2:**
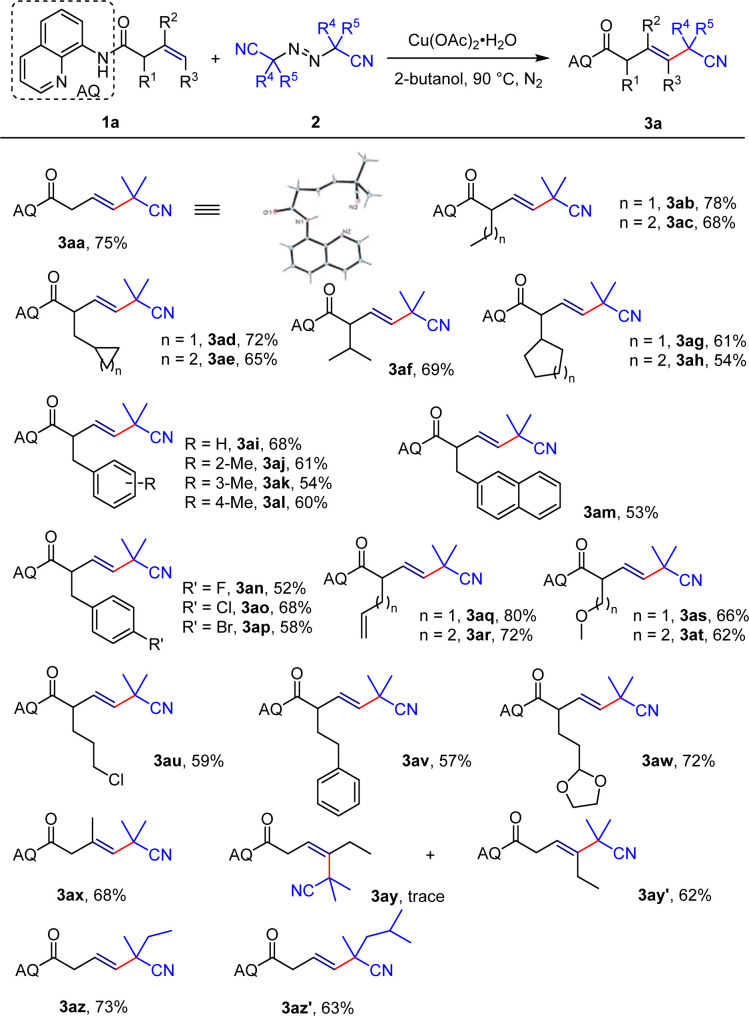
Substrate scope of heck-type coupling reaction. Reaction conditions: 1a (0.2 mmol), 2 (0.6 mmol), [Cu] (40 mol%) in solvent (1 mL) for 12 h. Isolated yield. rr or *E*/*Z* is >20 : 1 if not stated otherwise.

Based on previous research background and theoretical analysis,^18^ this study selected and optimized reaction conditions using 8-aminoquinoline or substituted 8-aminoquinoline directed unactivated olefins and commercially available, inexpensive 2,2′-azobis(2-methylpropionitrile) as template substrates. The investigation commences with the screening of copper salts, wherein commonly encountered monovalent copper salts have demonstrated effective catalytic activity towards the reaction (Table 1, entry 1–4). In particular, Cu(MeCN)_4_PF_6_ and CuOTf can only take the desired product 3 in very low yields, but can lead to the desired product 4 in good yields (Table 1, entry 3 and 4), which indicates the possibility of efficient coordination of this type of copper salt with the 8-aminoquinoline to construct β-lactam heterocycles. At the same time, we also investigated divalent copper salts and found that the target product 3 can be obtained with a separation yield of 65% in the presence of Cu(OAc)_2_·H_2_O (Table 1, entry 5–8). The enantiomeric ratio rr reaches 13 : 1 and the *E*/*Z* stereoselectivity exhibits 20 : 1, manifesting excellent regioselectivity and stereoselectivity. Additionally, the formation of trace amounts of product 4 signifies the possibility of achievement of two distinct classes of target products under appropriate conditions. After determining the copper catalyst, we screened solvents for their compatibility with the reaction. Common nonpolar solvents such as DCE and toluene decreased the yield and regioselectivity of the vinyl product 3 compared to in MeCN (Table 1, entry 9 and 10). Polar solvents DMSO, DMF, and THF were also not suitable (Table 1, entry 11–13). Surprisingly, alcohol solvents, the most commonly encountered in daily life, exhibited good compatibility with the reaction, achieving regioselectivity of >20 : 1 and stereoselectivity of >20 : 1 in these solvents (Table 1, entry 14–17). Specifically, we obtained product 3 with a separation yield of 75% using 2-butanol as the solvent, and avoided the production of allylic product 3′ and reductive elimination product 4 (Table 1, entry 18). Finally, following the guidance of Chen's research,^11^ we modified the 5-position of 8-aminoquinoline (Table 1, entry 19–21), and surprisingly, using Cu(MeCN)_4_PF_6_ as the catalyst and MeCN as the solvent, the reaction could produce β-lactam compounds 4 with a separation yield of 89% at 90 °C (Table 1, entry 21), which offers a novel approach for the construction of drug molecules containing this type of scaffold (Scheme 2).

**Table 1:** Optimization of the reaction conditions^a^

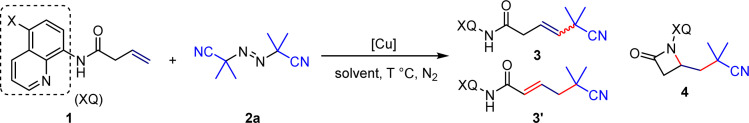								
Entry	X	[Cu]	Solvent	*T*/°C	Yield^b^ (%) of 3	Yield^b^ (%) of 4	rr (3 : 3′)	*E*/*Z*
1	H	CuCl	MeCN	90	35	32	10 : 1	>20 : 1
2	H	CuI	MeCN	90	30	38	10 : 1	>20 : 1
3	H	Cu(MeCN)_4_PF_6_	MeCN	90	Trace	67	—	—
4	H	CuOTf	MeCN	90	Trace	61	—	—
5	H	Cu(OTf)_2_	MeCN	90	Trace	63	—	—
6	H	Cu(OAc)_2_	MeCN	90	62	12	12 : 1	>20 : 1
7	H	CuBr_2_	MeCN	90	59	15	12 : 1	>20 : 1
8	H	Cu(OAc)_2_·H_2_O	MeCN	90	65	Trace	13 : 1	>20 : 1
9	H	Cu(OAc)_2_·H_2_O	DCE	90	28	41	12 : 1	>20 : 1
10	H	Cu(OAc)_2_·H_2_O	Toluene	90	32	21	5 : 1	>20 : 1
11	H	Cu(OAc)_2_·H_2_O	DMSO	90	32	Trace	8 : 1	>20 : 1
12	H	Cu(OAc)_2_·H_2_O	DMF	90	36	Trace	6 : 1	>20 : 1
13	H	Cu(OAc)_2_·H_2_O	THF	90	44	36	12 : 1	>20 : 1
14	H	Cu(OAc)_2_·H_2_O	MeOH	90	67	Trace	>20 : 1	>20 : 1
15	H	Cu(OAc)_2_·H_2_O	EtOH	90	69	Trace	>20 : 1	>20 : 1
16	H	Cu(OAc)_2_·H_2_O	^i^PrOH	90	68	Trace	>20 : 1	>20 : 1
17	H	Cu(OAc)_2_·H_2_O	2-Butanol	90	70	Trace	>20 : 1	>20 : 1
**18** c	**H**	**Cu(OAc)**_**2**_ **·H**_**2**_ **O**	**2-Butanol**	**90**	**75**	**Trace**	**>20** : **1**	**>20** : **1**
19^d^	Cl	Cu(MeCN)_4_PF_6_	MeCN	90	0	72	—	—
20^d^	Br	Cu(MeCN)_4_PF_6_	MeCN	90	0	75	—	—
**21** d	**I**	**Cu(MeCN)**_**4**_ **PF**_**6**_	**MeCN**	**90**	**0**	**89**	**—**	**—**

a Reaction conditions: 1 (0.2 mmol), 2a (0.6 mmol), [Cu] (20 mol%) in solvent (1 mL) for 12 h.

b Isolated yield.

c [Cu] (40 mol%).

d Reaction conditions: 1 (0.2 mmol), 2a (0.8 mmol), [Cu] (20 mol%) in solvent (2 mL) for 12 h.

**Scheme 3:**
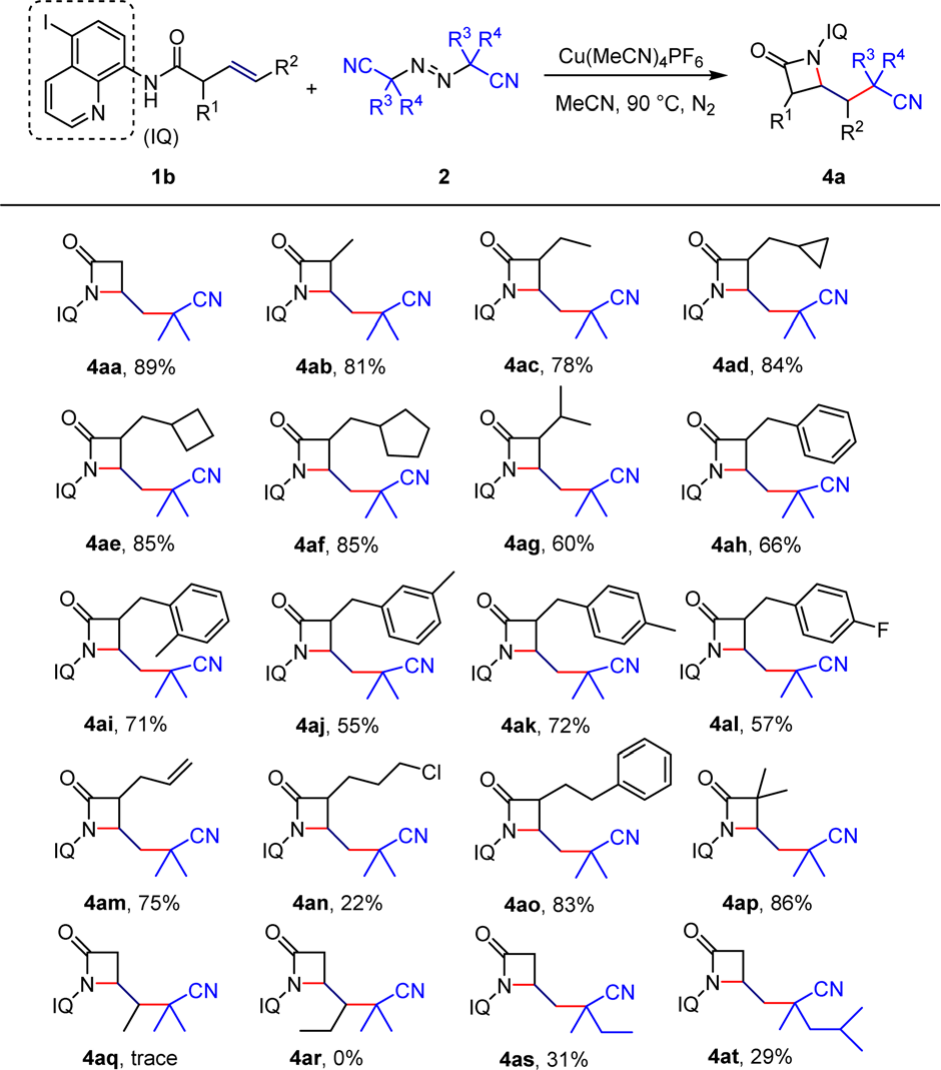
Substrate scope of β-lactams. Reaction conditions: 1b (0.2 mmol), 2 (0.8 mmol), [Cu] (20 mol%) in solvent (2 mL) for 12 h. Isolated yield.

After optimizing the conditions, we investigated the scope of substrates for the Heck-type coupling reaction. Various α-substituted enamide substrates can be successfully converted into corresponding vinyl products. The yields of the corresponding products derived from α-methyl- or α-*n*-butyl-substituted unactivated olefins are 78% and 68% (3ab–3ac) respectively. When the end of the substituent is replaced by the unstable 3-membered or 4-membered ring, the target product can also be obtained with yields of 72% and 65% (3ad–3ae). Substituting the α-position with isopropyl resulted in the target product with a separation yield of 69% (3af); however, when it is substituted with cyclopentane or cyclohexane, the yield decreases to 61% and 54% (3ag–3ah) respectively, maybe due to the steric hindrance of the cyclic structure. When the α-position is substituted with benzyl-type structures, including both electron-donating and electron-withdrawing benzyl structures and the naphthalene structure, the expected products can be obtained with moderate to good yields (3ai–3am). In particular, when the substrate is a diene structure, only the β–γ double bond can be activated, while the γ–δ and δ–ε double bonds don't participate in the reaction (3aq–3ar). Moreover, ethers, halogen atoms, aromatic rings, and aldehydes show good compatibility under the reaction condition (3as–3aw), providing the possibility of constructing more structurally diversified molecules. When the inner end of the olefin is substituted with a methyl group, the desired product can be obtained with a separation yield of 68% (3ax). Surprisingly, when the end of the olefin is substituted with an ethyl group, the *Z*-configured substrate can take the desired product with a separation yield of 62% while the *E*-configured olefin substrate can only get a trace amount of product (3ay–3ay′), which indicates that such intermolecular coupling reactions only apply to *Z*-configured olefin substrates. Finally, we investigated the application scope of diazo compounds and found that other diazo compounds also showed good compatibility (3az–3az′). The expanded substrates above displayed excellent regioselectivity and stereoselectivity with a ratio of > 20 : 1 (Scheme 3).

Furthermore, we explored the substrate scope of unactivated alkenes for the synthesis of β-lactams. Combining previous screening of reaction conditions, we chose to modify the 5-position of 8-aminoquinoline by replacing the original directing group with iodine-substituted aminoquinoline. When the α-position of the alkene was substituted with common methyl, ethyl, methylenecyclopropane, methylenecyclobutane, or methylenecyclopentane groups, the construction of β-lactams was achieved with high yields, and no open-ring products of unstable three-membered or four-membered rings were observed (4ab–4af). However, when the α-position was substituted with isopropyl, the yield decreased to 60% (4ag), indicating that steric hindrance may affect the construction of the β-lactams. By contrast, benzyl-type structures were obtained with good yields (4ah–4al), and particularly, compound 4al provided a method to introduce a fluorine atom into the molecule, which is of great significance for modifying drug molecules and changing their physiological properties. Similar to the previous Heck-type coupling, only the β–γ double bond of the diene substrate was activated, while the γ–δ double bond did not participate in the reaction (4am). The introduction of halogen atoms and aromatic groups was also compatible (4an–4ao), but the enantiomeric product of the halogen atom could only be obtained with a 22% separation yield. Substituting the α-position with dimethyl resulted in the target product with a separation yield of 86% (4ap). Furthermore, we investigated the reactivity of terminal-substituted alkene substrates and found that non-terminal alkenes were poorly compatible or even did not undergo the desired reaction (4aq–4ar). Finally, reaction attempts with different diazo compounds only yielded the target product in low yields(4as–4at).

**Scheme 4:**
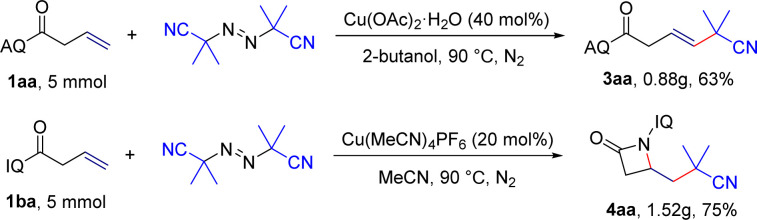
Gram-scale reactions.

To broaden the scope and applicability of the protocol, the gram-scale reactions were further performed. We found that the corresponding product 3aa and 4aa were acquired in 63% and 75% under the optimized reaction conditions (Scheme 4).

**Scheme 5:**
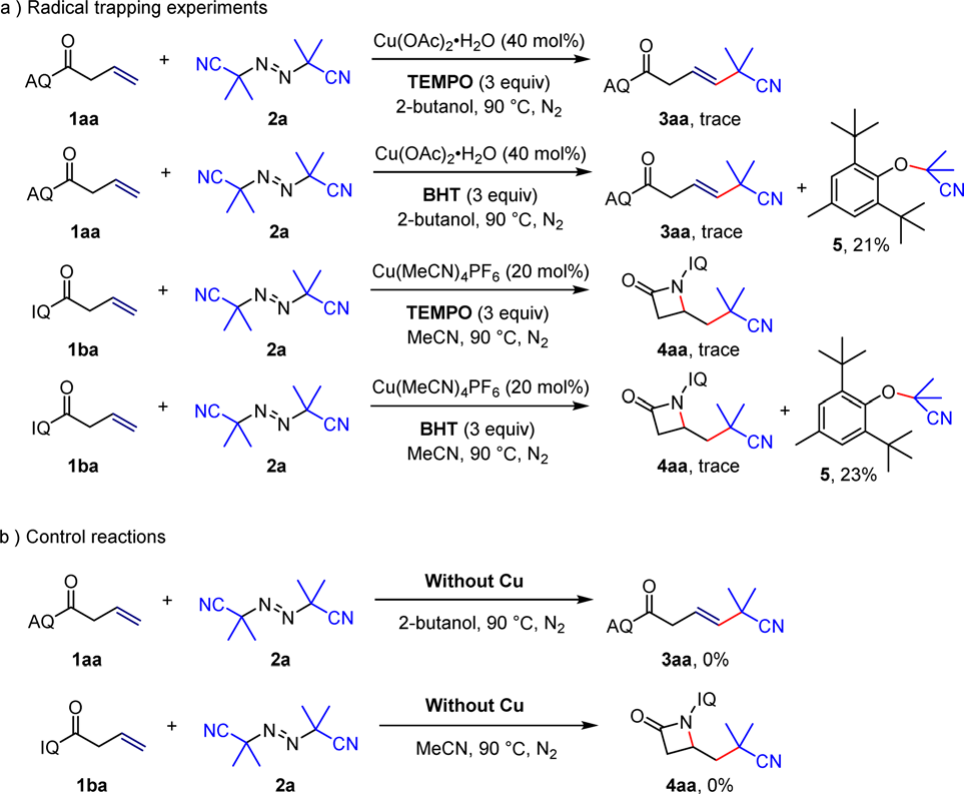
Mechanistic investigations.

In order to elucidate the reaction mechanism, radical trapping experiments and control experiments were performed. The addition of TEMPO or BHT into the reaction mixture suppressed the desired reactions and compound 5 could be isolated in the case of BHT (Scheme 5a), which suggested that this reaction involved a radical process. And then, when copper salt was not added to the reaction system (Scheme 5b), the reactions did not proceed, which showed that copper salt is essential in the reaction.

**Scheme 6:**
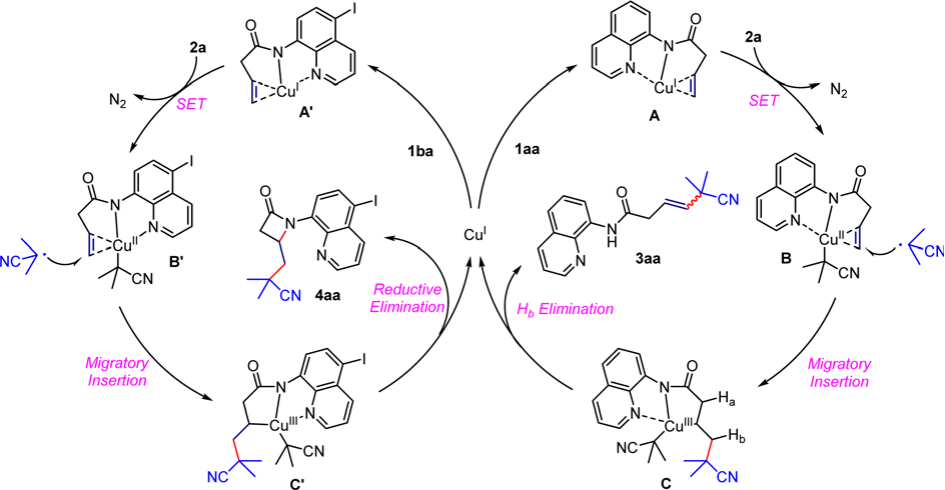
Plausible catalytic cycles.

Based on the literature and experimental results mentioned above,^1,19^ we have proposed two initial catalytic cycles for the reactions. For the Heck-type coupling reaction, substrate 1aa is activated by coordinating with Cu(i) to form a Cu(ii) intermediate B and an isobutyronitrile radical, which undergoes a single-electron transfer process. The isobutyronitrile radical migrates and inserts into the double bond to form a complex Cu(iii) intermediate C which quickly undergoes β-H elimination and demetalation in the presence of polar alcoholic solvents, producing the target product and Cu(i) for the next cycle. In the catalytic cycle for β-lactam formation, a similar Cu(i)-mediated activation of the double bond occurs through coordination with 5-iodo-8-aminoquinoline to form intermediate A′ which undergoes single-electron transfer again to form intermediate B′. The isobutyl radical migrates and inserts, generating an unstable Cu(iii) intermediate C′ which immediately undergoes intramolecular redox elimination to obtain the β-lactam skeleton and regenerate Cu(i) with catalytic activity. We hypothesize that the electronic effects of different directing groups and the ligand strength of the copper catalyst have an impact on the observed phenomenon (Scheme 6).

## Conclusions

In summary, we have successfully achieved an intermolecular free radical cascade reaction using the 8-aminoquinoline-directed copper-catalyzed strategy. We introduce the cyano groups into the molecule and, under appropriate conditions, selectively facilitate Heck-type coupling reactions or the formation of β-lactam frameworks. As a result, the functionalization of unactive C–H bonds in alkenes has been realized. This research work enhances the repertoire of strategies in organic synthesis and provides a pathway for the construction of increasingly intricate molecules.

## Data availability

The data supporting this article have been included as part of the ESI.† Crystallographic data for 3aa has been deposited at the CCDC under 2378715.

## Conflicts of interest

There are no conflicts to declare.

## Supplementary Material

RA-014-D4RA04113E-s001
