# Supplementary material for: Directed copper-catalyzed C–H functionalization of unactivated olefins with azodicarbonamide compounds
Source: RSC Adv. 2024 Aug 29;14(38):27475–80. doi: 10.1039/d4ra04113e (PMC11359497; doi:10.1039/d4ra04113e)

Supplementary Information (SI) for RSC Advances.
This journal is © The Royal Society of Chemistry 2024

Supporting Information

Directed Copper-Catalyzed C-H Functionalization of Unactivated Olefins with Azodicarbonamide Compounds

Jing Cui,[a] Xiaoya Wang[a] and Runsheng Zeng*

Table of Contents

1. General Information 1

2. Instruments 1

3. Optimization of Reaction Conditions *a* 2

4. General Procedures for the Preparation of Alkene Substrates 4

4.1 General procedure for the substrate 1aa, 1ay[21] 5

4.2 General procedure for the substrate 1ab-1aw[21] 6

4.3 General procedure for the substrate 1ax, 1ay’[20-21] 18

4.4 General procedure for amide bond formation[21-24] 20

4.5 General procedure for the substrate 1bp, 1bq, 1br[20-21] 25

4.6 General procedure for the substrate 1bb-1bo[20-21] 27

5. General Procedures for the Heck-type Coupling Reactions 36

6. General Procedures for the Synthesis of *β*-Lactams 52

7. Gram-scale Reactions 64

8. Mechanistic Experiments 65

9. X-Ray Crystallography 67

10. References 72

11. NMR Spectra 73

1. General Information

Unless otherwise noted, all reactions were performed in the oven-dried glass tubes with magnetic stirring. All reagents were purchased from commercial suppliers and used without further purification. TLC were performed on silica gel Huanghai HSGF254 plates and visualization of the developed chromatogram was performed by fluorescence quenching (λ_{max} = 254 nm). Column chromatography purifications were performed using 200 - 300 and 300 - 400 mesh silica gel.

2. Instruments

NMR spectra were recorded on Varian Inova-400 MHz, Inova-300 MHz, Bruker DRX-400 or Bruker DRX-500 instruments and chemical shifts were quoted in parts per million (ppm) referenced to the appropriate solvent peak. Multiplicities are recorded as: s = singlet, d = doublet, t = triplet, q = quartet, dd = doublet of doublets, dt = doublet of triplets, td = triplet of doublet, ddd = doublet of doublet of doublet, m = multiplet. Coupling constants, *J*, were reported in hertz unit (Hz). HRMS analysis were carried out using a Bruker micro TOF-Q instrument or a TOF-MS instrument. Melting point were recorded on X-4 micro melting point instrument. X-ray was carried out using the D8 VENTURE single crystal instrument.

3. Optimization of Reaction Conditions [a]

Table S1. Optimization Conditions of the Heck-Type Reaction

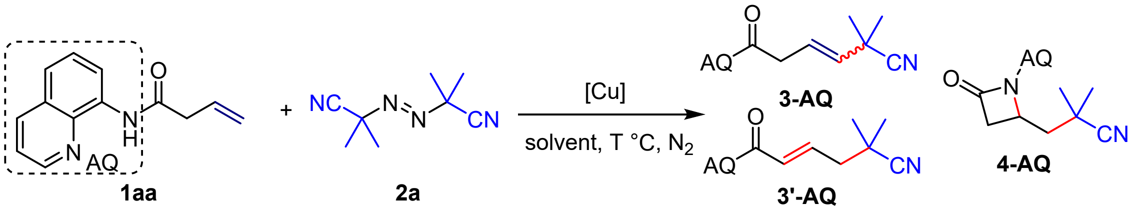

entry	[Cu]	solvent	T/°C	yield (%) [b] of **3-AQ**	yield (%) [b] bof **4-AQ**	*rr* (**3-AQ**:**3'-AQ**)	*E/Z*
1	CuCl	MeCN	90	35	32	10:1	>20:1
2	CuI	MeCN	90	30	38	10:1	>20:1
3	$Cu(MeCN)_4PF_6$	MeCN	90	trace	67	--	--
4	CuOTf	MeCN	90	trace	61	--	--
5	$Cu(OTf)_2$	MeCN	90	trace	63	--	--
6	$Cu(OAc)_2$	MeCN	90	62	12	12:1	>20:1
7	$CuBr_2$	MeCN	90	59	15	12:1	>20:1
8	$Cu(OAc)_2{\cdot}H_2O$	MeCN	90	65	trace	13:1	>20:1
9	$Cu(OAc)_2{\cdot}H_2O$	DCE	90	28	41	12:1	>20:1
10	$Cu(OAc)_2{\cdot}H_2O$	toluene	90	32	21	5:1	>20:1
11	$Cu(OAc)_2{\cdot}H_2O$	DMSO	90	32	trace	8:1	>20:1
12	$Cu(OAc)_2{\cdot}H_2O$	DMF	90	36	trace	6:1	>20:1
13	$Cu(OAc)_2{\cdot}H_2O$	THF	90	44	36	12:1	>20:1
14	$Cu(OAc)_2{\cdot}H_2O$	MeOH	90	67	trace	>20:1	>20:1
15	$Cu(OAc)_2{\cdot}H_2O$	EtOH	90	69	trace	>20:1	>20:1
16	$Cu(OAc)_2{\cdot}H_2O$	^{i}PrOH	90	68	trace	>20:1	>20:1
17	$Cu(OAc)_2{\cdot}H_2O$	2-butanol	90	70	trace	>20:1	>20:1
**18**[c]	$\mathbf{Cu(OAc)_2{\cdot}H_2O}$	**2-butanol**	**90**	**75**	**trace**	**>20:1**	**>20:1**
19[c]	$Cu(OAc)_2{\cdot}H_2O$	2-butanol	80	70	trace	>20:1	>20:1
20[c]	$Cu(OAc)_2{\cdot}H_2O$	2-butanol	100	66	trace	>20:1	>20:1

[a] Reaction conditions: **1aa** (0.2 mmol), **2a** (0.6 mmol), [Cu] (20 mol%) in solvent (1 mL) for 12 h. [b] Isolated yield. [c] [Cu] (40 mol%).

Table S2. Optimization Conditions of the Synthesis of β-Lactams [a]

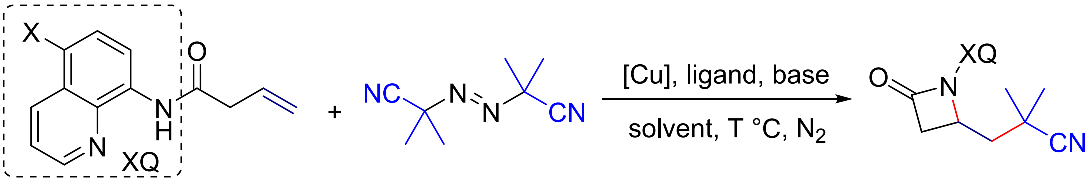

1 2a 4-XQ

entry	X	[Cu]	ligand	base	solvent	T/°C	yield (%) [b] of **4-XQ**
1	I	$Cu(MeCN)_4PF_6$	--	--	1,4-dioxane	110	39
2	I	$Cu(OTf)_2$	--	--	1,4-dioxane	110	28
3	I	CuOTf	--	--	1,4-dioxane	110	20
4	I	CuI	--	--	1,4-dioxane	110	trace
5	I	$Cu(MeCN)_4PF_6$	--	--	DCE	110	15
6	I	$Cu(MeCN)_4PF_6$	--	--	DCM	110	24
7	I	$Cu(MeCN)_4PF_6$	--	--	*t*-BuOH	110	trace
8	I	$Cu(MeCN)_4PF_6$	--	--	CH_3CN	110	49
9[c]	I	$Cu(MeCN)_4PF_6$	--	--	CH_3CN	110	61
10[d]	I	$Cu(MeCN)_4PF_6$	--	--	CH_3CN	110	70
11[d]	I	$Cu(MeCN)_4PF_6$	--	--	CH_3CN	100	72
**12[d]**	**I**	**$Cu(MeCN)_4PF_6$**	--	--	**CH_3CN**	**90**	**89**
13[d]	I	$Cu(MeCN)_4PF_6$	--	--	CH_3CN	80	80
14[d]	H	$Cu(MeCN)_4PF_6$	--	--	CH_3CN	90	70
15[d]	Cl	$Cu(MeCN)_4PF_6$	--	--	CH_3CN	90	72
16[d]	Br	$Cu(MeCN)_4PF_6$	--	--	CH_3CN	90	75
17[d]	I	$Cu(MeCN)_4PF_6$	phen	--	CH_3CN	90	71
18[d]	I	$Cu(MeCN)_4PF_6$	bpy	--	CH_3CN	90	66
19[d]	I	$Cu(MeCN)_4PF_6$	BINAP	--	CH_3CN	90	73
20[d]	I	$Cu(MeCN)_4PF_6$	--	Et_3N	CH_3CN	90	54
21[d]	I	$Cu(MeCN)_4PF_6$	--	K_2CO_3	CH_3CN	90	35
22[d]	I	$Cu(MeCN)_4PF_6$	--	Na_2CO_3	CH_3CN	90	32

[a] Reaction conditions: **1** (0.2 mmol), **2a** (0.4 mmol), [Cu] (20 mol%) in solvent (2 mL) for 12 h. [b] Isolated yield. [c] **2a** (0.6 mmol). [d] **2a** (0.8 mmol). Ligand (0.4 mmol). Base (0.4 mmol).

4. General Procedures for the Preparation of Alkene Substrates

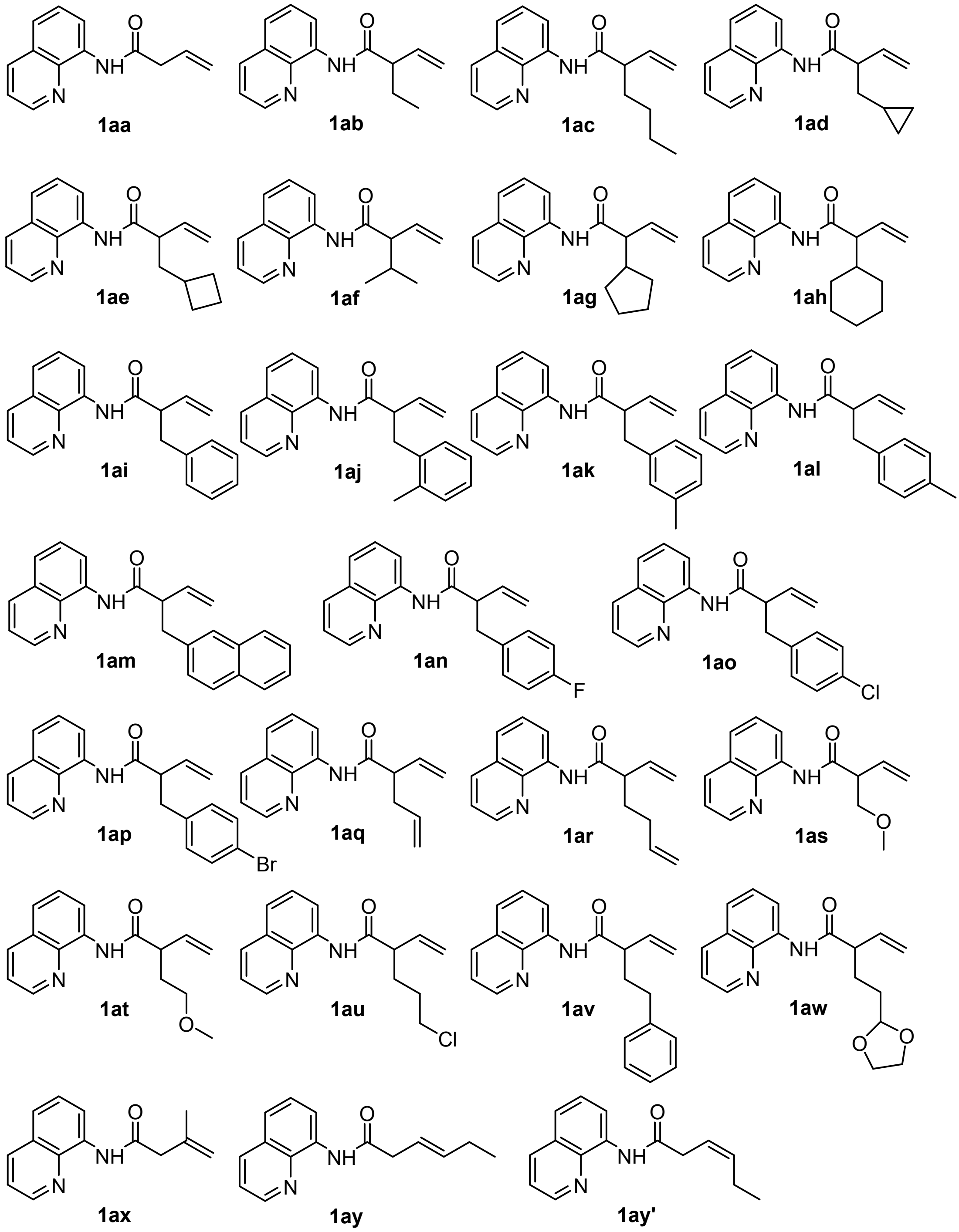

Scheme S1. Alkene Substrates (AQ-Directed, Yields are unoptimized)

4.1 General procedure for the substrate 1aa, 1ay[21]

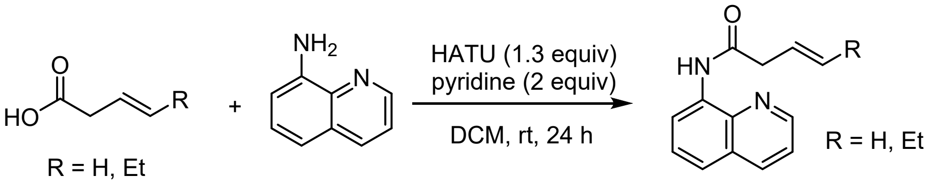

Scheme S2. Synthesis of substrate 1aa, 1ay

Vinyl acetic acid or *γ*-substituted vinyl acetic acid (12 mmol, 1.2 equiv, from commercial sources) was charged into a 150 mL flask containing 30 mL DCM. 8-Aminoquinoline (1.44 g, 10 mmol, 1 equiv), pyridine (2.6 mL, 20 mmol, 2 equiv), and HATU (4.94 g, 13 mmol, 1.3 equiv) were added sequentially, and the reaction was stirred at room temperature for 24 h. The deep brown solution was diluted with DCM (100 mL), washed with sat. $NaHCO_3$ (100 mL, ×2) and brine (100 mL, ×2). The organic layer was evaporated under reduced pressure and the residue was purified by column chromatography on silica gel (200 - 300 mesh), eluting with 10%-20% EtOAc/petroleum ether to give the desire product.

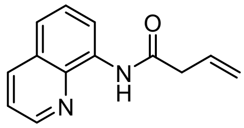

1aa

R_f = 0.5, Petroleum Ether: Ethyl Acetate = 5: 1 (v/v)

Compound **1aa** was prepared following the general procedure in 89% yield (1.89 g) as a yellow oil.

^{1}H NMR (400 MHz, Chloroform-*d*) δ 9.93 (s, 1H), 8.78 – 8.68 (m, 2H), 8.07 (dd, *J* = 8.2, 1.7 Hz, 1H), 7.50 – 7.40 (m, 2H), 7.37 (dd, *J* = 8.3, 4.2 Hz, 1H), 6.12 (ddt, *J* = 17.2, 10.2, 7.1 Hz, 1H), 5.40 – 5.26 (m, 2H), 3.32 (dt, *J* = 7.1, 1.3 Hz, 2H).

^{13}C NMR (101 MHz, Chloroform-*d*) δ 169.23, 148.19, 138.39, 136.28, 134.33, 130.98, 127.87, 127.29, 121.59, 121.56, 120.02, 116.42, 43.14.

HRMS (ESI-TOF): m/z Calcd for $C_{13}H_{13}N_2O^+$ [M+H$^+$]: 213.1022; found: 213.1029.

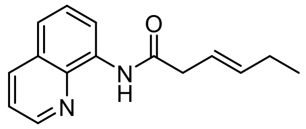

1ay

R_f = 0.4, Petroleum Ether: Ethyl Acetate = 10: 1 (v/v)

Compound **1ay** was prepared following the general procedure in 80% yield (1.92 g) as a yellow oil.

^{1}H NMR (400 MHz, Chloroform-*d*) δ 10.08 (s, 1H), 8.81 – 8.72 (m, 2H), 8.12 (dd, *J* = 8.3, 1.7 Hz, 1H), 7.55 – 7.44 (m, 2H), 7.41 (dd, *J* = 8.3, 4.2 Hz, 1H), 5.91 – 5.81 (m, 1H), 5.73 (dtt, *J* = 15.5, 7.1, 1.4 Hz, 1H), 3.26 (dq, *J* = 7.1, 1.1 Hz, 2H), 2.18 (qdd, *J* = 7.5, 6.3, 1.3 Hz, 2H), 1.11 (t, *J* = 7.5 Hz, 3H).

^{13}C NMR (101 MHz, Chloroform-*d*) δ 170.19, 148.17, 138.76, 138.58, 136.39, 134.53, 128.00, 127.46, 121.61, 121.56, 121.46, 116.42, 42.16, 25.87, 13.65.

HRMS (ESI-TOF): m/z Calcd for $C_{15}H_{17}N_2O^+$ [M+H$^+$]: 241.1335; found: 241.1338.

4.2 General procedure for the substrate 1ab-1aw[21]

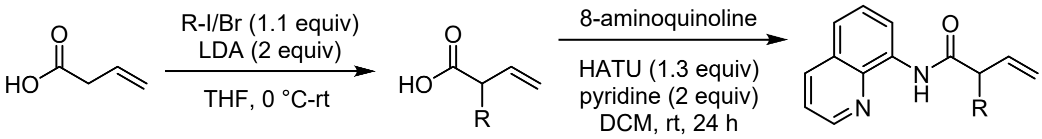

Scheme S3. Synthesis of substrate 1ab-1aw

Step1: A solution of commercial LDA 2 M (11.8 mL, 23.5 mmol, 2 equiv) in THF was cooled to ice-water temperature and a solution of 3-butenoic acid (1 mL, 11.77 mmol, 1 equiv) in 10 mL of THF was added slowly over a period of 15 min. The resulting mixture was stirred at the 0°C for 45 min to obtain a deep yellow solution. A total of R-I/Br (12.9 mmol, 1.1 equiv) of the alkylating agent was added, whereupon the reaction mixture immediately turned colorless. After 30 min at the same temperature and 3 h at room temperature, the pH of the solution was adjusted to 2.5 with 10% HCl. The organic phase was separated. The aqueous layer was saturated with solid NaCl and the mixture was extracted with DCM. The combined organic layers were dried over anhydrous Na_2SO_4, filtered and concentrated *in vacuo* to obtain the *α*-substituted 3-butenoic acid.

Step 2: *α*-Substituted vinyl acetic acid (12 mmol, 1.2 equiv) was charged into a 150

mL flask containing 30 mL DCM. 8-Aminoquinoline (1.44 g, 10 mmol, 1 equiv), pyridine (2.6 mL, 20 mmol, 2 equiv), and HATU (4.94 g, 13 mmol, 1.3 equiv) were added sequentially, and the reaction was stirred at room temperature for 24 h. The deep brown solution was diluted with DCM (100 mL), washed with sat. $NaHCO_3$ (100 mL, ×2) and brine (100 mL, ×2). The organic layer was evaporated under reduced pressure and the residue was purified by column chromatography on silica gel (200 - 300 mesh), eluting with 5%-20% EtOAc/petroleum ether to give the desire product.

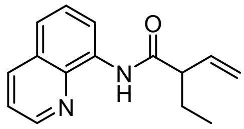

1ab

R_f = 0.4, Petroleum Ether: Ethyl Acetate = 15: 1 (v/v)

Compound **1ab** was prepared following the general procedure in 35% yield (0.83 g) as a yellow oil.

^{1}H NMR (400 MHz, Chloroform-*d*) δ 9.87 (s, 1H), 8.74 – 8.62 (m, 2H), 7.98 (dt, *J* = 8.3, 1.6 Hz, 1H), 7.43 – 7.31 (m, 2H), 7.29 (ddd, *J* = 8.3, 4.2, 1.5 Hz, 1H), 5.91 (ddd, *J* = 17.1, 10.2, 8.7 Hz, 1H), 5.31 – 5.12 (m, 2H), 3.05 – 2.92 (m, 1H), 1.93 (ddd, *J* = 14.0, 7.5, 6.6 Hz, 1H), 1.70 – 1.54 (m, 1H), 0.90 (t, *J* = 7.5 Hz, 3H).

^{13}C NMR (101 MHz, Chloroform-*d*) δ 172.12, 148.21, 138.50, 136.82, 136.28, 134.49, 127.91, 127.33, 121.56, 121.50, 118.15, 116.39, 55.09, 25.12, 11.82.

HRMS (ESI-TOF): m/z Calcd for $C_{15}H_{16}N_2NaO^+$ [M+Na$^+$]: 263.1155; found: 263.1162.

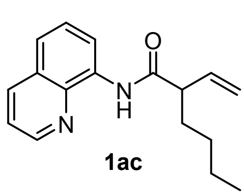

1ac

R_f = 0.5, Petroleum Ether: Ethyl Acetate = 10: 1 (v/v)

Compound **1ac** was prepared following the general procedure in 27% yield (0.72 g) as a yellow oil.

^{1}H NMR (400 MHz, Chloroform-*d*) δ 9.97 (s, 1H), 8.83 – 8.72 (m, 2H), 8.10 (dd, *J* = 8.3, 1.7 Hz, 1H), 7.54 – 7.42 (m, 2H), 7.40 (dd, *J* = 8.3, 4.2 Hz, 1H), 6.02 (ddd, *J* =

17.2, 10.1, 8.6 Hz, 1H), 5.41 – 5.21 (m, 2H), 3.17 (q, *J* = 7.7 Hz, 1H), 2.05 – 1.94 (m, 1H), 1.75 – 1.61 (m, 1H), 1.47 – 1.26 (m, 4H), 0.99 – 0.80 (m, 3H).

^{13}C NMR (101 MHz, Chloroform-*d*) δ 172.26, 148.25, 138.56, 137.17, 136.32, 134.56, 127.95, 127.39, 121.59, 121.51, 117.95, 116.43, 53.55, 31.71, 29.47, 22.64, 14.03.

HRMS (ESI-TOF): m/z Calcd for $C_{17}H_{20}N_2NaO^+$ [M+Na$^+$]: 291.1468; found: 291.1470.

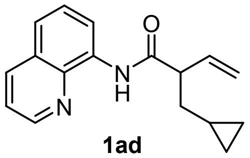

1ad

R$_f$ = 0.5, Petroleum Ether: Ethyl Acetate = 15: 1 (v/v)

Compound **1ad** was prepared following the general procedure in 36% yield (0.95 g) as a yellow oil.

^{1}H NMR (400 MHz, Chloroform-*d*) δ 9.98 (s, 1H), 8.76 (ddt, *J* = 17.8, 6.0, 2.0 Hz, 2H), 8.02 (td, *J* = 8.5, 1.8 Hz, 1H), 7.49 – 7.36 (m, 2H), 7.33 (td, *J* = 8.1, 4.3 Hz, 1H), 6.06 (dddd, *J* = 17.1, 10.2, 9.2, 2.6 Hz, 1H), 5.40 – 5.18 (m, 2H), 3.29 (qd, *J* = 7.3, 2.6 Hz, 1H), 1.81 (dtd, *J* = 14.3, 7.3, 2.5 Hz, 1H), 1.65 (dtd, *J* = 13.8, 6.9, 3.0 Hz, 1H), 0.77 (qdt, *J* = 11.3, 7.8, 4.0 Hz, 1H), 0.49 – 0.32 (m, 2H), 0.10 (dq, *J* = 5.1, 3.7 Hz, 2H).

^{13}C NMR (101 MHz, Chloroform-*d*) δ 171.98, 148.10, 137.00, 136.14, 134.41, 127.78, 127.20, 121.44, 121.39, 117.65, 116.26, 53.81, 37.22, 8.97, 4.68, 4.57.

HRMS (ESI-TOF): m/z Calcd for $C_{17}H_{18}N_2NaO^+$ [M+Na$^+$]: 289.1311; found: 289.1316.

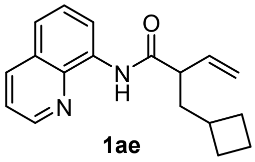

1ae

R$_f$ = 0.3, Petroleum Ether: Ethyl Acetate = 10: 1 (v/v)

Compound **1ae** was prepared following the general procedure in 38% yield (1.05 g) as a yellow oil.

^{1}H NMR (400 MHz, Chloroform-*d*) δ 9.94 (s, 1H), 8.85 – 8.73 (m, 2H), 8.11 (dd, *J* = 8.3, 1.7 Hz, 1H), 7.53 – 7.43 (m, 2H), 7.41 (dd, *J* = 8.3, 4.2 Hz, 1H), 6.01 (ddd, *J* = 17.1, 10.1, 8.6 Hz, 1H), 5.37 – 5.21 (m, 2H), 3.12 (q, *J* = 7.7 Hz, 1H), 2.41 (hept, *J* = 7.9 Hz, 1H), 2.15 – 1.99 (m, 3H), 1.86 – 1.74 (m, 3H), 1.69 (tt, *J* = 10.9, 8.4 Hz, 2H).

^{13}C NMR (101 MHz, Chloroform-*d*) δ 172.21, 148.26, 138.56, 137.17, 136.37, 134.57, 127.99, 127.43, 121.61, 121.53, 117.72, 116.48, 51.77, 39.29, 34.00, 28.59, 28.32, 18.55.

HRMS (ESI-TOF): m/z Calcd for $C_{18}H_{20}N_2NaO^+$ [M+Na$^+$]: 303.1468; found: 303.1474.

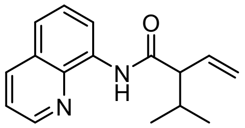

1af

R$_f$ = 0.5, Petroleum Ether: Ethyl Acetate = 10: 1 (v/v)

Compound **1af** was prepared following the general procedure in 32% yield (0.82 g) as a yellow oil.

^{1}H NMR (400 MHz, Chloroform-*d*) δ 9.91 (s, 1H), 8.87 – 8.69 (m, 2H), 8.06 (dt, *J* = 8.3, 1.5 Hz, 1H), 7.51 – 7.40 (m, 2H), 7.37 (ddd, *J* = 8.3, 4.2, 1.2 Hz, 1H), 6.03 (ddd, *J* = 17.0, 10.2, 9.4 Hz, 1H), 5.34 – 5.21 (m, 2H), 2.93 – 2.78 (m, 1H), 2.33 – 2.20 (m, 1H), 1.03 (d, *J* = 6.7 Hz, 3H), 0.98 (d, *J* = 6.7 Hz, 3H).

^{13}C NMR (101 MHz, Chloroform-*d*) δ 172.06, 148.17, 138.44, 136.25, 135.75, 134.45, 127.88, 127.31, 121.54, 121.47, 118.73, 116.39, 61.37, 30.30, 21.02, 19.71.

HRMS (ESI-TOF): m/z Calcd for $C_{16}H_{18}N_2NaO^+$ [M+Na$^+$]: 277.1311; found: 277.1317.

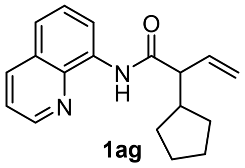

1ag

R$_f$ = 0.5, Petroleum Ether: Ethyl Acetate = 10: 1 (v/v)

Compound **1ag** was prepared following the general procedure in 17% yield (0.48 g) as a yellow oil.

^{1}H NMR (400 MHz, Chloroform-*d*) δ 9.90 (s, 1H), 8.84 – 8.74 (m, 2H), 8.14 (dd, *J* = 8.2, 1.7 Hz, 1H), 7.55 – 7.46 (m, 2H), 7.44 (dd, *J* = 8.3, 4.2 Hz, 1H), 6.05 (ddd, *J* = 17.1, 10.2, 9.0 Hz, 1H), 5.37 – 5.16 (m, 2H), 2.97 (t, *J* = 9.2 Hz, 1H), 2.42 (qt, *J* = 9.0, 7.4 Hz, 1H), 1.93 – 1.77 (m, 2H), 1.71 – 1.49 (m, 4H), 1.38 – 1.25 (m, 2H).

^{13}C NMR (101 MHz, Chloroform-*d*) δ 172.36, 148.29, 138.58, 136.76, 136.47, 134.63, 128.05, 127.53, 121.67, 121.54, 117.77, 116.58, 59.74, 42.11, 31.11, 30.50, 25.32, 25.12.

HRMS (ESI-TOF): m/z Calcd for $C_{18}H_{21}N_2O^+$ [M+H$^+$]: 281.1648; found: 281.1652.

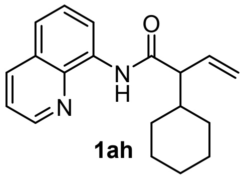

1ah

R$_f$ = 0.5, Petroleum Ether: Ethyl Acetate = 10: 1 (v/v)

Compound **1ah** was prepared following the general procedure in 12% yield (0.35 g) as a yellow oil.

^{1}H NMR (400 MHz, Chloroform-*d*) δ 9.89 (s, 1H), 8.86 – 8.75 (m, 2H), 8.13 (dd, *J* = 8.3, 1.7 Hz, 1H), 7.55 – 7.45 (m, 2H), 7.43 (dd, *J* = 8.2, 4.2 Hz, 1H), 6.03 (ddd, *J* = 17.0, 10.2, 9.4 Hz, 1H), 5.32 – 5.21 (m, 2H), 2.89 (t, *J* = 8.9 Hz, 1H), 2.00 – 1.89 (m, 1H), 1.89 – 1.79 (m, 2H), 1.77 – 1.60 (m, 3H), 1.35 – 1.22 (m, 2H), 1.20 – 1.05 (m, 2H), 0.97 (tdd, *J* = 12.6, 11.4, 3.5 Hz, 1H).

^{13}C NMR (101 MHz, Chloroform-*d*) δ 172.19, 148.28, 138.56, 136.41, 136.03, 134.54, 128.02, 127.48, 121.65, 121.56, 118.50, 116.52, 60.82, 39.81, 31.56, 30.33, 26.45, 26.29.

HRMS (ESI-TOF): m/z Calcd for $C_{19}H_{23}N_2O^+$ [M+H$^+$]: 295.1805; found: 295.1812.

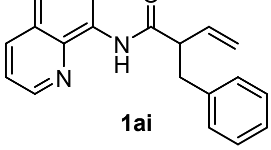

1ai

R$_f$ = 0.5, Petroleum Ether: Ethyl Acetate = 5: 1 (v/v)

Compound **1ai** was prepared following the general procedure in 21% yield (0.62 g) as a yellow oil.

^{1}H NMR (400 MHz, Chloroform-*d*) δ 9.93 (s, 1H), 8.88 – 8.68 (m, 2H), 8.06 (dd, *J* = 8.3, 1.7 Hz, 1H), 7.52 – 7.42 (m, 2H), 7.36 (dd, *J* = 8.3, 4.3 Hz, 1H), 7.29 – 7.22 (m, 4H), 7.15 (ddt, *J* = 8.6, 5.5, 2.7 Hz, 1H), 6.06 (ddd, *J* = 17.2, 10.2, 8.5 Hz, 1H), 5.33 – 5.22 (m, 2H), 3.57 – 3.46 (m, 1H), 3.39 (dd, *J* = 13.7, 6.9 Hz, 1H), 2.99 (dd, *J* = 13.7, 7.6 Hz, 1H).

^{13}C NMR (101 MHz, Chloroform-*d*) δ 171.26, 148.19, 139.19, 138.48, 136.35, 136.28, 134.42, 129.26, 127.91, 127.35, 126.33, 121.62, 121.57, 118.71, 116.47, 55.14, 38.16.

HRMS (ESI-TOF): m/z Calcd for $C_{20}H_{18}N_2NaO^+$ [M+Na$^+$]: 325.1311; found: 325.1307.

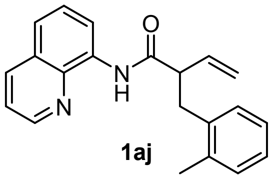

1aj

R$_f$ = 0.4, Petroleum Ether: Ethyl Acetate = 20: 1 (v/v)

Compound **1aj** was prepared following the general procedure in 30% yield (0.95 g) as a yellow oil.

^{1}H NMR (400 MHz, Chloroform-*d*) δ 9.94 (s, 1H), 8.83 – 8.74 (m, 2H), 8.13 (dd, *J* = 8.3, 1.7 Hz, 1H), 7.57 – 7.46 (m, 2H), 7.42 (dd, *J* = 8.3, 4.2 Hz, 1H), 7.25 – 7.19 (m, 1H), 7.18 – 7.13 (m, 1H), 7.12 – 7.06 (m, 2H), 6.11 (ddd, *J* = 17.3, 9.9, 8.4 Hz, 1H), 5.34 – 5.19 (m, 2H), 3.59 – 3.34 (m, 2H), 2.99 (dd, *J* = 13.7, 7.4 Hz, 1H), 2.40 (s, 3H).

^{13}C NMR (101 MHz, Chloroform-*d*) δ 171.49, 148.27, 138.57, 137.49, 136.49, 136.38, 134.51, 130.39, 129.94, 128.00, 127.45, 126.50, 125.93, 121.67, 121.65, 118.66, 116.55, 54.06, 35.32, 19.78.

HRMS (ESI-TOF): m/z Calcd for $C_{21}H_{20}N_2NaO^+$ [M+Na$^+$]: 339.1468; found: 339.1475.

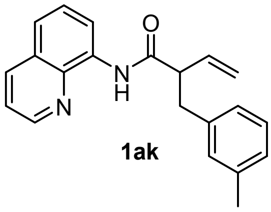

1ak

R_f = 0.6, Petroleum Ether: Ethyl Acetate = 10: 1 (v/v)

Compound **1ak** was prepared following the general procedure in 17% yield (0.53 g) as a yellow oil.

^{1}H NMR (400 MHz, Chloroform-*d*) δ 9.95 (s, 1H), 8.83 (dd, *J* = 7.6, 1.4 Hz, 1H), 8.75 (dd, *J* = 4.3, 1.7 Hz, 1H), 8.09 (dd, *J* = 8.2, 1.8 Hz, 1H), 7.56 – 7.43 (m, 2H), 7.39 (dd, *J* = 8.2, 4.2 Hz, 1H), 7.17 (t, *J* = 7.4 Hz, 1H), 7.13 – 7.06 (m, 2H), 6.99 (d, *J* = 7.4 Hz, 1H), 6.10 (ddd, *J* = 17.2, 10.2, 8.5 Hz, 1H), 5.40 – 5.24 (m, 2H), 3.54 (q, *J* = 7.6 Hz, 1H), 3.39 (dd, *J* = 13.7, 6.9 Hz, 1H), 2.99 (dd, *J* = 13.7, 7.6 Hz, 1H), 2.30 (s, 3H).

^{13}C NMR (101 MHz, Chloroform-*d*) δ 171.35, 148.14, 139.05, 138.44, 137.84, 136.42, 136.24, 134.39, 130.01, 128.25, 127.87, 127.31, 127.07, 126.23, 121.56, 121.52, 118.56, 116.44, 55.10, 38.13, 21.39.

HRMS (ESI-TOF): m/z Calcd for $C_{21}H_{20}N_2NaO^+$ [M+Na$^+$]: 339.1468; found: 339.1472.

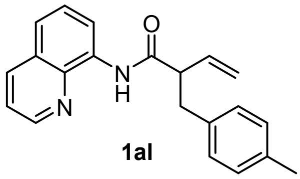

1al

R_f = 0.6, Petroleum Ether: Ethyl Acetate = 10: 1 (v/v)

Compound **1al** was prepared following the general procedure in 31% yield (0.97 g) as a yellow oil.

^{1}H NMR (400 MHz, Chloroform-*d*) δ 9.96 (s, 1H), 8.83 (dd, *J* = 7.5, 1.5 Hz, 1H), 8.75 (dd, *J* = 4.2, 1.7 Hz, 1H), 8.09 (dd, *J* = 8.2, 1.7 Hz, 1H), 7.56 – 7.44 (m, 2H), 7.39 (dd, *J* = 8.3, 4.2 Hz, 1H), 7.23 – 7.14 (m, 2H), 7.08 (d, *J* = 7.8 Hz, 2H), 6.09 (ddd, *J* = 17.2, 10.2, 8.5 Hz, 1H), 5.41 – 5.22 (m, 2H), 3.58 – 3.47 (m, 1H), 3.38 (dd, *J* = 13.8, 6.9 Hz, 1H), 2.99 (dd, *J* = 13.8, 7.6 Hz, 1H), 2.29 (s, 3H).

^{13}C NMR (101 MHz, Chloroform-*d*) δ 171.35, 148.15, 138.46, 136.44, 136.24, 136.03, 135.72, 134.43, 129.10, 129.07, 127.88, 127.32, 121.57, 121.54, 118.60, 116.44, 55.21, 37.75, 21.05.

HRMS (ESI-TOF): m/z Calcd for $C_{21}H_{20}N_2NaO^+$ [M+Na$^+$]: 339.1468; found: 339.1473.

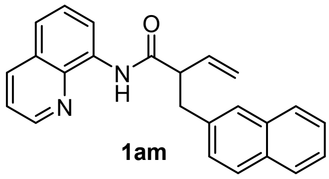

1am

R_f = 0.3, Petroleum Ether: Ethyl Acetate = 10: 1 (v/v)

Compound **1am** was prepared following the general procedure in 40% yield (1.41 g) as a yellow solid. m.p. 73-75 °C.

^{1}H NMR (400 MHz, Chloroform-*d*) δ 9.95 (s, 1H), 8.81 (dd, *J* = 7.5, 1.5 Hz, 1H), 8.68 (dd, *J* = 4.3, 1.7 Hz, 1H), 8.10 (dd, *J* = 8.3, 1.7 Hz, 1H), 7.81 – 7.70 (m, 4H), 7.56 – 7.45 (m, 2H), 7.45 – 7.35 (m, 4H), 6.12 (ddd, *J* = 17.1, 10.2, 8.4 Hz, 1H), 5.36 – 5.23 (m, 2H), 3.68 – 3.49 (m, 2H), 3.17 (dd, *J* = 13.4, 7.3 Hz, 1H).

^{13}C NMR (101 MHz, Chloroform-*d*) δ 171.34, 148.21, 138.53, 136.81, 136.37, 136.34, 134.46, 133.65, 132.32, 128.05, 127.98, 127.79, 127.67, 127.42, 125.96, 125.40, 121.70, 121.61, 118.90, 116.61, 55.17, 38.43.

HRMS (ESI-TOF): m/z Calcd for $C_{24}H_{21}N_2O^+$ [M+H$^+$]: 353.1648; found: 353.1643.

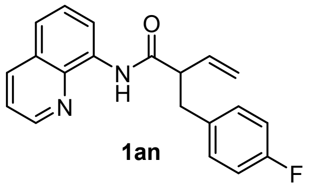

1an

R_f = 0.4, Petroleum Ether: Ethyl Acetate = 20: 1 (v/v)

Compound **1an** was prepared following the general procedure in 31% yield (1.00 g) as a white solid. m.p. 128-130 °C.

^{1}H NMR (400 MHz, Chloroform-*d*) δ 9.92 (s, 1H), 8.79 (dd, *J* = 7.5, 1.5 Hz, 1H), 8.74 (dd, *J* = 4.3, 1.7 Hz, 1H), 8.09 (dt, *J* = 8.3, 1.3 Hz, 1H), 7.54 – 7.43 (m, 2H), 7.41 – 7.35 (m, 1H), 7.25 – 7.17 (m, 2H), 6.99 – 6.87 (m, 2H), 6.13 – 5.98 (m, 1H), 5.35 – 5.23 (m, 2H), 3.46 (q, *J* = 7.5 Hz, 1H), 3.34 (dd, *J* = 13.8, 7.0 Hz, 1H), 2.96 (dd, *J* = 13.8, 7.4 Hz, 1H).

^{13}**C NMR** (101 MHz, Chloroform-*d*) δ 171.04, 162.78, 160.35, 148.22, 138.46, 136.30, 136.17, 134.85, 134.82, 134.34, 130.73, 130.65, 127.92, 127.32, 121.70, 121.61, 118.85, 116.48, 115.25, 115.04, 55.25, 37.35.

^{19}**F NMR** (376 MHz, Chloroform-*d*) δ -116.92.

HRMS (ESI-TOF): m/z Calcd for $C_{20}H_{17}FN_2NaO^+$ [M+Na$^+$]: 343.1217; found: 343.1223.

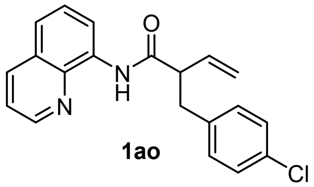

1ao

R$_f$ = 0.4, Petroleum Ether: Ethyl Acetate = 15: 1 (v/v)

Compound **1ao** was prepared following the general procedure in 15% yield (0.52 g) as a white solid. m.p. 126-128 °C.

^{1}**H NMR** (400 MHz, Chloroform-*d*) δ 9.92 (s, 1H), 8.83 – 8.70 (m, 2H), 8.11 (dd, *J* = 8.3, 1.7 Hz, 1H), 7.56 – 7.45 (m, 2H), 7.41 (dd, *J* = 8.3, 4.2 Hz, 1H), 7.24 – 7.13 (m, 4H), 6.14 – 5.90 (m, 1H), 5.35 – 5.21 (m, 2H), 3.46 (q, *J* = 7.6 Hz, 1H), 3.34 (dd, *J* = 13.8, 7.0 Hz, 1H), 2.95 (dd, *J* = 13.7, 7.4 Hz, 1H).

^{13}**C NMR** (101 MHz, Chloroform-*d*) δ 170.94, 148.28, 138.49, 137.69, 136.36, 136.06, 134.33, 132.14, 130.67, 128.53, 127.96, 127.37, 121.77, 121.66, 119.05, 116.54, 55.05, 37.48.

HRMS (ESI-TOF): m/z Calcd for $C_{20}H_{17}ClN_2NaO^+$ [M+Na$^+$]: 359.0922; found: 359.0928.

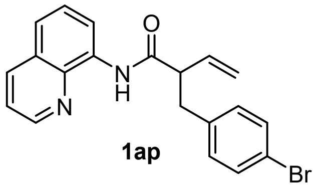

1ap

R$_f$ = 0.4, Petroleum Ether: Ethyl Acetate = 15: 1 (v/v)

Compound **1ap** was prepared following the general procedure in 10% yield (0.36 g) as a yellow solid. m.p. 125-127 °C.

^{1}**H NMR** (400 MHz, Chloroform-*d*) δ 9.91 (s, 1H), 8.87 – 8.67 (m, 2H), 8.13 (dd, *J* = 8.3, 1.7 Hz, 1H), 7.57 – 7.46 (m, 2H), 7.42 (dd, *J* = 8.3, 4.2 Hz, 1H), 7.39 – 7.30 (m,

2H), 7.18 – 7.06 (m, 2H), 6.11 – 5.93 (m, 1H), 5.37 – 5.20 (m, 2H), 3.46 (q, J = 7.6 Hz, 1H), 3.32 (dd, J = 13.8, 7.0 Hz, 1H), 2.93 (dd, J = 13.8, 7.4 Hz, 1H).

^{13}C NMR (101 MHz, Chloroform-*d*) δ 170.96, 148.33, 138.54, 138.26, 136.43, 136.08, 134.37, 131.52, 131.11, 128.02, 127.44, 121.81, 121.70, 120.30, 119.11, 116.61, 55.02, 37.59.

HRMS (ESI-TOF): m/z Calcd for $C_{20}H_{18}BrN_2O^+$ [M+H$^+$]: 381.0597; found: 381.0592.

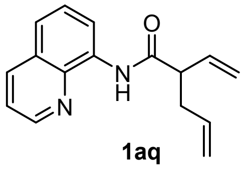

1aq

R$_f$ = 0.5, Petroleum Ether: Ethyl Acetate = 10: 1 (v/v)

Compound **1aq** was prepared following the general procedure in 22% yield (0.55 g) as a yellow oil.

^{1}H NMR (400 MHz, Chloroform-*d*) δ 10.00 (s, 1H), 8.78 (dq, J = 7.6, 3.3, 2.5 Hz, 2H), 8.11 (dd, J = 8.3, 1.7 Hz, 1H), 7.54 – 7.44 (m, 2H), 7.41 (dd, J = 8.3, 4.3 Hz, 1H), 6.03 (ddd, J = 17.2, 10.2, 8.6 Hz, 1H), 5.86 (ddt, J = 17.1, 10.2, 7.0 Hz, 1H), 5.42 – 5.27 (m, 2H), 5.20 – 5.01 (m, 2H), 3.29 (ddd, J = 8.5, 7.6, 6.7 Hz, 1H), 2.76 (dtt, J = 13.8, 6.8, 1.3 Hz, 1H), 2.55 – 2.42 (m, 1H).

^{13}C NMR (101 MHz, Chloroform-*d*) δ 171.32, 148.25, 138.51, 136.38, 136.33, 135.38, 134.41, 127.93, 127.36, 121.62, 121.60, 118.51, 117.12, 116.47, 53.02, 36.16.

HRMS (ESI-TOF): m/z Calcd for $C_{16}H_{17}N_2O^+$ [M+H$^+$]: 253.1335; found: 253.1334.

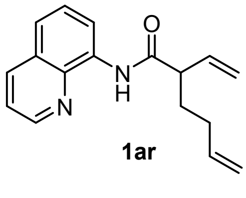

1ar

R$_f$ = 0.6, Petroleum Ether: Ethyl Acetate = 10: 1 (v/v)

Compound **1ar** was prepared following the general procedure in 19% yield (0.50 g) as a yellow oil.

^{1}H NMR (400 MHz, Chloroform-*d*) δ 9.98 (s, 1H), 8.82 – 8.72 (m, 2H), 8.09 (dd, J = 8.3, 1.7 Hz, 1H), 7.52 – 7.42 (m, 2H), 7.39 (dd, J = 8.3, 4.2 Hz, 1H), 6.01 (ddd, J =

17.1, 10.1, 8.7 Hz, 1H), 5.83 (ddt, *J* = 16.8, 10.2, 6.5 Hz, 1H), 5.41 – 5.24 (m, 2H), 5.10 – 4.96 (m, 2H), 3.21 (td, *J* = 8.1, 5.9 Hz, 1H), 2.22 – 2.06 (m, 3H), 1.83 – 1.70 (m, 1H).

^{13}C NMR (101 MHz, Chloroform-*d*) δ 171.88, 148.23, 138.50, 137.82, 136.80, 136.28, 134.47, 127.91, 127.33, 121.58, 121.55, 118.34, 116.41, 115.42, 52.59, 31.24, 30.89.

HRMS (ESI-TOF): m/z Calcd for $C_{17}H_{19}N_2O^+$ [M+H$^+$]: 267.1492; found: 267.1493.

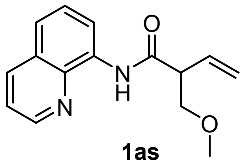

1as

R$_f$ = 0.4, Petroleum Ether: Ethyl Acetate = 5: 1 (v/v)

Compound **1as** was prepared following the general procedure in 8% yield (0.21 g) as a yellow oil.

^{1}H NMR (400 MHz, Chloroform-*d*) δ 10.35 (s, 1H), 8.84 – 8.73 (m, 2H), 8.13 (dd, *J* = 8.3, 1.7 Hz, 1H), 7.55 – 7.45 (m, 2H), 7.42 (dd, *J* = 8.3, 4.2 Hz, 1H), 6.09 (ddd, *J* = 17.2, 10.3, 8.3 Hz, 1H), 5.48 – 5.28 (m, 2H), 3.86 (dd, *J* = 9.2, 7.2 Hz, 1H), 3.72 (dd, *J* = 9.2, 5.2 Hz, 1H), 3.51 (dddd, *J* = 8.2, 7.2, 3.6, 2.6 Hz, 1H), 3.45 (s, 3H).

^{13}C NMR (101 MHz, Chloroform-*d*) δ 170.46, 148.30, 138.67, 136.40, 134.67, 133.75, 128.03, 127.45, 121.72, 121.63, 119.49, 116.79, 73.24, 59.29, 53.29.

HRMS (ESI-TOF): m/z Calcd for $C_{15}H_{16}N_2NaO_2^+$ [M+Na$^+$]: 279.1104; found: 279.1107.

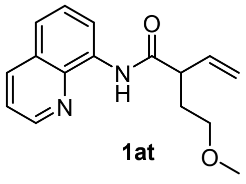

1at

R$_f$ = 0.5, Petroleum Ether: Ethyl Acetate = 5: 1 (v/v)

Compound **1at** was prepared following the general procedure in 13% yield (0.36 g) as a yellow oil.

^{1}H NMR (400 MHz, Chloroform-*d*) δ 10.02 (s, 1H), 8.86 – 8.71 (m, 2H), 8.12 (dd, *J* = 8.3, 1.7 Hz, 1H), 7.53 – 7.44 (m, 2H), 7.42 (dd, *J* = 8.3, 4.2 Hz, 1H), 6.02 (ddd, *J* =

17.1, 10.1, 8.7 Hz, 1H), 5.42 – 5.24 (m, 2H), 3.52 – 3.40 (m, 3H), 3.32 (s, 3H), 2.27 (ddt, *J* = 13.4, 7.2, 6.3 Hz, 1H), 1.91 (ddt, *J* = 13.7, 7.5, 6.1 Hz, 1H).

^{13}C NMR (101 MHz, Chloroform-*d*) δ 171.93, 148.27, 138.55, 136.64, 136.39, 134.56, 128.00, 127.42, 121.65, 121.62, 118.31, 116.54, 69.95, 58.73, 49.77, 31.84.

HRMS (ESI-TOF): m/z Calcd for $C_{16}H_{19}N_2O_2^+$ [M+H$^+$]: 271.1441; found: 271.1438.

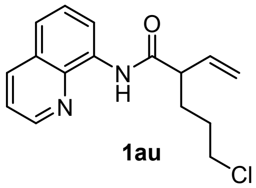

1au

R$_f$ = 0.5, Petroleum Ether: Ethyl Acetate = 15: 1 (v/v)

Compound **1au** was prepared following the general procedure in 10% yield (0.28 g) as a yellow oil.

^{1}H NMR (400 MHz, Chloroform-*d*) δ 10.00 (s, 1H), 8.84 – 8.70 (m, 2H), 8.14 (dd, *J* = 8.3, 1.7 Hz, 1H), 7.56 – 7.46 (m, 2H), 7.43 (dd, *J* = 8.3, 4.2 Hz, 1H), 6.01 (ddd, *J* = 17.1, 10.1, 8.6 Hz, 1H), 5.45 – 5.29 (m, 2H), 3.67 – 3.50 (m, 2H), 3.26 – 3.17 (m, 1H), 2.22 – 2.06 (m, 1H), 1.93 – 1.82 (m, 3H).

^{13}C NMR (101 MHz, Chloroform-*d*) δ 171.50, 148.35, 138.58, 136.51, 136.45, 134.44, 128.03, 127.44, 121.75, 121.70, 118.83, 116.57, 52.73, 44.82, 30.38, 29.16.

HRMS (ESI-TOF): m/z Calcd for $C_{16}H_{18}ClN_2O^+$ [M+H$^+$]: 289.1102; found: 289.1108.

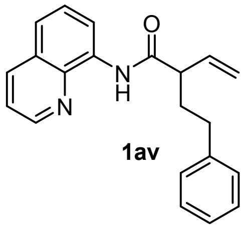

1av

R$_f$ = 0.3, Petroleum Ether: Ethyl Acetate = 10: 1 (v/v)

Compound **1av** was prepared following the general procedure in 9% yield (0.27 g) as a yellow oil.

^{1}H NMR (400 MHz, Chloroform-*d*) δ 10.00 (s, 1H), 8.89 – 8.75 (m, 2H), 8.15 (dd, *J* = 8.2, 1.7 Hz, 1H), 7.58 – 7.47 (m, 2H), 7.45 (dd, *J* = 8.3, 4.2 Hz, 1H), 7.33 – 7.19 (m, 5H), 6.07 (ddd, *J* = 17.2, 10.2, 8.7 Hz, 1H), 5.48 – 5.24 (m, 2H), 3.23 (q, *J* = 7.6 Hz,

1H), 2.83 – 2.69 (m, 2H), 2.40 (ddt, *J* = 13.6, 9.1, 6.7 Hz, 1H), 2.02 (dddd, *J* = 13.9, 9.1, 7.6, 6.5 Hz, 1H).

^{13}C NMR (101 MHz, Chloroform-*d*) δ 171.87, 148.31, 148.24, 141.63, 138.61, 136.88, 136.44, 134.54, 128.52, 128.04, 127.49, 126.04, 121.68, 121.67, 118.63, 116.59, 52.67, 33.40, 33.36.

HRMS (ESI-TOF): m/z Calcd for $C_{21}H_{21}N_2O^+$ [M+H$^+$]: 317.1648; found: 317.1654.

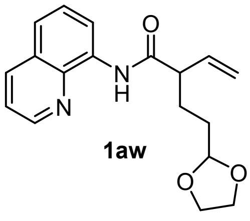

1aw

R$_f$ = 0.4, Petroleum Ether: Ethyl Acetate = 5: 1 (v/v)

Compound **1aw** was prepared following the general procedure in 13% yield (0.42 g) as a yellow oil.

^{1}H NMR (400 MHz, Chloroform-*d*) δ 10.00 (s, 1H), 8.83 – 8.72 (m, 2H), 8.12 (dd, *J* = 8.3, 1.7 Hz, 1H), 7.54 – 7.45 (m, 2H), 7.42 (dd, *J* = 8.3, 4.2 Hz, 1H), 6.01 (ddd, *J* = 17.1, 10.1, 8.6 Hz, 1H), 5.43 – 5.24 (m, 2H), 4.92 (t, *J* = 4.4 Hz, 1H), 4.01 – 3.89 (m, 2H), 3.88 – 3.77 (m, 2H), 3.26 (q, *J* = 7.8, 7.3 Hz, 1H), 2.21 – 2.07 (m, 1H), 1.87 – 1.71 (m, 3H).

^{13}C NMR (101 MHz, Chloroform-*d*) δ 171.91, 148.25, 138.56, 136.82, 136.43, 134.54, 128.01, 127.45, 121.63, 121.61, 118.45, 116.58, 104.41, 65.02, 64.97, 52.99, 31.43, 26.29.

HRMS (ESI-TOF): m/z Calcd for $C_{18}H_{20}N_2NaO_3^+$ [M+Na$^+$]: 335.1366; found: 335.1367.

4.3 General procedure for the substrate 1ax, 1ay'[20-21]

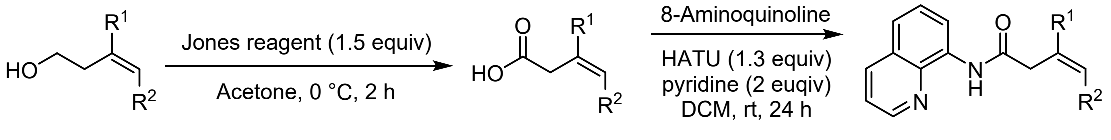

Scheme S4. Synthesis of substrate 1ax, 1ay'

Step 1: 3-methylbut-3-en-1-ol (10 mmol, 1 equiv) or (*Z*)-hex-3-en-1-ol (10 mmol, 1 equiv) in acetone (50 mL) was added 2.5 M Jones reagent (6 mL, 15 mmol, 1.5 equiv) dropwisely for 10 min at 0 °C. The reaction was stirred at 0 °C for 2 h. The reaction mixture was quenched with 2-propanol at 0 °C, diluted with water, and basified with 10% NaOH solution (25 mL) at 0 °C. The aqueous layer was washed with DCM (50 mL), acidified with conc. HCl to pH = 2 under ice cooling, and then extracted with DCM (50 mL, ×2). The combined organic layer was dried over Na_2SO_4 and concentrated under reduced pressure to afford the crude product, which was used in the next step without further purification.

Step 2: *β*-Substituted or *γ*-substituted vinyl acetic acid (12 mmol, 1.2 equiv) was charged into a 150 mL flask containing 30 mL DCM. 8-Aminoquinoline (1.44 g, 10 mmol, 1 equiv), pyridine (2.6 mL, 20 mmol, 2 equiv), and HATU (4.94 g, 13 mmol, 1.3 equiv) were added sequentially, and the reaction was stirred at room temperature for 24 h. The deep brown solution was diluted with DCM (100 mL), washed with sat. $NaHCO_3$ (100 mL, ×2) and brine (100 mL, ×2). The organic layer was evaporated under reduced pressure and the residue was purified by column chromatography on silica gel (200 - 300 mesh), eluting with 10% EtOAc/petroleum ether to give the desire product.

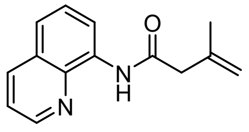

1ax

R_f = 0.3, Petroleum Ether: Ethyl Acetate = 10: 1 (v/v)

Compound **1ax** was prepared following the general procedure in 19% yield (0.42 g) as a yellow oil.

^{1}H NMR (400 MHz, Chloroform-*d*) δ 10.05 (s, 1H), 8.81 – 8.68 (m, 2H), 8.10 (dd, *J* = 8.3, 1.7 Hz, 1H), 7.53 – 7.43 (m, 2H), 7.40 (dd, *J* = 8.3, 4.2 Hz, 1H), 5.15 – 5.03 (m, 2H), 3.28 (d, *J* = 1.1 Hz, 2H), 1.91 (t, *J* = 1.2 Hz, 3H).

^{13}C NMR (101 MHz, Chloroform-*d*) δ 169.19, 148.31, 139.86, 138.56, 136.29, 134.45, 127.94, 127.35, 121.59, 116.41, 115.96, 47.91, 22.58.

HRMS (ESI-TOF): m/z Calcd for $C_{14}H_{14}N_2NaO^+$ [M+Na$^+$]: 249.0998; found: 249.0997.

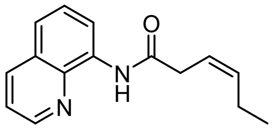

1ay'

R_f = 0.4, Petroleum Ether: Ethyl Acetate = 10: 1 (v/v)

Compound **1ay'** was prepared following the general procedure in 22% yield (0.52 g) as a yellow oil.

^{1}H NMR (400 MHz, Chloroform-*d*) δ 10.03 (s, 1H), 8.81 – 8.70 (m, 2H), 8.07 (dd, *J* = 8.3, 1.7 Hz, 1H), 7.51 – 7.40 (m, 2H), 7.37 (dd, *J* = 8.3, 4.2 Hz, 1H), 5.85 – 5.68 (m, 2H), 3.32 (dd, *J* = 7.3, 1.1 Hz, 2H), 2.18 (pd, *J* = 7.5, 1.4 Hz, 2H), 1.05 (t, *J* = 7.5 Hz, 3H).

^{13}C NMR (101 MHz, Chloroform-*d*) δ 169.63, 148.17, 138.48, 137.11, 136.22, 134.45, 127.88, 127.30, 121.53, 121.50, 120.68, 116.36, 36.77, 20.84, 13.95.

HRMS (ESI-TOF): m/z Calcd for $C_{15}H_{17}N_2O^+$ [M+H$^+$]: 241.1335; found: 241.1337.

4.4 General procedure for amide bond formation[21-24]

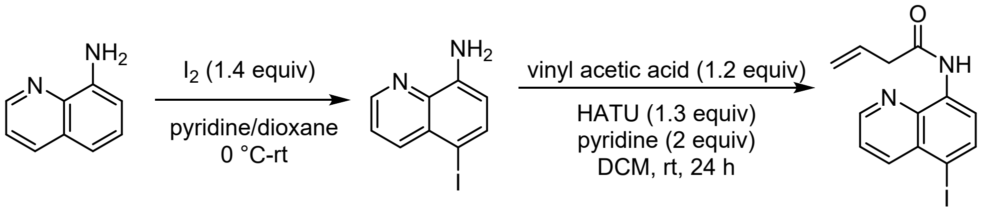

Scheme S5. Synthesis of substrate 1ba (1-IQ)

Step 1: The 8-aminoquinoline (20 g, 0.14 mol, 1 equiv) was dissolved in dioxane (150 mL) and pyridine (150 mL) and the solution was cooled to 0°C. Iodine (40 g, 0.16 mol, 1.14 equiv) was added in one portion. The solution progressively took a dark brown color. After 1 h, the ice bath was removed and a supplementary portion of iodine (10g, 0.04 mol) was added. The solution was further stirred for one hour at room temperature. A saturated solution of sodium thiosulfate was then added until the

brown color disappeared. The mixture was extracted with DCM (200mL, ×3) and washed with water, and dried by Na_2SO_4. The solvent was evaporated under reduced pressure and the residue was purified by column chromatography on silica gel (200 - 300 mesh), eluting with 10% EtOAc/petroleum ether to give the target compound as a bright yellow solid (28.2 g, 0.1 mol, 75% yield).

Step 2: Vinyl acetic acid (12 mmol, 1.2 equiv) was charged into a 150 mL flask containing 30 mL DCM. 5-Iodo-8-aminoquinoline (2.7 g, 10 mmol, 1 equiv), pyridine (2.6 mL, 20 mmol, 2 equiv), and HATU (4.94 g, 13 mmol, 1.3 equiv) were added sequentially, and the reaction was stirred at room temperature for 24 h. The deep brown solution was diluted with DCM (100 mL), washed with sat. $NaHCO_3$ (100 mL, ×2) and brine (100 mL, ×2). The organic layer was evaporated under reduced pressure and the residue was purified by column chromatography on silica gel (200 - 300 mesh), eluting with 10% EtOAc/petroleum ether to give the desire product.

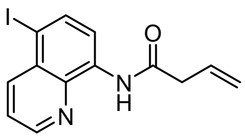

1ba

R_f = 0.4, Petroleum Ether: Ethyl Acetate = 10: 1 (v/v)

Compound **1ba** was prepared following the general procedure in 80% yield (2.70 g) as a yellow solid. m.p. 78-80 °C.

^{1}H NMR (400 MHz, Chloroform-*d*) δ 9.97 (s, 1H), 8.77 (dd, *J* = 4.2, 1.6 Hz, 1H), 8.54 (d, *J* = 8.3 Hz, 1H), 8.36 (dd, *J* = 8.5, 1.6 Hz, 1H), 8.07 (d, *J* = 8.3 Hz, 1H), 7.53 (dd, *J* = 8.5, 4.2 Hz, 1H), 6.13 (ddt, *J* = 16.6, 10.4, 7.1 Hz, 1H), 5.44 – 5.34 (m, 2H), 3.35 (dt, *J* = 7.1, 1.3 Hz, 2H).

^{13}C NMR (101 MHz, Chloroform-*d*) δ 169.46, 148.95, 140.85, 139.21, 138.38, 135.41, 130.87, 129.72, 123.28, 120.45, 117.96, 89.55, 43.32.

HRMS (ESI-TOF): m/z Calcd for $C_{13}H_{12}IN_2O^+$ [M+H$^+$]: 338.9989; found: 338.9989.

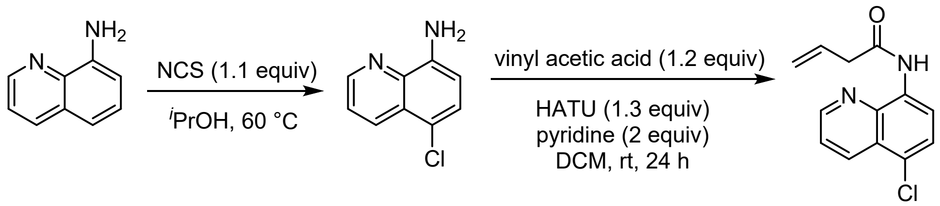

Scheme S6. Synthesis of substrate 1c (1-ClQ)

Step 1: *N*-Chlorosuccinimide (2.5 g, 19.1 mmol, 1.1 equiv) was added in one portion to a solution of 8-aminoquinoline (2.5g, 17.3 mmol, 1 equiv) in ^{i}PrOH (25 mL) at 60 °C. The reaction mixture was heated at reflux for 3 h. Saturated aqueous $NaHCO_3$ solution was added and the mixture was extracted with DCM (30 mL, ×3), washed with brine and dried by Na_2SO_4. The solvent was removed under reduced pressure to give a brown solid. Purified by column chromatography on silica gel (200 - 300 mesh), eluting with 20% EtOAc/petroleum ether to give the target compound as a bright yellow solid. (1.2g, 40% yield)

Step 2: Vinyl acetic acid (12 mmol, 1.2 equiv) was charged into a 150 mL flask containing 30 mL DCM. 5-Chloro-8-aminoquinoline (1.8 g, 10 mmol, 1 equiv), pyridine (2.6 mL, 20 mmol, 2 equiv), and HATU (4.94 g, 13 mmol, 1.3 equiv) were added sequentially, and the reaction was stirred at room temperature for 24 h. The deep brown solution was diluted with DCM (100 mL), washed with sat. $NaHCO_3$ (100 mL, ×2) and brine (100 mL, ×2). The organic layer was evaporated under reduced pressure and the residue was purified by column chromatography on silica gel (200 - 300 mesh), eluting with 10% EtOAc/petroleum ether to give the desire product.

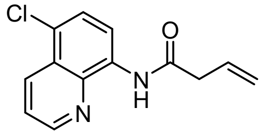

1c

R_f = 0.5, Petroleum Ether: Ethyl Acetate = 10: 1 (v/v)

Compound **1c** was prepared following the general procedure in 62% yield (1.53 g) as a white solid. m.p. 80-82 °C.

^{1}**H NMR** (400 MHz, Chloroform-*d*) δ 9.82 (s, 1H), 8.75 (dd, *J* = 4.3, 1.7 Hz, 1H), 8.63 (d, *J* = 8.4 Hz, 1H), 8.43 (dd, *J* = 8.5, 1.7 Hz, 1H), 7.52 – 7.42 (m, 2H), 6.11 (ddt, *J* = 16.5, 10.4, 7.1 Hz, 1H), 5.43 – 5.29 (m, 2H), 3.32 (dt, *J* = 7.1, 1.3 Hz, 2H).

^{13}**C NMR** (101 MHz, Chloroform-*d*) δ 169.18, 148.64, 138.87, 133.58, 133.20, 130.82, 127.10, 125.76, 124.26, 122.26, 120.26, 116.24, 43.13.

HRMS (ESI-TOF): m/z Calcd for $C_{13}H_{12}ClN_2O^+$ [M+H$^+$]: 247.0633; found: 247.0631.

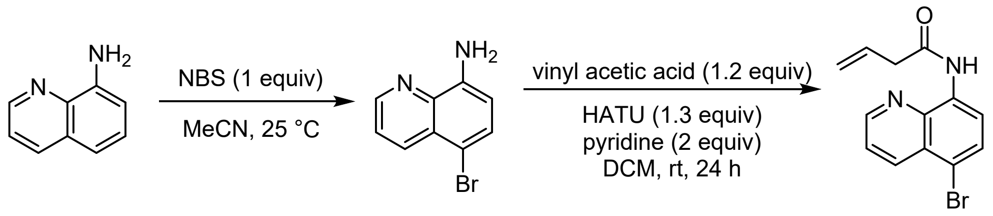

Scheme S7. Synthesis of substrate 1d (1-BrQ)

Step 1: *N*-Bromosuccinimide (6.1 g, 34.7 mmol, 1 equiv) was added to a solution of 8-aminoquinoline (5.0 g, 34.7 mmol, 1 equiv) in MeCN (80 mL). The reaction was stirred for 30 min at 25 °C. The reaction was quenched with saturated aqueous $NaHCO_3$ solution (100 mL) and dried by Na_2SO_4. The solvent was evaporated under reduced pressure and the residue was purified by column chromatography on silica gel (200 - 300 mesh), eluting with 20% EtOAc/petroleum ether to give the target compound as a yellow solid (3.8 g, 17 mmol, 50% yield).

Step 2: Vinyl acetic acid (12 mmol, 1.2 equiv) was charged into a 150 mL flask containing 30 mL DCM. 5-Bromo-8-aminoquinoline (2.2 g, 10 mmol, 1 equiv), pyridine (2.6 mL, 20 mmol, 2 equiv), and HATU (4.94 g, 13 mmol, 1.3 equiv) were added sequentially, and the reaction was stirred at room temperature for 24 h. The deep brown solution was diluted with DCM (100 mL), washed with sat. $NaHCO_3$ (100 mL, ×2) and brine (100 mL, ×2). The organic layer was evaporated under reduced pressure and the residue was purified by column chromatography on silica gel (200 - 300 mesh), eluting with 10% EtOAc/petroleum ether to give the desire product.

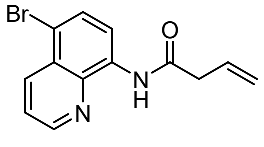

1d

R_f = 0.5, Petroleum Ether: Ethyl Acetate = 10: 1 (v/v)

Compound **1d** was prepared following the general procedure in 82% yield (2.38 g) as

a yellow solid. m.p. 74-76 °C.

^{1}H NMR (400 MHz, Chloroform-*d*) δ 9.94 (s, 1H), 8.82 (dd, *J* = 4.3, 1.6 Hz, 1H), 8.67 (d, *J* = 8.4 Hz, 1H), 8.52 (dd, *J* = 8.5, 1.6 Hz, 1H), 7.79 (d, *J* = 8.4 Hz, 1H), 7.56 (dd, *J* = 8.5, 4.2 Hz, 1H), 6.13 (ddt, *J* = 17.2, 10.3, 7.1 Hz, 1H), 5.48 – 5.30 (m, 2H), 3.35 (dt, *J* = 7.1, 1.3 Hz, 2H).

^{13}C NMR (101 MHz, Chloroform-*d*) δ 169.43, 148.82, 139.26, 136.16, 134.42, 131.06, 130.89, 127.33, 122.78, 120.43, 117.16, 114.45, 43.29.

HRMS (ESI-TOF): m/z Calcd for $C_{13}H_{12}BrN_2O^+$ [M+H$^+$]: 291.0128; found: 291.0132.

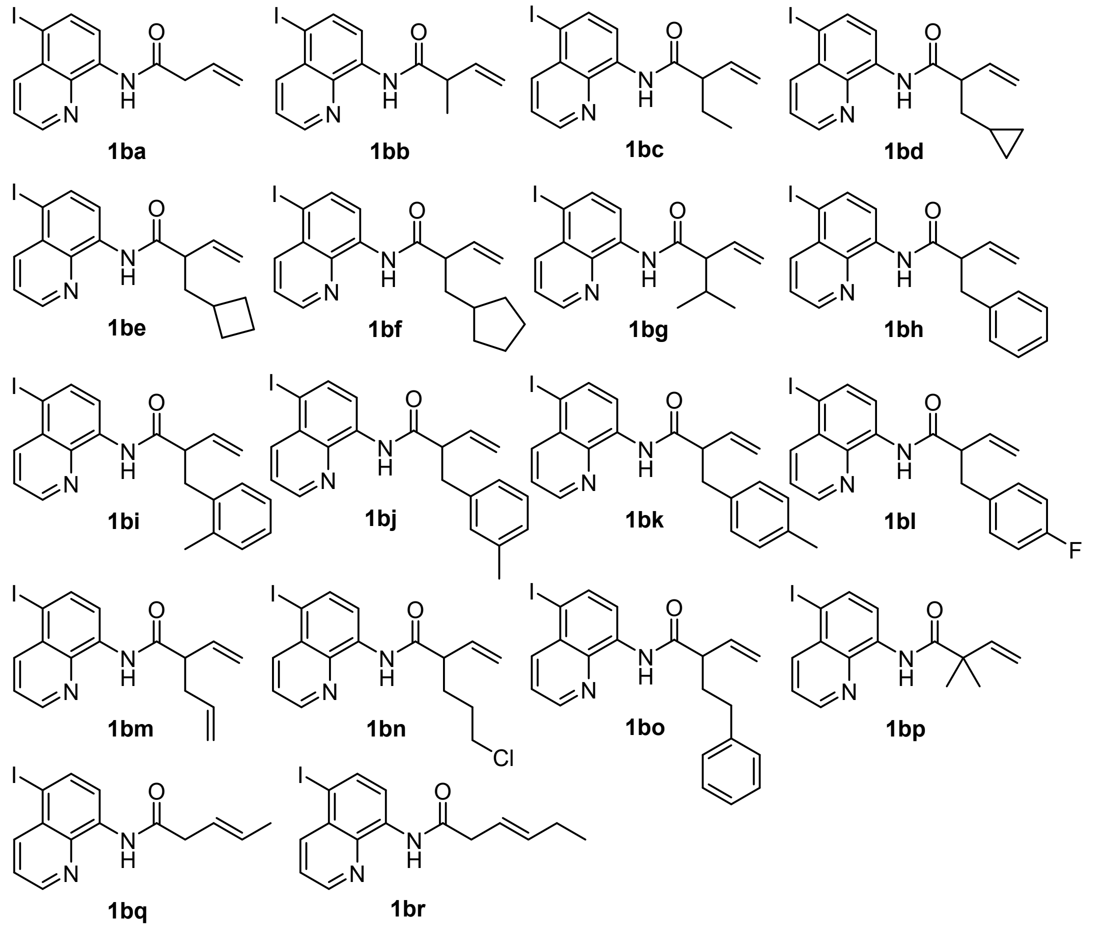

Scheme S8. Alkene Substrates (IQ-Directed, Yields are unoptimized)

4.5 General procedure for the substrate 1bp, 1bq, 1br[20-21]

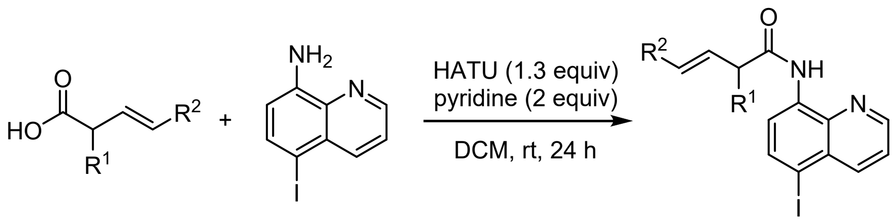

Scheme S9. Synthesis of substrate 1bp, 1bq, 1br

α-Substituted vinyl acetic acid or γ-substituted vinyl acetic acid (12 mmol, 1.2 equiv, from commercial sources) was charged into a 150 mL flask containing 30 mL DCM. 5-Iodo-8-aminoquinoline (2.7 g, 10 mmol, 1 equiv), pyridine (2.6 mL, 20 mmol, 2 equiv), and HATU (4.94 g, 13 mmol, 1.3 equiv) were added sequentially, and the reaction was stirred at room temperature for 24 h. The deep brown solution was diluted with DCM (100 mL), washed with sat. $NaHCO_3$ (100 mL, ×2) and brine (100 mL, ×2). The organic layer was evaporated under reduced pressure and the residue was purified by column chromatography on silica gel (200 - 300 mesh), eluting with 10% EtOAc/petroleum ether to give the desire product.

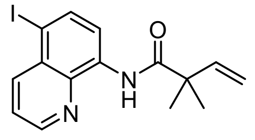

1bp

R_f = 0.3, Petroleum Ether: Ethyl Acetate = 10: 1 (v/v)

Compound **1bp** was prepared following the general procedure in 65% yield (2.38 g) as a white solid. m.p. 68-70 °C.

^{1}H NMR (400 MHz, Chloroform-*d*) δ 10.23 (s, 1H), 8.75 (dd, *J* = 4.2, 1.6 Hz, 1H), 8.54 (d, *J* = 8.3 Hz, 1H), 8.33 (dd, *J* = 8.5, 1.6 Hz, 1H), 8.05 (d, *J* = 8.3 Hz, 1H), 7.50 (dd, *J* = 8.5, 4.2 Hz, 1H), 6.22 (dd, *J* = 17.5, 10.6 Hz, 1H), 5.50 – 5.33 (m, 2H), 1.47 (s, 6H).

^{13}C NMR (101 MHz, Chloroform-*d*) δ 175.05, 148.98, 142.81, 140.71, 139.57, 138.34, 135.70, 129.67, 123.17, 117.72, 115.31, 89.26, 47.01, 24.93.

HRMS (ESI-TOF): m/z Calcd for $C_{15}H_{16}IN_2O^+$ [M+H$^+$]: 367.0302; found: 367.0298.

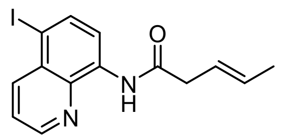

1bq

R$_f$ = 0.5, Petroleum Ether: Ethyl Acetate = 10: 1 (v/v)

Compound **1bq** was prepared following the general procedure in 89% yield (3.13 g) as a yellow solid. m.p. 78-80 °C.

^{1}H NMR (400 MHz, Chloroform-*d*) δ 10.00 (s, 1H), 8.74 (dd, *J* = 4.3, 1.6 Hz, 1H), 8.52 (d, *J* = 8.2 Hz, 1H), 8.33 (dd, *J* = 8.5, 1.6 Hz, 1H), 8.04 (d, *J* = 8.3 Hz, 1H), 7.50 (dd, *J* = 8.5, 4.2 Hz, 1H), 5.90 – 5.63 (m, 2H), 3.25 (dd, *J* = 6.6, 1.5 Hz, 2H), 1.82 (dd, *J* = 5.9, 1.3 Hz, 3H).

^{13}C NMR (101 MHz, Chloroform-*d*) δ 170.23, 148.86, 140.77, 139.15, 138.34, 135.43, 131.69, 129.64, 123.36, 123.19, 117.82, 89.36, 42.19, 18.23.

HRMS (ESI-TOF): m/z Calcd for $C_{14}H_{13}IN_2NaO^+$ [M+Na$^+$]: 374.9965; found: 374.9967.

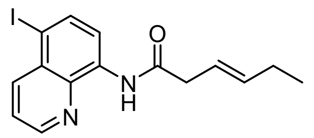

1br

R$_f$ = 0.4, Petroleum Ether: Ethyl Acetate = 10: 1 (v/v)

Compound **1br** was prepared following the general procedure in 82% yield (3.00 g) as a yellow solid. m.p. 83-85 °C.

^{1}H NMR (400 MHz, Chloroform-*d*) δ 10.07 (s, 1H), 8.72 (dd, *J* = 4.2, 1.6 Hz, 1H), 8.53 (d, *J* = 8.2 Hz, 1H), 8.33 (dd, *J* = 8.5, 1.6 Hz, 1H), 8.04 (d, *J* = 8.3 Hz, 1H), 7.50 (dd, *J* = 8.5, 4.2 Hz, 1H), 5.87 (dtt, *J* = 15.0, 6.2, 1.1 Hz, 1H), 5.72 (dtt, *J* = 15.5, 7.2, 1.5 Hz, 1H), 3.26 (dd, *J* = 7.0, 1.2 Hz, 2H), 2.19 (qdd, *J* = 7.5, 6.3, 1.3 Hz, 2H), 1.12 (t, *J* = 7.5 Hz, 3H).

^{13}C NMR (101 MHz, Chloroform-*d*) δ 170.27, 148.78, 140.75, 139.19, 139.08, 138.35, 135.45, 129.65, 123.19, 121.27, 117.78, 89.35, 42.19, 25.91, 13.70.

HRMS (ESI-TOF): m/z Calcd for $C_{15}H_{16}IN_2O^+$ [M+H$^+$]: 367.0302; found: 367.0308.

4.6 General procedure for the substrate 1bb-1bo[20-21]

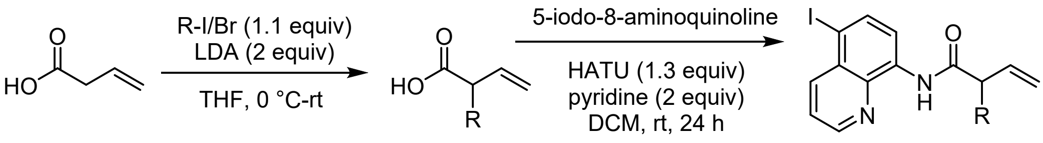

Scheme S10. Synthesis of substrate 1bb-1bo

Step1: A solution of commercial LDA 2 M (11.8 mL, 23.5 mmol, 2 equiv) in THF was cooled to ice-water temperature and a solution of 3-butenoic acid (1 mL, 11.77 mmol, 1 equiv) in 10 mL of THF was added slowly over a period of 15 min. The resulting mixture was stirred at the 0°C for 45 min to obtain a deep yellow solution. A total of R-I/Br (12.9 mmol, 1.1 equiv) of the alkylating agent was added, whereupon the reaction mixture immediately turned colorless. After 30 min at the same temperature and 3 h at room temperature, the pH of the solution was adjusted to 2.5 with 10% HCl. The organic phase was separated. The aqueous layer was saturated with solid NaCl and the mixture was extracted with DCM. The combined organic layers were dried over anhydrous Na_2SO_4, filtered and concentrated *in vacuo* to obtain the α-substituted 3-butenoic acid.

Step 2: α-Substituted vinyl acetic acid (12 mmol, 1.2 equiv) was charged into a 150 mL flask containing 30 mL DCM. 5-Iodo-8-aminoquinoline (2.7 g, 10 mmol, 1 equiv), pyridine (2.6 mL, 20 mmol, 2 equiv), and HATU (4.94 g, 13 mmol, 1.3 equiv) were added sequentially, and the reaction was stirred at room temperature for 24 h. The deep brown solution was diluted with DCM (100 mL), washed with sat. $NaHCO_3$ (100 mL, ×2) and brine (100 mL, ×2). The organic layer was evaporated under reduced pressure and the residue was purified by column chromatography on silica gel (200 - 300 mesh), eluting with 5%-15% EtOAc/petroleum ether to give the desire product.

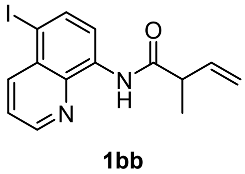

1bb

R$_f$ = 0.6, Petroleum Ether: Ethyl Acetate = 10: 1 (v/v)

Compound **1bb** was prepared following the general procedure in 52% yield (1.62 g) as a yellow solid. m.p. 60-62 °C.

^{1}H NMR (400 MHz, Chloroform-*d*) δ 9.98 (s, 1H), 8.71 (dd, *J* = 4.2, 1.6 Hz, 1H), 8.51 (d, *J* = 8.3 Hz, 1H), 8.27 (dd, *J* = 8.5, 1.6 Hz, 1H), 8.00 (d, *J* = 8.3 Hz, 1H), 7.45 (dd, *J* = 8.5, 4.2 Hz, 1H), 6.08 (ddd, *J* = 17.3, 10.2, 7.9 Hz, 1H), 5.43 – 5.26 (m, 2H), 3.41 – 3.27 (m, 1H), 1.43 (d, *J* = 7.0 Hz, 3H).

^{13}C NMR (101 MHz, Chloroform-*d*) δ 172.55, 148.80, 140.60, 139.09, 138.20, 137.83, 135.39, 129.50, 123.12, 117.72, 117.59, 89.35, 47.05, 16.94.

HRMS (ESI-TOF): m/z Calcd for $C_{14}H_{13}IN_2NaO^+$ [M+Na$^+$]: 374.9965; found: 374.9968.

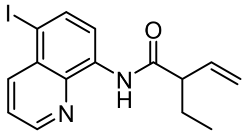

1bc

R$_f$ = 0.5, Petroleum Ether: Ethyl Acetate = 10: 1 (v/v)

Compound **1bc** was prepared following the general procedure in 42% yield (1.54 g) as a yellow solid. m.p. 74-75 °C.

^{1}H NMR (400 MHz, Chloroform-*d*) δ 9.98 (s, 1H), 8.77 (dd, *J* = 4.3, 1.6 Hz, 1H), 8.57 (d, *J* = 8.3 Hz, 1H), 8.36 (dd, *J* = 8.5, 1.6 Hz, 1H), 8.07 (d, *J* = 8.3 Hz, 1H), 7.53 (dd, *J* = 8.5, 4.2 Hz, 1H), 6.00 (ddd, *J* = 17.1, 10.2, 8.6 Hz, 1H), 5.44 – 5.23 (m, 2H), 3.09 (q, *J* = 7.5 Hz, 1H), 2.03 (tt, *J* = 13.8, 7.3 Hz, 1H), 1.72 (dp, *J* = 13.6, 7.5 Hz, 1H), 1.01 (t, *J* = 7.4 Hz, 3H).

^{13}C NMR (101 MHz, Chloroform-*d*) δ 172.34, 148.91, 140.86, 139.26, 138.40, 136.73, 135.54, 129.72, 123.25, 118.55, 117.95, 89.36, 55.23, 25.13, 11.92.

HRMS (ESI-TOF): m/z Calcd for $C_{15}H_{15}IN_2NaO^+$ [M+Na$^+$]: 389.0121; found: 389.0118.

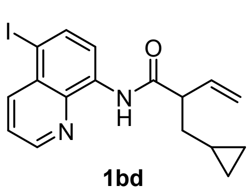

1bd

R_f = 0.4, Petroleum Ether: Ethyl Acetate = 10: 1 (v/v)

Compound **1bd** was prepared following the general procedure in 27% yield (1.06 g) as a yellow solid. m.p. 65-67 °C.

^{1}H NMR (400 MHz, Chloroform-*d*) δ 10.01 (s, 1H), 8.77 (dd, *J* = 4.2, 1.6 Hz, 1H), 8.56 (d, *J* = 8.3 Hz, 1H), 8.36 (dd, *J* = 8.5, 1.6 Hz, 1H), 8.06 (d, *J* = 8.3 Hz, 1H), 7.52 (dd, *J* = 8.5, 4.2 Hz, 1H), 6.07 (ddd, *J* = 17.1, 10.2, 8.6 Hz, 1H), 5.42 – 5.26 (m, 2H), 3.31 (q, *J* = 7.5 Hz, 1H), 1.82 (dt, *J* = 14.4, 7.3 Hz, 1H), 1.68 (dt, *J* = 13.9, 6.9 Hz, 1H), 0.85 – 0.74 (m, 1H), 0.54 – 0.37 (m, 2H), 0.20 – 0.06 (m, 2H).

^{13}C NMR (101 MHz, Chloroform-*d*) δ 172.34, 148.91, 140.84, 139.26, 138.41, 136.99, 135.56, 129.71, 123.24, 118.13, 117.93, 89.35, 54.07, 37.33, 9.14, 4.85, 4.74.

HRMS (ESI-TOF): m/z Calcd for $C_{17}H_{18}IN_2O^+$ [M+H$^+$]: 393.0458; found: 393.0462.

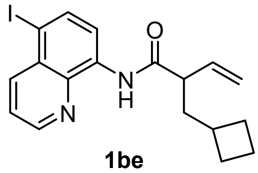

1be

R_f = 0.5, Petroleum Ether: Ethyl Acetate = 10: 1 (v/v)

Compound **1be** was prepared following the general procedure in 17% yield (0.69 g) as a yellow solid. m.p. 58-60 °C.

^{1}H NMR (400 MHz, Chloroform-*d*) δ 9.94 (s, 1H), 8.76 (dd, *J* = 4.2, 1.6 Hz, 1H), 8.55 (d, *J* = 8.3 Hz, 1H), 8.34 (dd, *J* = 8.5, 1.6 Hz, 1H), 8.05 (d, *J* = 8.3 Hz, 1H), 7.51 (dd, *J* = 8.5, 4.2 Hz, 1H), 5.99 (ddd, *J* = 17.1, 10.1, 8.6 Hz, 1H), 5.37 – 5.23 (m, 2H), 3.17 – 3.05 (m, 1H), 2.40 (hept, *J* = 7.9 Hz, 1H), 2.15 – 1.97 (m, 3H), 1.89 – 1.74 (m, 3H), 1.71 – 1.60 (m, 2H).

^{13}C NMR (101 MHz, Chloroform-*d*) δ 172.33, 148.90, 140.80, 139.22, 138.37, 136.99, 135.51, 129.68, 123.22, 118.05, 117.90, 89.35, 51.83, 39.21, 34.02, 28.63, 28.35, 18.60.

HRMS (ESI-TOF): m/z Calcd for $C_{18}H_{19}IN_2NaO^+$ [M+Na$^+$]: 429.0434; found: 429.0436.

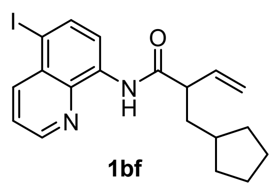

1bf

R_f = 0.6, Petroleum Ether: Ethyl Acetate = 10: 1 (v/v)

Compound **1bf** was prepared following the general procedure in 21% yield (0.88 g) as a yellow solid. m.p. 65-67 °C.

^{1}H NMR (400 MHz, Chloroform-*d*) δ 9.98 (s, 1H), 8.78 (dd, *J* = 4.3, 1.6 Hz, 1H), 8.56 (d, *J* = 8.3 Hz, 1H), 8.36 (dd, *J* = 8.5, 1.6 Hz, 1H), 8.06 (d, *J* = 8.3 Hz, 1H), 7.53 (dd, *J* = 8.5, 4.2 Hz, 1H), 6.00 (ddd, *J* = 17.1, 10.1, 8.7 Hz, 1H), 5.40 – 5.24 (m, 2H), 3.22 (q, *J* = 7.8 Hz, 1H), 2.00 (dt, *J* = 13.2, 7.1 Hz, 1H), 1.94 – 1.76 (m, 3H), 1.72 (ddd, *J* = 13.2, 8.0, 6.1 Hz, 1H), 1.63 – 1.58 (m, 2H), 1.55 – 1.45 (m, 2H), 1.24 – 1.07 (m, 2H).

^{13}C NMR (101 MHz, Chloroform-*d*) δ 172.53, 148.93, 140.85, 139.27, 138.40, 137.24, 135.56, 129.72, 123.25, 118.09, 117.95, 89.34, 53.00, 38.39, 37.79, 33.05, 32.48, 25.32, 25.26.

HRMS (ESI-TOF): m/z Calcd for $C_{19}H_{22}IN_2O^+$ [M+H$^+$]: 421.0771; found: 421.0768.

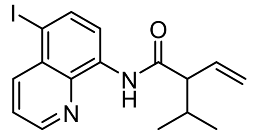

1bg

R_f = 0.4, Petroleum Ether: Ethyl Acetate = 10: 1 (v/v)

Compound **1bg** was prepared following the general procedure in 12% yield (0.34 g) as a yellow oil.

^{1}H NMR (400 MHz, Chloroform-*d*) δ 9.91 (s, 1H), 8.76 (dd, *J* = 4.2, 1.6 Hz, 1H), 8.57 (d, *J* = 8.3 Hz, 1H), 8.34 (dd, *J* = 8.5, 1.6 Hz, 1H), 8.05 (d, *J* = 8.3 Hz, 1H), 7.51 (dd, *J* = 8.5, 4.2 Hz, 1H), 6.08 – 5.96 (m, 1H), 5.35 – 5.26 (m, 2H), 2.86 (dd, *J* = 9.4, 8.0 Hz, 1H), 2.27 (dp, *J* = 8.0, 6.6 Hz, 1H), 1.03 (d, *J* = 6.7 Hz, 3H), 0.99 (d, *J* = 6.7 Hz, 3H).

^{13}C NMR (101 MHz, Chloroform-*d*) δ 172.28, 148.87, 140.80, 139.18, 138.35, 135.58, 135.46, 129.66, 123.22, 119.14, 117.93, 89.36, 61.46, 30.38, 21.14, 19.79.

HRMS (ESI-TOF): m/z Calcd for $C_{16}H_{18}IN_2O^+$ [M+H$^+$]: 381.0458; found: 381.0465.

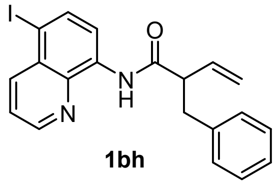

1bh

R$_f$ = 0.5, Petroleum Ether: Ethyl Acetate = 8: 1 (v/v)

Compound **1bh** was prepared following the general procedure in 61% yield (2.61 g) as a white solid. m.p. 88-90 °C.

^{1}H NMR (400 MHz, Chloroform-*d*) δ 9.93 (s, 1H), 8.73 (dd, *J* = 4.3, 1.6 Hz, 1H), 8.57 (d, *J* = 8.3 Hz, 1H), 8.35 (dd, *J* = 8.5, 1.6 Hz, 1H), 8.07 (d, *J* = 8.3 Hz, 1H), 7.51 (dd, *J* = 8.5, 4.2 Hz, 1H), 7.27 (d, *J* = 1.6 Hz, 4H), 7.18 (ddd, *J* = 8.7, 5.0, 3.7 Hz, 1H), 6.12 – 5.99 (m, 1H), 5.36 – 5.23 (m, 2H), 3.50 (dt, *J* = 8.5, 7.1 Hz, 1H), 3.38 (dd, *J* = 13.7, 6.9 Hz, 1H), 2.99 (dd, *J* = 13.7, 7.5 Hz, 1H).

^{13}C NMR (101 MHz, Chloroform-*d*) δ 171.44, 148.87, 140.77, 139.18, 139.13, 138.33, 136.23, 135.39, 129.66, 129.32, 128.50, 126.47, 123.23, 119.01, 117.93, 89.50, 55.27, 38.20.

HRMS (ESI-TOF): m/z Calcd for $C_{20}H_{17}IN_2NaO^+$ [M+Na$^+$]: 451.0278; found: 451.0277.

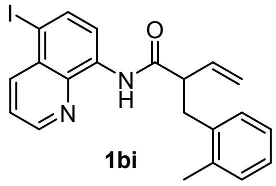

1bi

R$_f$ = 0.4, Petroleum Ether: Ethyl Acetate = 8: 1 (v/v)

Compound **1bi** was prepared following the general procedure in 54% yield (2.39 g) as a yellow solid. m.p. 99-101 °C.

^{1}H NMR (400 MHz, Chloroform-*d*) δ 9.92 (s, 1H), 8.72 (dd, *J* = 4.2, 1.5 Hz, 1H), 8.56 (d, *J* = 8.2 Hz, 1H), 8.36 (dd, *J* = 8.5, 1.5 Hz, 1H), 8.07 (d, *J* = 8.3 Hz, 1H), 7.51 (dd, *J* = 8.5, 4.2 Hz, 1H), 7.21 – 7.15 (m, 1H), 7.15 – 7.11 (m, 1H), 7.10 – 7.02 (m, 2H), 6.14 – 6.02 (m, 1H), 5.32 – 5.20 (m, 2H), 3.48 (q, *J* = 7.6 Hz, 1H), 3.39 (dd, *J* = 13.9, 6.7 Hz, 1H), 2.96 (dd, *J* = 13.9, 7.5 Hz, 1H), 2.38 (s, 3H).

^{13}C NMR (101 MHz, Chloroform-*d*) δ 171.63, 148.76, 140.97, 139.03, 138.41, 137.37, 136.41, 136.31, 135.34, 130.45, 129.94, 129.69, 126.58, 125.98, 123.21, 118.87, 118.09, 89.51, 54.08, 35.32, 19.80.

HRMS (ESI-TOF): m/z Calcd for $C_{21}H_{19}IN_2NaO^+$ [M+Na$^+$]: 465.0434; found: 465.0438.

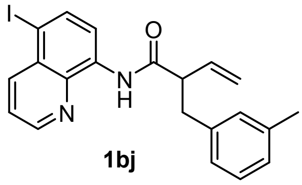

1bj

R$_f$ = 0.4, Petroleum Ether: Ethyl Acetate = 8: 1 (v/v)

Compound **1bj** was prepared following the general procedure in 32% yield (1.41 g) as a yellow solid. m.p. 102-104 °C.

^{1}H NMR (400 MHz, Chloroform-*d*) δ 9.90 (s, 1H), 8.71 (dd, *J* = 4.3, 1.6 Hz, 1H), 8.55 (dd, *J* = 8.3, 1.2 Hz, 1H), 8.33 (dd, *J* = 8.5, 1.6 Hz, 1H), 8.05 (dd, *J* = 8.3, 1.2 Hz, 1H), 7.49 (dd, *J* = 8.5, 4.2 Hz, 1H), 7.14 (t, *J* = 7.4 Hz, 1H), 7.11 – 7.02 (m, 2H), 6.97 (d, *J* = 7.5 Hz, 1H), 6.05 (dddd, *J* = 17.0, 10.0, 8.5, 1.2 Hz, 1H), 5.35 – 5.23 (m, 2H), 3.50 (q, *J* = 7.6 Hz, 1H), 3.33 (dd, *J* = 13.7, 6.9 Hz, 1H), 2.95 (dd, *J* = 13.7, 7.5 Hz, 1H), 2.28 (s, 3H).

^{13}C NMR (101 MHz, Chloroform-*d*) δ 171.54, 148.75, 140.82, 139.06, 139.01, 138.34, 138.00, 136.31, 135.34, 130.08, 129.63, 128.36, 127.20, 126.29, 123.18, 118.85, 118.00, 89.46, 55.21, 38.17, 21.49.

HRMS (ESI-TOF): m/z Calcd for $C_{21}H_{19}IN_2NaO^+$ [M+Na$^+$]: 465.0434; found: 465.0434.

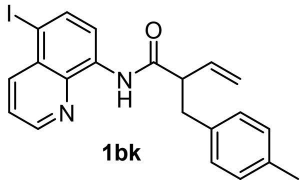

1bk

R$_f$ = 0.5, Petroleum Ether: Ethyl Acetate = 10: 1 (v/v)

Compound **1bk** was prepared following the general procedure in 58% yield (2.56 g) as a yellow solid. m.p. 106-108 °C.

^{1}H NMR (400 MHz, Chloroform-*d*) δ 9.92 (s, 1H), 8.72 (dd, *J* = 4.2, 1.5 Hz, 1H), 8.55 (d, *J* = 8.3 Hz, 1H), 8.34 (dd, *J* = 8.5, 1.6 Hz, 1H), 8.06 (d, *J* = 8.3 Hz, 1H), 7.50 (dd, *J* = 8.5, 4.2 Hz, 1H), 7.16 – 7.11 (m, 2H), 7.05 (d, *J* = 8.0 Hz, 2H), 6.04 (ddd, *J* = 17.0, 10.2, 8.5 Hz, 1H), 5.33 – 5.23 (m, 2H), 3.48 (q, *J* = 7.6 Hz, 1H), 3.32 (dd, *J* = 13.8, 6.9 Hz, 1H), 2.95 (dd, *J* = 13.8, 7.6 Hz, 1H), 2.26 (s, 3H).

^{13}C NMR (101 MHz, Chloroform-*d*) δ 171.57, 148.76, 140.88, 139.08, 138.38, 136.32, 135.98, 135.91, 135.37, 129.66, 129.19, 129.17, 123.18, 118.89, 118.05, 89.46, 55.31, 37.79, 21.15.

HRMS (ESI-TOF): m/z Calcd for $C_{21}H_{19}IN_2NaO^+$ [M+Na$^+$]: 465.0434; found: 465.0434.

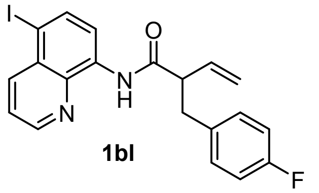

1bl

R$_f$ = 0.4, Petroleum Ether: Ethyl Acetate = 15: 1 (v/v)

Compound **1bl** was prepared following the general procedure in 49% yield (2.19 g) as a white solid. m.p. 114-116 °C.

^{1}H NMR (400 MHz, Chloroform-*d*) δ 9.89 (s, 1H), 8.72 (dd, *J* = 4.2, 1.5 Hz, 1H), 8.53 (d, *J* = 8.3 Hz, 1H), 8.34 (dd, *J* = 8.5, 1.5 Hz, 1H), 8.06 (d, *J* = 8.3 Hz, 1H), 7.51 (dd, *J* = 8.5, 4.2 Hz, 1H), 7.23 – 7.15 (m, 2H), 6.98 – 6.87 (m, 2H), 6.09 – 5.95 (m, 1H), 5.32 – 5.24 (m, 2H), 3.43 (q, *J* = 7.6 Hz, 1H), 3.31 (dd, *J* = 13.8, 7.1 Hz, 1H), 2.95 (dd, *J* = 13.8, 7.3 Hz, 1H).

^{13}C NMR (101 MHz, Chloroform-*d*) δ 171.23, 162.90, 160.47, 148.91, 140.81, 139.17, 138.33, 136.04, 135.30, 134.79, 130.80, 130.72, 129.70, 123.27, 119.17, 117.95, 115.40, 115.19, 89.61, 55.41, 37.40.

^{19}F NMR (376 MHz, Chloroform-*d*) δ -116.87.

HRMS (ESI-TOF): m/z Calcd for $C_{20}H_{17}FIN_2O^+$ [M+H$^+$]: 447.0364; found: 447.0357.

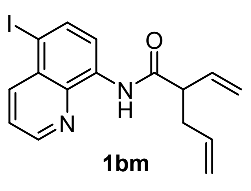

1bm

R_f = 0.5, Petroleum Ether: Ethyl Acetate = 15: 1 (v/v)

Compound **1bm** was prepared following the general procedure in 32% yield (1.21 g) as a yellow solid. m.p. 60-62 °C.

^{1}H NMR (400 MHz, Chloroform-*d*) δ 10.01 (s, 1H), 8.77 (dd, *J* = 4.2, 1.6 Hz, 1H), 8.56 (d, *J* = 8.3 Hz, 1H), 8.37 (dd, *J* = 8.5, 1.6 Hz, 1H), 8.07 (d, *J* = 8.3 Hz, 1H), 7.53 (dd, *J* = 8.5, 4.2 Hz, 1H), 6.02 (ddd, *J* = 17.2, 10.2, 8.6 Hz, 1H), 5.84 (ddt, *J* = 17.1, 10.2, 7.0 Hz, 1H), 5.42 – 5.31 (m, 2H), 5.19 – 5.02 (m, 2H), 3.28 (q, *J* = 7.6 Hz, 1H), 2.80 – 2.69 (m, 1H), 2.54 – 2.42 (m, 1H).

^{13}C NMR (101 MHz, Chloroform-*d*) δ 171.45, 148.86, 140.80, 139.14, 138.32, 136.25, 135.36, 135.33, 129.65, 123.20, 118.82, 117.96, 117.29, 89.48, 53.09, 36.14.

HRMS (ESI-TOF): m/z Calcd for $C_{16}H_{16}IN_2O^+$ [M+H$^+$]: 379.0302; found: 379.0309.

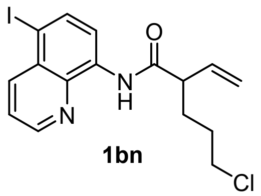

1bn

R_f = 0.4, Petroleum Ether: Ethyl Acetate = 20: 1 (v/v)

Compound **1bn** was prepared following the general procedure in 16% yield (0.66 g) as a yellow solid. m.p. 78-80 °C.

^{1}H NMR (400 MHz, Chloroform-*d*) δ 10.00 (s, 1H), 8.76 (dd, *J* = 4.2, 1.6 Hz, 1H), 8.53 (d, *J* = 8.3 Hz, 1H), 8.35 (dd, *J* = 8.5, 1.6 Hz, 1H), 8.06 (d, *J* = 8.3 Hz, 1H), 7.52 (dd, *J* = 8.5, 4.2 Hz, 1H), 6.00 (ddd, *J* = 17.0, 10.1, 8.6 Hz, 1H), 5.46 – 5.30 (m, 2H), 3.68 – 3.48 (m, 2H), 3.21 (t, *J* = 7.7 Hz, 1H), 2.12 (dtd, *J* = 14.8, 9.7, 6.6 Hz, 1H), 1.98 – 1.77 (m, 3H).

^{13}C NMR (101 MHz, Chloroform-*d*) δ 171.55, 148.96, 140.80, 139.20, 138.31, 136.32, 135.35, 129.68, 123.28, 119.15, 117.91, 89.58, 52.76, 44.81, 30.36, 29.05.

HRMS (ESI-TOF): m/z Calcd for $C_{16}H_{16}ClIN_2NaO^+$ [M+Na$^+$]: 436.9888; found: 436.9884.

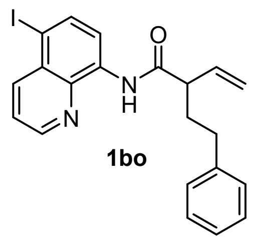

1bo

R_f = 0.3, Petroleum Ether: Ethyl Acetate = 10: 1 (v/v)

Compound **1bo** was prepared following the general procedure in 21% yield (0.93 g) as a yellow oil.

^{1}H NMR (400 MHz, Chloroform-*d*) δ 9.98 (s, 1H), 8.77 (dd, *J* = 4.2, 1.6 Hz, 1H), 8.57 (d, *J* = 8.3 Hz, 1H), 8.36 (dd, *J* = 8.5, 1.5 Hz, 1H), 8.07 (d, *J* = 8.3 Hz, 1H), 7.53 (dd, *J* = 8.5, 4.2 Hz, 1H), 7.32 – 7.26 (m, 2H), 7.25 – 7.16 (m, 3H), 6.04 (ddd, *J* = 17.2, 10.2, 8.7 Hz, 1H), 5.43 – 5.31 (m, 2H), 3.24 – 3.14 (m, 1H), 2.82 – 2.65 (m, 2H), 2.37 (ddt, *J* = 13.6, 9.2, 6.7 Hz, 1H), 2.00 (dddd, *J* = 13.8, 9.0, 7.7, 6.4 Hz, 1H).

^{13}C NMR (101 MHz, Chloroform-*d*) δ 171.94, 148.93, 141.55, 140.84, 139.26, 138.38, 136.69, 135.48, 129.72, 128.70, 128.57, 126.12, 123.27, 118.93, 117.96, 89.46, 52.67, 33.34, 33.28.

HRMS (ESI-TOF): m/z Calcd for $C_{21}H_{19}IN_2NaO^+$ [M+Na$^+$]: 465.0434; found: 465.0435.

5. General Procedures for the Heck-type Coupling Reactions

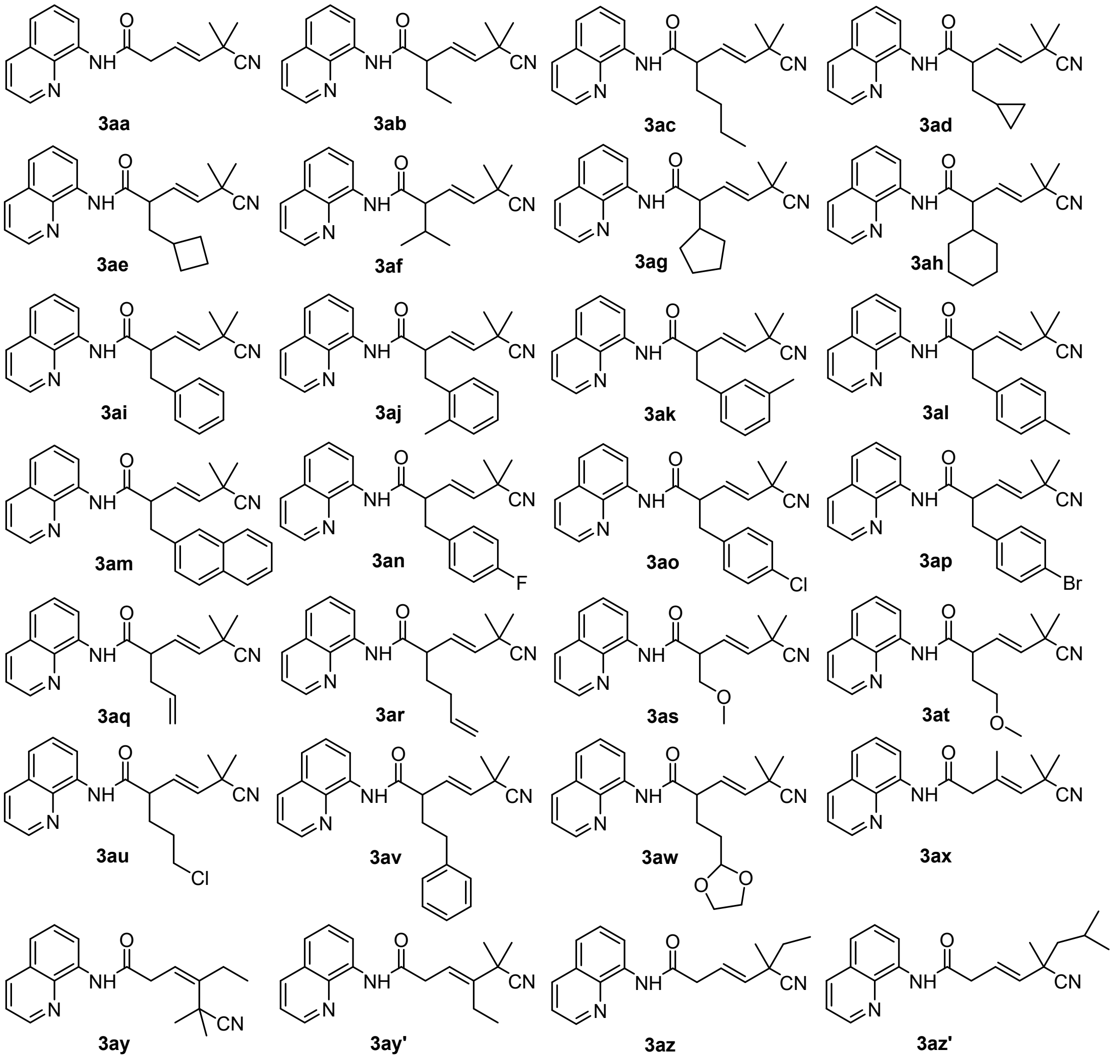

Scheme S11. Products of the Heck-type Coupling Reactions

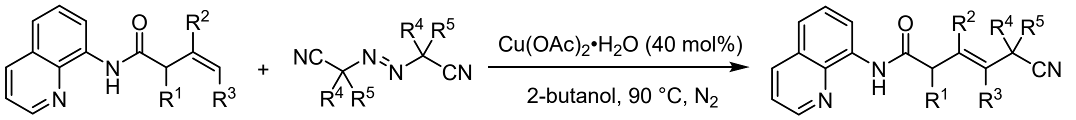

Scheme S12. Synthesis of products of the Heck-type Coupling Reactions

The alkenes substrate (0.2 mmol, 1 equiv), azo compounds (0.6 mmol, 3 equiv), $Cu(OAc)_2 \cdot H_2O$ (16mg, 0.08 mmol, 0.4 equiv), 2-butanol (1 mL) were added into a 15 mL sealed glass vial under N_2 atmosphere. The reaction mixture was heated at 90 °C for 12 h. After being cooled to room temperature, the mixture was concentrated *in vacuo* and purified by silica gel (300 - 400 mesh) column chromatography to give the

desired products.

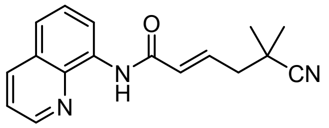

3'-AQ

R$_f$ = 0.4, Petroleum Ether: Ethyl Acetate = 2: 1 (v/v)

Compound **3'-AQ** was prepared following the general procedure as a white solid. m.p. 136-138 °C.

^{1}H NMR (400 MHz, Chloroform-*d*) δ 9.94 (s, 1H), 8.89 – 8.77 (m, 2H), 8.19 (dd, *J* = 8.3, 1.7 Hz, 1H), 7.60 – 7.51 (m, 2H), 7.48 (dd, *J* = 8.3, 4.2 Hz, 1H), 7.06 (dt, *J* = 15.3, 7.7 Hz, 1H), 6.36 (dt, *J* = 15.1, 1.4 Hz, 1H), 2.54 (dd, *J* = 7.7, 1.3 Hz, 2H), 1.43 (s, 6H).

^{13}C NMR (101 MHz, Chloroform-*d*) δ 163.20, 148.26, 138.49, 136.75, 134.44, 129.43, 128.12, 127.65, 124.37, 122.02, 121.82, 117.20, 43.25, 32.53, 26.50.

HRMS (ESI-TOF): m/z Calcd for $C_{17}H_{17}N_3NaO^+$ [M+Na$^+$]: 302.1264; found: 302.1267.

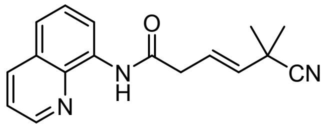

3aa

R$_f$ = 0.4, Petroleum Ether: Ethyl Acetate = 4: 1 (v/v)

Compound **3aa** was prepared following the general procedure in 75% yield (42 mg) as a white solid. m.p. 140-142 °C.

^{1}H NMR (400 MHz, Chloroform-*d*) δ 10.00 (s, 1H), 8.78 – 8.64 (m, 2H), 8.12 (dd, *J* = 8.3, 1.7 Hz, 1H), 7.53 – 7.45 (m, 2H), 7.42 (dd, *J* = 8.3, 4.2 Hz, 1H), 6.15 (dt, *J* = 15.5, 7.2 Hz, 1H), 5.73 (dt, *J* = 15.5, 1.3 Hz, 1H), 3.32 (dd, *J* = 7.2, 1.3 Hz, 2H), 1.54 (s, 6H).

^{13}C NMR (101 MHz, Chloroform-*d*) δ 168.44, 148.20, 138.39, 136.85, 136.42, 134.15, 127.93, 127.35, 123.39, 123.33, 121.80, 121.71, 116.39, 41.19, 34.74, 27.37.

HRMS (ESI-TOF): m/z Calcd for $C_{17}H_{18}N_3O^+$ [M+H$^+$]: 280.1444; found: 280.1447.

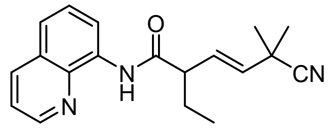

3ab

R_f = 0.4, Petroleum Ether: Ethyl Acetate = 5: 1 (v/v)

Compound **3ab** was prepared following the general procedure in 78% yield (48 mg) as a white solid. m.p. 139-141 °C.

^{1}H NMR (400 MHz, Chloroform-*d*) δ 9.92 (s, 1H), 8.69 – 8.62 (m, 2H), 8.03 (dt, *J* = 8.3, 1.5 Hz, 1H), 7.44 – 7.35 (m, 2H), 7.33 (ddd, *J* = 8.3, 4.2, 1.2 Hz, 1H), 5.94 (dd, *J* = 15.5, 8.8 Hz, 1H), 5.61 (dd, *J* = 15.5, 0.9 Hz, 1H), 3.05 – 2.95 (m, 1H), 2.03 – 1.89 (m, 1H), 1.63 (dt, *J* = 13.6, 7.5 Hz, 1H), 1.41 (s, 6H), 0.90 (t, *J* = 7.4 Hz, 3H).

^{13}C NMR (101 MHz, Chloroform-*d*) δ 171.34, 148.23, 138.52, 136.42, 135.21, 134.32, 129.03, 127.97, 127.40, 123.43, 121.71, 116.41, 53.09, 34.72, 27.56, 27.38, 25.11, 11.85.

HRMS (ESI-TOF): m/z Calcd for $C_{19}H_{21}N_3NaO^+$ [M+Na$^+$]: 330.1577; found: 330.1557.

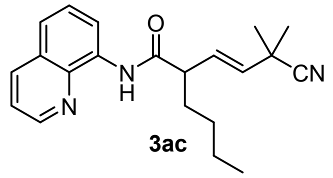

3ac

R_f = 0.5, Petroleum Ether: Ethyl Acetate = 5: 1 (v/v)

Compound **3ac** was prepared following the general procedure in 68% yield (46 mg) as a white solid. m.p. 148-150 °C.

^{1}H NMR (400 MHz, Chloroform-*d*) δ 10.02 (s, 1H), 8.81 – 8.71 (m, 2H), 8.15 (dd, *J* = 8.3, 1.7 Hz, 1H), 7.55 – 7.47 (m, 2H), 7.44 (dd, *J* = 8.3, 4.2 Hz, 1H), 6.05 (dd, *J* = 15.5, 8.8 Hz, 1H), 5.71 (dd, *J* = 15.5, 0.9 Hz, 1H), 3.23 – 3.12 (m, 1H), 2.09 – 1.97 (m, 1H), 1.74 – 1.64 (m, 1H), 1.54 (s, 3H), 1.51 (s, 3H), 1.36 (qd, *J* = 9.0, 7.7, 3.8 Hz, 4H), 0.94 – 0.86 (m, 3H).

^{13}C NMR (101 MHz, Chloroform-*d*) δ 171.50, 148.25, 138.56, 136.49, 134.95, 134.38, 129.40, 128.03, 127.47, 123.47, 121.73, 116.49, 51.61, 34.75, 31.71, 29.52, 27.60, 27.41, 22.64, 14.04.

HRMS (ESI-TOF): m/z Calcd for $C_{21}H_{25}N_3NaO^+$ [M+Na$^+$]: 358.1890; found: 358.1893.

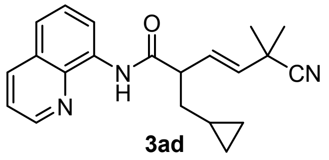

3ad

R$_f$ = 0.4, Petroleum Ether: Ethyl Acetate = 5: 1 (v/v)

Compound **3ad** was prepared following the general procedure in 72% yield (48 mg) as a white solid. m.p. 106-108 °C.

^{1}H NMR (400 MHz, Chloroform-*d*) δ 9.95 (s, 1H), 8.66 (ddd, *J* = 9.0, 5.7, 1.9 Hz, 2H), 8.03 (dd, *J* = 8.3, 1.7 Hz, 1H), 7.44 – 7.36 (m, 2H), 7.33 (dd, *J* = 8.3, 4.2 Hz, 1H), 6.02 (dd, *J* = 15.5, 8.8 Hz, 1H), 5.64 (dd, *J* = 15.5, 0.8 Hz, 1H), 3.28 – 3.15 (m, 1H), 1.77 (dt, *J* = 13.9, 7.0 Hz, 1H), 1.58 (dt, *J* = 14.1, 7.2 Hz, 1H), 1.40 (s, 3H), 0.66 (ddt, *J* = 10.3, 7.6, 3.8 Hz, 1H), 0.43 – 0.29 (m, 2H), 0.10 – 0.04 (m, 2H).

^{13}C NMR (101 MHz, Chloroform-*d*) δ 171.34, 148.23, 138.52, 136.41, 134.72, 134.34, 129.35, 127.97, 127.40, 123.44, 121.70, 116.39, 52.06, 37.21, 34.67, 27.49, 27.32, 9.04, 4.98, 4.59.

HRMS (ESI-TOF): m/z Calcd for $C_{21}H_{23}N_3NaO^+$ [M+Na$^+$]: 356.1733; found: 356.1734.

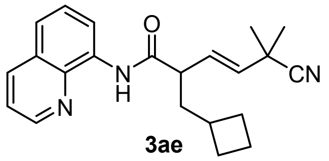

3ae

R$_f$ = 0.5, Petroleum Ether: Ethyl Acetate = 5: 1 (v/v)

Compound **3ae** was prepared following the general procedure in 65% yield (45 mg) as a white solid. m.p. 104-106 °C.

^{1}H NMR (400 MHz, Chloroform-*d*) δ 9.99 (s, 1H), 8.80 – 8.71 (m, 2H), 8.14 (dd, *J* = 8.3, 1.7 Hz, 1H), 7.54 – 7.46 (m, 2H), 7.43 (dd, *J* = 8.3, 4.2 Hz, 1H), 6.03 (dd, *J* = 15.5, 8.8 Hz, 1H), 5.68 (dd, *J* = 15.5, 0.8 Hz, 1H), 3.13 (td, *J* = 8.3, 6.2 Hz, 1H), 2.38 (hept, *J* = 7.9 Hz, 1H), 2.18 – 2.07 (m, 2H), 2.06 – 2.00 (m, 1H), 1.86 – 1.76 (m, 3H), 1.68 (qd, *J* = 8.8, 1.8 Hz, 2H), 1.53 (s, 3H), 1.50 (s, 3H).

^{13}C NMR (101 MHz, Chloroform-*d*) δ 171.36, 148.22, 138.51, 136.44, 134.59, 134.34, 129.41, 127.99, 127.42, 123.41, 121.70, 121.69, 116.43, 49.98, 39.14, 34.66, 34.08, 28.73, 28.26, 27.48, 27.31, 18.55.

HRMS (ESI-TOF): m/z Calcd for $C_{22}H_{25}N_3NaO^+$ [M+Na$^+$]: 370.1890; found: 370.1889.

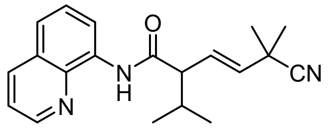

3af

R$_f$ = 0.5, Petroleum Ether: Ethyl Acetate = 5: 1 (v/v)

Compound **3af** was prepared following the general procedure in 69% yield (44 mg) as a white solid. m.p. 101-103 °C.

^{1}H NMR (400 MHz, Chloroform-*d*) δ 9.95 (s, 1H), 8.82 – 8.73 (m, 2H), 8.13 (dd, *J* = 8.2, 1.7 Hz, 1H), 7.54 – 7.46 (m, 2H), 7.43 (dd, *J* = 8.3, 4.2 Hz, 1H), 6.08 (dd, *J* = 15.5, 9.5 Hz, 1H), 5.67 (d, *J* = 15.5 Hz, 1H), 2.89 (dd, *J* = 9.5, 7.6 Hz, 1H), 2.39 – 2.27 (m, *J* = 6.8 Hz, 1H), 1.51 (s, 3H), 1.49 (s, 3H), 1.03 (d, *J* = 6.7 Hz, 3H), 0.98 (d, *J* = 6.8 Hz, 3H).

^{13}C NMR (101 MHz, Chloroform-*d*) δ 171.31, 148.24, 138.47, 136.44, 135.67, 134.29, 127.98, 127.76, 127.40, 123.46, 121.71, 116.48, 59.06, 34.69, 30.45, 27.60, 27.35, 21.08, 19.58.

HRMS (ESI-TOF): m/z Calcd for $C_{20}H_{24}N_3O^+$ [M+H$^+$]: 322.1914; found: 322.1919.

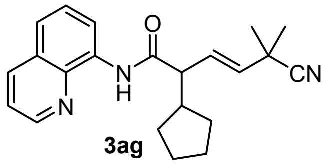

3ag

R$_f$ = 0.4, Petroleum Ether: Ethyl Acetate = 5: 1 (v/v)

Compound **3ag** was prepared following the general procedure in 61% yield (42 mg) as a yellow oil.

^{1}H NMR (400 MHz, Chloroform-*d*) δ 9.93 (s, 1H), 8.79 (dd, *J* = 4.3, 1.7 Hz, 1H), 8.76 (dd, *J* = 7.0, 2.0 Hz, 1H), 8.15 (dd, *J* = 8.3, 1.7 Hz, 1H), 7.55 – 7.47 (m, 2H), 7.44 (dd, *J* = 8.3, 4.2 Hz, 1H), 6.08 (dd, *J* = 15.5, 9.1 Hz, 1H), 5.67 (dd, *J* = 15.5, 0.7 Hz, 1H), 2.98 (t, *J* = 9.1 Hz, 1H), 2.42 (qt, *J* = 9.1, 7.4 Hz, 1H), 1.92 – 1.85 (m, 1H),

1.84 – 1.76 (m, 1H), 1.64 (tdd, J = 7.5, 3.5, 1.9 Hz, 2H), 1.61 – 1.53 (m, 2H), 1.49 (s, 3H), 1.48 (s, 3H), 1.38 – 1.29 (m, 1H), 1.25 (dddd, J = 13.7, 7.7, 3.4, 1.5 Hz, 1H).

^{13}C NMR (101 MHz, Chloroform-*d*) δ 171.54, 148.26, 138.50, 136.50, 134.46, 134.39, 128.87, 128.03, 127.46, 123.51, 121.72, 121.70, 116.57, 57.51, 42.26, 34.64, 31.09, 30.43, 27.61, 27.37, 25.22, 25.04.

HRMS (ESI-TOF): m/z Calcd for $C_{22}H_{25}N_3NaO^+$ [M+Na$^+$]: 370.1890; found: 370.1896.

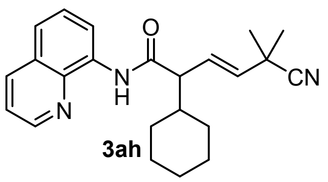

3ah

R$_f$ = 0.5, Petroleum Ether: Ethyl Acetate = 5: 1 (v/v)

Compound **3ah** was prepared following the general procedure in 54% yield (39 mg) as a yellow solid. m.p. 113-115 °C.

^{1}H NMR (400 MHz, Chloroform-*d*) δ 9.93 (s, 1H), 8.83 – 8.75 (m, 2H), 8.15 (dd, J = 8.3, 1.7 Hz, 1H), 7.55 – 7.47 (m, 2H), 7.45 (dd, J = 8.3, 4.2 Hz, 1H), 6.07 (dd, J = 15.5, 9.5 Hz, 1H), 5.64 (dd, J = 15.5, 0.6 Hz, 1H), 2.91 (dd, J = 9.5, 8.0 Hz, 1H), 1.97 (tdt, J = 11.5, 7.9, 3.3 Hz, 1H), 1.85 – 1.75 (m, 3H), 1.71 (dq, J = 16.0, 2.9 Hz, 2H), 1.50 (s, 3H), 1.49 (s, 3H), 1.33 – 1.23 (m, 2H), 1.20 – 1.05 (m, 2H), 0.98 (qd, J = 12.3, 3.2 Hz, 1H).

^{13}C NMR (101 MHz, Chloroform-*d*) δ 171.40, 148.27, 138.51, 136.52, 135.25, 134.33, 128.13, 128.05, 127.49, 123.57, 121.74, 116.57, 58.60, 40.01, 34.67, 31.62, 30.22, 27.68, 27.37, 26.35, 26.27.

HRMS (ESI-TOF): m/z Calcd for $C_{23}H_{28}N_3O^+$ [M+H$^+$]: 362.2227; found: 362.2229.

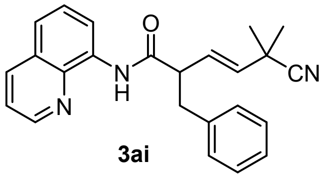

3ai

R$_f$ = 0.4, Petroleum Ether: Ethyl Acetate = 5: 1 (v/v)

Compound **3ai** was prepared following the general procedure in 68% yield (50 mg) as a white solid. m.p. 114-116 °C.

^{1}H NMR (400 MHz, Chloroform-*d*) δ 9.98 (s, 1H), 8.74 (dd, *J* = 7.2, 1.8 Hz, 1H), 8.72 (dd, *J* = 4.2, 1.7 Hz, 1H), 8.10 (dd, *J* = 8.3, 1.7 Hz, 1H), 7.54 – 7.44 (m, 2H), 7.39 (dd, *J* = 8.3, 4.2 Hz, 1H), 7.26 – 7.18 (m, 4H), 7.17 – 7.12 (m, 1H), 6.08 – 5.99 (m, 1H), 5.47 (dd, *J* = 15.5, 0.7 Hz, 1H), 3.47 – 3.36 (m, 2H), 2.95 (tt, *J* = 10.5, 3.5 Hz, 1H), 1.42 (s, 3H), 1.34 (s, 3H).

^{13}C NMR (101 MHz, Chloroform-*d*) δ 170.54, 148.22, 138.81, 138.47, 136.41, 135.77, 134.22, 129.39, 128.43, 128.31, 127.96, 127.39, 126.48, 123.25, 121.82, 121.71, 116.48, 53.43, 38.11, 34.65, 27.35, 27.25.

HRMS (ESI-TOF): m/z Calcd for $C_{24}H_{23}N_3NaO^+$ [M+Na$^+$]: 392.1733; found: 392.1731.

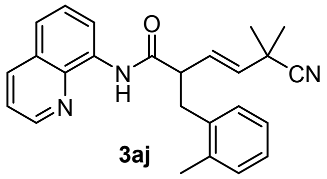

3aj

R$_f$ = 0.5, Petroleum Ether: Ethyl Acetate = 5: 1 (v/v)

Compound **3aj** was prepared following the general procedure in 61% yield (47 mg) as a white solid. m.p. 119-121 °C.

^{1}H NMR (400 MHz, Chloroform-*d*) δ 10.03 (s, 1H), 8.78 (dd, *J* = 7.3, 1.8 Hz, 1H), 8.75 (dd, *J* = 4.2, 1.7 Hz, 1H), 8.13 (dd, *J* = 8.3, 1.7 Hz, 1H), 7.56 – 7.48 (m, 2H), 7.43 (dd, *J* = 8.3, 4.2 Hz, 1H), 7.17 – 7.12 (m, 2H), 7.12 – 7.04 (m, 2H), 6.15 – 6.05 (m, 1H), 5.45 (dd, *J* = 15.4, 0.7 Hz, 1H), 3.51 – 3.40 (m, 2H), 3.04 – 2.91 (m, 1H), 2.39 (s, 3H), 1.45 (s, 3H), 1.36 (s, 3H).

^{13}C NMR (101 MHz, Chloroform-*d*) δ 170.68, 148.22, 138.46, 137.06, 136.40, 136.24, 135.59, 134.22, 130.40, 130.13, 128.24, 127.95, 127.38, 126.60, 125.88, 123.21, 121.80, 121.71, 116.44, 52.30, 35.14, 34.64, 27.35, 27.18, 19.70.

HRMS (ESI-TOF): m/z Calcd for $C_{25}H_{25}N_3NaO^+$ [M+Na$^+$]: 406.1890; found: 406.1894.

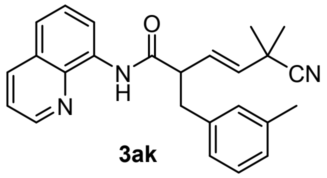

3ak

R_f = 0.6, Petroleum Ether: Ethyl Acetate = 5: 1 (v/v)

Compound **3ak** was prepared following the general procedure in 54% yield (41 mg) as a white solid. m.p. 134-136 °C.

^{1}H NMR (400 MHz, Chloroform-*d*) δ 10.00 (s, 1H), 8.78 (dd, *J* = 7.3, 1.7 Hz, 1H), 8.75 (dd, *J* = 4.3, 1.7 Hz, 1H), 8.13 (dd, *J* = 8.3, 1.7 Hz, 1H), 7.56 – 7.47 (m, 2H), 7.42 (dd, *J* = 8.3, 4.2 Hz, 1H), 7.16 (t, *J* = 7.5 Hz, 1H), 7.06 – 6.95 (m, 3H), 6.07 (dd, *J* = 15.5, 8.7 Hz, 1H), 5.51 (dd, *J* = 15.5, 0.7 Hz, 1H), 3.50 – 3.34 (m, 2H), 2.94 (dd, *J* = 13.2, 8.2 Hz, 1H), 2.29 (s, 3H), 1.46 (s, 3H), 1.39 (s, 3H).

^{13}C NMR (101 MHz, Chloroform-*d*) δ 170.63, 148.20, 138.70, 138.49, 137.91, 136.37, 135.62, 134.25, 130.20, 128.45, 128.31, 127.95, 127.38, 127.19, 126.37, 123.28, 121.77, 121.69, 116.46, 53.44, 38.11, 34.64, 27.35, 27.29, 21.40.

HRMS (ESI-TOF): m/z Calcd for $C_{25}H_{25}N_3NaO^+$ [M+Na$^+$]: 406.1890; found: 406.1892.

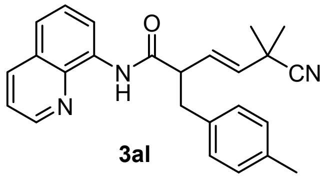

3al

R_f = 0.6, Petroleum Ether: Ethyl Acetate = 5: 1 (v/v)

Compound **3al** was prepared following the general procedure in 60% yield (46 mg) as a white solid. m.p. 126-128 °C.

^{1}H NMR (400 MHz, Chloroform-*d*) δ 9.99 (s, 1H), 8.80 – 8.73 (m, 2H), 8.14 (dd, *J* = 8.2, 1.7 Hz, 1H), 7.57 – 7.48 (m, 2H), 7.43 (dd, *J* = 8.3, 4.2 Hz, 1H), 7.11 (d, *J* = 8.1 Hz, 2H), 7.07 (d, *J* = 8.0 Hz, 2H), 6.06 (dd, *J* = 15.5, 8.7 Hz, 1H), 5.53 (dd, *J* = 15.6, 0.7 Hz, 1H), 3.49 – 3.31 (m, 2H), 2.94 (dd, *J* = 13.4, 8.1 Hz, 1H), 2.28 (s, 3H), 1.46 (s, 3H), 1.39 (s, 3H).

^{13}C NMR (101 MHz, Chloroform-*d*) δ 170.70, 148.24, 138.54, 136.45, 135.98, 135.70, 135.59, 134.31, 129.29, 129.15, 128.53, 128.01, 127.46, 123.35, 121.81, 121.73, 116.54, 53.56, 37.80, 34.70, 27.40, 27.33, 21.10.

HRMS (ESI-TOF): m/z Calcd for $C_{25}H_{26}N_3O^+$ [M+H$^+$]: 384.2070; found: 384.2074.

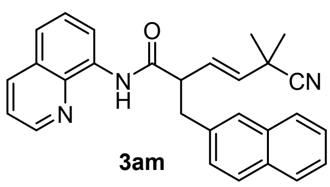

R_f = 0.5, Petroleum Ether: Ethyl Acetate = 5: 1 (v/v)

Compound **3am** was prepared following the general procedure in 53% yield (44 mg) as a yellow solid. m.p. 151-153 °C.

^{1}H NMR (400 MHz, Chloroform-*d*) δ 10.02 (s, 1H), 8.79 (dd, *J* = 7.5, 1.6 Hz, 1H), 8.68 (dd, *J* = 4.3, 1.7 Hz, 1H), 8.11 (dd, *J* = 8.3, 1.7 Hz, 1H), 7.76 (ddd, *J* = 8.1, 5.0, 2.5 Hz, 3H), 7.70 – 7.66 (m, 1H), 7.57 – 7.47 (m, 2H), 7.45 – 7.35 (m, 4H), 6.19 – 6.09 (m, 1H), 5.55 – 5.46 (m, 1H), 3.65 – 3.49 (m, 2H), 3.14 (tt, *J* = 12.2, 4.1 Hz, 1H), 1.42 (s, 3H), 1.32 (s, 3H).

^{13}C NMR (101 MHz, Chloroform-*d*) δ 170.53, 148.19, 138.46, 136.41, 136.39, 135.82, 134.21, 133.55, 132.28, 128.41, 128.06, 127.96, 127.78, 127.65, 127.58, 127.40, 126.06, 125.49, 123.27, 121.85, 121.70, 116.55, 53.45, 38.33, 34.68, 27.38, 27.24.

HRMS (ESI-TOF): m/z Calcd for $C_{28}H_{25}N_3NaO^+$ [M+Na$^+$]: 442.1890; found: 442.1891.

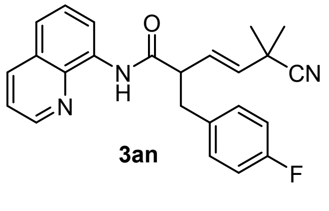

R_f = 0.4, Petroleum Ether: Ethyl Acetate = 5: 1 (v/v)

Compound **3an** was prepared following the general procedure in 52% yield (40 mg) as a yellow solid. m.p. 129-131 °C.

^{1}H NMR (400 MHz, Chloroform-*d*) δ 9.98 (s, 1H), 8.78 – 8.71 (m, 2H), 8.13 (dd, *J* = 8.3, 1.7 Hz, 1H), 7.56 – 7.47 (m, 2H), 7.42 (dd, *J* = 8.3, 4.2 Hz, 1H), 7.21 – 7.13 (m, 2H), 6.98 – 6.90 (m, 2H), 6.05 (dd, *J* = 15.5, 8.7 Hz, 1H), 5.52 (d, *J* = 15.5 Hz, 1H), 3.47 – 3.33 (m, 2H), 2.95 (dt, *J* = 13.0, 6.7 Hz, 1H), 1.46 (s, 3H), 1.38 (s, 3H).

^{13}C NMR (101 MHz, Chloroform-*d*) δ 170.30, 162.84, 160.41, 148.23, 138.44, 136.43, 135.95, 134.51, 134.48, 134.13, 130.86, 130.78, 128.20, 127.96, 127.36, 123.19, 121.89, 121.74, 116.48, 115.32, 115.10, 53.45, 37.27, 34.69, 27.33, 27.30.
^{19}F NMR (376 MHz, Chloroform-*d*) δ -116.64.
HRMS (ESI-TOF): m/z Calcd for $C_{24}H_{23}FN_3O^+$ [M+H$^+$]: 388.1820; found: 388.1823.

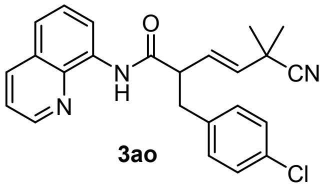

3ao

R$_f$ = 0.5, Petroleum Ether: Ethyl Acetate = 5: 1 (v/v)

Compound **3ao** was prepared following the general procedure in 68% yield (55 mg) as a yellow solid. m.p. 121-123 °C.

^{1}H NMR (400 MHz, Chloroform-*d*) δ 9.98 (s, 1H), 8.79 – 8.69 (m, 2H), 8.15 (dd, *J* = 8.3, 1.7 Hz, 1H), 7.56 – 7.48 (m, 2H), 7.44 (dd, *J* = 8.3, 4.2 Hz, 1H), 7.25 – 7.20 (m, 2H), 7.18 – 7.12 (m, 2H), 6.05 (dd, *J* = 15.4, 8.7 Hz, 1H), 5.55 (dd, *J* = 15.6, 0.7 Hz, 1H), 3.47 – 3.32 (m, 2H), 2.94 (dd, *J* = 13.1, 7.6 Hz, 1H), 1.47 (s, 3H), 1.40 (s, 3H).
^{13}C NMR (101 MHz, Chloroform-*d*) δ 170.20, 148.29, 138.51, 137.37, 136.51, 136.08, 134.16, 132.37, 130.78, 128.61, 128.21, 128.03, 127.44, 123.21, 121.97, 121.79, 116.59, 53.29, 37.48, 34.77, 27.41, 27.38.
HRMS (ESI-TOF): m/z Calcd for $C_{24}H_{23}ClN_3O^+$ [M+H$^+$]: 404.1524; found: 404.1521.

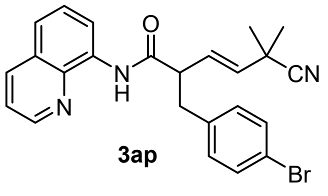

3ap

R$_f$ = 0.4, Petroleum Ether: Ethyl Acetate = 5: 1 (v/v)

Compound **3ap** was prepared following the general procedure in 58% yield (52 mg) as a yellow solid. m.p. 132-134 °C.

^{1}H NMR (400 MHz, Chloroform-*d*) δ 9.98 (s, 1H), 8.79 – 8.70 (m, 2H), 8.15 (dd, *J* = 8.3, 1.7 Hz, 1H), 7.57 – 7.49 (m, 2H), 7.44 (dd, *J* = 8.3, 4.3 Hz, 1H), 7.40 – 7.34 (m,

2H), 7.13 – 7.07 (m, 2H), 6.05 (dd, *J* = 15.5, 8.8 Hz, 1H), 5.55 (dd, *J* = 15.5, 0.8 Hz, 1H), 3.47 – 3.29 (m, 2H), 2.93 (dd, *J* = 13.4, 7.9 Hz, 1H), 1.47 (s, 3H), 1.40 (s, 3H).

^{13}C NMR (101 MHz, Chloroform-*d*) δ 170.18, 148.29, 138.49, 137.90, 136.54, 136.10, 134.14, 131.57, 131.18, 128.19, 128.04, 127.45, 123.22, 121.98, 121.80, 120.44, 116.61, 53.23, 37.54, 34.78, 27.42, 27.39.

HRMS (ESI-TOF): m/z Calcd for $C_{24}H_{23}BrN_3O^+$ [M+H$^+$]: 448.1019; found: 448.1027.

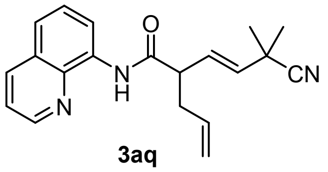

3aq

R$_f$ = 0.5, Petroleum Ether: Ethyl Acetate = 5: 1 (v/v)

Compound **3aq** was prepared following the general procedure in 80% yield (51 mg) as a white solid. m.p. 112-114 °C.

^{1}H NMR (400 MHz, Chloroform-*d*) δ 10.06 (s, 1H), 8.77 (ddd, *J* = 9.0, 5.5, 2.0 Hz, 2H), 8.16 (dd, *J* = 8.2, 1.7 Hz, 1H), 7.56 – 7.49 (m, 2H), 7.45 (dd, *J* = 8.3, 4.3 Hz, 1H), 6.06 (dd, *J* = 15.6, 8.8 Hz, 1H), 5.82 (ddt, *J* = 17.1, 10.1, 7.0 Hz, 1H), 5.74 (dd, *J* = 15.5, 0.9 Hz, 1H), 5.20 – 5.04 (m, 2H), 3.30 (dddd, *J* = 8.7, 7.3, 6.2, 0.8 Hz, 1H), 2.78 (dddt, *J* = 14.4, 7.4, 6.2, 1.3 Hz, 1H), 2.55 – 2.45 (m, 1H), 1.55 (s, 3H), 1.52 (s, 3H).

^{13}C NMR (101 MHz, Chloroform-*d*) δ 170.64, 148.27, 138.56, 136.55, 135.62, 135.03, 134.29, 128.66, 128.06, 127.50, 123.41, 121.86, 121.78, 117.66, 116.58, 51.08, 36.18, 34.81, 27.59, 27.44.

HRMS (ESI-TOF): m/z Calcd for $C_{20}H_{21}N_3NaO^+$ [M+Na$^+$]: 342.1577; found: 342.1579.

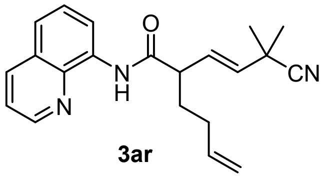

3ar

R$_f$ = 0.6, Petroleum Ether: Ethyl Acetate = 5: 1 (v/v)

Compound **3ar** was prepared following the general procedure in 72% yield (48 mg) as a white solid. m.p. 118-120 °C.

^{1}H NMR (400 MHz, Chloroform-*d*) δ 10.03 (s, 1H), 8.81 – 8.69 (m, 2H), 8.14 (dd, *J* = 8.3, 1.7 Hz, 1H), 7.55 – 7.46 (m, 2H), 7.43 (dd, *J* = 8.3, 4.2 Hz, 1H), 6.04 (dd, *J* = 15.5, 8.9 Hz, 1H), 5.87 – 5.75 (m, 1H), 5.73 (dd, *J* = 15.5, 0.8 Hz, 1H), 5.11 – 4.96 (m, 2H), 3.23 (td, *J* = 8.5, 5.2 Hz, 1H), 2.23 – 2.08 (m, 3H), 1.82 – 1.73 (m, 1H), 1.54 (s, 3H), 1.51 (s, 3H).

^{13}C NMR (101 MHz, Chloroform-*d*) δ 171.14, 148.24, 138.54, 137.60, 136.44, 135.39, 134.31, 129.03, 128.00, 127.41, 123.36, 121.76, 121.73, 116.45, 115.71, 50.64, 34.74, 31.28, 30.79, 27.54, 27.36.

HRMS (ESI-TOF): m/z Calcd for $C_{21}H_{23}N_3NaO^+$ [M+Na$^+$]: 356.1733; found: 356.1728.

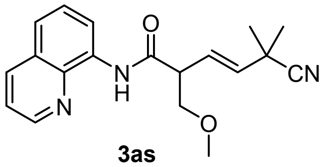

3as

R$_f$ = 0.4, Petroleum Ether: Ethyl Acetate = 2: 1 (v/v)

Compound **3as** was prepared following the general procedure in 66% yield (43 mg) as a yellow oil.

^{1}H NMR (400 MHz, Chloroform-*d*) δ 10.36 (s, 1H), 8.80 – 8.71 (m, 2H), 8.11 (dd, *J* = 8.3, 1.7 Hz, 1H), 7.53 – 7.45 (m, 2H), 7.41 (dd, *J* = 8.3, 4.2 Hz, 1H), 6.13 (dd, *J* = 15.6, 8.5 Hz, 1H), 5.77 (dd, *J* = 15.7, 0.9 Hz, 1H), 3.81 (dd, *J* = 9.2, 6.6 Hz, 1H), 3.75 (dd, *J* = 9.3, 5.0 Hz, 1H), 3.47 (dddd, *J* = 8.7, 6.3, 5.0, 1.0 Hz, 1H), 3.42 (s, 3H), 1.52 (s, 3H), 1.50 (s, 3H).

^{13}C NMR (101 MHz, Chloroform-*d*) δ 169.67, 148.23, 138.57, 136.34, 136.13, 134.40, 127.95, 127.32, 126.08, 123.32, 121.81, 121.66, 116.61, 72.88, 59.25, 51.45, 34.77, 27.45, 27.28.

HRMS (ESI-TOF): m/z Calcd for $C_{19}H_{22}N_3O_2^+$ [M+H$^+$]: 324.1707; found: 324.1701.

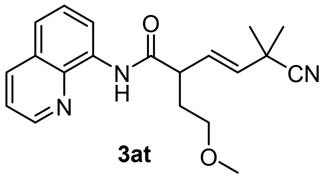

3at

R$_f$ = 0.5, Petroleum Ether: Ethyl Acetate = 2: 1 (v/v)

Compound **3at** was prepared following the general procedure in 62% yield (42 mg) as a white solid. m.p. 112-114 °C.

^{1}H NMR (400 MHz, Chloroform-*d*) δ 10.06 (s, 1H), 8.75 (ddd, *J* = 9.0, 5.5, 1.9 Hz, 2H), 8.13 (dd, *J* = 8.3, 1.7 Hz, 1H), 7.54 – 7.46 (m, 2H), 7.43 (dd, *J* = 8.2, 4.2 Hz, 1H), 6.06 (dd, *J* = 15.5, 9.0 Hz, 1H), 5.73 (dd, *J* = 15.5, 0.8 Hz, 1H), 3.52 – 3.41 (m, 3H), 3.32 (s, 3H), 2.30 (dtd, *J* = 14.0, 6.9, 5.4 Hz, 1H), 1.94 – 1.84 (m, 1H), 1.52 (s, 3H), 1.50 (s, 3H).

^{13}C NMR (101 MHz, Chloroform-*d*) δ 171.15, 148.22, 138.51, 136.43, 135.19, 134.36, 128.86, 128.00, 127.40, 123.38, 121.75, 121.72, 116.48, 69.73, 58.72, 47.91, 34.66, 31.85, 27.53, 27.30.

HRMS (ESI-TOF): m/z Calcd for $C_{20}H_{23}N_3NaO_2^+$ [M+Na$^+$]: 360.1682; found: 360.1682.

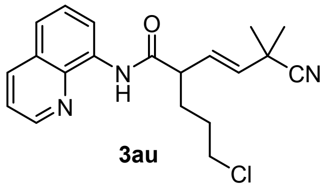

3au

R$_f$ = 0.4, Petroleum Ether: Ethyl Acetate = 5: 1 (v/v)

Compound **3au** was prepared following the general procedure in 59% yield (42 mg) as a yellow solid. m.p. 95-97 °C.

^{1}H NMR (400 MHz, Chloroform-*d*) δ 10.06 (s, 1H), 8.78 (dd, *J* = 4.2, 1.7 Hz, 1H), 8.74 (dd, *J* = 6.4, 2.6 Hz, 1H), 8.16 (dd, *J* = 8.3, 1.7 Hz, 1H), 7.57 – 7.49 (m, 2H), 7.45 (dd, *J* = 8.3, 4.2 Hz, 1H), 6.06 (dd, *J* = 15.5, 8.8 Hz, 1H), 5.77 (dd, *J* = 15.5, 0.8 Hz, 1H), 3.58 (tt, *J* = 7.4, 3.6 Hz, 2H), 3.23 (dt, *J* = 8.8, 6.7 Hz, 1H), 2.24 – 2.11 (m, 1H), 1.93 – 1.82 (m, 3H), 1.55 (s, 3H), 1.53 (s, 3H).

^{13}C NMR (101 MHz, Chloroform-*d*) δ 170.69, 148.33, 138.56, 136.54, 135.72, 134.23, 128.79, 128.05, 127.47, 123.32, 121.93, 121.82, 116.56, 50.82, 44.73, 34.83, 30.41, 29.23, 27.57, 27.42.

HRMS (ESI-TOF): m/z Calcd for $C_{20}H_{22}ClN_3NaO^+$ [M+Na$^+$]: 378.1344; found: 378.1338.

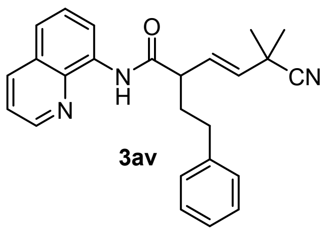

3av

R_f = 0.4, Petroleum Ether: Ethyl Acetate = 5: 1 (v/v)

Compound **3av** was prepared following the general procedure in 57% yield (44 mg) as a white solid. m.p. 100-102 °C.

^{1}H NMR (400 MHz, Chloroform-*d*) δ 10.04 (s, 1H), 8.84 – 8.71 (m, 2H), 8.15 (dd, *J* = 8.2, 1.7 Hz, 1H), 7.57 – 7.48 (m, 2H), 7.44 (dd, *J* = 8.3, 4.2 Hz, 1H), 7.34 – 7.27 (m, 2H), 7.25 – 7.18 (m, 3H), 6.09 (dd, *J* = 15.5, 8.9 Hz, 1H), 5.72 (dd, *J* = 15.5, 0.8 Hz, 1H), 3.25 – 3.17 (m, 1H), 2.83 – 2.67 (m, 2H), 2.44 (ddt, *J* = 13.5, 8.9, 6.7 Hz, 1H), 2.02 (dtd, *J* = 14.2, 8.4, 6.3 Hz, 1H), 1.55 (s, 3H), 1.53 (s, 3H).

^{13}C NMR (101 MHz, Chloroform-*d*) δ 170.98, 148.22, 141.15, 138.51, 136.42, 135.49, 134.26, 129.00, 128.60, 128.53, 127.97, 127.39, 126.12, 123.33, 121.78, 121.72, 116.45, 50.54, 34.73, 33.26, 33.12, 27.52, 27.30.

HRMS (ESI-TOF): m/z Calcd for $C_{25}H_{25}N_3NaO^+$ [M+Na$^+$]: 406.1890; found: 406.1899.

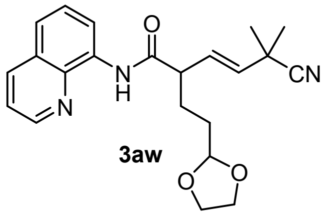

3aw

R_f = 0.4, Petroleum Ether: Ethyl Acetate = 3: 1 (v/v)

Compound **3aw** was prepared following the general procedure in 72% yield (55 mg) as a yellow solid. m.p. 101-103 °C.

^{1}H NMR (400 MHz, Chloroform-*d*) δ 10.06 (s, 1H), 8.76 (ddd, *J* = 8.9, 5.5, 2.0 Hz, 2H), 8.15 (dd, *J* = 8.3, 1.7 Hz, 1H), 7.55 – 7.48 (m, 2H), 7.44 (dd, *J* = 8.3, 4.2 Hz, 1H), 6.05 (dd, *J* = 15.5, 8.8 Hz, 1H), 5.74 (dd, *J* = 15.6, 0.8 Hz, 1H), 4.92 (t, *J* = 4.4 Hz, 1H), 4.02 – 3.92 (m, 2H), 3.89 – 3.81 (m, 2H), 3.29 (q, *J* = 8.5, 7.6 Hz, 1H), 2.17 (dtt, *J* = 13.6, 6.8, 4.4 Hz, 1H), 1.87 – 1.79 (m, 1H), 1.78 (dt, *J* = 6.7, 2.9 Hz, 2H), 1.53 (s, 3H), 1.51 (s, 3H).

^{13}C NMR (101 MHz, Chloroform-*d*) δ 171.14, 148.21, 138.53, 136.56, 135.27, 134.36, 129.13, 128.07, 127.51, 123.42, 121.80, 121.74, 116.64, 104.34, 65.06, 65.01, 51.08, 34.75, 31.42, 27.56, 27.43, 26.37.

HRMS (ESI-TOF): m/z Calcd for $C_{22}H_{26}N_3O_3^+$ [M+H$^+$]: 380.1969; found: 380.1965.

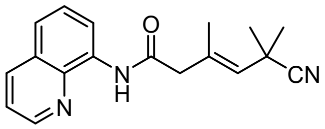

3ax

R$_f$ = 0.5, Petroleum Ether: Ethyl Acetate = 5: 1 (v/v)

Compound **3ax** was prepared following the general procedure in 68% yield (40 mg) as a white solid. m.p. 132-134 °C.

^{1}H NMR (400 MHz, Chloroform-*d*) δ 10.04 (s, 1H), 8.79 (dd, *J* = 4.2, 1.7 Hz, 1H), 8.76 (dd, *J* = 6.8, 2.2 Hz, 1H), 8.15 (dd, *J* = 8.3, 1.7 Hz, 1H), 7.56 – 7.47 (m, 2H), 7.44 (dd, *J* = 8.3, 4.2 Hz, 1H), 5.43 – 5.34 (m, 1H), 3.71 (d, *J* = 0.8 Hz, 2H), 1.93 (d, *J* = 1.5 Hz, 3H), 1.58 (s, 6H).

^{13}C NMR (101 MHz, Chloroform-*d*) δ 168.02, 148.35, 138.58, 136.42, 134.47, 134.10, 130.26, 128.04, 127.46, 124.69, 121.78, 121.74, 116.62, 41.28, 31.00, 29.41, 25.14.

HRMS (ESI-TOF): m/z Calcd for $C_{18}H_{20}N_3O^+$ [M+H$^+$]: 294.1601; found: 294.1607.

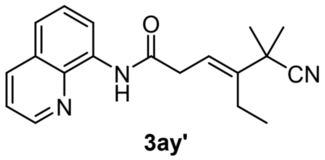

3ay'

R$_f$ = 0.4, Petroleum Ether: Ethyl Acetate = 5: 1 (v/v)

Compound **3ay'** was prepared following the general procedure in 62% yield (38 mg) as a white solid. m.p. 130-132 °C.

^{1}H NMR (400 MHz, Chloroform-*d*) δ 10.06 (s, 1H), 8.78 – 8.70 (m, 2H), 8.14 (dd, *J* = 8.3, 1.7 Hz, 1H), 7.55 – 7.47 (m, 2H), 7.43 (dd, *J* = 8.3, 4.2 Hz, 1H), 5.97 (t, *J* = 7.6 Hz, 1H), 3.37 (d, *J* = 7.6 Hz, 2H), 2.34 (q, *J* = 7.6 Hz, 2H), 1.63 (s, 6H), 1.18 (t, *J* = 7.6 Hz, 3H).

^{13}**C NMR** (101 MHz, Chloroform-*d*) δ 168.86, 148.24, 145.87, 138.48, 136.46, 134.23, 127.99, 127.43, 124.20, 121.79, 121.75, 118.63, 116.45, 38.94, 37.27, 27.06, 21.93, 14.79.

HRMS (ESI-TOF): m/z Calcd for $C_{19}H_{21}N_3NaO^+$ [M+Na$^+$]: 330.1577; found: 330.1571.

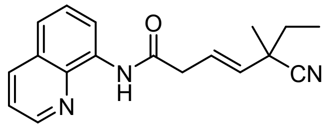

3az

R$_f$ = 0.3, Petroleum Ether: Ethyl Acetate = 4: 1 (v/v)

Compound **3az** was prepared following the general procedure in 73% yield (43 mg) as a white solid. m.p. 104-106 °C.

^{1}**H NMR** (400 MHz, Chloroform-*d*) δ 10.00 (s, 1H), 8.77 – 8.70 (m, 2H), 8.12 (dd, *J* = 8.3, 1.7 Hz, 1H), 7.54 – 7.46 (m, 2H), 7.42 (dd, *J* = 8.3, 4.2 Hz, 1H), 6.17 (dt, *J* = 15.5, 7.2 Hz, 1H), 5.60 (dt, *J* = 15.5, 1.4 Hz, 1H), 3.34 (dd, *J* = 7.2, 1.4 Hz, 2H), 1.80 – 1.67 (m, 2H), 1.51 (s, 3H), 1.10 (t, *J* = 7.5 Hz, 3H).

^{13}**C NMR** (101 MHz, Chloroform-*d*) δ 168.52, 148.20, 138.46, 136.39, 135.93, 134.23, 127.95, 127.37, 124.30, 122.34, 121.79, 121.72, 116.42, 41.39, 40.55, 33.42, 25.59, 9.70.

HRMS (ESI-TOF): m/z Calcd for $C_{18}H_{20}N_3O^+$ [M+H$^+$]: 294.1601; found: 294.1609.

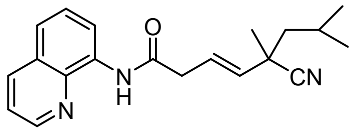

3az'

R$_f$ = 0.4, Petroleum Ether: Ethyl Acetate = 5: 1 (v/v)

Compound **3az'** was prepared following the general procedure in 63% yield (40 mg) as a white solid. m.p. 88-90 °C.

^{1}**H NMR** (400 MHz, Chloroform-*d*) δ 9.98 (s, 1H), 8.78 – 8.70 (m, 2H), 8.13 (dd, *J* = 8.3, 1.7 Hz, 1H), 7.54 – 7.46 (m, 2H), 7.42 (dd, *J* = 8.3, 4.3 Hz, 1H), 6.20 (dt, *J* = 15.5, 7.2 Hz, 1H), 5.62 (dt, *J* = 15.5, 1.3 Hz, 1H), 3.34 (dd, *J* = 7.2, 1.4 Hz, 2H), 1.87 (dt, *J* = 13.1, 6.7 Hz, 1H), 1.67 – 1.54 (m, 2H), 1.52 (s, 3H), 1.01 (d, *J* = 6.7 Hz, 3H), 0.96 (d, *J* = 6.6 Hz, 3H).

^{13}C NMR (101 MHz, Chloroform-*d*) δ 168.55, 148.18, 138.42, 136.52, 136.44, 134.23, 127.97, 127.39, 123.54, 122.96, 121.80, 121.71, 116.49, 48.72, 41.36, 38.99, 27.26, 25.72, 23.94, 23.65.

HRMS (ESI-TOF): m/z Calcd for $C_{20}H_{23}N_3NaO^+$ [M+Na$^+$]: 344.1733; found: 344.1735.

6. General Procedures for the Synthesis of *β*-Lactams

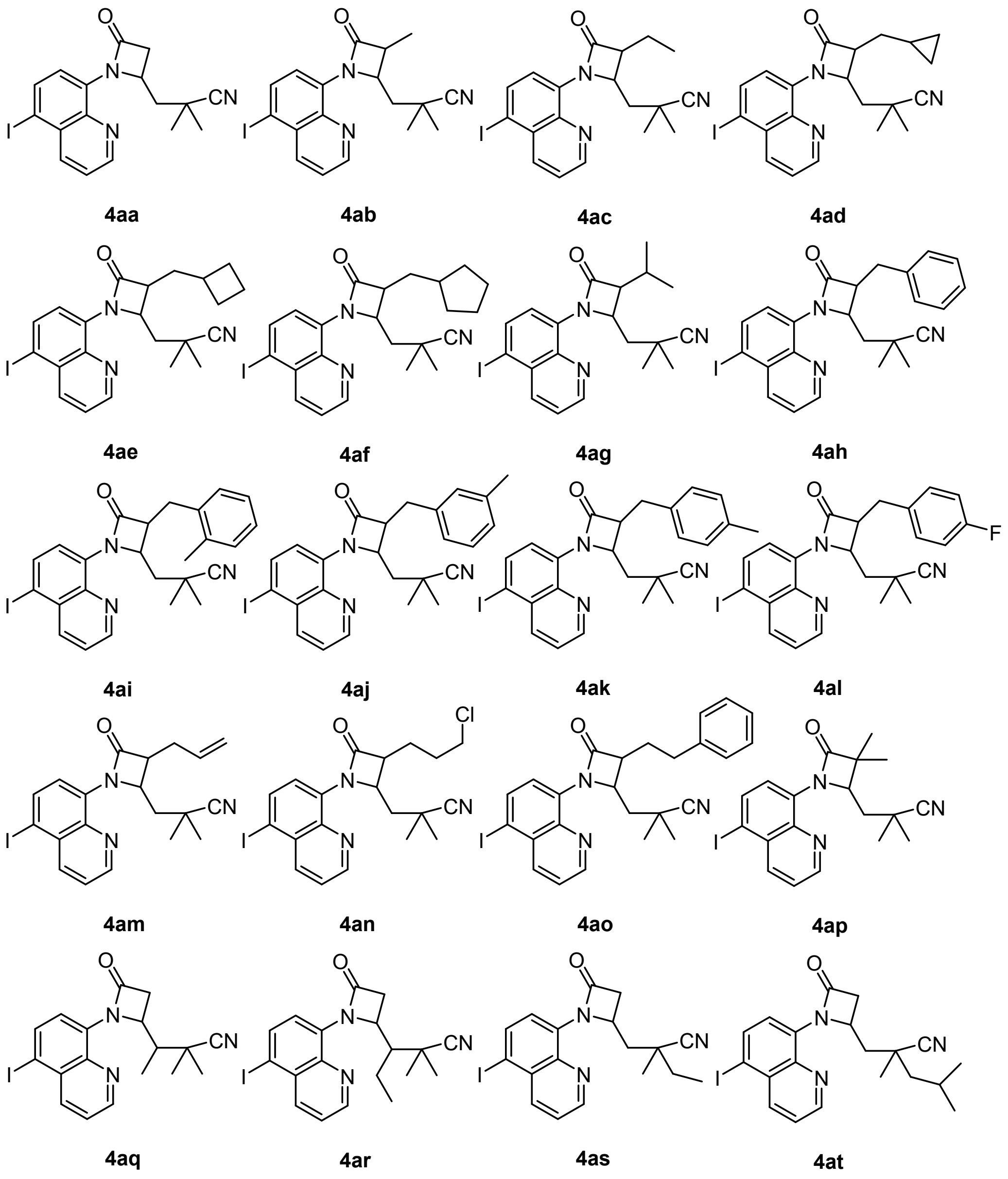

Scheme S13. Products of the Synthesis of *β*-Lactams

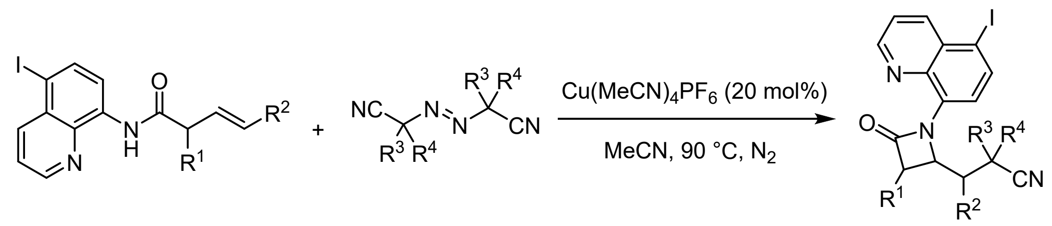

Scheme S14. Synthesis of products of the Synthesis of *β*-Lactams

The alkenes substrate (0.2 mmol, 1 equiv), azo compounds (0.8 mmol, 4 equiv), $Cu(MeCN)_4PF_6$ (15mg, 0.04 mmol, 0.2 equiv), MeCN (2 mL) were added into a 15 mL sealed glass vial under N_2 atmosphere. The reaction mixture was heated at 90 °C for 12 h. After being cooled to room temperature, the mixture was concentrated *in vacuo* and purified by silica gel (300 - 400 mesh) column chromatography to give the desired products.

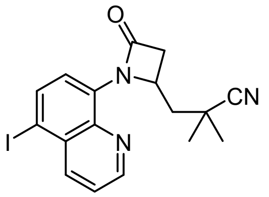

4aa

R_f = 0.4, Petroleum Ether: Ethyl Acetate = 4: 1 (v/v)

Compound **4aa** was prepared following the general procedure in 89% yield (72 mg) as a yellow solid. m.p. 132-134 °C.

^{1}H NMR (400 MHz, Chloroform-*d*) δ 8.85 (dt, *J* = 3.6, 1.7 Hz, 1H), 8.35 (ddd, *J* = 8.6, 2.9, 1.5 Hz, 1H), 8.11 (dd, *J* = 8.2, 1.5 Hz, 1H), 8.04 (dd, *J* = 8.3, 2.3 Hz, 1H), 7.49 (ddd, *J* = 8.6, 4.1, 1.7 Hz, 1H), 5.46 (ddt, *J* = 12.9, 4.6, 2.2 Hz, 1H), 3.52 (dd, *J* = 15.3, 5.2 Hz, 1H), 3.02 (ddd, *J* = 15.4, 2.6, 1.0 Hz, 1H), 2.39 (dt, *J* = 13.6, 1.5 Hz, 1H), 1.71 – 1.65 (m, 1H), 1.45 (d, *J* = 1.0 Hz, 3H), 1.38 (d, *J* = 1.0 Hz, 3H).

^{13}C NMR (101 MHz, Chloroform-*d*) δ 165.77, 149.79, 140.64, 140.56, 137.86, 134.03, 130.59, 124.28, 123.23, 122.14, 92.04, 54.20, 45.58, 44.16, 30.82, 27.33, 27.14.

HRMS (ESI-TOF): m/z Calcd for $C_{17}H_{16}IN_3NaO^+$ [M+Na$^+$]: 428.0230; found: 428.0237.

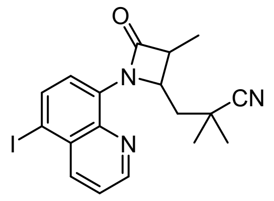

4ab

R_f = 0.5, Petroleum Ether: Ethyl Acetate = 4: 1 (v/v)

Compound **4ab** was prepared following the general procedure in 81% yield (68 mg) as a yellow oil.

^{1}H NMR (400 MHz, Chloroform-*d*) δ 8.84 (dd, *J* = 4.1, 1.7 Hz, 1H), 8.36 (dd, *J* = 8.6, 1.6 Hz, 1H), 8.12 (d, *J* = 8.2 Hz, 1H), 8.05 (d, *J* = 8.2 Hz, 1H), 7.49 (dd, *J* = 8.6, 4.1 Hz, 1H), 5.07 (dt, *J* = 10.8, 2.0 Hz, 1H), 3.18 (qd, *J* = 7.3, 2.2 Hz, 1H), 2.33 (dd, *J* = 13.6, 1.7 Hz, 1H), 1.74 (dd, *J* = 13.6, 10.7 Hz, 1H), 1.58 (d, *J* = 7.3 Hz, 3H), 1.48 (s, 3H), 1.39 (s, 3H).

^{13}C NMR (101 MHz, Chloroform-*d*) δ 169.59, 149.73, 140.72, 140.64, 137.89, 134.00, 130.64, 124.39, 123.19, 122.51, 91.94, 62.41, 53.15, 44.23, 30.57, 27.58, 27.18, 13.97.

HRMS (ESI-TOF): m/z Calcd for $C_{18}H_{18}IN_3NaO^+$ [M+Na$^+$]: 442.0387; found: 442.0381.

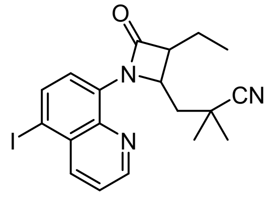

4ac

R_f = 0.6, Petroleum Ether: Ethyl Acetate = 4: 1 (v/v)

Compound **4ac** was prepared following the general procedure in 78% yield (68 mg) as a yellow oil.

^{1}H NMR (400 MHz, Chloroform-*d*) δ 8.84 (dd, *J* = 4.1, 1.6 Hz, 1H), 8.36 (dd, *J* = 8.6, 1.6 Hz, 1H), 8.14 (d, *J* = 8.2 Hz, 1H), 8.05 (d, *J* = 8.2 Hz, 1H), 7.49 (dd, *J* = 8.6, 4.1 Hz, 1H), 5.15 (dt, *J* = 10.6, 2.0 Hz, 1H), 3.18 (ddd, *J* = 7.7, 5.4, 2.2 Hz, 1H), 2.31 (dd, *J* = 13.6, 1.9 Hz, 1H), 2.11 – 1.95 (m, 2H), 1.75 (dd, *J* = 13.6, 10.7 Hz, 1H), 1.46 (s, 3H), 1.38 (s, 3H), 1.19 (t, *J* = 7.5 Hz, 3H).

^{13}**C NMR** (101 MHz, Chloroform-*d*) δ 169.17, 149.72, 140.78, 140.64, 137.92, 134.01, 130.64, 124.40, 123.17, 122.43, 91.89, 59.61, 59.27, 44.17, 30.56, 27.74, 27.14, 22.36, 11.24.

HRMS (ESI-TOF): m/z Calcd for $C_{19}H_{21}IN_3O^+$ [M+H$^+$]: 434.0724; found: 434.0725.

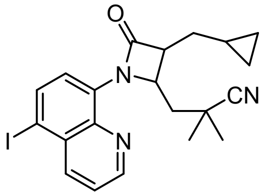

4ad

R$_f$ = 0.4, Petroleum Ether: Ethyl Acetate = 8: 1 (v/v)

Compound **4ad** was prepared following the general procedure in 84% yield (77 mg) as a yellow oil.

^{1}**H NMR** (400 MHz, Chloroform-*d*) δ 8.83 (dd, *J* = 4.1, 1.6 Hz, 1H), 8.35 (dd, *J* = 8.6, 1.7 Hz, 1H), 8.15 (d, *J* = 8.2 Hz, 1H), 8.05 (d, *J* = 8.2 Hz, 1H), 7.48 (dd, *J* = 8.6, 4.1 Hz, 1H), 5.30 (dt, *J* = 10.7, 2.0 Hz, 1H), 3.30 (ddd, *J* = 7.6, 5.5, 2.2 Hz, 1H), 2.32 (dd, *J* = 13.6, 1.9 Hz, 1H), 2.00 (ddd, *J* = 14.2, 7.6, 6.4 Hz, 1H), 1.85 – 1.78 (m, 1H), 1.77 – 1.69 (m, 1H), 1.46 (s, 3H), 1.39 (s, 3H), 1.03 (qdt, *J* = 7.9, 6.5, 4.9 Hz, 1H), 0.61 – 0.45 (m, 2H), 0.20 (dq, *J* = 4.8, 1.7 Hz, 2H).

^{13}**C NMR** (101 MHz, Chloroform-*d*) δ 169.20, 149.67, 140.74, 140.59, 137.89, 134.03, 130.60, 124.38, 123.15, 122.37, 91.84, 59.63, 58.31, 44.28, 34.13, 30.55, 27.68, 27.16, 8.51, 5.29, 4.74.

HRMS (ESI-TOF): m/z Calcd for $C_{21}H_{23}IN_3O^+$ [M+H$^+$]: 460.0880; found: 460.0883.

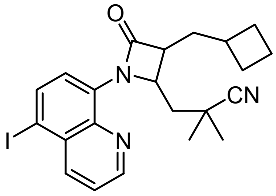

4ae

R$_f$ = 0.4, Petroleum Ether: Ethyl Acetate = 8: 1 (v/v)

Compound **4ae** was prepared following the general procedure in 85% yield (80 mg) as a yellow oil.

^{1}**H NMR** (400 MHz, Chloroform-*d*) δ 8.84 (dd, *J* = 4.1, 1.7 Hz, 1H), 8.36 (dd, *J* = 8.6, 1.6 Hz, 1H), 8.13 (d, *J* = 8.3 Hz, 1H), 8.05 (d, *J* = 8.3 Hz, 1H), 7.49 (dd, *J* = 8.6, 4.1

Hz, 1H), 5.14 (dt, J = 10.6, 2.0 Hz, 1H), 3.13 (ddd, J = 7.9, 5.5, 2.2 Hz, 1H), 2.70 (p, J = 7.8 Hz, 1H), 2.29 (dd, J = 13.6, 1.9 Hz, 1H), 2.21 – 2.04 (m, 4H), 1.92 – 1.79 (m, 2H), 1.78 – 1.68 (m, 3H), 1.46 (s, 3H), 1.38 (s, 3H).

^{13}C NMR (101 MHz, Chloroform-d) δ 169.25, 149.70, 140.74, 140.69, 137.95, 134.02, 130.65, 123.18, 122.42, 91.87, 59.90, 56.66, 44.36, 36.42, 33.64, 30.59, 29.26, 28.48, 27.66, 27.22, 18.61.

HRMS (ESI-TOF): m/z Calcd for $C_{22}H_{25}IN_3O^+$ [M+H$^+$]: 474.1037; found: 474.1037.

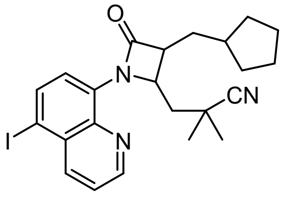

4af

R$_f$ = 0.4, Petroleum Ether: Ethyl Acetate = 8: 1 (v/v)

Compound **4af** was prepared following the general procedure in 85% yield (83 mg) as a yellow oil.

^{1}H NMR (400 MHz, Chloroform-d) δ 8.83 (dd, J = 4.1, 1.7 Hz, 1H), 8.36 (dd, J = 8.6, 1.7 Hz, 1H), 8.15 (d, J = 8.2 Hz, 1H), 8.06 (d, J = 8.2 Hz, 1H), 7.48 (dd, J = 8.6, 4.1 Hz, 1H), 5.12 (dt, J = 10.6, 2.0 Hz, 1H), 3.22 (ddd, J = 10.1, 4.8, 2.1 Hz, 1H), 2.35 – 2.19 (m, 2H), 2.10 (ddd, J = 13.4, 10.2, 5.4 Hz, 1H), 2.01 – 1.84 (m, 3H), 1.74 (dd, J = 13.7, 10.6 Hz, 1H), 1.68 – 1.51 (m, 5H), 1.46 (s, 3H), 1.39 (s, 3H), 1.21 (q, J = 11.3, 9.4 Hz, 2H).

^{13}C NMR (101 MHz, Chloroform-d) δ 169.45, 149.69, 140.77, 140.62, 137.93, 134.12, 130.62, 124.43, 123.16, 122.39, 91.82, 60.72, 57.58, 44.32, 37.92, 36.21, 33.65, 32.48, 30.57, 27.70, 27.20, 25.27, 25.23.

HRMS (ESI-TOF): m/z Calcd for $C_{23}H_{26}IN_3NaO^+$ [M+Na$^+$]: 510.1013; found: 510.1011.

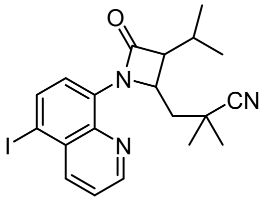

4ag

R$_f$ = 0.3, Petroleum Ether: Ethyl Acetate = 8: 1 (v/v)

Compound **4ag** was prepared following the general procedure in 60% yield (54 mg) as a yellow oil.

^{1}H NMR (400 MHz, Chloroform-*d*) δ 8.84 (dd, *J* = 4.1, 1.7 Hz, 1H), 8.36 (dd, *J* = 8.6, 1.6 Hz, 1H), 8.15 (d, *J* = 8.2 Hz, 1H), 8.05 (d, *J* = 8.2 Hz, 1H), 7.49 (dd, *J* = 8.6, 4.1 Hz, 1H), 5.20 (dt, *J* = 10.4, 2.1 Hz, 1H), 3.16 (dd, *J* = 5.3, 2.1 Hz, 1H), 2.35 – 2.22 (m, 2H), 1.76 (dd, *J* = 13.7, 10.5 Hz, 1H), 1.45 (s, 3H), 1.37 (s, 3H), 1.23 (d, *J* = 6.9 Hz, 3H), 1.18 (d, *J* = 6.7 Hz, 3H).

^{13}C NMR (101 MHz, Chloroform-*d*) δ 168.46, 149.71, 140.87, 140.64, 137.93, 133.93, 130.63, 124.42, 123.16, 122.44, 91.92, 64.16, 58.27, 44.17, 30.48, 28.39, 28.01, 27.03, 21.67, 18.70.

HRMS (ESI-TOF): m/z Calcd for $C_{20}H_{22}IN_3NaO^+$ [M+Na$^+$]: 470.0700; found: 470.0708.

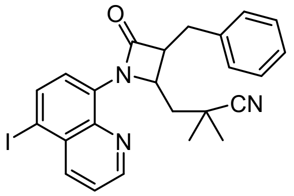

4ah

R$_f$ = 0.6, Petroleum Ether: Ethyl Acetate = 4: 1 (v/v)

Compound **4ah** was prepared following the general procedure in 66% yield (65 mg) as a yellow solid. m.p. 140-142 °C.

^{1}H NMR (400 MHz, Chloroform-*d*) δ 8.80 (dd, *J* = 4.1, 1.7 Hz, 1H), 8.34 (dd, *J* = 8.6, 1.7 Hz, 1H), 8.11 (d, *J* = 8.2 Hz, 1H), 8.04 (d, *J* = 8.2 Hz, 1H), 7.47 (dd, *J* = 8.6, 4.1 Hz, 1H), 7.42 – 7.35 (m, 2H), 7.34 – 7.27 (m, 2H), 7.24 – 7.16 (m, 1H), 5.20 (dt, *J* = 10.6, 2.0 Hz, 1H), 3.43 (ddd, *J* = 7.2, 6.2, 2.1 Hz, 1H), 3.31 (d, *J* = 6.7 Hz, 2H), 2.27 (dd, *J* = 13.7, 1.9 Hz, 1H), 1.72 (dd, *J* = 13.7, 10.6 Hz, 1H), 1.34 (s, 3H), 1.16 (s, 3H).

^{13}C NMR (101 MHz, Chloroform-*d*) δ 168.42, 149.71, 140.71, 140.57, 138.07, 137.85, 133.83, 130.60, 129.56, 128.77, 126.86, 124.41, 123.17, 122.48, 92.13, 59.70, 59.33, 44.11, 35.42, 30.55, 27.25, 27.11.

HRMS (ESI-TOF): m/z Calcd for $C_{24}H_{22}IN_3NaO^+$ [M+Na$^+$]: 518.0700; found: 518.0702.

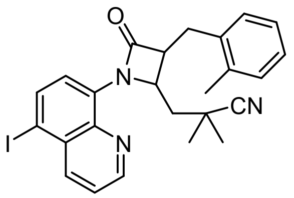

4ai

R_f = 0.4, Petroleum Ether: Ethyl Acetate = 8: 1 (v/v)

Compound **4ai** was prepared following the general procedure in 71% yield (72 mg) as a yellow oil.

^{1}H NMR (400 MHz, Chloroform-*d*) δ 8.88 (dd, *J* = 4.1, 1.7 Hz, 1H), 8.37 (dd, *J* = 8.6, 1.7 Hz, 1H), 8.15 (d, *J* = 8.2 Hz, 1H), 8.06 (d, *J* = 8.2 Hz, 1H), 7.50 (dd, *J* = 8.6, 4.1 Hz, 1H), 7.39 (dd, *J* = 7.0, 1.6 Hz, 1H), 7.22 – 7.10 (m, 3H), 5.33 – 5.26 (m, 1H), 3.45 – 3.33 (m, 2H), 3.26 (tt, *J* = 11.1, 5.7 Hz, 1H), 2.42 (s, 3H), 2.30 (dd, *J* = 13.8, 1.8 Hz, 1H), 1.67 (dd, *J* = 13.8, 10.5 Hz, 1H), 1.25 (s, 3H), 1.04 (s, 3H).

^{13}C NMR (101 MHz, Chloroform-*d*) δ 168.46, 149.89, 140.74, 140.61, 137.87, 136.43, 136.41, 133.91, 130.66, 130.64, 130.42, 127.13, 126.40, 124.28, 123.24, 122.50, 92.18, 60.57, 58.16, 44.13, 33.23, 30.58, 27.42, 26.43, 19.77.

HRMS (ESI-TOF): m/z Calcd for $C_{25}H_{24}IN_3NaO^+$ [M+Na$^+$]: 532.0856; found: 532.0855.

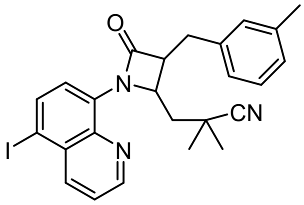

4aj

R_f = 0.4, Petroleum Ether: Ethyl Acetate = 8: 1 (v/v)

Compound **4aj** was prepared following the general procedure in 55% yield (56 mg) as a yellow oil.

^{1}H NMR (400 MHz, Chloroform-*d*) δ 8.83 (dd, *J* = 4.1, 1.7 Hz, 1H), 8.36 (dd, *J* = 8.6, 1.6 Hz, 1H), 8.10 (d, *J* = 8.2 Hz, 1H), 8.05 (d, *J* = 8.2 Hz, 1H), 7.48 (dd, *J* = 8.6, 4.1 Hz, 1H), 7.23 – 7.15 (m, 3H), 7.02 (dt, *J* = 7.1, 2.0 Hz, 1H), 5.20 (dt, *J* = 10.6, 2.0 Hz, 1H), 3.41 (td, *J* = 6.9, 2.1 Hz, 1H), 3.32 – 3.21 (m, 2H), 2.32 (s, 3H), 2.28 (dd, *J* = 13.7, 1.9 Hz, 1H), 1.71 (dd, *J* = 13.7, 10.6 Hz, 1H), 1.32 (s, 3H), 1.13 (s, 3H).

^{13}C NMR (101 MHz, Chloroform-*d*) δ 168.49, 149.68, 140.68, 140.62, 138.38, 137.99, 137.88, 133.80, 130.61, 130.32, 128.66, 127.59, 126.50, 124.39, 123.17, 122.52, 92.14, 59.86, 59.36, 44.07, 35.43, 30.57, 27.34, 26.90, 21.50.

HRMS (ESI-TOF): m/z Calcd for $C_{25}H_{24}IN_3NaO^+$ [M+Na$^+$]: 532.0856; found: 532.0866.

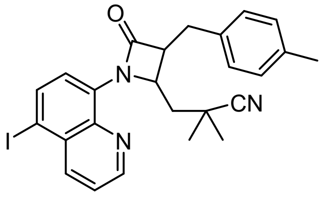

4ak

R$_f$ = 0.6, Petroleum Ether: Ethyl Acetate = 4: 1 (v/v)

Compound **4ak** was prepared following the general procedure in 72% yield (73 mg) as a white solid. m.p. 144-146 °C.

^{1}H NMR (400 MHz, Chloroform-*d*) δ 8.81 (dd, *J* = 4.1, 1.7 Hz, 1H), 8.34 (dd, *J* = 8.6, 1.6 Hz, 1H), 8.11 (d, *J* = 8.2 Hz, 1H), 8.04 (d, *J* = 8.2 Hz, 1H), 7.47 (dd, *J* = 8.6, 4.1 Hz, 1H), 7.26 (d, *J* = 8.0 Hz, 2H), 7.11 (d, *J* = 7.8 Hz, 2H), 5.19 (dt, *J* = 10.6, 2.0 Hz, 1H), 3.40 (ddd, *J* = 8.1, 6.2, 2.1 Hz, 1H), 3.27 (d, *J* = 6.3 Hz, 2H), 2.32 – 2.24 (m, 4H), 1.71 (dd, *J* = 13.7, 10.6 Hz, 1H), 1.35 (s, 3H), 1.17 (s, 3H).

^{13}C NMR (101 MHz, Chloroform-*d*) δ 168.52, 149.71, 140.72, 140.57, 137.87, 136.33, 134.95, 133.92, 130.61, 129.44, 129.41, 124.44, 123.16, 122.46, 92.05, 59.78, 59.49, 44.14, 35.03, 30.58, 27.31, 27.10, 21.19.

HRMS (ESI-TOF): m/z Calcd for $C_{25}H_{25}IN_3O^+$ [M+H$^+$]: 510.1037; found: 510.1033.

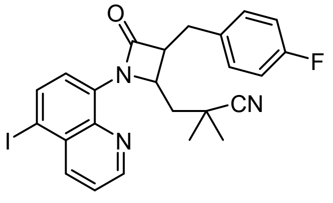

4al

R$_f$ = 0.6, Petroleum Ether: Ethyl Acetate = 4: 1 (v/v)

Compound **4al** was prepared following the general procedure in 57% yield (58 mg) as a yellow solid. m.p. 158-160 °C.

^{1}H NMR (400 MHz, Chloroform-*d*) δ 8.78 (dd, *J* = 4.5, 1.6 Hz, 1H), 8.35 (dt, *J* = 8.7, 1.4 Hz, 1H), 8.08 (d, *J* = 8.2 Hz, 1H), 8.06 – 8.02 (m, 1H), 7.47 (dd, *J* = 8.6, 4.1 Hz,

1H), 7.38 – 7.31 (m, 2H), 7.02 – 6.93 (m, 2H), 5.16 (dt, *J* = 10.7, 2.1 Hz, 1H), 3.43 (ddd, *J* = 7.7, 5.3, 2.2 Hz, 1H), 3.29 (qd, *J* = 14.2, 6.6 Hz, 2H), 2.25 (dd, *J* = 13.7, 2.0 Hz, 1H), 1.76 (dd, *J* = 13.7, 10.7 Hz, 1H), 1.40 (s, 3H), 1.23 (s, 3H).

^{13}C NMR (101 MHz, Chloroform-*d*) δ 168.25, 163.13, 160.70, 149.66, 140.68, 137.87, 133.73, 133.66, 131.13, 131.05, 130.63, 124.42, 123.20, 122.56, 115.62, 115.41, 92.28, 59.39, 59.19, 44.11, 34.44, 30.50, 27.52, 27.08.

^{19}F NMR (376 MHz, Chloroform-*d*) δ -116.38.

HRMS (ESI-TOF): m/z Calcd for $C_{24}H_{21}FIN_3NaO^+$ [M+Na$^+$]: 536.0606; found: 536.0608.

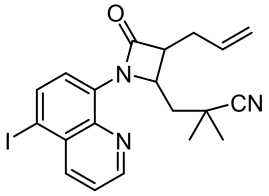

4am

R$_f$ = 0.6, Petroleum Ether: Ethyl Acetate = 4: 1 (v/v)

Compound **4am** was prepared following the general procedure in 75% yield (68 mg) as a yellow oil.

^{1}H NMR (400 MHz, Chloroform-*d*) δ 8.83 (dd, *J* = 4.1, 1.7 Hz, 1H), 8.36 (dd, *J* = 8.6, 1.7 Hz, 1H), 8.14 (d, *J* = 8.3 Hz, 1H), 8.06 (d, *J* = 8.2 Hz, 1H), 7.49 (dd, *J* = 8.6, 4.2 Hz, 1H), 6.00 (ddt, *J* = 17.1, 10.1, 7.1 Hz, 1H), 5.28 (dq, *J* = 17.0, 1.5 Hz, 1H), 5.22 – 5.12 (m, 2H), 3.31 (ddd, *J* = 6.8, 6.0, 2.2 Hz, 1H), 2.75 (dtt, *J* = 7.0, 5.5, 1.3 Hz, 2H), 2.31 (dd, *J* = 13.7, 1.9 Hz, 1H), 1.77 (dd, *J* = 13.6, 10.7 Hz, 1H), 1.47 (s, 3H), 1.39 (s, 3H).

^{13}C NMR (101 MHz, Chloroform-*d*) δ 168.40, 149.72, 140.73, 140.65, 137.91, 134.15, 133.94, 130.65, 124.44, 123.19, 122.46, 118.39, 92.01, 59.31, 57.38, 44.09, 33.31, 30.51, 27.91, 26.97.

HRMS (ESI-TOF): m/z Calcd for $C_{20}H_{20}IN_3NaO^+$ [M+Na$^+$]: 468.0543; found: 468.0548.

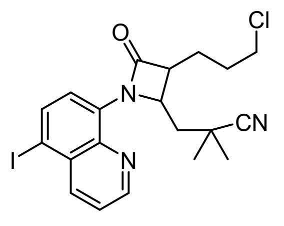

4an

R_f = 0.5, Petroleum Ether: Ethyl Acetate = 4: 1 (v/v)

Compound **4an** was prepared following the general procedure in 22% yield (21 mg) as a white solid. m.p. 150-152 °C.

^{1}H NMR (400 MHz, Chloroform-*d*) δ 8.84 (dd, *J* = 4.2, 1.6 Hz, 1H), 8.36 (dd, *J* = 8.6, 1.6 Hz, 1H), 8.06 (s, 2H), 7.49 (dd, *J* = 8.6, 4.1 Hz, 1H), 5.18 (d, *J* = 9.8 Hz, 1H), 2.66 – 2.50 (m, 3H), 2.43 (ddt, *J* = 11.9, 7.4, 3.6 Hz, 1H), 2.27 (dp, *J* = 11.4, 8.9 Hz, 1H), 2.06 – 1.96 (m, 2H), 1.77 (dd, *J* = 14.2, 10.0 Hz, 1H), 1.71 – 1.65 (m, 1H), 1.53 (s, 3H), 1.44 (s, 3H).

^{13}C NMR (101 MHz, Chloroform-*d*) δ 172.47, 149.73, 141.06, 140.81, 137.98, 133.69, 130.67, 125.02, 123.24, 123.17, 92.14, 64.19, 59.71, 40.27, 30.94, 28.26, 27.76, 27.52, 25.13, 16.47.

HRMS (ESI-TOF): m/z Calcd for $C_{20}H_{22}ClIN_3O^+$ [M+H$^+$]: 482.0491; found: 482.0482.

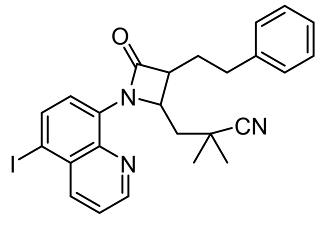

4ao

R_f = 0.4, Petroleum Ether: Ethyl Acetate = 8: 1 (v/v)

Compound **4ao** was prepared following the general procedure in 83% yield (85 mg) as a yellow oil.

^{1}H NMR (400 MHz, Chloroform-*d*) δ 8.83 (dd, *J* = 4.2, 1.7 Hz, 1H), 8.37 (dd, *J* = 8.6, 1.6 Hz, 1H), 8.15 (d, *J* = 8.2 Hz, 1H), 8.07 (d, *J* = 8.2 Hz, 1H), 7.49 (dd, *J* = 8.6, 4.1 Hz, 1H), 7.27 (d, *J* = 4.4 Hz, 4H), 7.19 (h, *J* = 4.0 Hz, 1H), 5.16 (dt, *J* = 10.7, 2.0 Hz, 1H), 3.23 (ddd, *J* = 9.3, 5.1, 2.2 Hz, 1H), 3.13 – 3.02 (m, 1H), 2.88 (ddd, *J* = 13.8, 9.9,

6.9 Hz, 1H), 2.45 – 2.23 (m, 3H), 1.72 (dd, J = 13.6, 10.7 Hz, 1H), 1.42 (s, 3H), 1.32 (s, 3H).

^{13}C NMR (101 MHz, Chloroform-*d*) δ 168.91, 149.72, 141.45, 140.75, 140.62, 137.89, 133.92, 130.63, 128.67, 128.51, 126.11, 124.33, 123.19, 122.44, 92.02, 60.15, 57.46, 44.16, 33.00, 31.20, 30.50, 27.58, 27.09.

HRMS (ESI-TOF): m/z Calcd for $C_{25}H_{24}IN_3NaO^+$ [M+Na$^+$]: 532.0856; found: 532.0863.

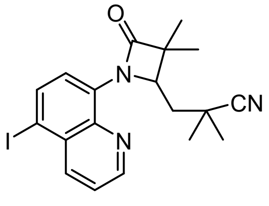

4ap

R$_f$ = 0.5, Petroleum Ether: Ethyl Acetate = 4: 1 (v/v)

Compound **4ap** was prepared following the general procedure in 86% yield (74 mg) as a yellow oil.

^{1}H NMR (400 MHz, Chloroform-*d*) δ 8.87 (dd, J = 4.2, 1.6 Hz, 1H), 8.36 (dd, J = 8.6, 1.7 Hz, 1H), 8.06 (d, J = 8.2 Hz, 1H), 7.99 (d, J = 8.2 Hz, 1H), 7.49 (dd, J = 8.6, 4.2 Hz, 1H), 5.18 (dd, J = 10.3, 1.4 Hz, 1H), 2.03 (dd, J = 14.4, 1.5 Hz, 1H), 1.91 (dd, J = 14.4, 10.3 Hz, 1H), 1.59 (s, 3H), 1.48 (s, 3H), 1.42 (s, 3H), 1.36 (s, 3H).

^{13}C NMR (101 MHz, Chloroform-*d*) δ 172.96, 149.98, 141.39, 140.63, 137.81, 133.56, 130.67, 124.93, 123.74, 123.19, 92.58, 66.16, 54.16, 39.10, 30.83, 27.95, 27.75, 22.89, 18.16.

HRMS (ESI-TOF): m/z Calcd for $C_{19}H_{20}IN_3NaO^+$ [M+Na$^+$]: 456.0543; found: 456.0541.

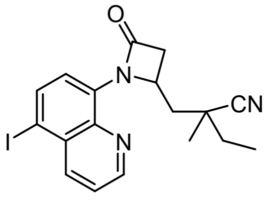

4as

R$_f$ = 0.4, Petroleum Ether: Ethyl Acetate = 4: 1 (v/v)

Compound **4as** was prepared following the general procedure in 31% yield (26 mg) as a yellow solid. m.p. 136-138 °C.

^{1}H NMR (400 MHz, Chloroform-*d*) δ 8.87 (ddd, *J* = 32.5, 4.1, 1.6 Hz, 1H), 8.37 (ddd, *J* = 8.6, 3.5, 1.6 Hz, 1H), 8.12 (d, *J* = 8.2 Hz, 1H), 8.06 (dd, *J* = 8.2, 1.9 Hz, 1H), 7.50 (dd, *J* = 8.6, 4.1 Hz, 1H), 5.48 (ddt, *J* = 13.0, 7.2, 2.2 Hz, 1H), 3.52 (dt, *J* = 15.2, 5.6 Hz, 1H), 3.04 (ddd, *J* = 30.1, 15.3, 2.6 Hz, 1H), 2.54 – 2.23 (m, 1H), 1.82 – 1.73 (m, 1H), 1.68 – 1.62 (m, 2H), 1.57 – 1.49 (m, 1H), 1.50 – 1.39 (m, 3H), 1.06 (dt, *J* = 10.6, 7.4 Hz, 3H).

^{13}C NMR (101 MHz, Chloroform-*d*) δ 165.98, 149.66, 140.82, 140.70, 137.98, 134.05, 130.67, 123.42, 123.23, 122.39, 92.10, 54.02, 45.51, 42.66, 35.68, 33.14, 24.04, 9.28.

HRMS (ESI-TOF): m/z Calcd for $C_{18}H_{19}IN_3O^+$ [M+H$^+$]: 420.0567; found: 420.0567.

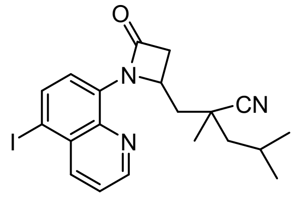

4at

R$_f$ = 0.5, Petroleum Ether: Ethyl Acetate = 4: 1 (v/v)

Compound **4at** was prepared following the general procedure in 29% yield (26 mg) as a yellow solid. m.p. 146-148 °C.

^{1}H NMR (400 MHz, Chloroform-*d*) δ 8.81 (dd, *J* = 4.1, 1.6 Hz, 1H), 8.38 (dd, *J* = 8.5, 1.6 Hz, 1H), 8.12 (d, *J* = 8.2 Hz, 1H), 8.06 (d, *J* = 8.2 Hz, 1H), 7.50 (dd, *J* = 8.6, 4.1 Hz, 1H), 5.46 (ddt, *J* = 10.7, 4.6, 2.0 Hz, 1H), 3.54 (dd, *J* = 15.5, 5.2 Hz, 1H), 3.09 (dd, *J* = 15.5, 2.5 Hz, 1H), 2.24 (dd, *J* = 13.5, 1.8 Hz, 1H), 1.91 – 1.76 (m, 2H), 1.50 (s, 3H), 1.47 – 1.40 (m, 2H), 1.02 (d, *J* = 6.7 Hz, 3H), 1.00 (d, *J* = 6.7 Hz, 3H).

^{13}C NMR (101 MHz, Chloroform-*d*) δ 166.05, 149.62, 140.80, 140.71, 137.99, 134.05, 130.67, 124.06, 123.22, 122.44, 92.10, 53.95, 48.78, 45.61, 43.76, 33.90, 25.26, 25.07, 24.23, 23.80.

HRMS (ESI-TOF): m/z Calcd for $C_{20}H_{22}IN_3NaO^+$ [M+Na$^+$]: 470.0700; found: 470.0704.

7. Gram-scale Reactions

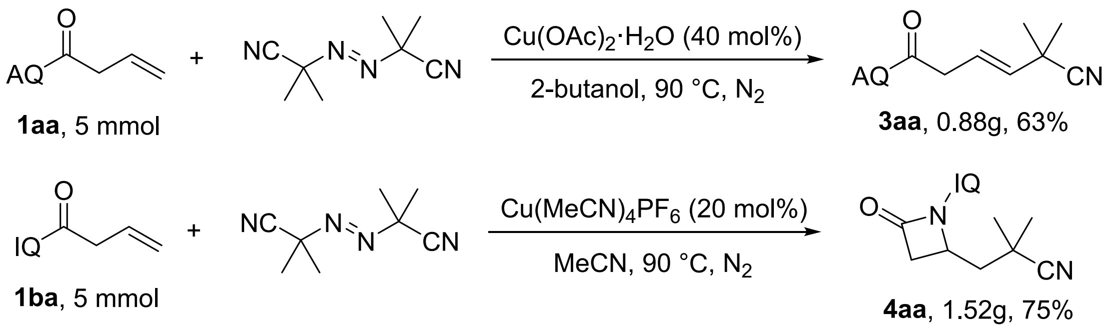

Scheme S15. Gram-scale reactions

a) The alkenes substrate **1aa** (1.06g, 5 mmol, 1 equiv), azo compounds **2a** (2.46g, 15 mmol, 3 equiv), $Cu(OAc)_2 \cdot H_2O$ (0.4g, 2 mmol, 0.4 equiv), 2-butanol (25 mL) were added into a 50 mL sealed glass vial under N_2 atmosphere. The reaction mixture was heated at 90 °C for 12 h. After being cooled to room temperature, the mixture was concentrated in vacuo and purified by silica gel (300 - 400 mesh) column chromatography to give the desired products 0.88g.

b) The alkenes substrate **1ba** (1.69g, 5 mmol, 1 equiv), azo compounds **2a** (3.28g, 20 mmol, 4 equiv), $Cu(MeCN)_4PF_6$ (0.38g, 1 mmol, 0.2 equiv), MeCN (50 mL) were added into a 100 mL sealed glass vial under N_2 atmosphere. The reaction mixture was heated at 90 °C for 12 h. After being cooled to room temperature, the mixture was concentrated in vacuo and purified by silica gel (300 - 400 mesh) column chromatography to give the desired products 1.52g.

8. Mechanistic Experiments

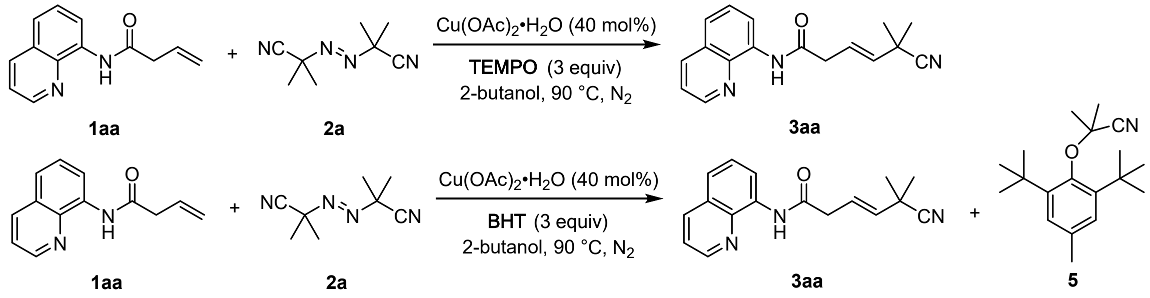

Scheme S16. Mechanistic Experiments of 1aa

a) The alkenes substrate **1aa** (0.0424g, 0.2 mmol, 1 equiv), azo compounds **2a** (0.0984g, 0.6 mmol, 3 equiv), $Cu(OAc)_2 \cdot H_2O$ (16mg, 0.08 mmol, 0.4 equiv), TEMPO (0.0938g, 0.6 mmol, 3 equiv), 2-butanol (1 mL) were added into a 15 mL sealed glass vial under N_2 atmosphere. The reaction mixture was heated at 90 °C for 12 h. After being cooled to room temperature, the mixture was concentrated *in vacuo* and purified by silica gel (300 - 400 mesh) column chromatography to give the desired products.

b) The alkenes substrate **1aa** (0.0424g, 0.2 mmol, 1 equiv), azo compounds **2a** (0.0984g, 0.6 mmol, 3 equiv), $Cu(OAc)_2 \cdot H_2O$ (16mg, 0.08 mmol, 0.4 equiv), BHT (0.1322g, 0.6 mmol, 3 equiv), 2-butanol (1 mL) were added into a 15 mL sealed glass vial under N_2 atmosphere. The reaction mixture was heated at 90 °C for 12 h. After being cooled to room temperature, the mixture was concentrated *in vacuo* and purified by silica gel (300 - 400 mesh) column chromatography to give the product **5**.

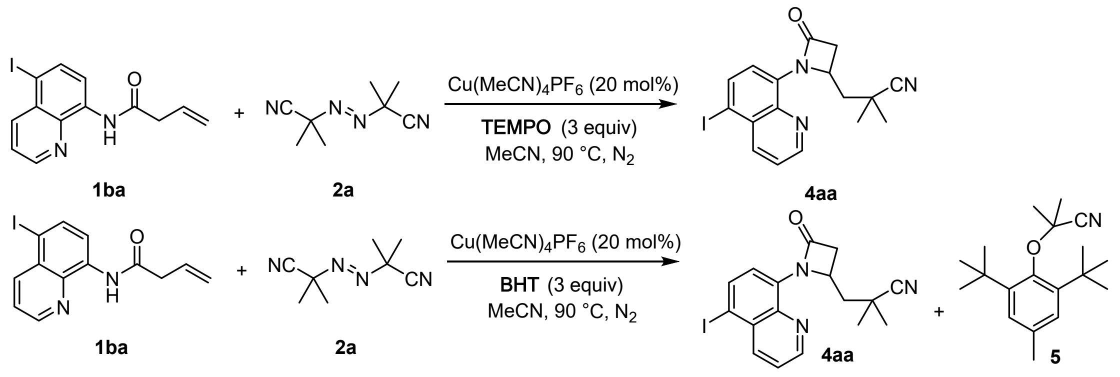

Scheme S17. Mechanistic Experiments of 1ba

a) The alkenes substrate **1ba** (0.0676g, 0.2 mmol, 1 equiv), azo compounds **2a** (0.1312g, 0.8 mmol, 4 equiv), $Cu(MeCN)_4PF_6$ (15mg, 0.04 mmol, 0.2 equiv), TEMPO (0.0938g, 0.6 mmol, 3 equiv), MeCN (2 mL) were added into a 15 mL sealed glass vial under N_2 atmosphere. The reaction mixture was heated at 90 °C for 12 h. After being cooled to room temperature, the mixture was concentrated *in vacuo* and purified by silica gel (300 - 400 mesh) column chromatography to give the desired products.

b) The alkenes substrate **1ba** (0.0676g, 0.2 mmol, 1 equiv), azo compounds **2a** (0.1312g, 0.8 mmol, 4 equiv), $Cu(MeCN)_4PF_6$ (15mg, 0.04 mmol, 0.2 equiv), BHT (0.1322g, 0.6 mmol, 3 equiv), MeCN (2 mL) were added into a 15 mL sealed glass vial under N_2 atmosphere. The reaction mixture was heated at 90 °C for 12 h. After being cooled to room temperature, the mixture was concentrated *in vacuo* and purified by silica gel (300 - 400 mesh) column chromatography to give the product **5**.

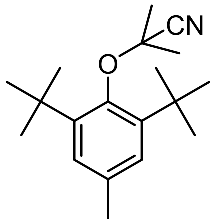

5

R_f = 0.5, Petroleum Ether: Ethyl Acetate = 10: 1 (v/v)

Compound **5** was prepared following the general procedure as a white solid. m.p. 106-108 °C.

^{1}H NMR (400 MHz, Chloroform-*d*) δ 6.52 (s, 2H), 1.40 (s, 3H), 1.28 (s, 6H), 1.23 (s, 18H).

^{13}C NMR (101 MHz, Chloroform-*d*) δ 185.67, 149.41, 140.81, 123.57, 42.81, 39.88, 35.24, 29.55, 23.00, 22.70.

HRMS (ESI-TOF): m/z Calcd for $C_{19}H_{30}NO^+$ $[M+H^+]$: 288.2322; found: 288.2317.

9. X-Ray Crystallography

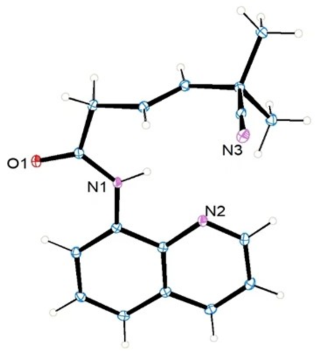

X-ray structure of compound 3aa

mo_b230717b_0m

Table S3 Crystal data and structure refinement for mo_b230717b_0m.

Identification code	mo_b230717b_0m
Empirical formula	$C_{17}H_{17}N_3O$
Formula weight	279.33
Temperature/K	100.0
Crystal system	triclinic
Space group	P-1
a/Å	8.6338(15)
b/Å	8.7425(17)
c/Å	10.074(2)
α/°	90.512(7)
β/°	105.948(6)
γ/°	95.537(6)
Volume/Å^3	727.2(2)
Z	2
ρ_{calc}g/cm^3	1.276
μ/mm^{-1}	0.082
F(000)	296.0
Crystal size/mm^3	0.2 × 0.1 × 0.05
Radiation	MoKα (λ = 0.71073)
2Θ range for data collection/°	5.528 to 55.054
Index ranges	$-11 \leq h \leq 11$, $-11 \leq k \leq 11$, $-13 \leq l \leq 13$
Reflections collected	15069
Independent reflections	3298 [R_{int} = 0.0703, R_{sigma} = 0.0512]
Data/restraints/parameters	3298/0/192

Goodness-of-fit on F^2	1.086
Final R indexes [I>=2σ (I)]	R_1 = 0.0591, wR_2 = 0.1348
Final R indexes [all data]	R_1 = 0.0720, wR_2 = 0.1445
Largest diff. peak/hole / e Å^{-3}	0.33/-0.31

Table S4 Fractional Atomic Coordinates (×10^4) and Equivalent Isotropic Displacement Parameters (Å^2×10^3) for mo_b230717b_0m. U_{eq} is defined as 1/3 of of the trace of the orthogonalised U_{IJ} tensor.

**Atom**	***x***	***y***	***z***	**U(eq)**
O001	249.0(15)	1542.1(13)	6793.6(13)	24.6(3)
N002	2781.8(16)	5959.7(16)	4929.3(14)	20.0(3)
N003	1521.9(17)	3743.9(16)	6223.4(14)	19.5(3)
N004	5412.8(18)	7477.8(17)	10331.1(16)	25.3(3)
C005	4142(2)	7728.1(19)	9716.7(17)	19.6(4)
C006	2514.9(19)	8071.4(19)	8892.4(17)	19.5(4)
C007	1941.9(19)	3278.8(19)	5045.3(17)	18.6(3)
C008	3034(2)	2597(2)	2736.7(18)	22.7(4)
C009	2325(2)	1468(2)	3371.6(18)	22.1(4)
C00A	1393(2)	6593.2(19)	8533.1(17)	20.4(4)
C00B	780.1(19)	2890.9(19)	7038.0(17)	19.0(3)
C00C	615(2)	3759.3(19)	8302.5(18)	20.4(4)
C00D	1783.2(19)	5180.0(19)	8762.0(17)	18.8(3)
C00E	3951(2)	5385(2)	2657.0(18)	22.5(4)
C00F	2659.7(18)	4495.6(19)	4392.4(17)	18.8(4)
C00G	3454(2)	7075(2)	4336.3(18)	22.1(4)
C00H	2704(2)	8816(2)	7556.0(18)	23.9(4)
C00I	1780.1(19)	1792.9(19)	4533.1(17)	20.5(4)
C00J	4069(2)	6842(2)	3199.1(18)	23.4(4)
C00K	1843(2)	9180(2)	9736(2)	26.4(4)
C00L	3219.9(19)	4145(2)	3237.6(17)	20.6(4)

Table S5 Anisotropic Displacement Parameters (Å^2×10^3) for mo_b230717b_0m. The Anisotropic displacement factor exponent takes the form: $-2\pi^2[h^2a^{*2}U_{11}+2hka^*b^*U_{12}+\ldots]$.

Atom	U_{11}	U_{22}	U_{33}	U_{23}	U_{13}	U_{12}
O001	27.7(6)	16.4(6)	31.1(7)	-0.1(5)	11.6(5)	-1.4(5)
N002	19.3(7)	18.1(7)	21.4(7)	0.8(6)	3.8(5)	0.7(5)
N003	23.4(7)	14.0(7)	21.4(7)	-0.8(5)	7.8(6)	-1.3(5)
N004	22.5(7)	26.0(8)	26.9(8)	3.3(6)	6.8(6)	-0.4(6)
C005	22.8(8)	17.9(8)	19.5(8)	-1.2(6)	10.0(7)	-3.4(6)
C006	19.3(8)	17.4(8)	21.7(8)	-0.4(6)	5.7(6)	1.3(6)
C007	15.1(7)	21.0(8)	18.2(8)	2.0(6)	2.3(6)	1.4(6)
C008	19.9(8)	26.8(9)	21.5(8)	-3.1(7)	5.6(6)	4.3(7)
C009	20.2(8)	19.7(8)	24.7(9)	-3.6(7)	3.4(7)	2.6(6)
C00A	17.0(8)	21.9(9)	22.0(8)	-1.0(7)	5.6(6)	0.8(6)
C00B	15.1(7)	17.2(8)	23.2(8)	2.0(6)	2.8(6)	2.0(6)
C00C	20.2(8)	18.9(8)	22.6(8)	0.7(6)	7.6(6)	-0.6(6)
C00D	17.6(7)	20.6(8)	17.8(8)	0.3(6)	4.6(6)	0.6(6)
C00E	18.0(8)	30.9(10)	18.7(8)	2.7(7)	5.1(6)	2.6(7)
C00F	14.6(7)	20.9(8)	18.5(8)	1.4(6)	0.7(6)	1.6(6)
C00G	21.7(8)	18.6(8)	23.1(9)	2.2(7)	2.0(7)	-0.8(6)
C00H	27.0(9)	20.4(9)	24.5(9)	2.1(7)	7.5(7)	2.2(7)
C00I	19.2(8)	18.5(8)	22.2(8)	0.6(6)	3.6(6)	0.3(6)
C00J	20.1(8)	26.4(9)	22.0(9)	7.3(7)	3.7(7)	-0.5(7)
C00K	26.4(9)	20.6(9)	34.7(10)	-5.3(7)	13.6(8)	0.5(7)
C00L	15.7(7)	25.6(9)	18.9(8)	1.3(7)	2.0(6)	2.1(6)

Table S6 Bond Lengths for mo_b230717b_0m.

Atom	Atom	Length/Å	Atom	Atom	Length/Å
O001	C00B	1.221(2)	C007	C00I	1.374(2)
N002	C00F	1.368(2)	C008	C009	1.366(3)
N002	C00G	1.317(2)	C008	C00L	1.419(2)
N003	C007	1.400(2)	C009	C00I	1.412(2)
N003	C00B	1.359(2)	C00A	C00D	1.317(2)
N004	C005	1.145(2)	C00B	C00C	1.524(2)
C005	C006	1.482(2)	C00C	C00D	1.505(2)
C006	C00A	1.515(2)	C00E	C00J	1.365(3)
C006	C00H	1.543(2)	C00E	C00L	1.414(2)
C006	C00K	1.538(2)	C00F	C00L	1.419(2)
C007	C00F	1.434(2)	C00G	C00J	1.410(3)

Table S7 Bond Angles for mo_b230717b_0m.

Atom	Atom	Atom	Angle/°	Atom	Atom	Atom	Angle/°
C00G	N002	C00F	117.69(15)	O001	C00B	N003	124.19(16)
C00B	N003	C007	128.98(14)	O001	C00B	C00C	121.08(15)
N004	C005	C006	178.46(18)	N003	C00B	C00C	114.72(14)
C005	C006	C00A	109.68(14)	C00D	C00C	C00B	115.28(14)
C005	C006	C00H	107.65(13)	C00A	C00D	C00C	124.13(15)
C005	C006	C00K	109.18(14)	C00J	C00E	C00L	119.86(16)
C00A	C006	C00H	109.67(14)	N002	C00F	C007	117.73(15)
C00A	C006	C00K	110.06(14)	N002	C00F	C00L	122.77(15)
C00K	C006	C00H	110.56(14)	C00L	C00F	C007	119.49(15)
N003	C007	C00F	114.60(14)	N002	C00G	C00J	123.84(16)
C00I	C007	N003	125.61(15)	C007	C00I	C009	119.98(16)
C00I	C007	C00F	119.73(15)	C00E	C00J	C00G	118.83(16)
C009	C008	C00L	119.77(16)	C008	C00L	C00F	119.23(16)
C008	C009	C00I	121.78(16)	C00E	C00L	C008	123.78(16)
C00D	C00A	C006	127.21(15)	C00E	C00L	C00F	116.99(16)

Table S8 Torsion Angles for mo_b230717b_0m.

A	B	C	D	Angle/°	A	B	C	D	Angle/°
O001	C00B	C00C	C00D	159.44(15)	C009	C008	C00L	C00F	-0.2(2)
N002	C00F	C00L	C008	-179.41(14)	C00B	N003	C007	C00F	177.56(15)
N002	C00F	C00L	C00E	1.0(2)	C00B	N003	C007	C00I	-5.2(3)
N002	C00G	C00J	C00E	0.9(3)	C00B	C00C	C00D	C00A	103.44(19)
N003	C007	C00F	N002	-3.2(2)	C00F	N002	C00G	C00J	-0.9(2)
N003	C007	C00F	C00L	176.34(13)	C00F	C007	C00I	C009	0.2(2)
N003	C007	C00I	C009	-176.88(15)	C00G	N002	C00F	C007	179.44(14)
N003	C00B	C00C	C00D	-21.8(2)	C00G	N002	C00F	C00L	-0.1(2)
C005	C006	C00A	C00D	-6.9(2)	C00H	C006	C00A	C00D	111.15(19)
C006	C00A	C00D	C00C	-176.42(15)	C00I	C007	C00F	N002	179.37(14)
C007	N003	C00B	O001	-5.3(3)	C00I	C007	C00F	C00L	-1.1(2)
C007	N003	C00B	C00C	175.95(15)	C00J	C00E	C00L	C008	179.48(15)
C007	C00F	C00L	C008	1.1(2)	C00J	C00E	C00L	C00F	-0.9(2)
C007	C00F	C00L	C00E	-178.53(14)	C00K	C006	C00A	C00D	-127.01(19)
C008	C009	C00I	C007	0.7(2)	C00L	C008	C009	C00I	-0.7(3)
C009	C008	C00L	C00E	179.37(16)	C00L	C00E	C00J	C00G	0.1(2)

Table S9 Hydrogen Atom Coordinates (Å×10^4) and Isotropic Displacement Parameters (Å^2×10^3) for mo_b230717b_0m.

**Atom**	***x***	***y***	***z***	**U(eq)**
H003	1773.14	4724.43	6470.38	23
H008	3400.23	2346.17	1962.57	27
H009	2195	436.98	3021.93	26
H00A	286.23	6697.15	8094.27	24
H00B	-501.31	4058.11	8099.85	24
H00C	763.55	3051.33	9078.95	24
H00D	2870.21	5049.38	9247.49	23
H00E	4359.78	5204.87	1891.18	27
H00G	3527.78	8095.59	4696.27	27
H00F	3411.28	9783.14	7790.92	36
H00H	1639.14	9024.03	6973.92	36
H00I	3183.03	8115.15	7054.09	36
H00J	1301.88	984.08	4960.36	25
H00K	4556.55	7684.43	2816.68	28
H00L	1754.27	8698.8	10590.66	40
H00M	770.79	9417.25	9192.38	40
H00N	2575.85	10132.15	9963.74	40

mo_b230717b_0m

Table S10 Crystal data and structure refinement for mo_b230717b_0m.

Identification code	mo_b230717b_0m
Empirical formula	$C_{17}H_{17}N_3O$
Formula weight	279.33
Temperature/K	100.0
Crystal system	triclinic
Space group	P-1
a/Å	8.6338(15)
b/Å	8.7425(17)
c/Å	10.074(2)
α/°	90.512(7)
β/°	105.948(6)
γ/°	95.537(6)
Volume/Å^3	727.2(2)
Z	2
ρ_{calc}g/cm^3	1.276
μ/mm^{-1}	0.082
F(000)	296.0
Crystal size/mm^3	0.2 × 0.1 × 0.05

Radiation	MoKα (λ = 0.71073)
2Θ range for data collection/°	5.528 to 55.054
Index ranges	-11 ≤ h ≤ 11, -11 ≤ k ≤ 11, -13 ≤ l ≤ 13
Reflections collected	15069
Independent reflections	3298 [R_{int} = 0.0703, R_{sigma} = 0.0512]
Data/restraints/parameters	3298/0/192
Goodness-of-fit on F^2	1.086
Final R indexes [I>=2σ (I)]	R_1 = 0.0591, wR_2 = 0.1348
Final R indexes [all data]	R_1 = 0.0720, wR_2 = 0.1445
Largest diff. peak/hole / e Å^{-3}	0.33/-0.31

10. References

[20] P. Shi, J. Wang, Z. Gan, J. Zhang, R. Zeng, Y. Zhao, *Chem. Commun.* **2019**, *55*, 10523–10526.
[21] H. Zhang, X. Lv, H. Yu, Z. Bai, G. Chen, G. He, *Org. Lett.* **2021**, *23*, 3620–3625.
[22] C.-B. Huang, L.-J. Chen, J. Huang, L. Xu, *RSC Adv.* **2014**, *4*, 19538.
[23] M. L. Czyz, D. W. Lupton, A. Polyzos, *Chemistry A European J* **2017**, *23*, 14450–14453.
[24] W. Su, J. Cui, R. Zeng, *ChemistryOpen* **2023**, *12*, e202300068.

11. NMR Spectra

^{1}H NMR of 1aa ($CDCl_3$, 400 MHz)

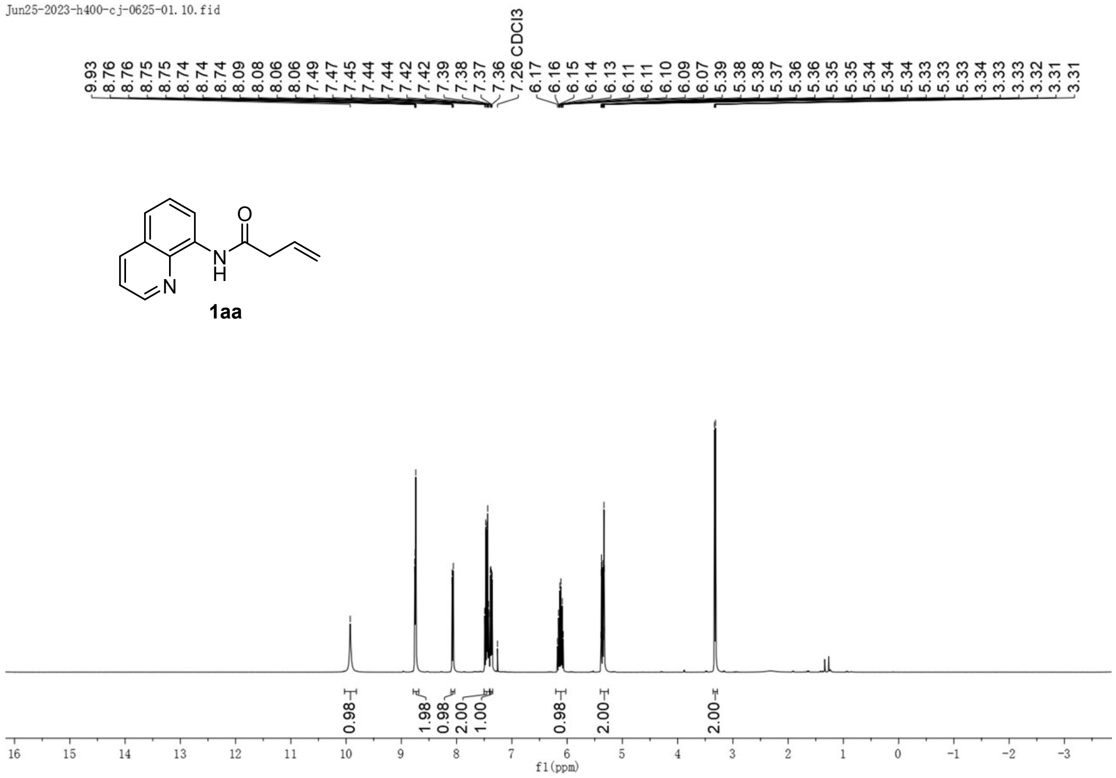

^{13}C NMR of 1aa ($CDCl_3$, 101 MHz)

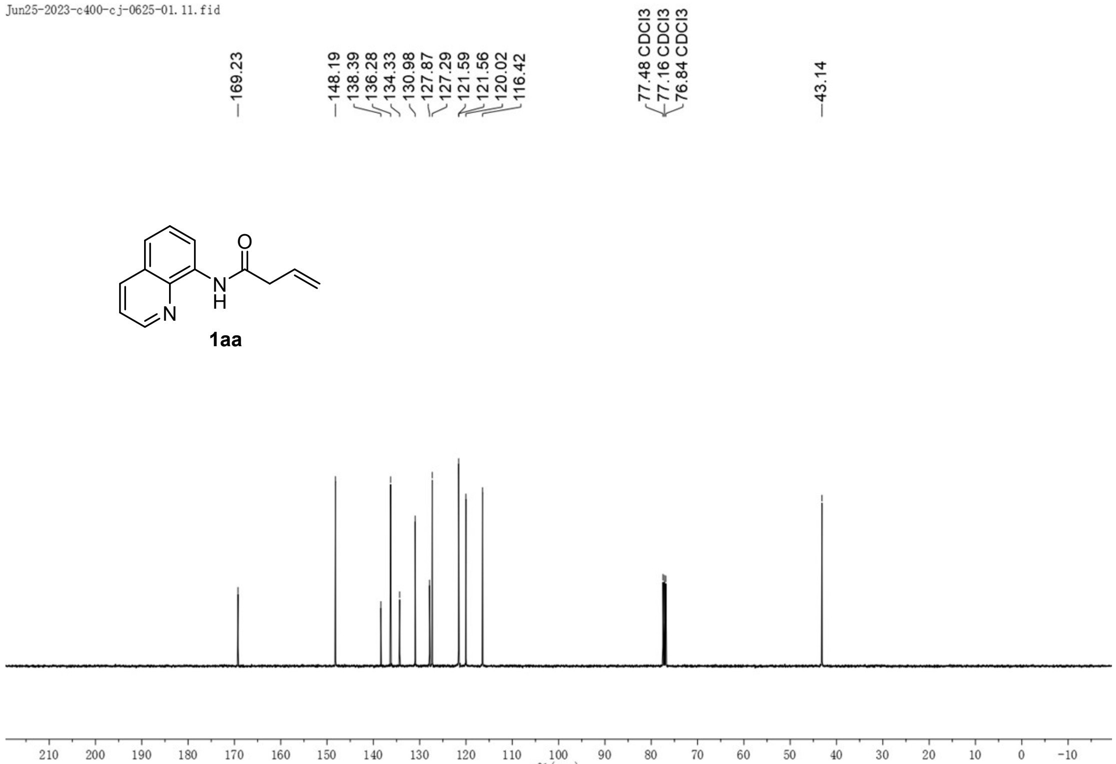

^{1}H NMR of 1ba ($CDCl_3$, 400 MHz)

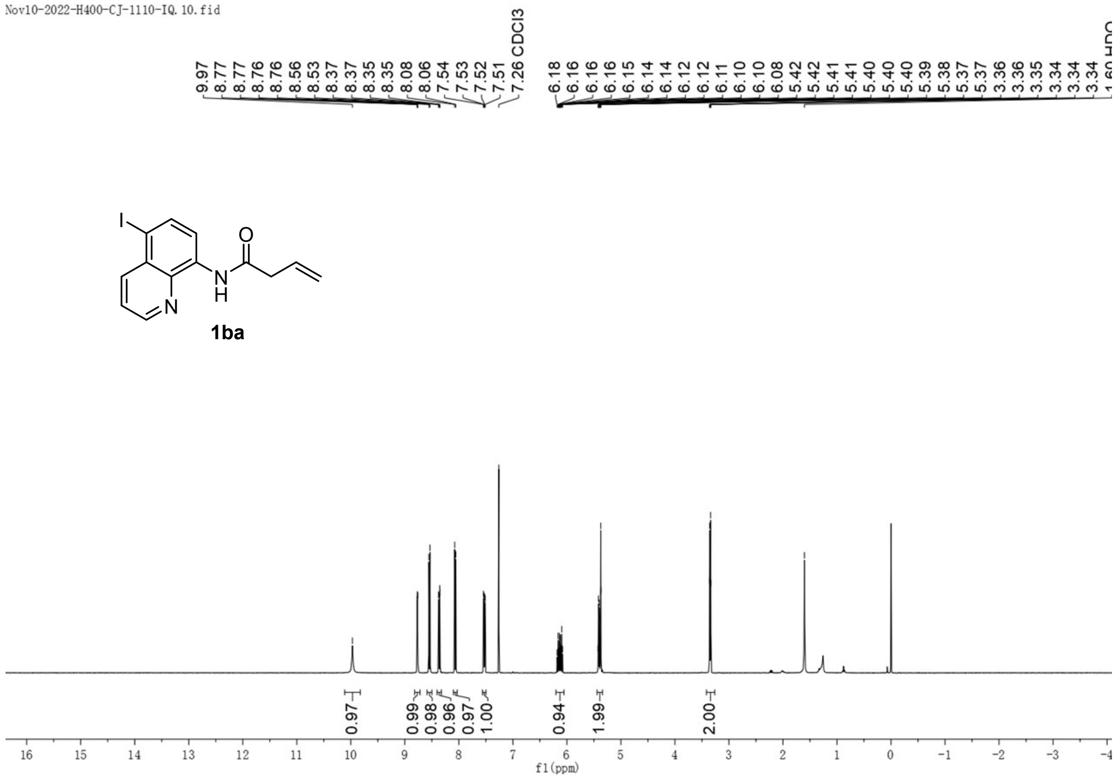

^{13}C NMR of 1ba ($CDCl_3$, 101 MHz)

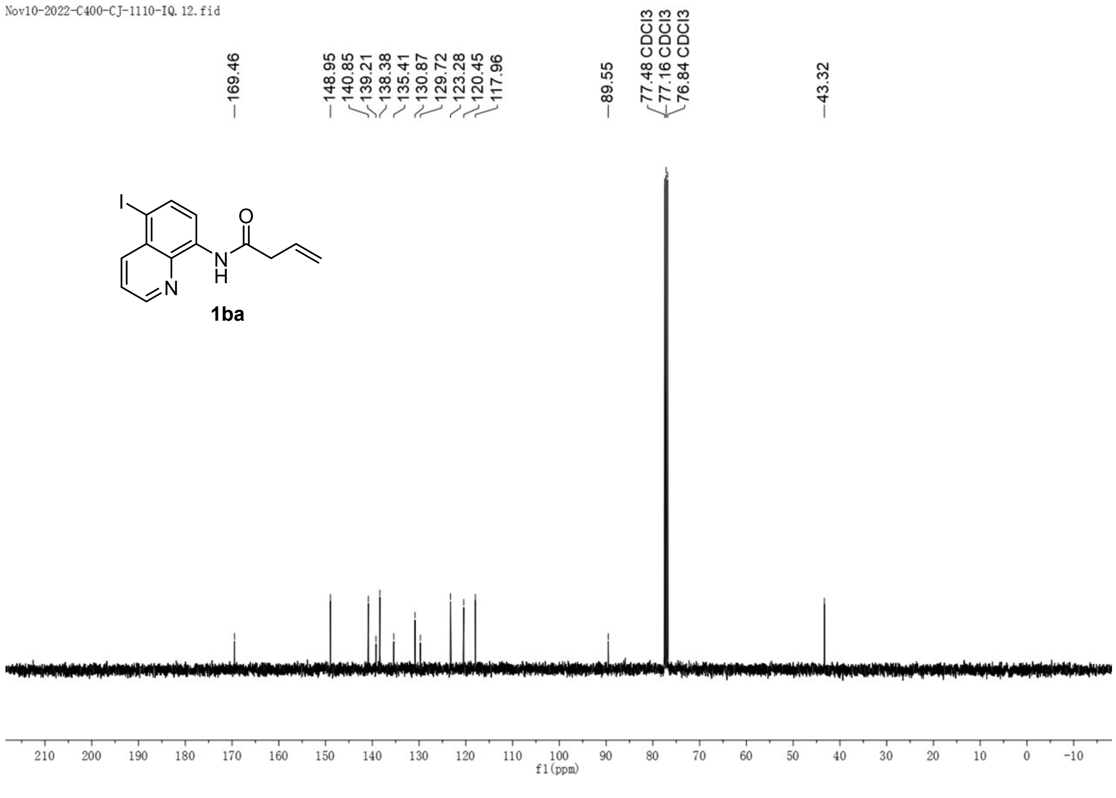

^{1}H NMR of 1c ($CDCl_3$, 400 MHz)

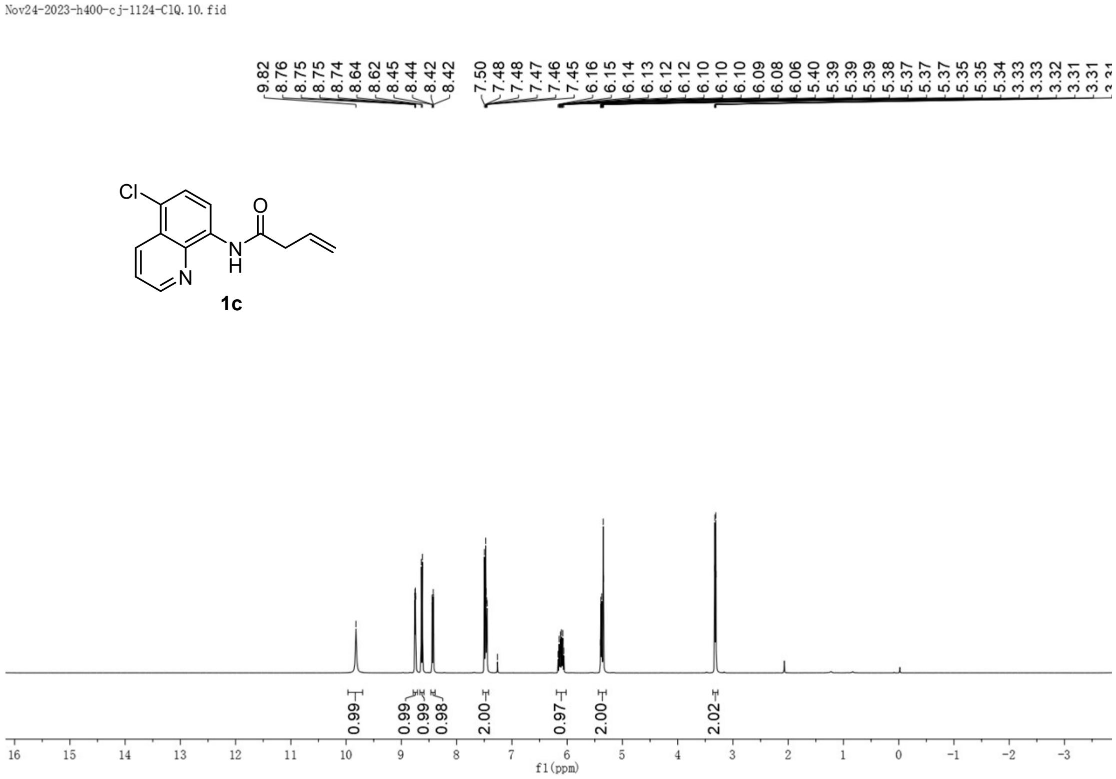

^{13}C NMR of 1c ($CDCl_3$, 101 MHz)

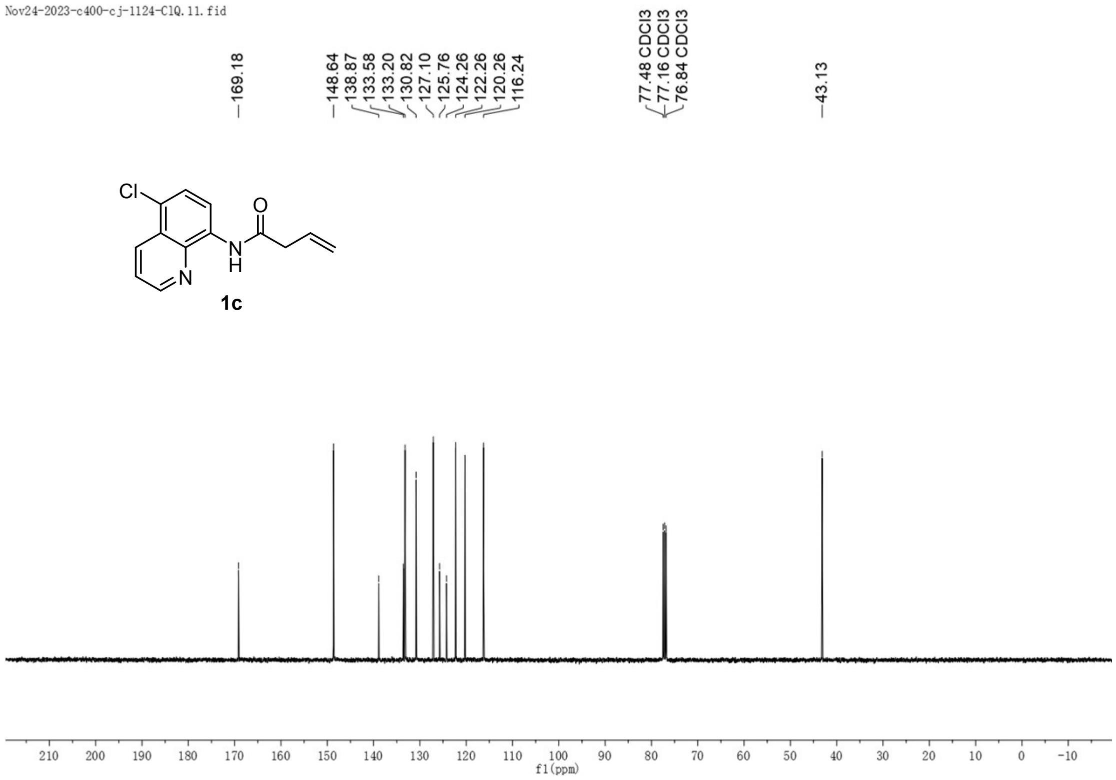

^{1}H NMR of 1d ($CDCl_3$, 400 MHz)

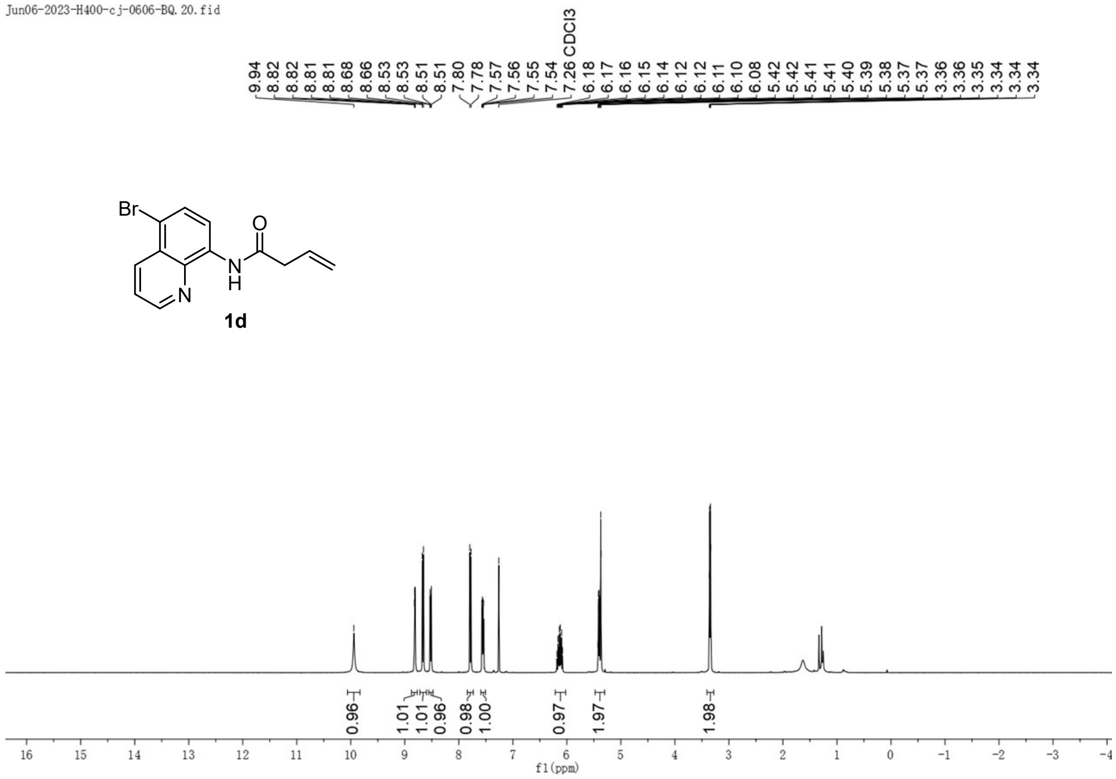

^{13}C NMR of 1d ($CDCl_3$, 101 MHz)

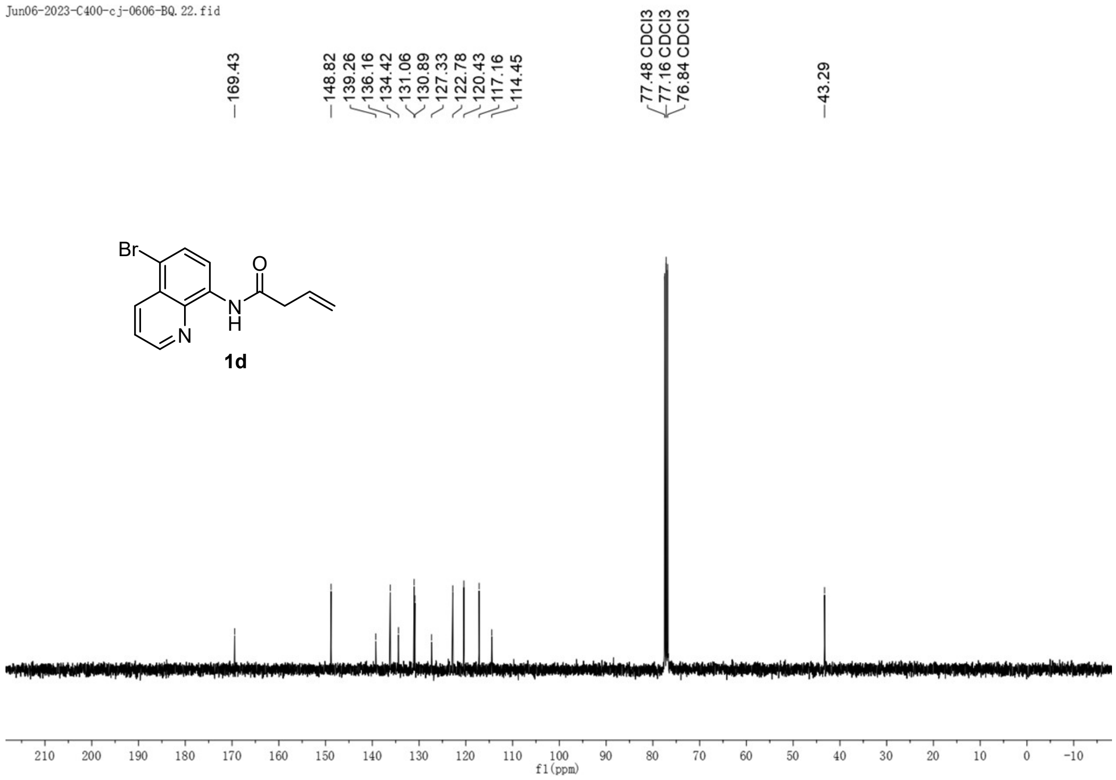

^{1}H NMR of 1ab ($CDCl_3$, 400 MHz)

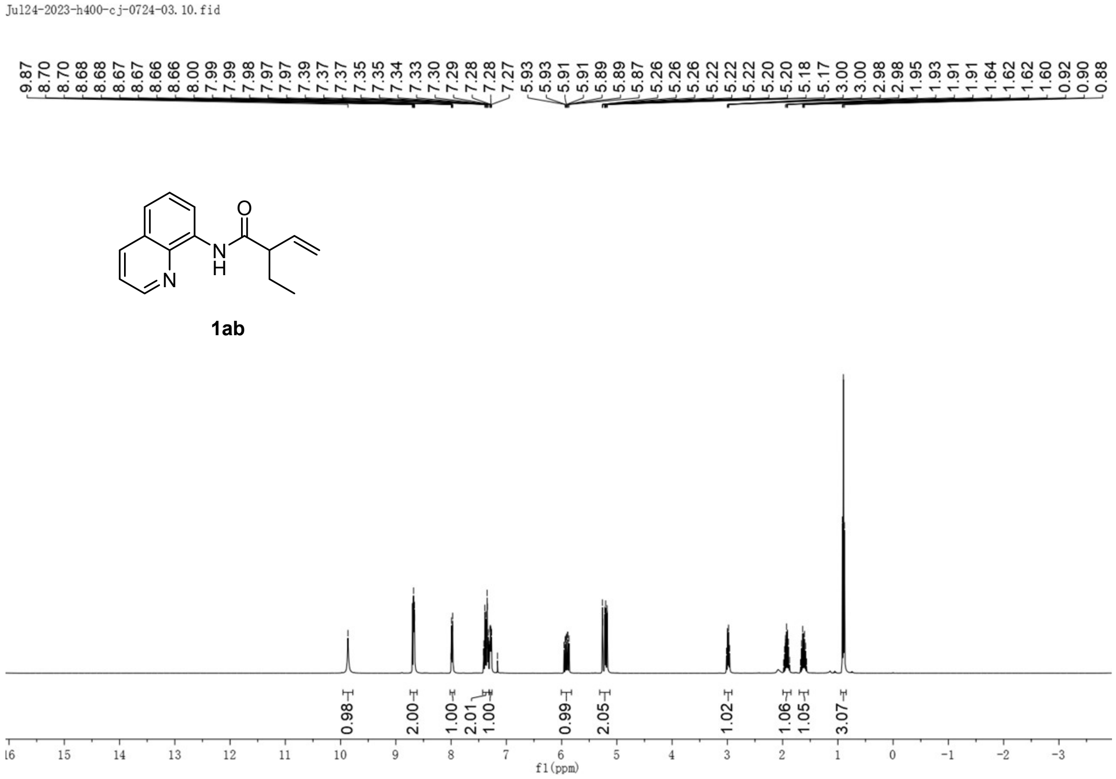

^{13}C NMR of 1ab ($CDCl_3$, 101 MHz)

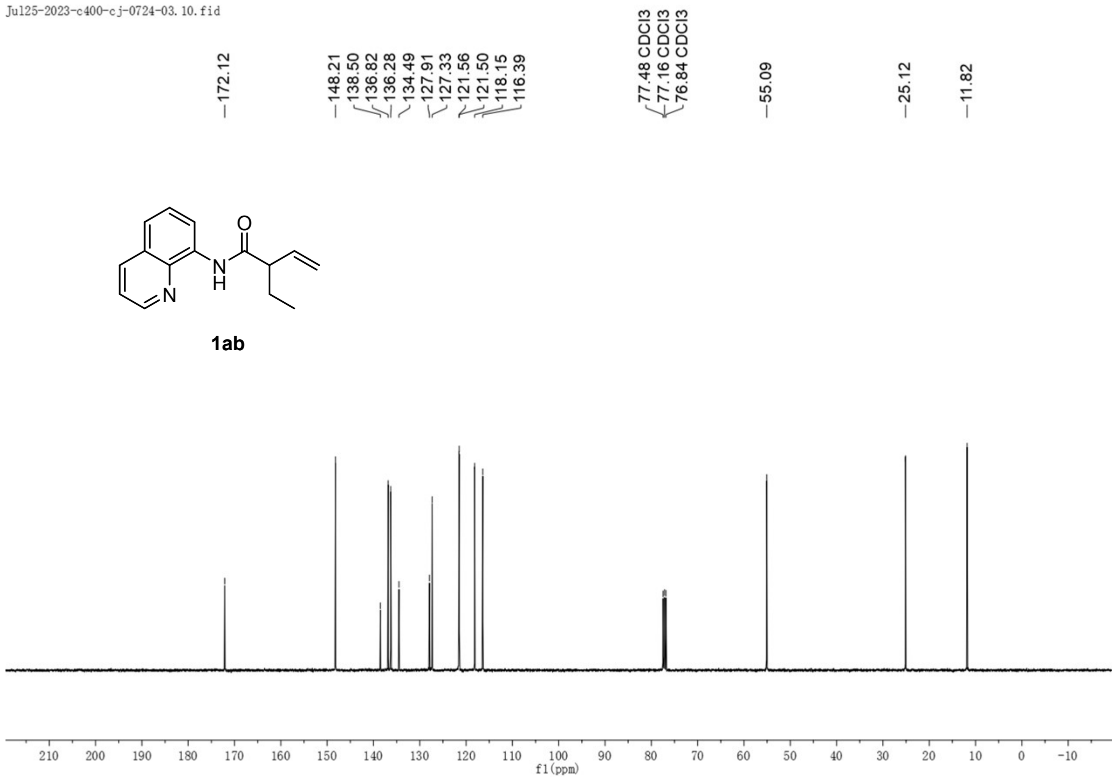

^{1}H NMR of 1ac ($CDCl_3$, 400 MHz)

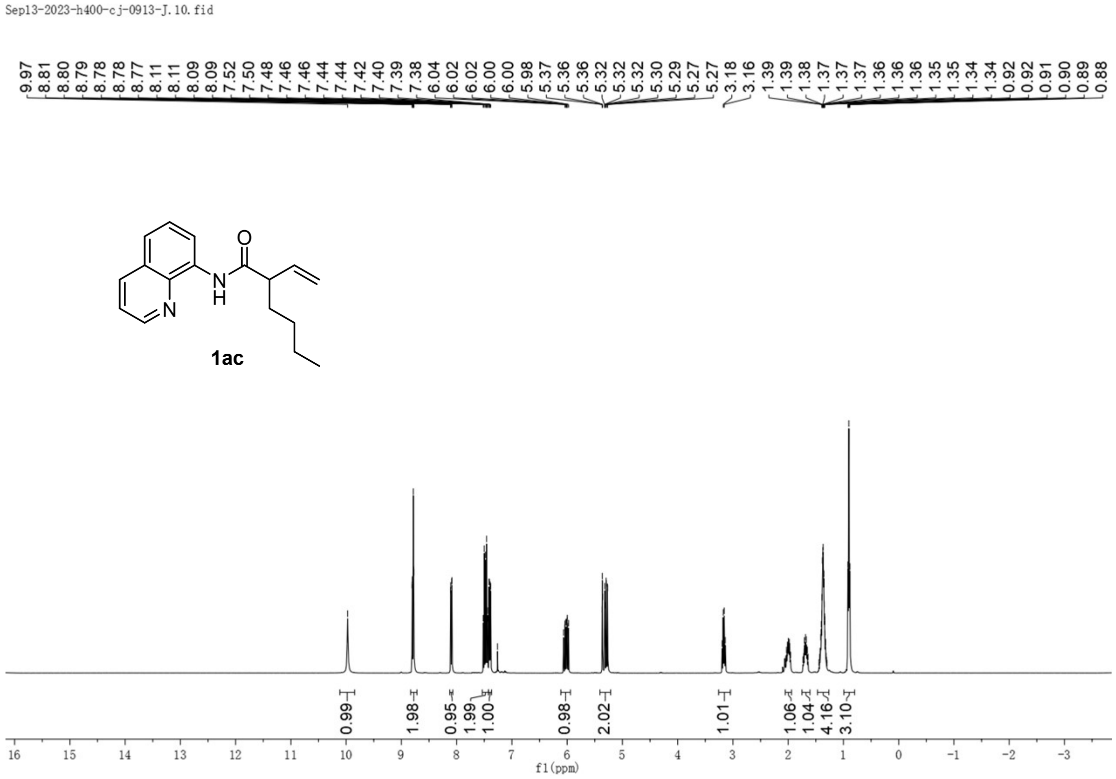

^{13}C NMR of 1ac ($CDCl_3$, 101 MHz)

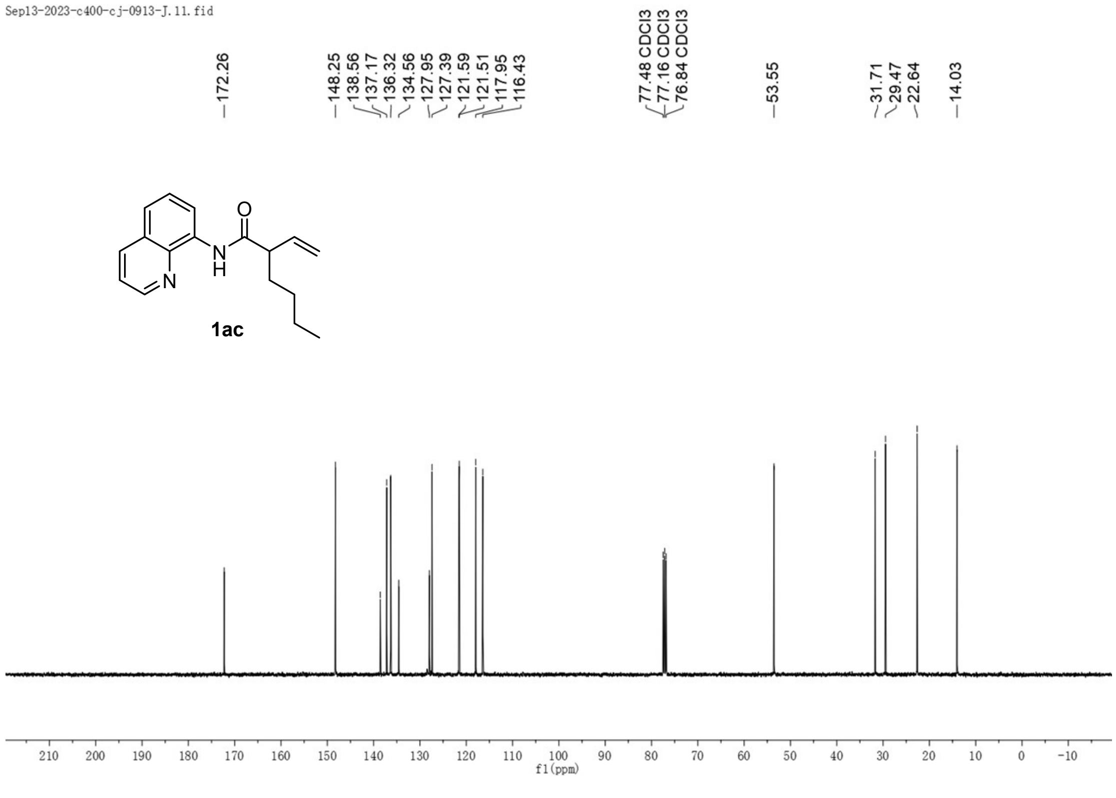

^{1}H NMR of 1ad ($CDCl_3$, 400 MHz)

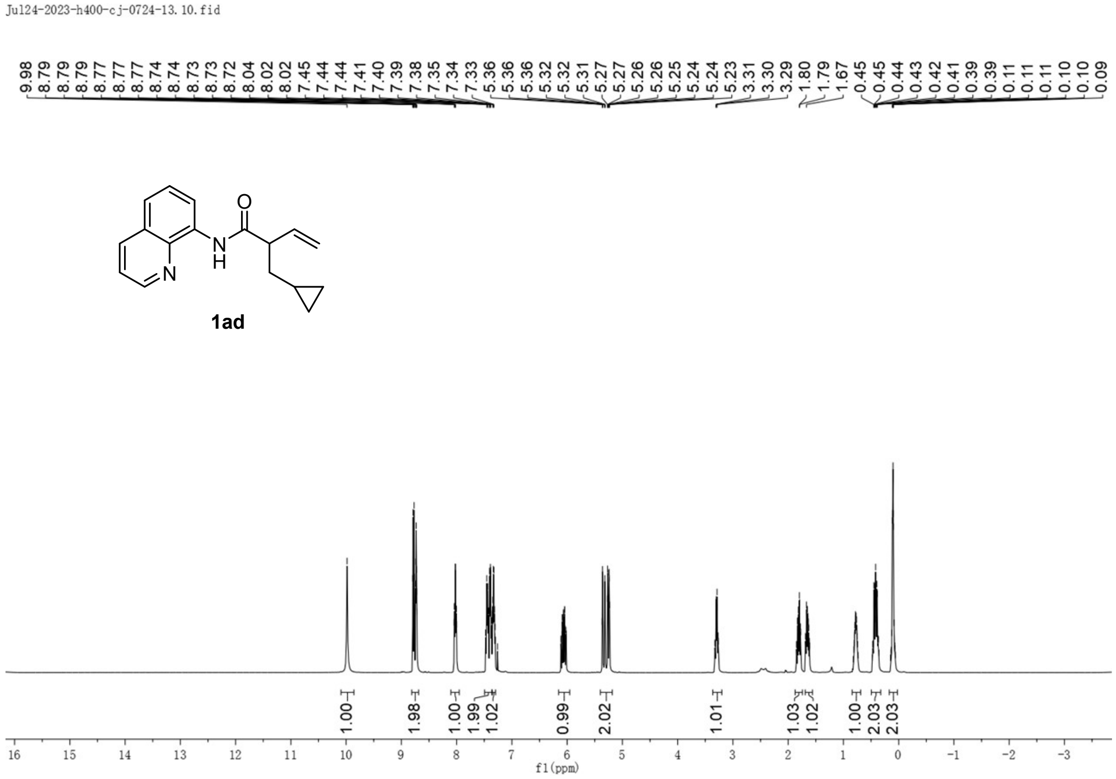

^{13}C NMR of 1ad ($CDCl_3$, 101 MHz)

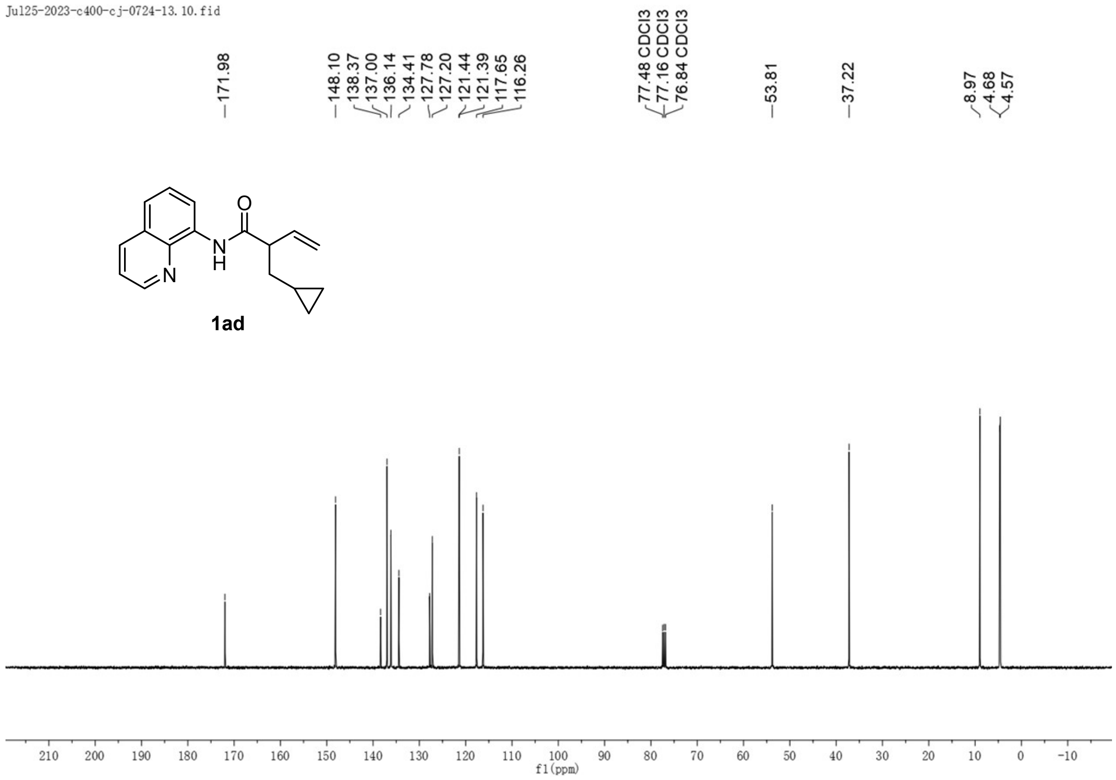

^{1}H NMR of 1ae ($CDCl_3$, 400 MHz)

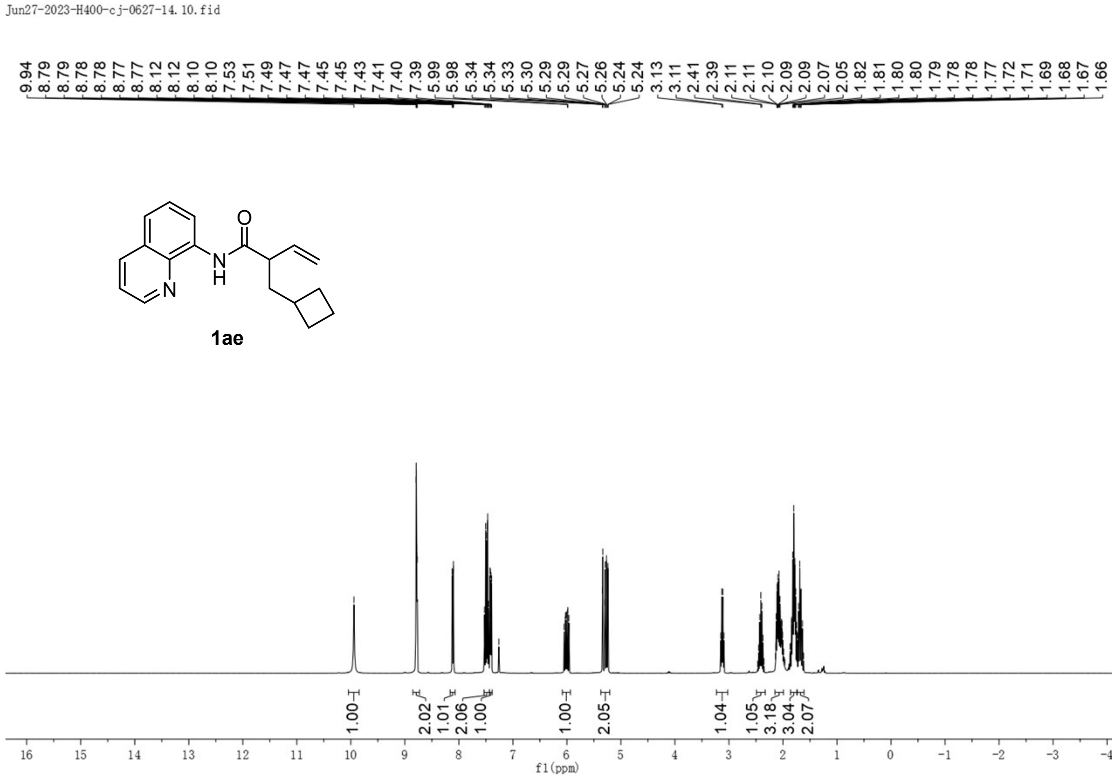

^{13}C NMR of 1ae ($CDCl_3$, 101 MHz)

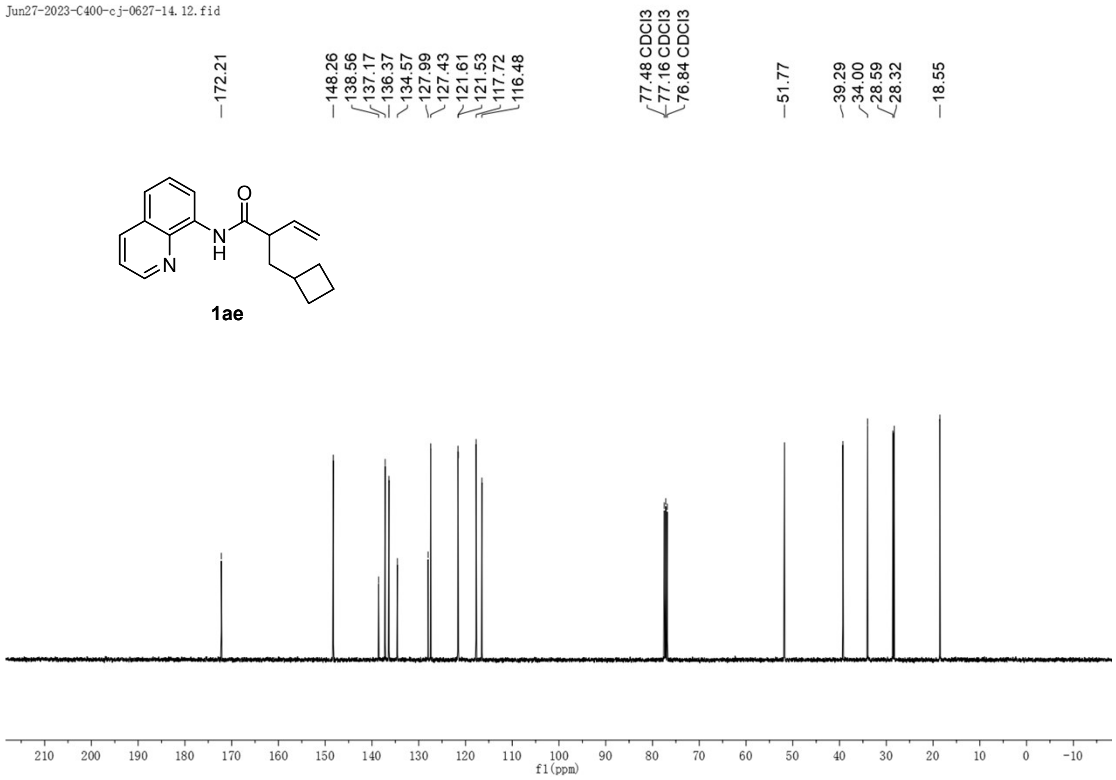

^{1}H NMR of 1af ($CDCl_3$, 400 MHz)

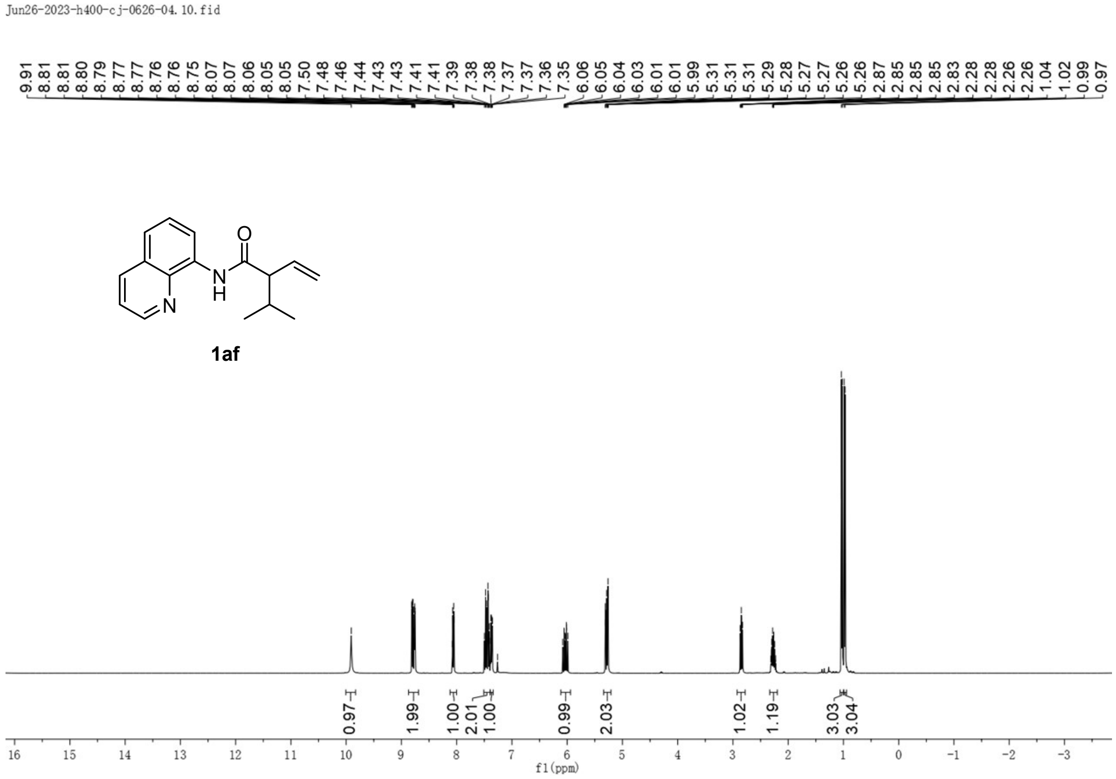

^{13}C NMR of 1af ($CDCl_3$, 101 MHz)

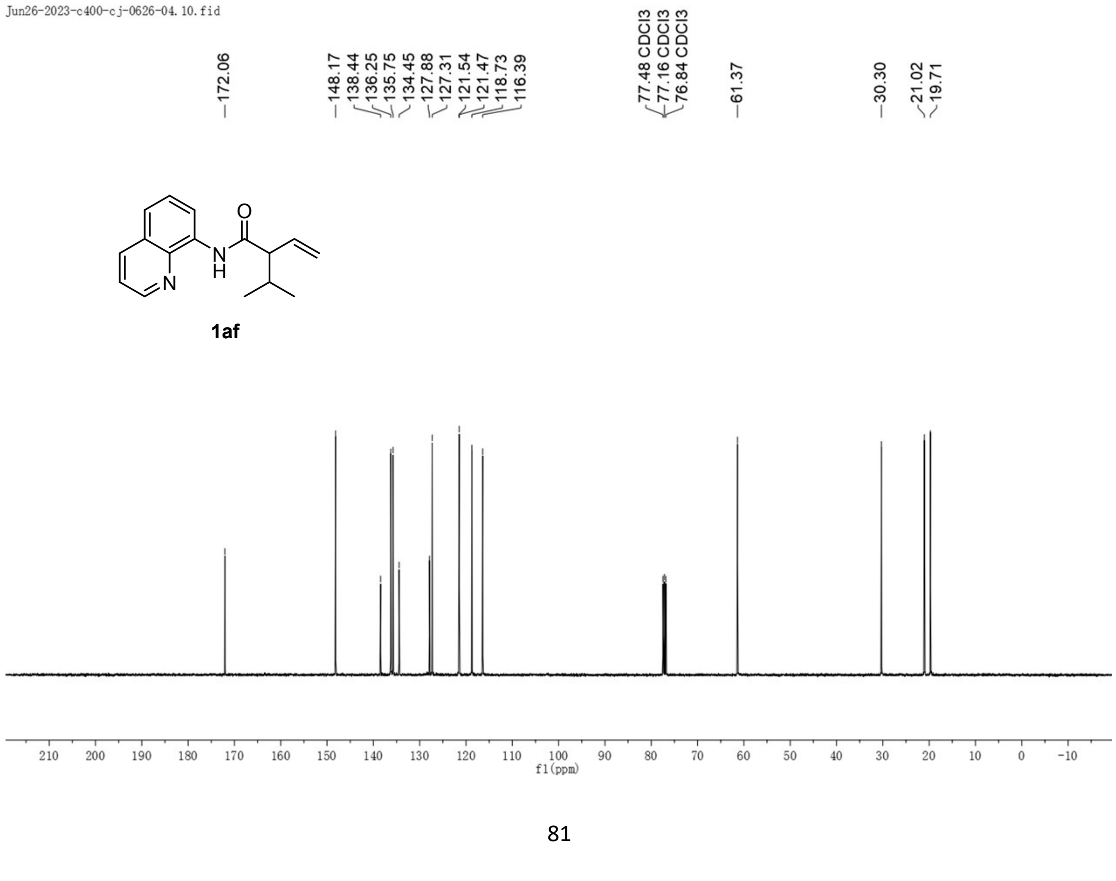

^{1}H NMR of 1ag ($CDCl_3$, 400 MHz)

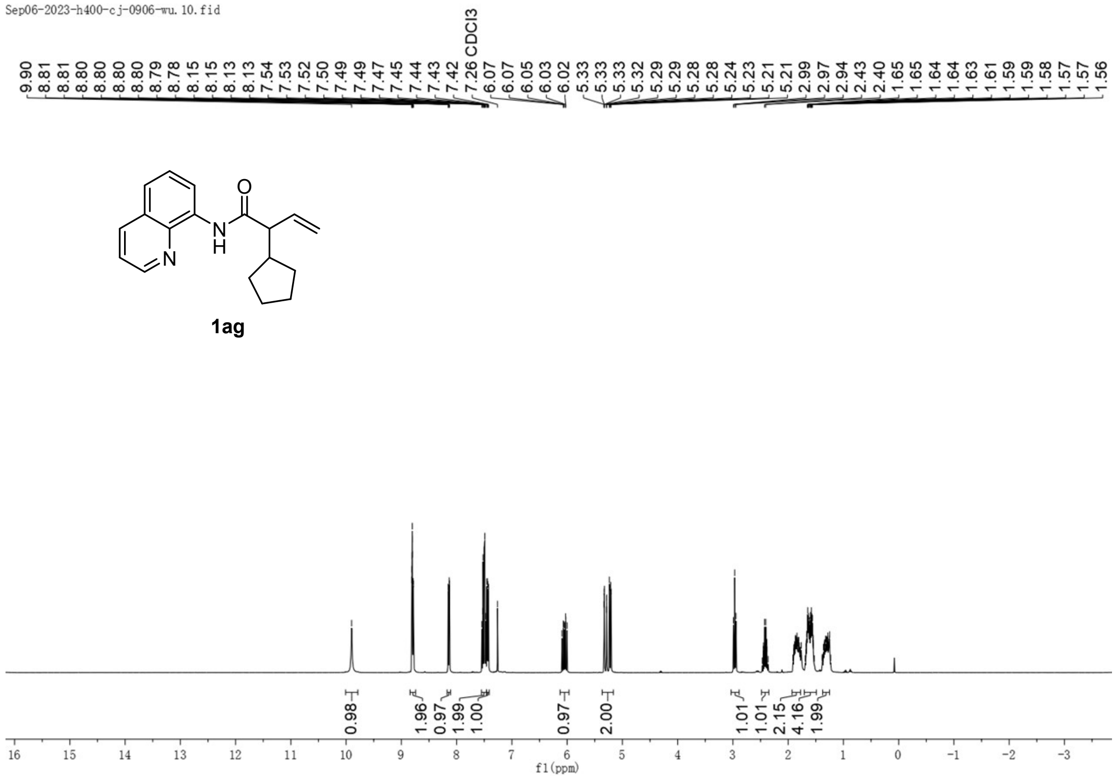

^{13}C NMR of 1ag ($CDCl_3$, 101 MHz)

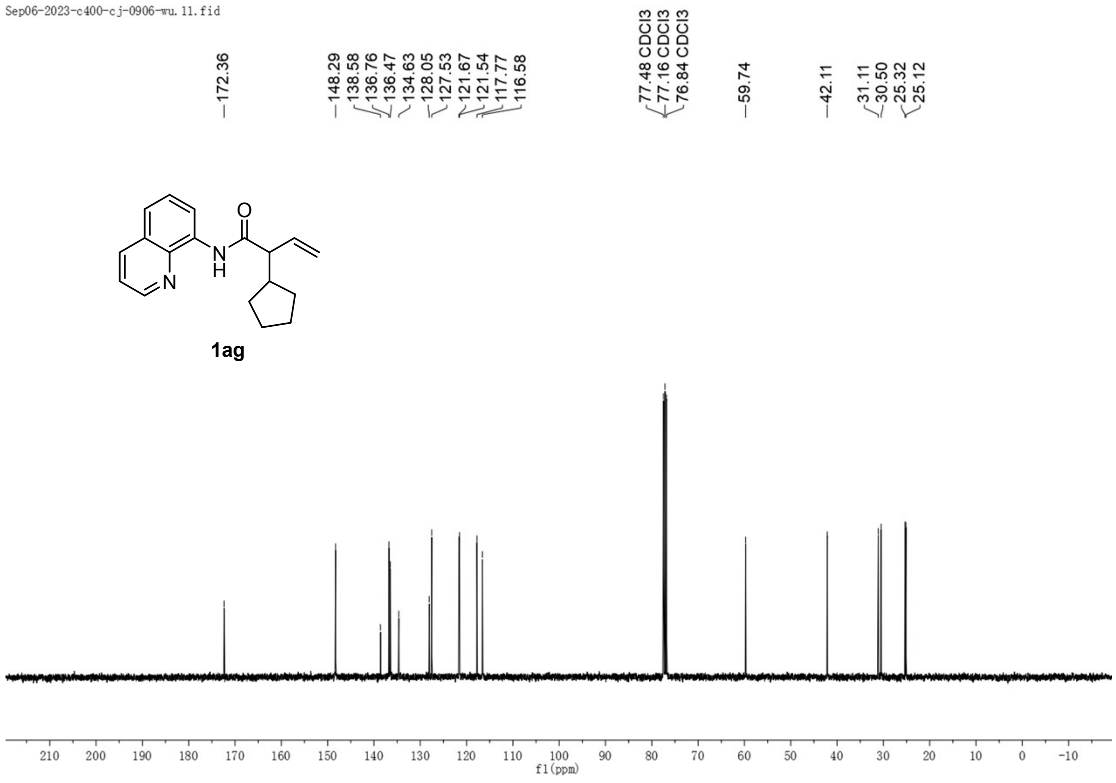

^{1}H NMR of 1ah ($CDCl_3$, 400 MHz)

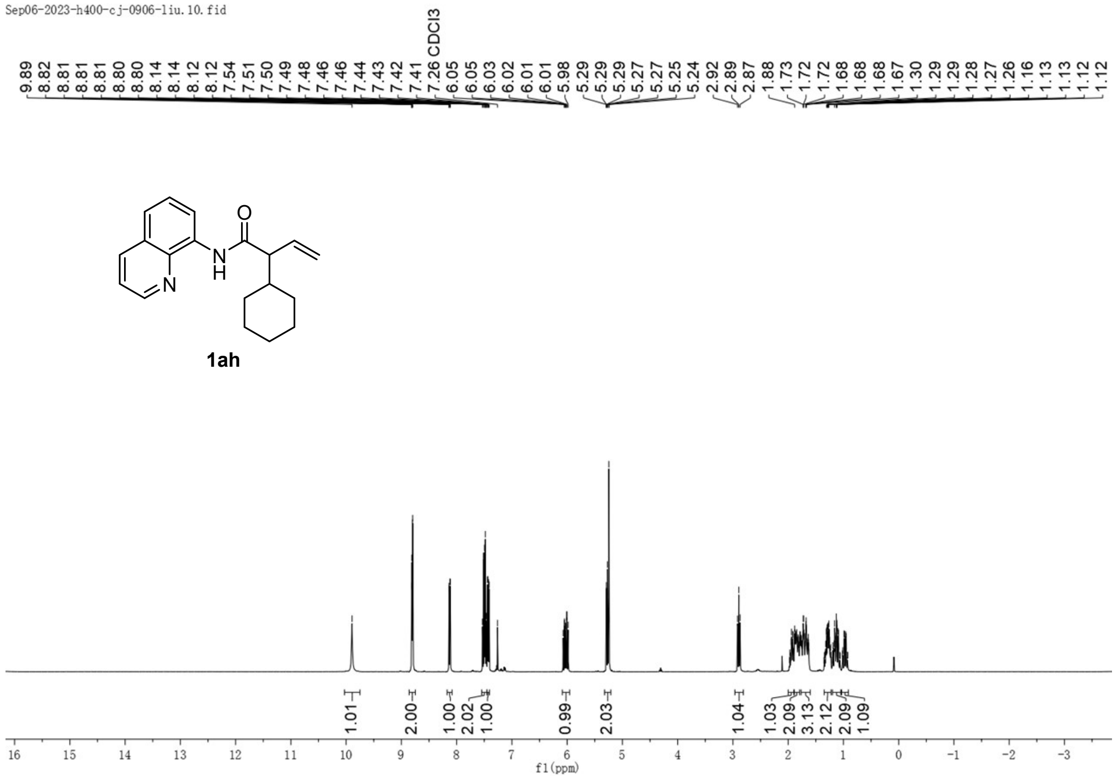

^{13}C NMR of 1ah ($CDCl_3$, 101 MHz)

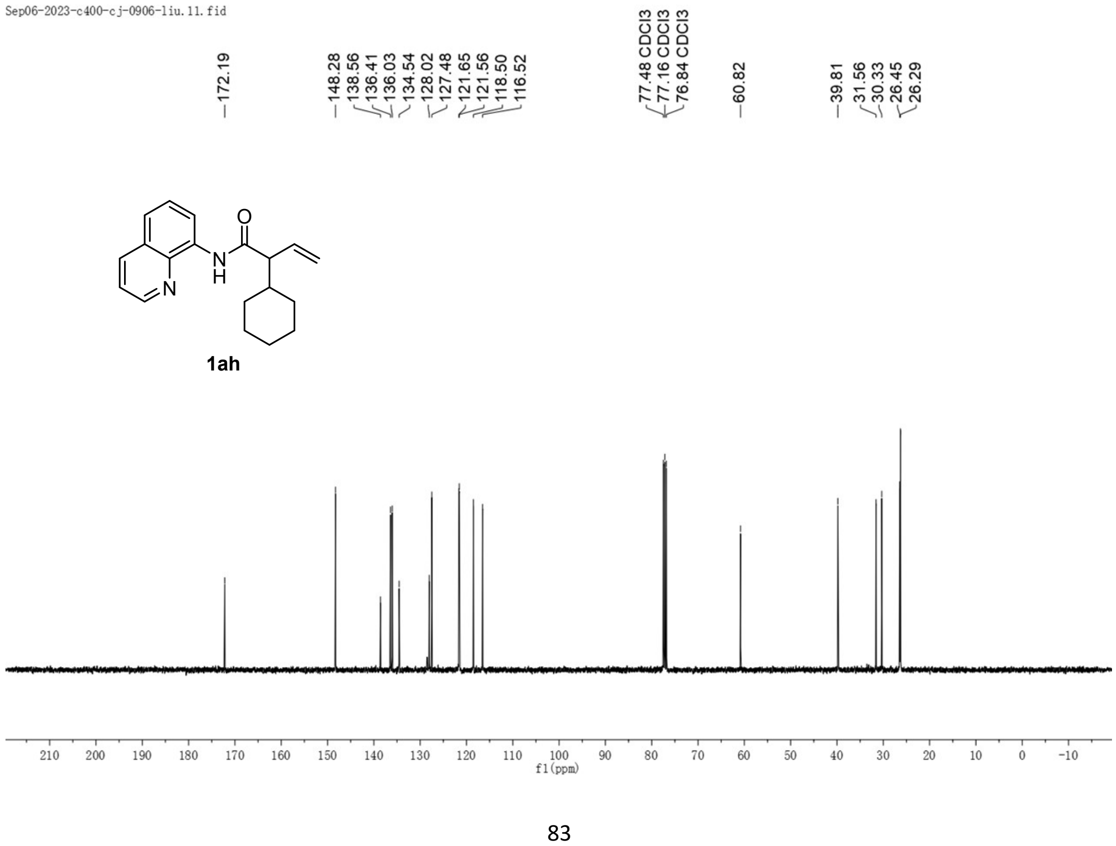

^{1}H NMR of 1ai ($CDCl_3$, 400 MHz)

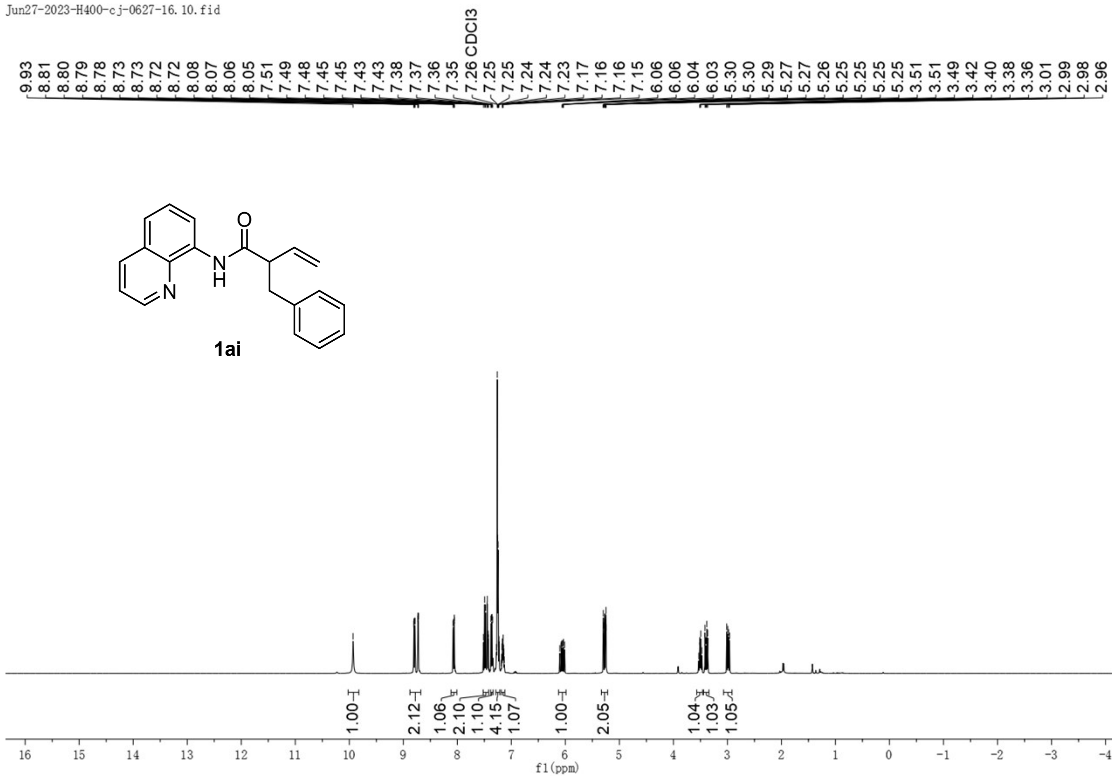

^{13}C NMR of 1ai ($CDCl_3$, 101 MHz)

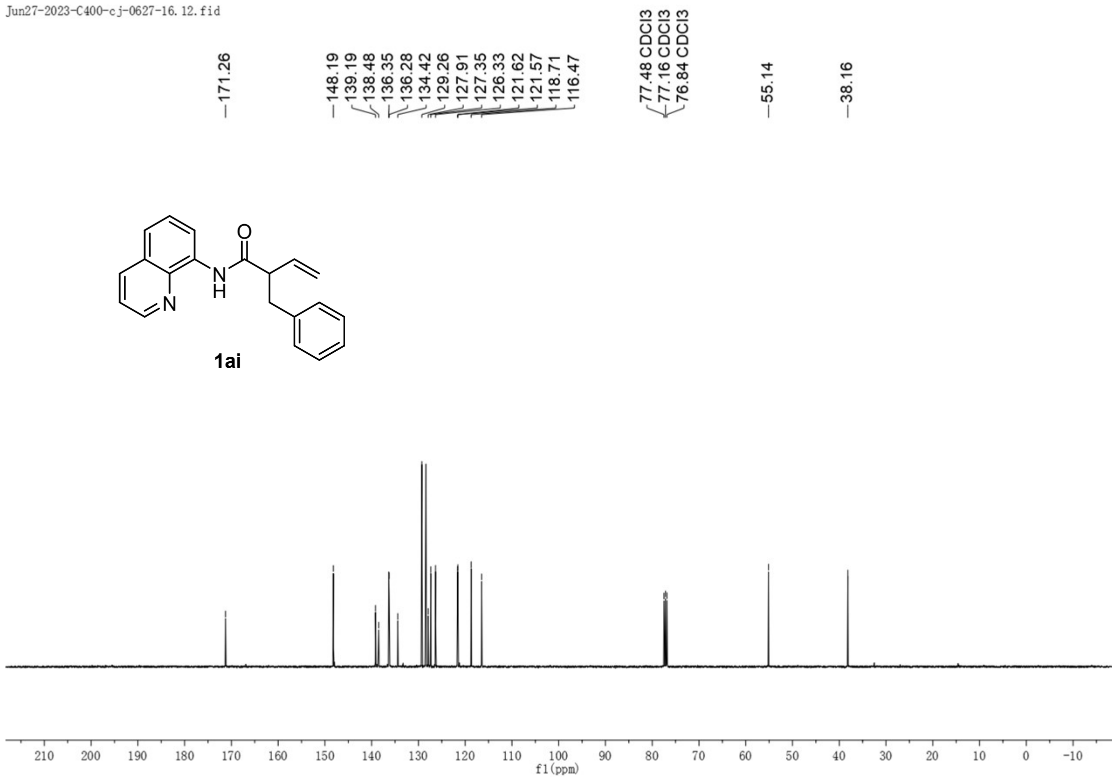

^{1}H NMR of 1aj ($CDCl_3$, 400 MHz)

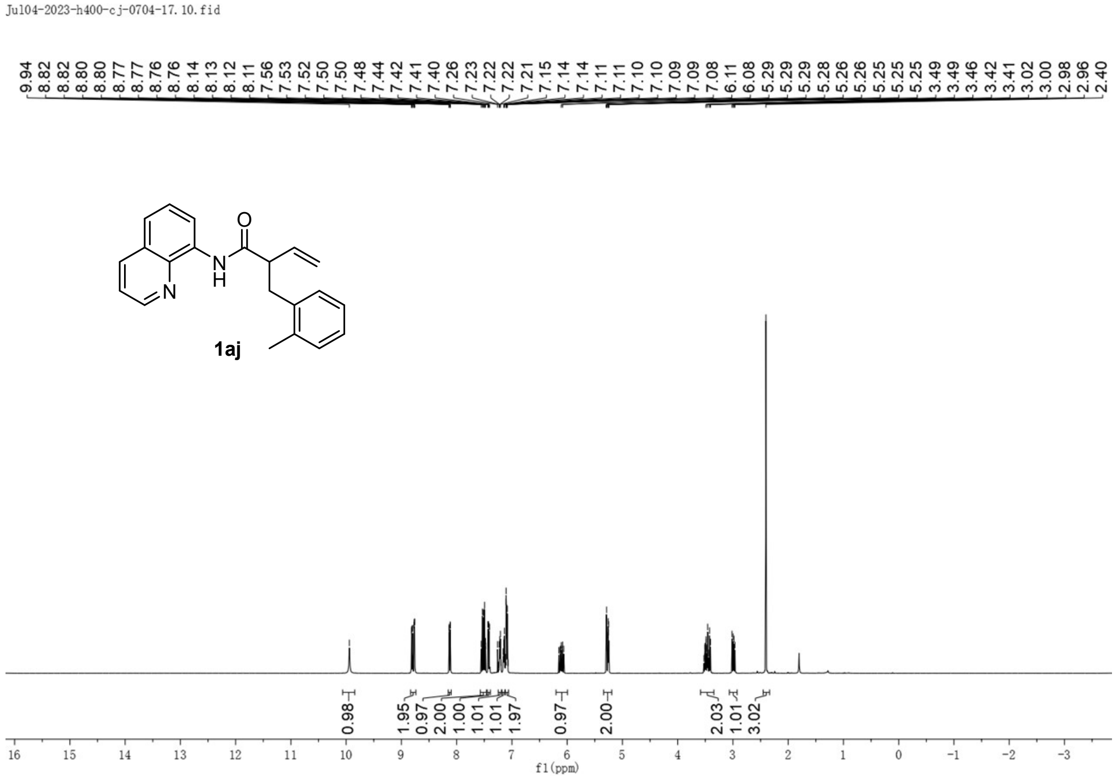

^{13}C NMR of 1aj ($CDCl_3$, 101 MHz)

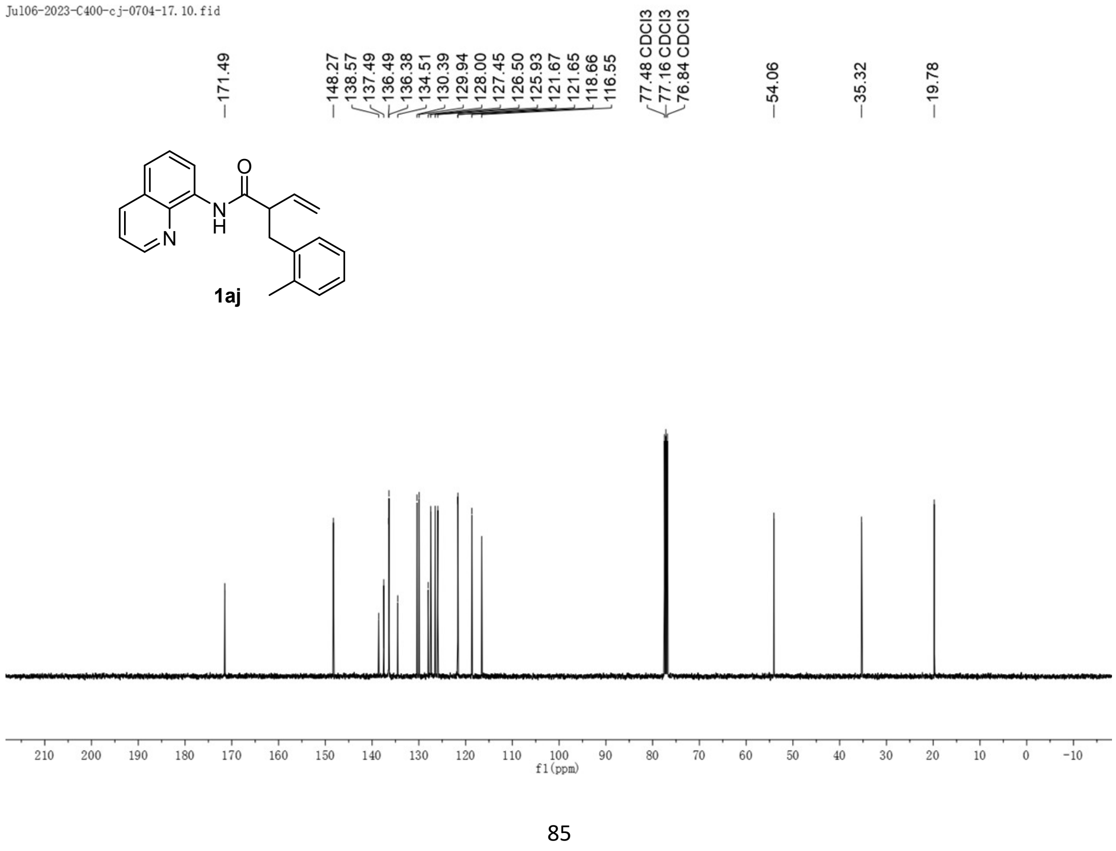

^{1}H NMR of 1ak ($CDCl_3$, 400 MHz)

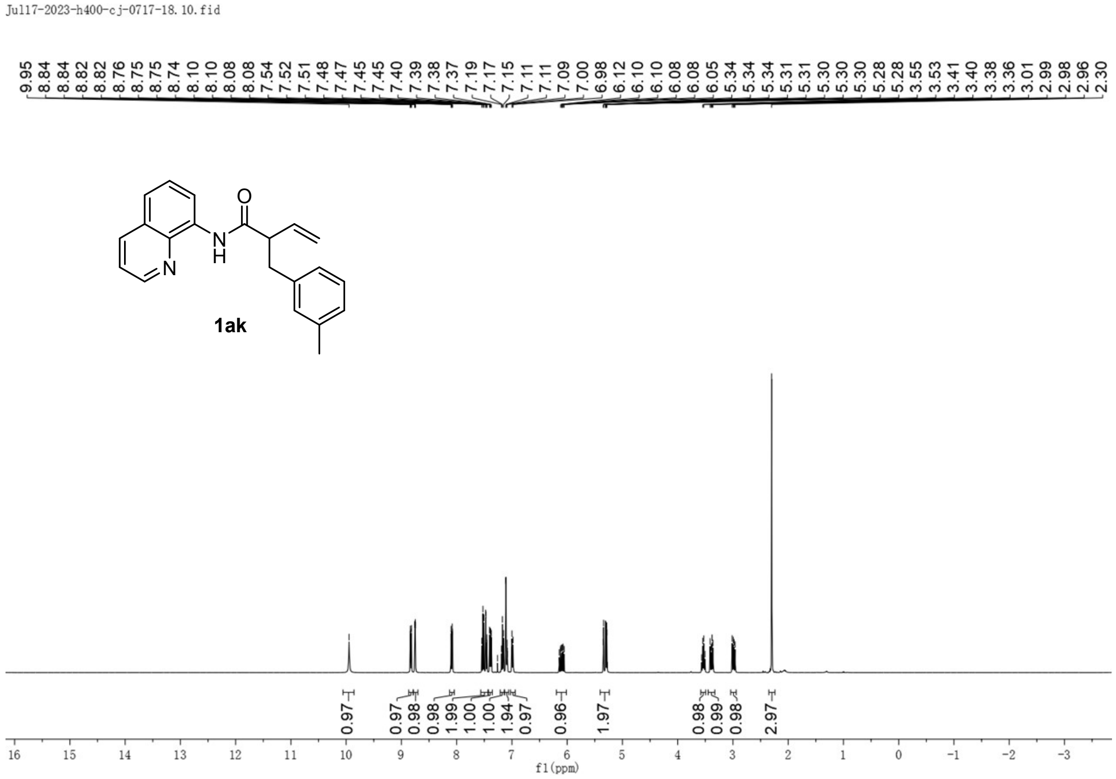

^{13}C NMR of 1ak ($CDCl_3$, 101 MHz)

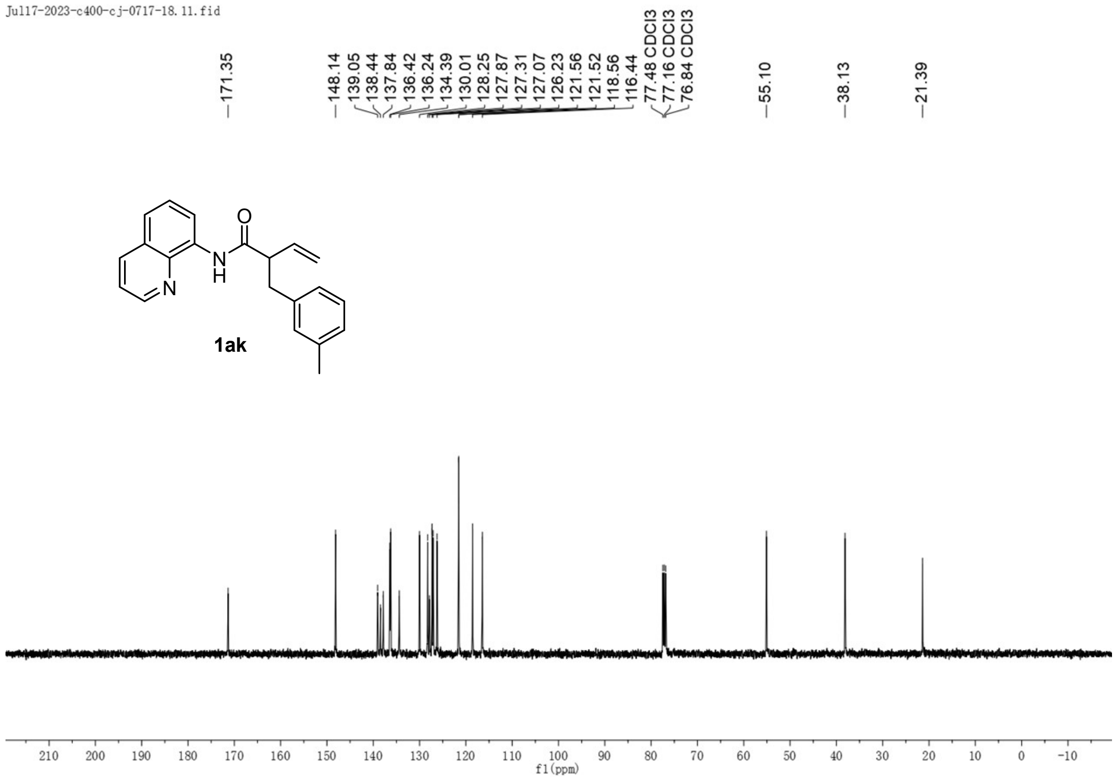

^{1}H NMR of 1al ($CDCl_3$, 400 MHz)

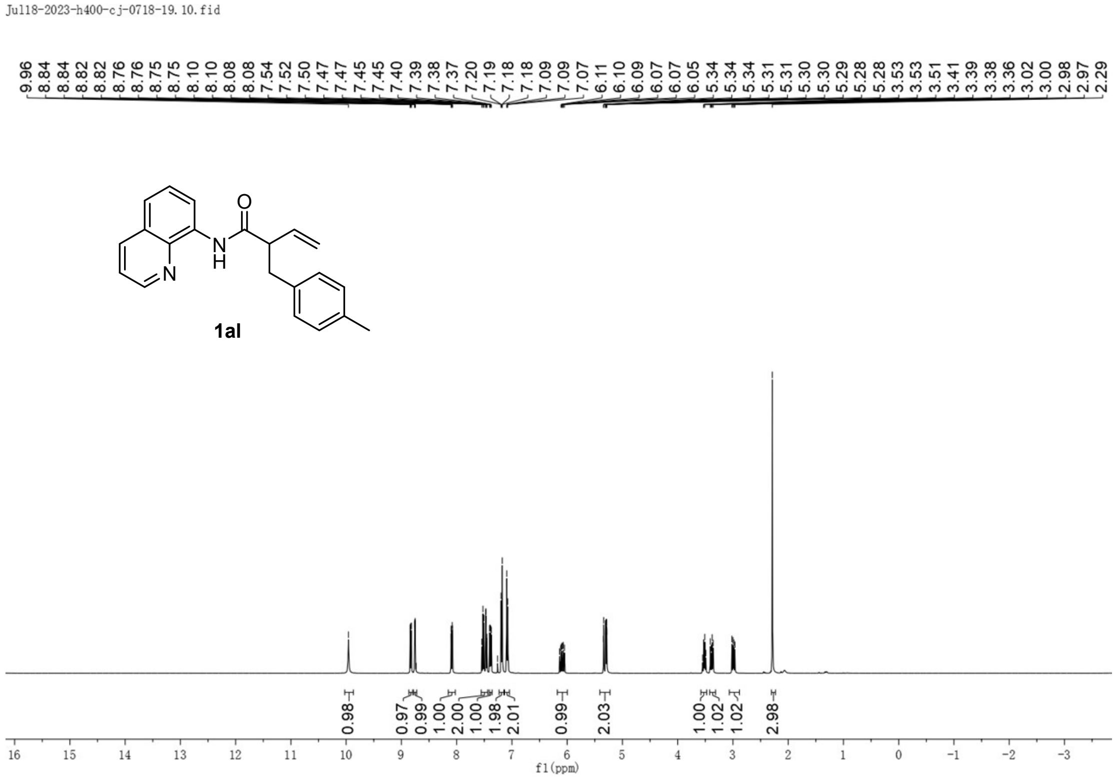

^{13}C NMR of 1al ($CDCl_3$, 101 MHz)

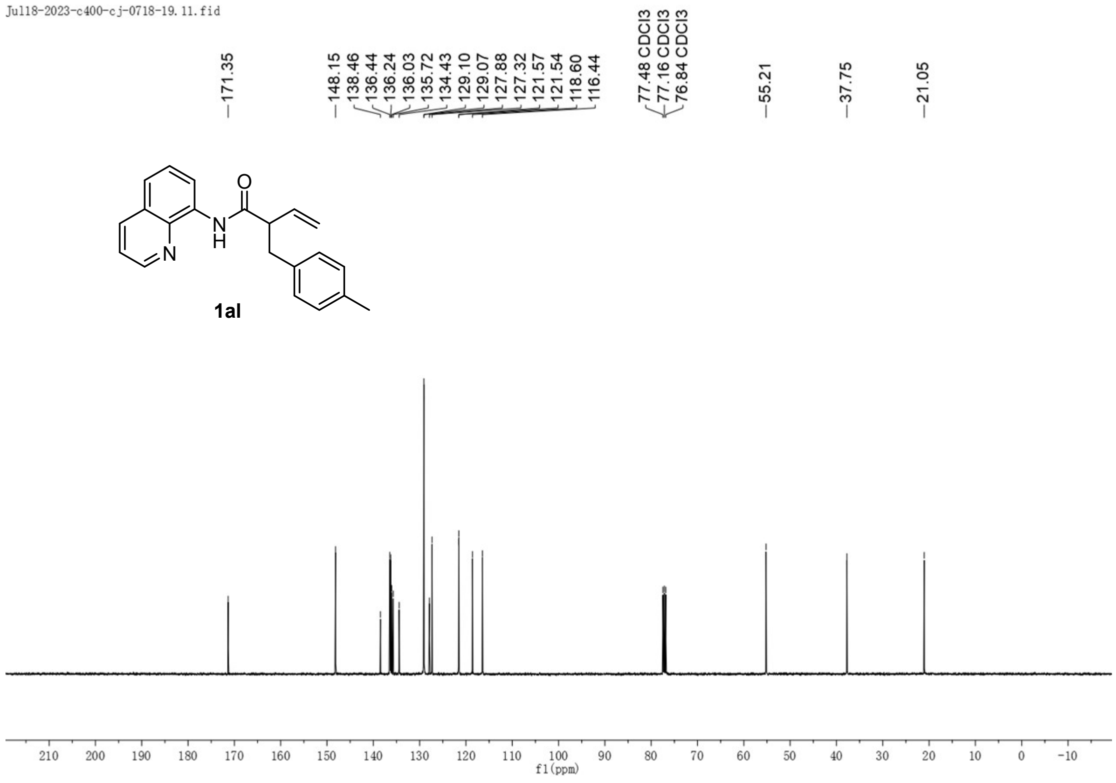

^{1}H NMR of 1am ($CDCl_3$, 400 MHz)

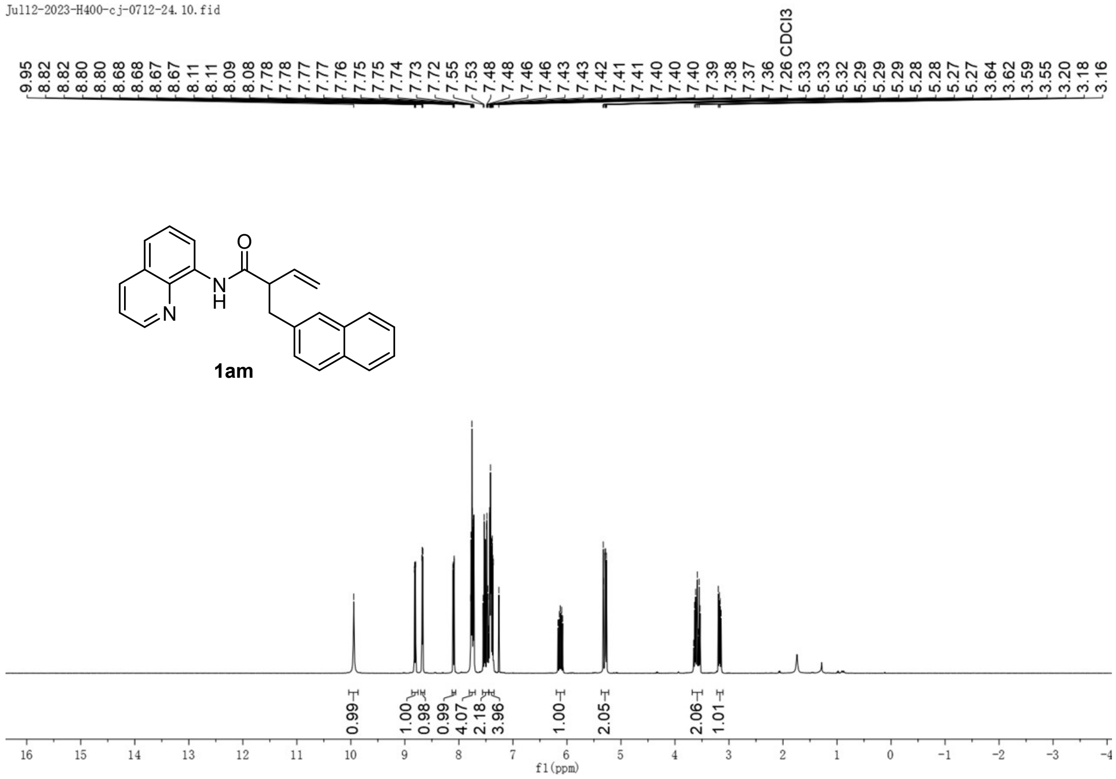

^{13}C NMR of 1am ($CDCl_3$, 101 MHz)

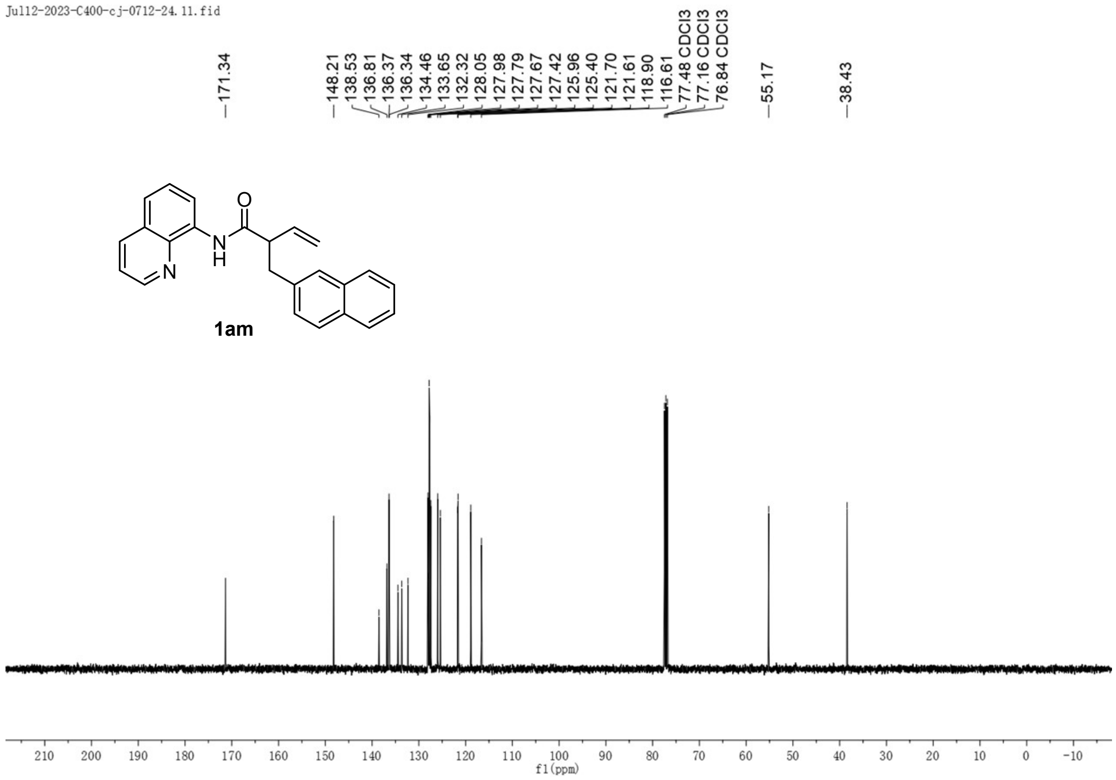

^{1}H NMR of 1an ($CDCl_3$, 400 MHz)

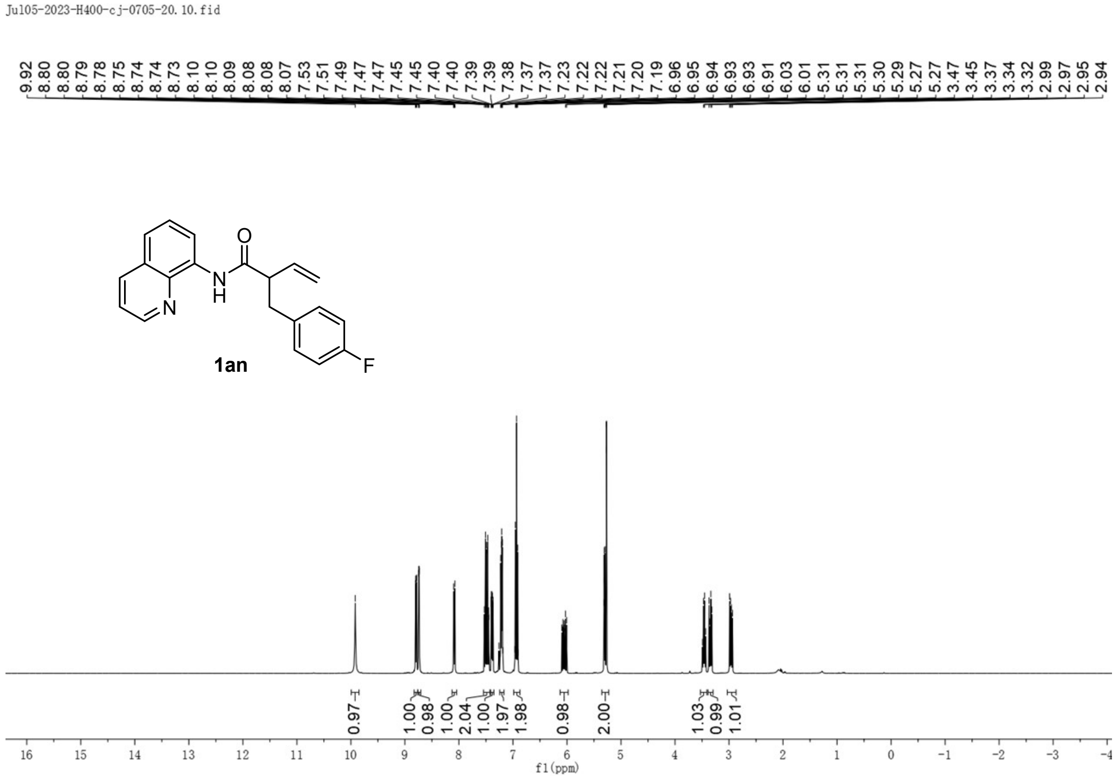

^{13}C NMR of 1an ($CDCl_3$, 101 MHz)

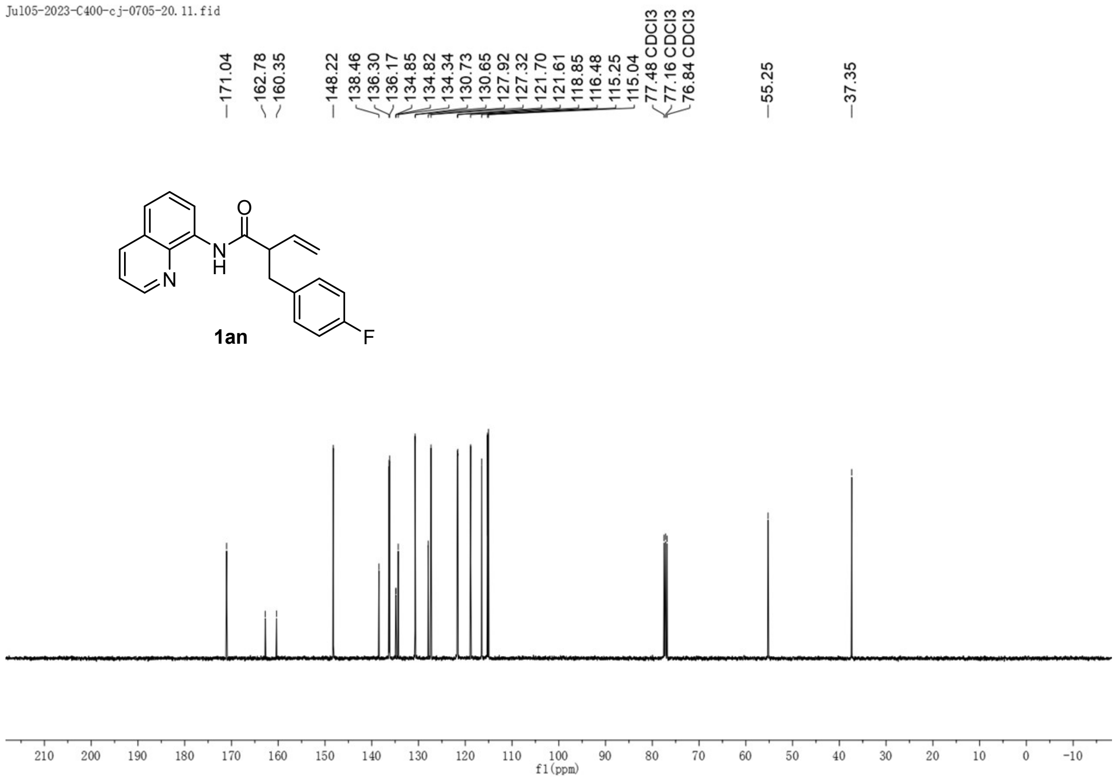

^{19}F NMR of 1an ($CDCl_3$, 376 MHz)

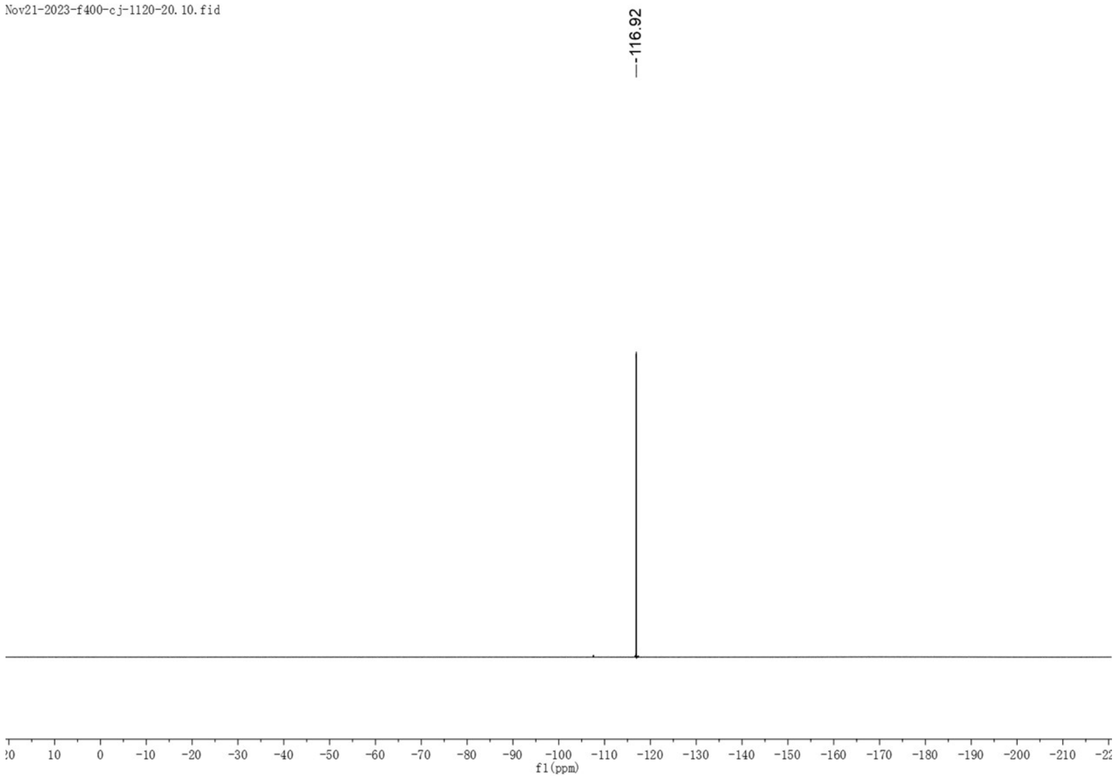

^{1}H NMR of 1ao ($CDCl_3$, 400 MHz)

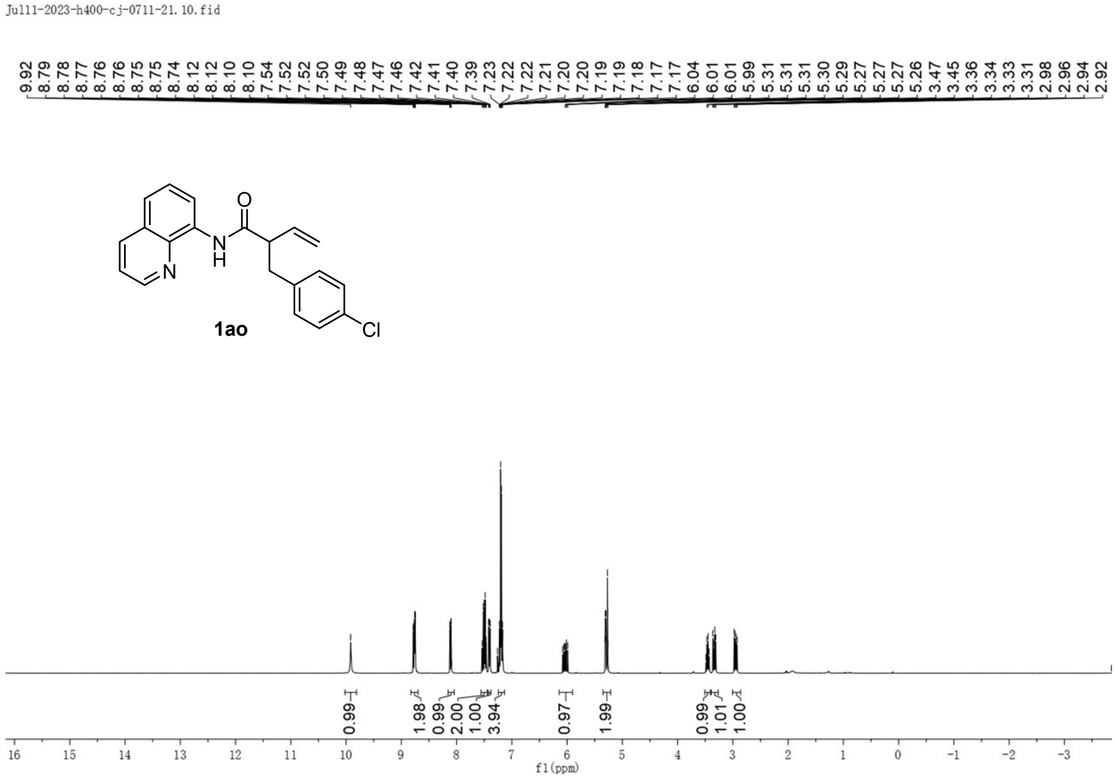

^{13}C NMR of 1ao ($CDCl_3$, 101 MHz)

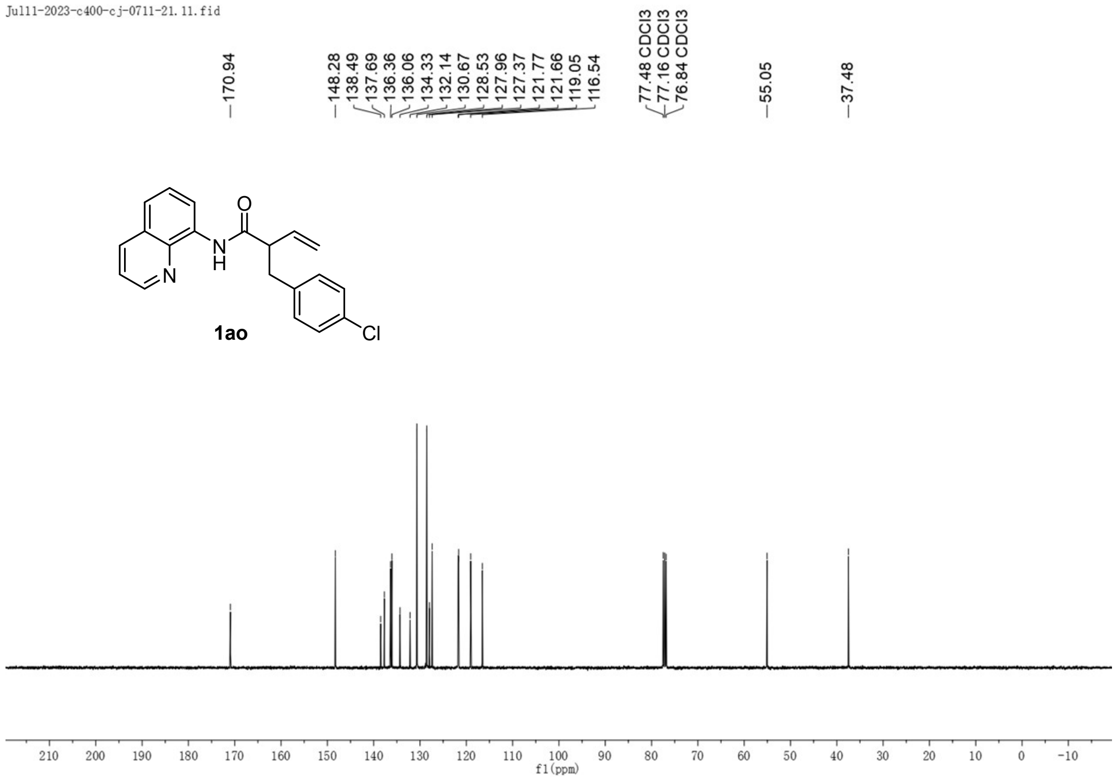

^{1}H NMR of 1ap ($CDCl_3$, 400 MHz)

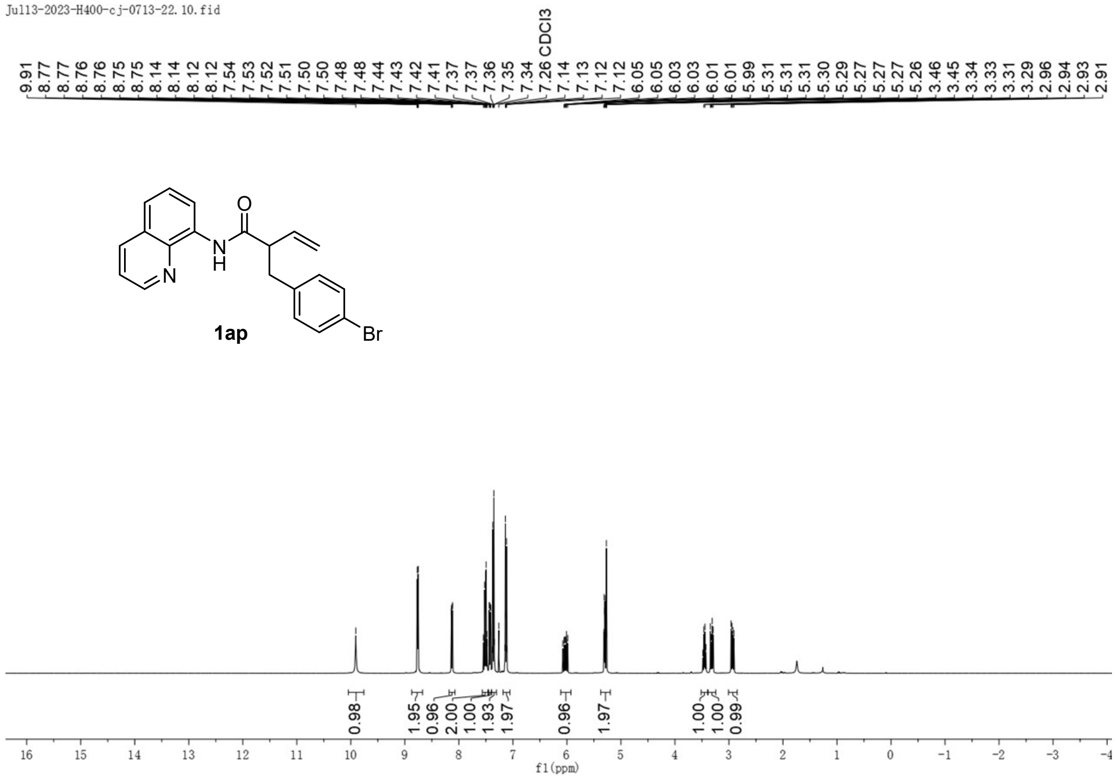

^{13}C NMR of 1ap ($CDCl_3$, 101 MHz)

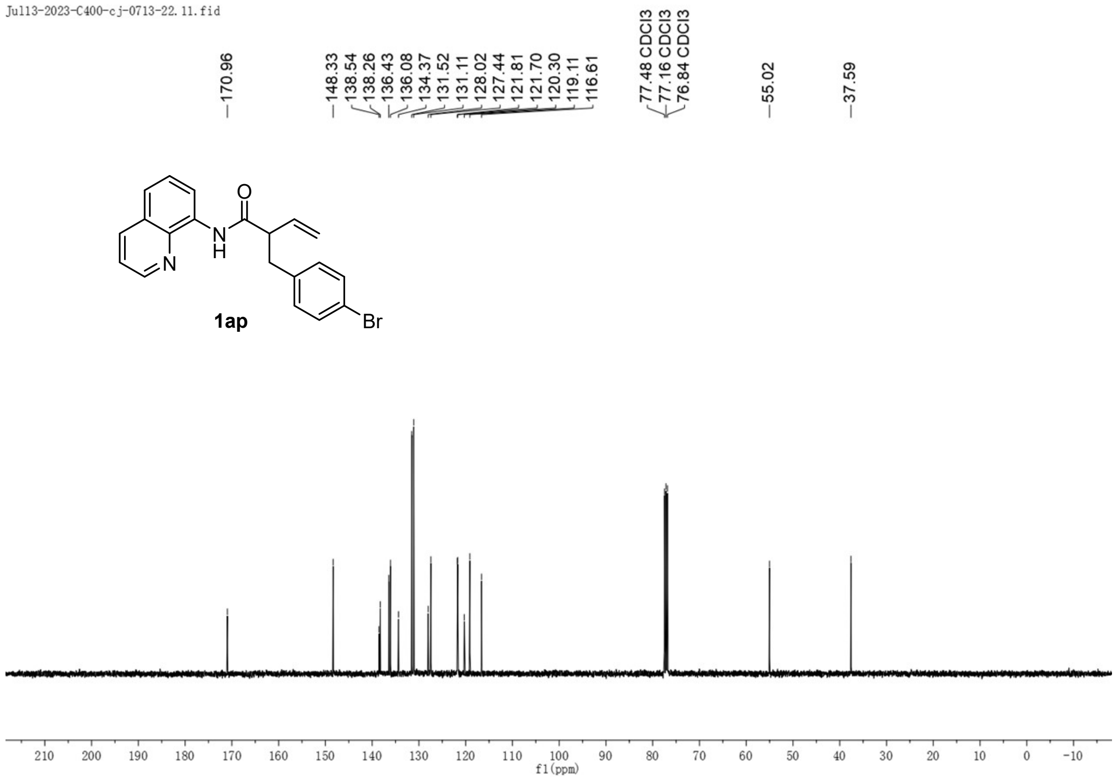

^1H NMR of 1aq ($CDCl_3$, 400 MHz)

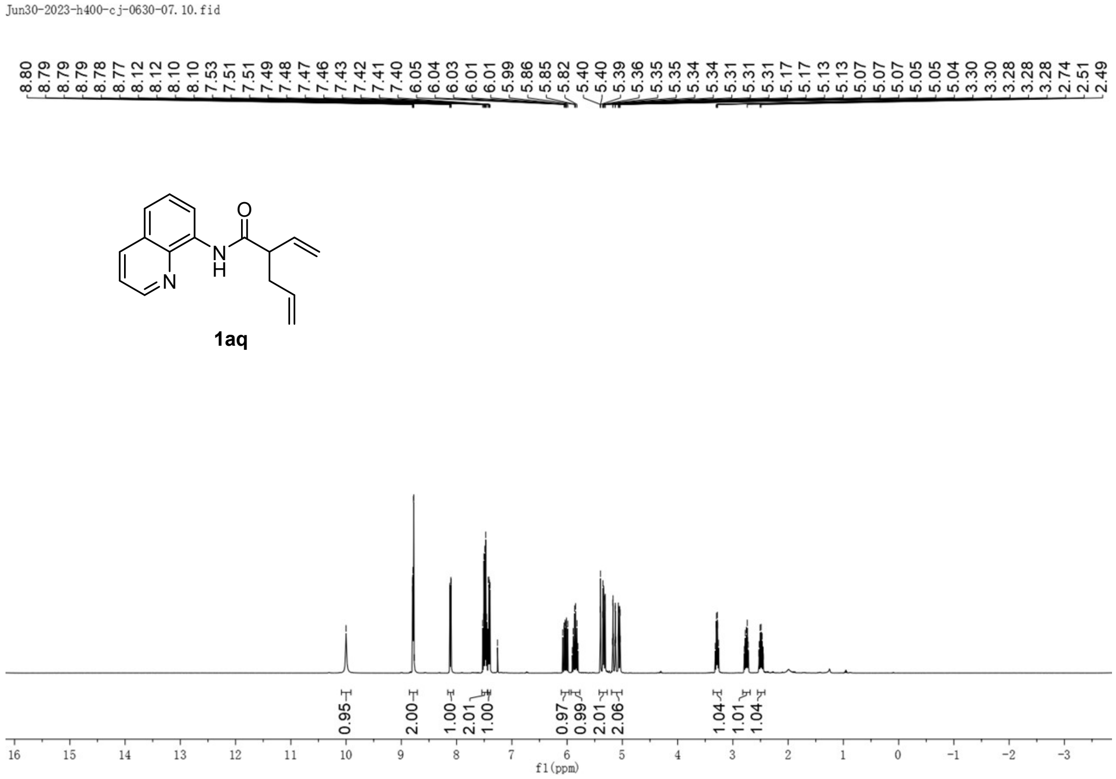

^{13}C NMR of 1aq ($CDCl_3$, 101 MHz)

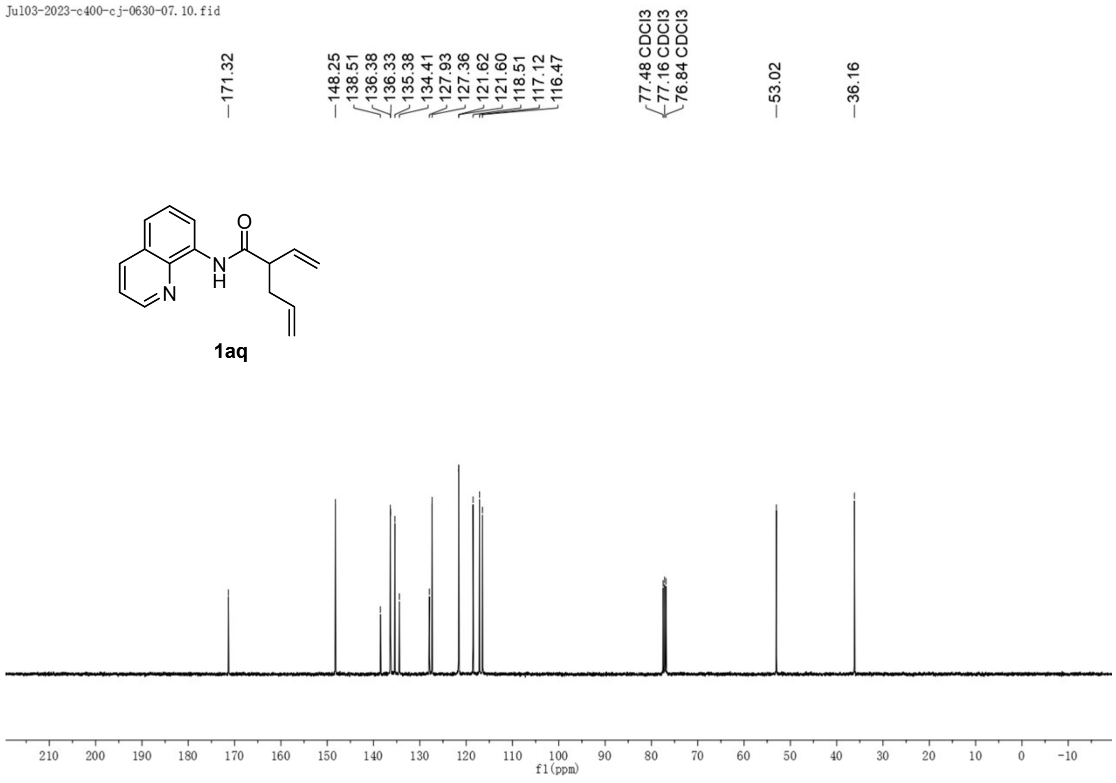

^{1}H NMR of 1ar ($CDCl_3$, 400 MHz)

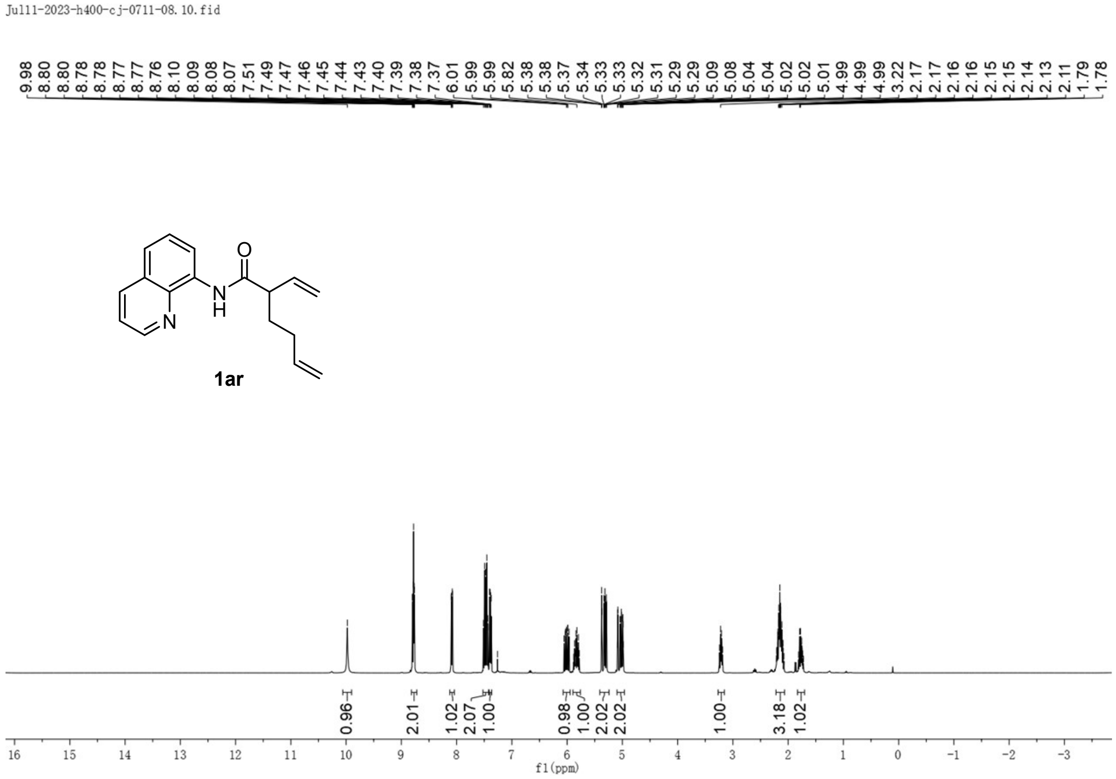

^{13}C NMR of 1ar ($CDCl_3$, 101 MHz)

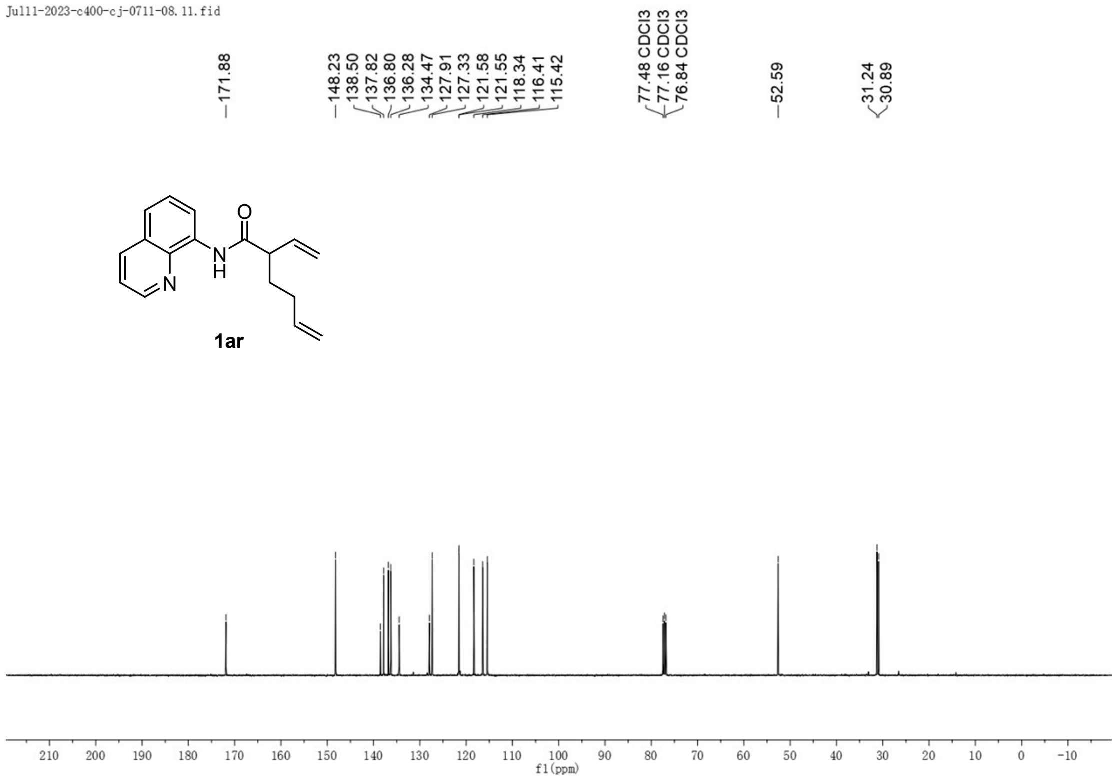

^1H NMR of 1as ($CDCl_3$, 400 MHz)

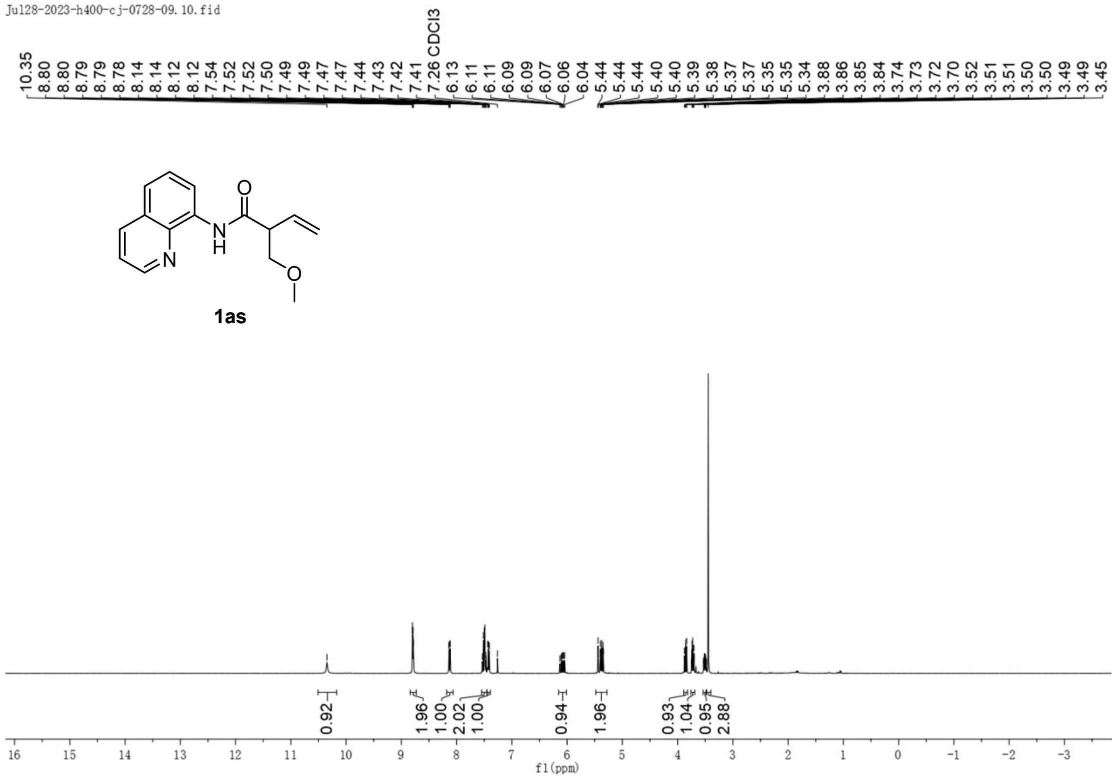

^{13}C NMR of 1as ($CDCl_3$, 101 MHz)

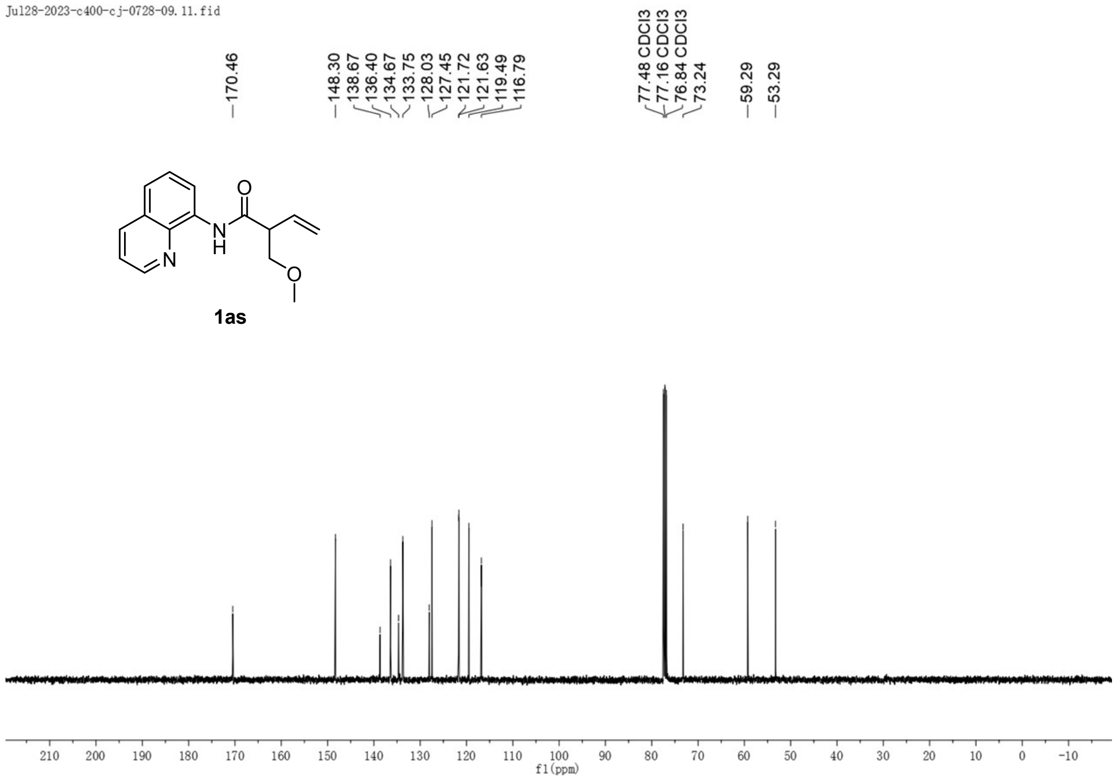

^{1}H NMR of 1at ($CDCl_3$, 400 MHz)

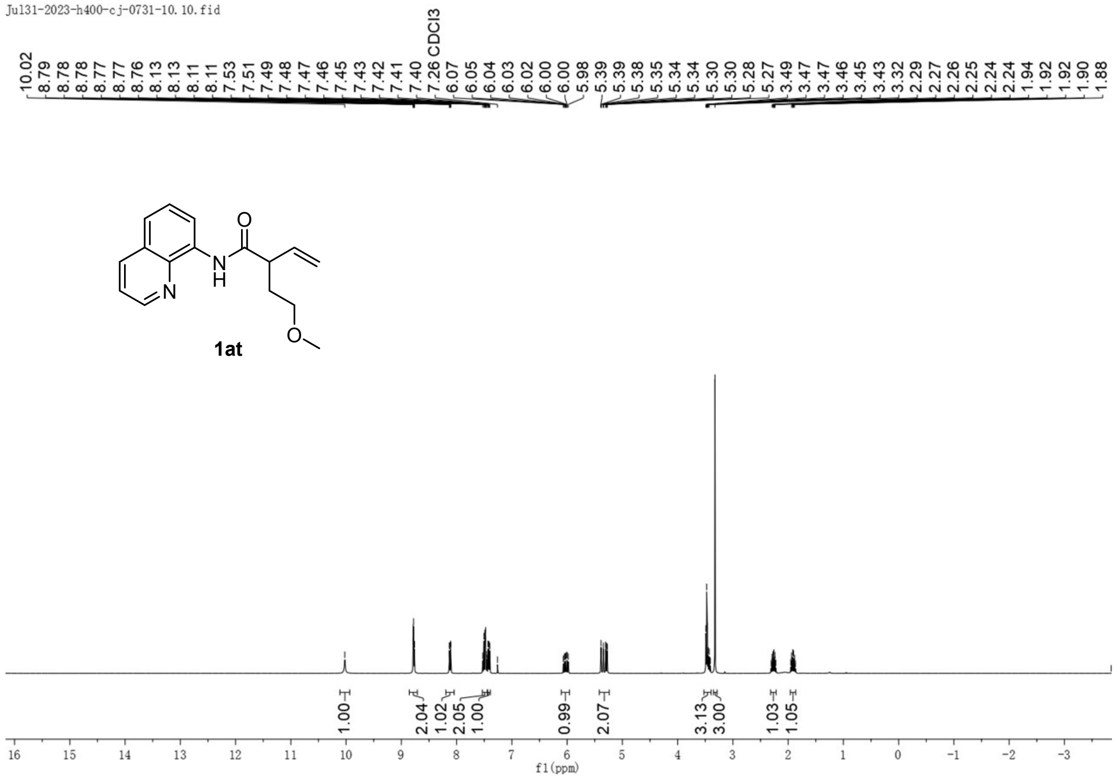

^{13}C NMR of 1at ($CDCl_3$, 101 MHz)

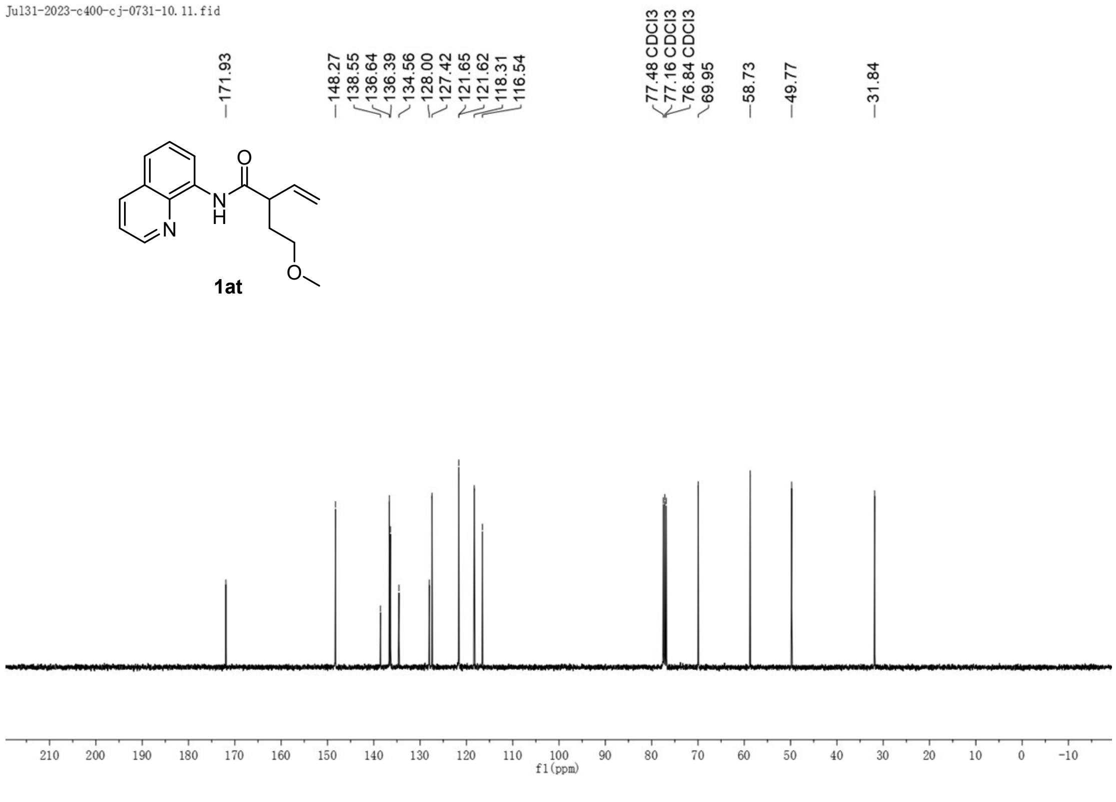

^{1}H NMR of 1au ($CDCl_3$, 400 MHz)

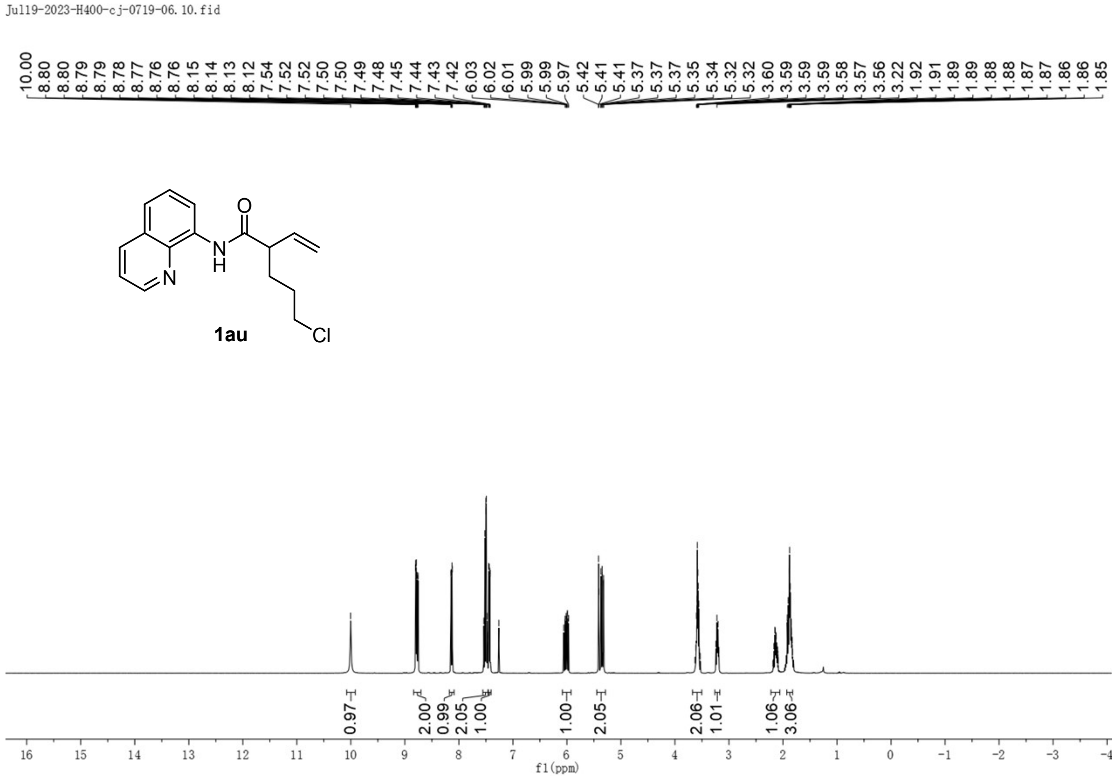

^{13}C NMR of 1au ($CDCl_3$, 101 MHz)

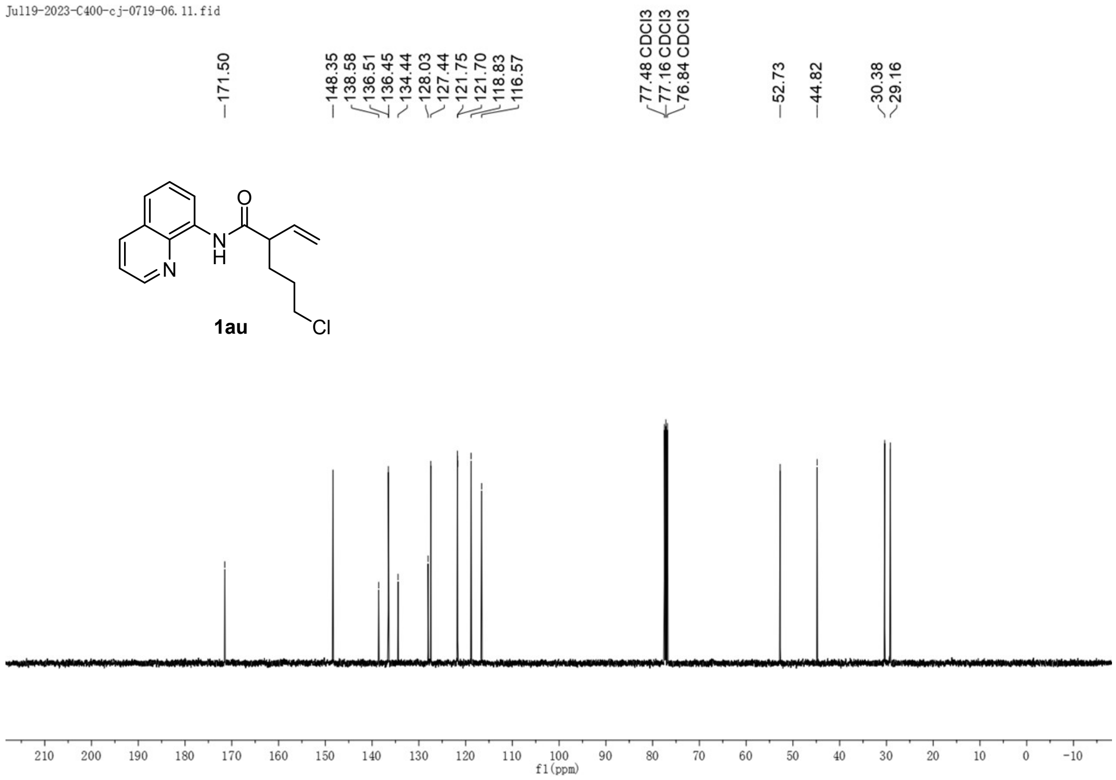

^{1}H NMR of 1av ($CDCl_3$, 400 MHz)

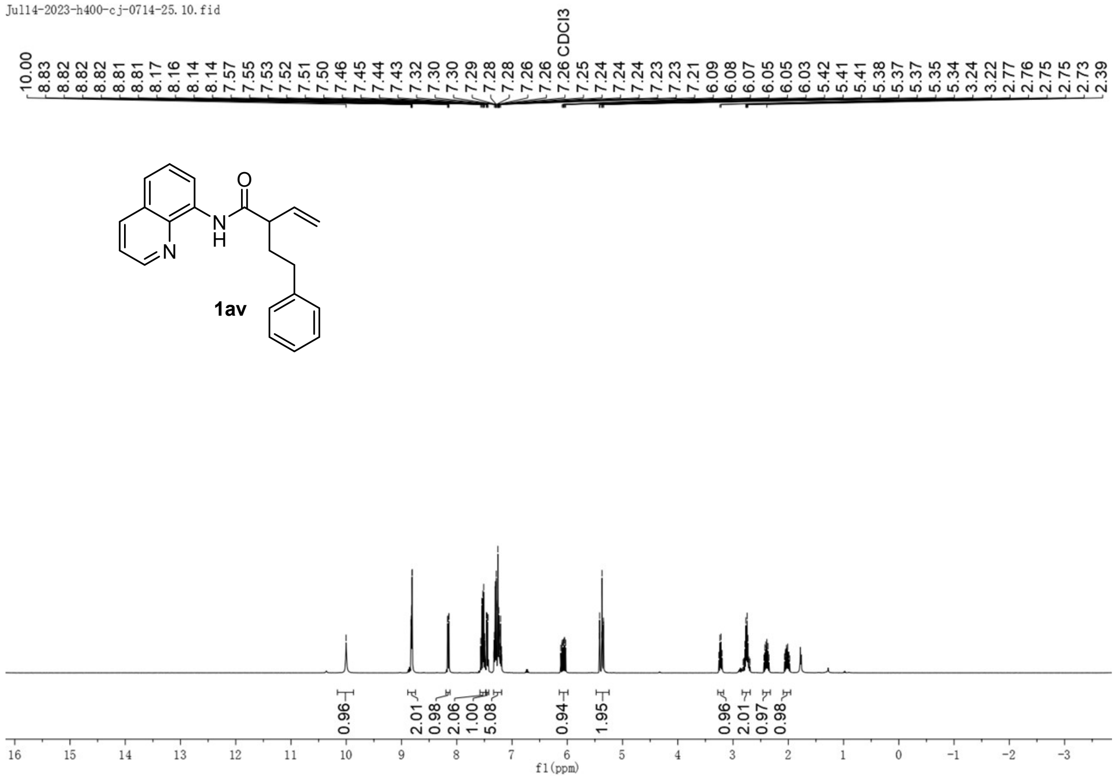

^{13}C NMR of 1av ($CDCl_3$, 101 MHz)

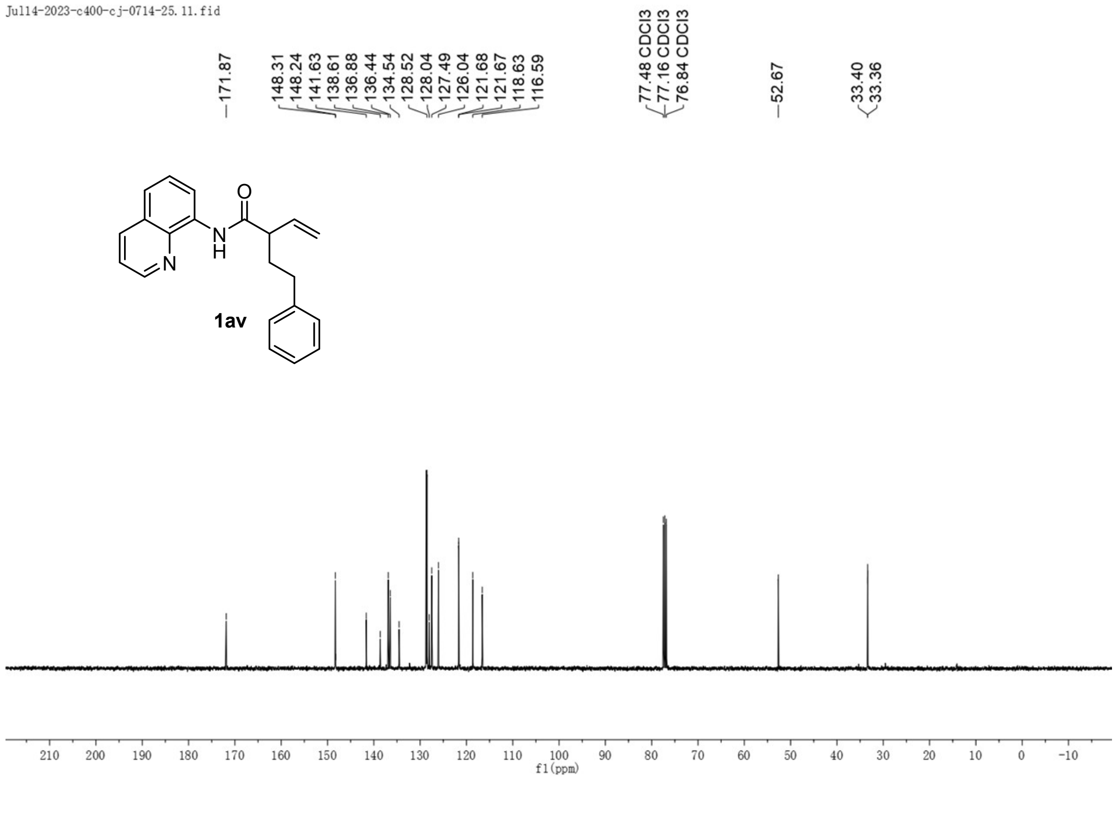

^{1}H NMR of 1aw ($CDCl_3$, 400 MHz)

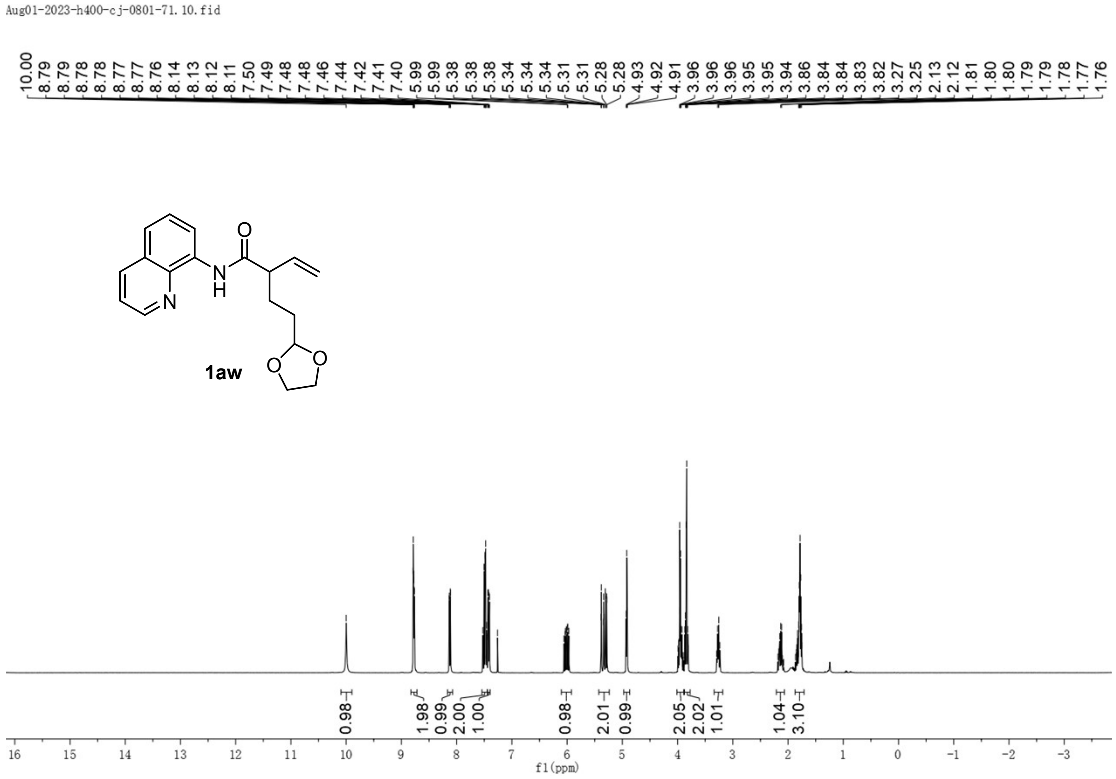

^{13}C NMR of 1aw ($CDCl_3$, 101 MHz)

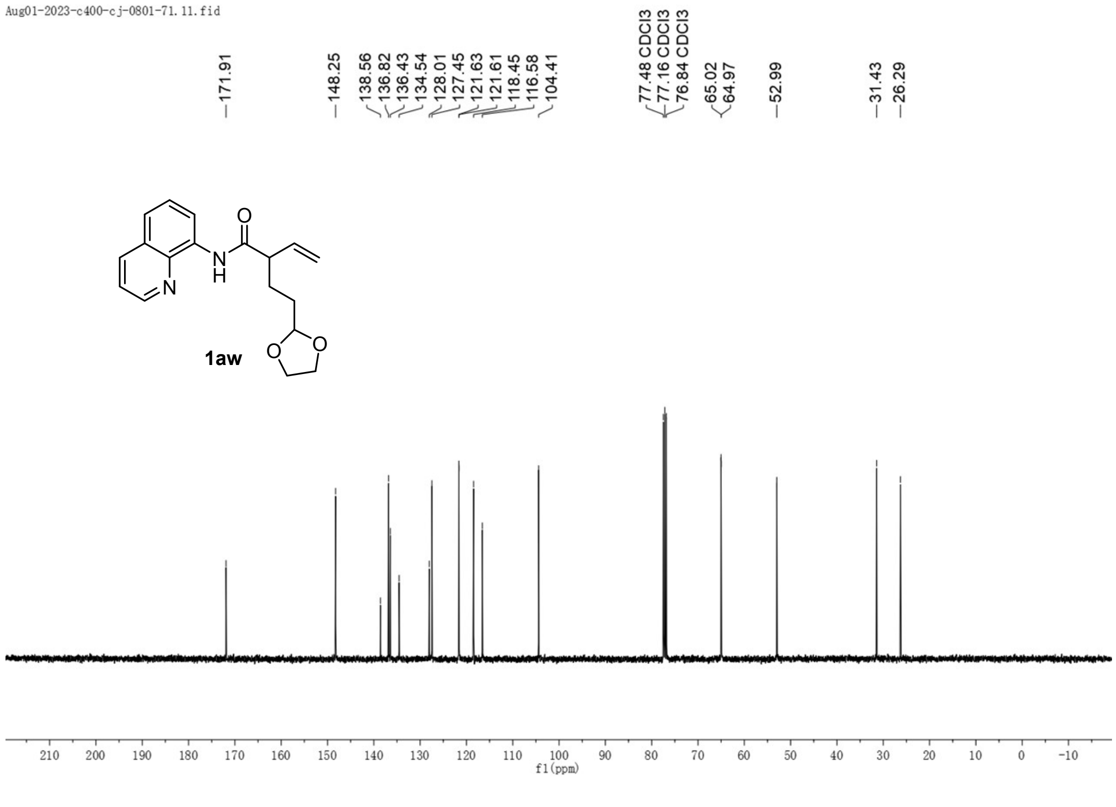

^{1}H NMR of 1ax ($CDCl_3$, 400 MHz)

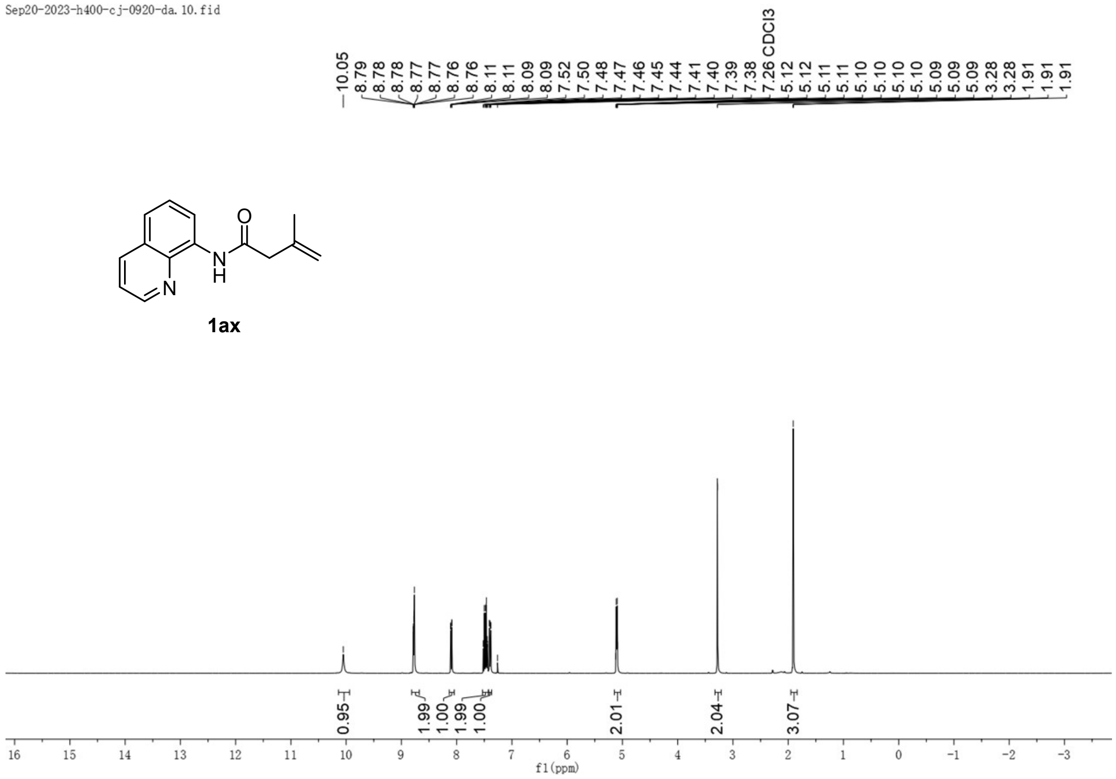

^{13}C NMR of 1ax ($CDCl_3$, 101 MHz)

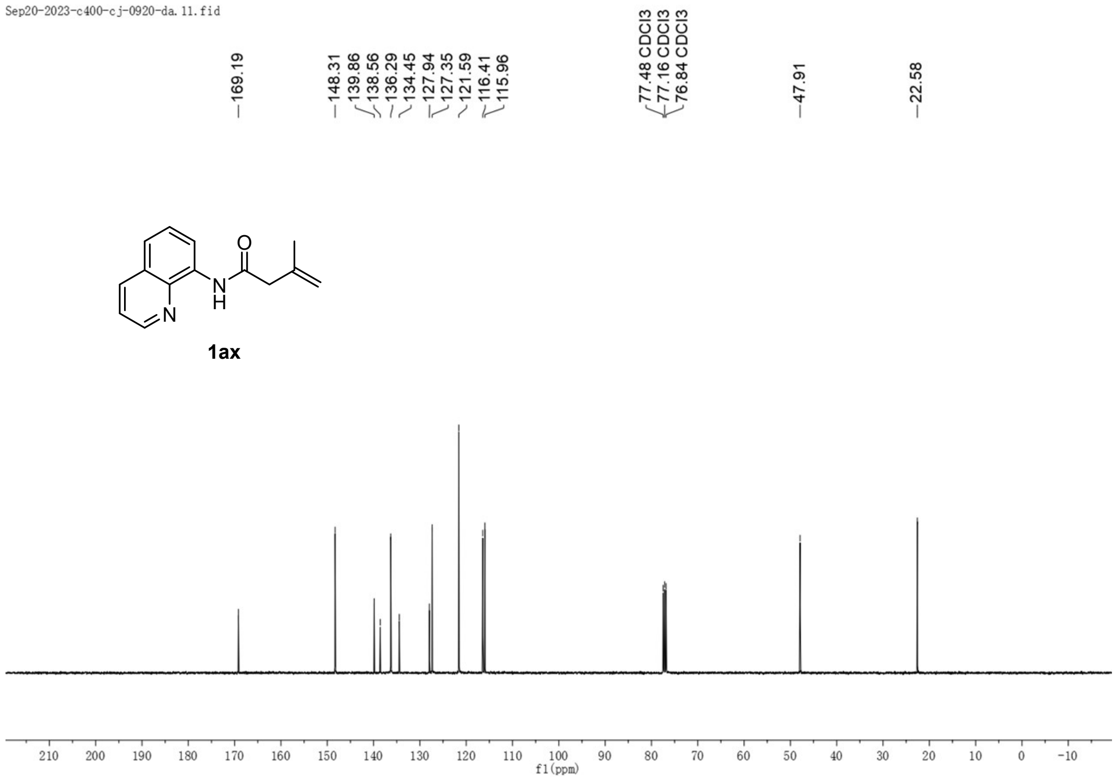

^{1}H NMR of 1ay ($CDCl_3$, 400 MHz)

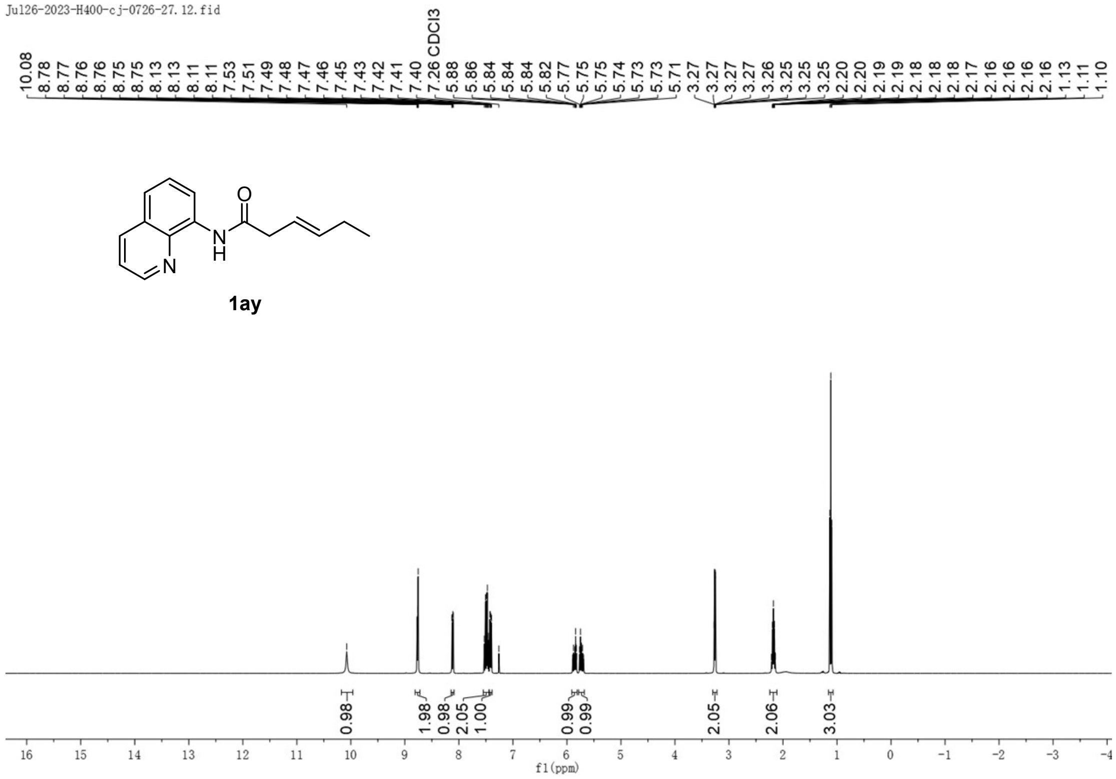

^{13}C NMR of 1ay ($CDCl_3$, 101 MHz)

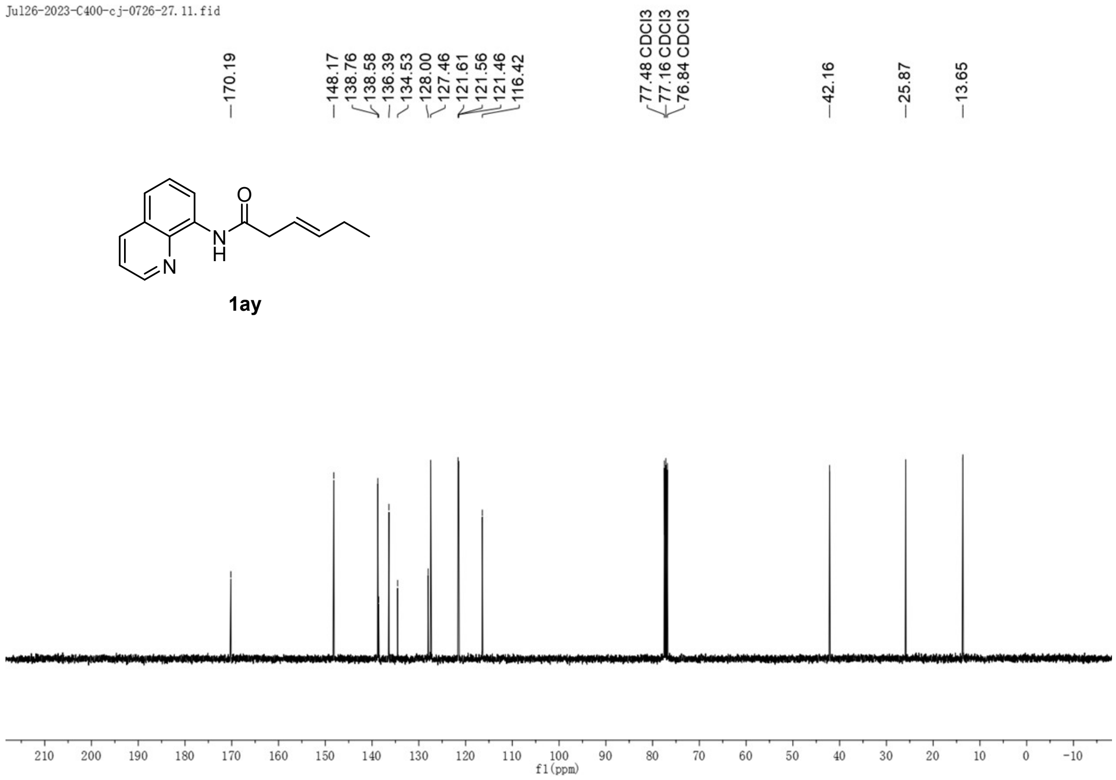

^{1}H NMR of 1ay' ($CDCl_3$, 400 MHz)

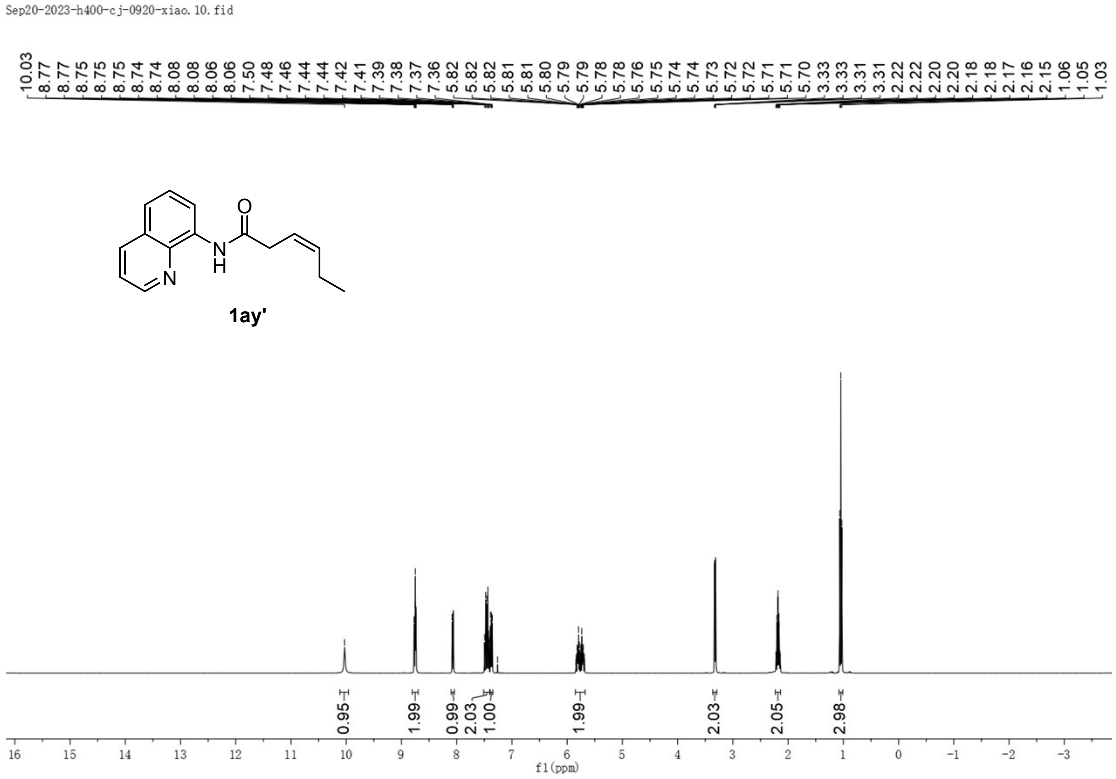

^{13}C NMR of 1ay' ($CDCl_3$, 101 MHz)

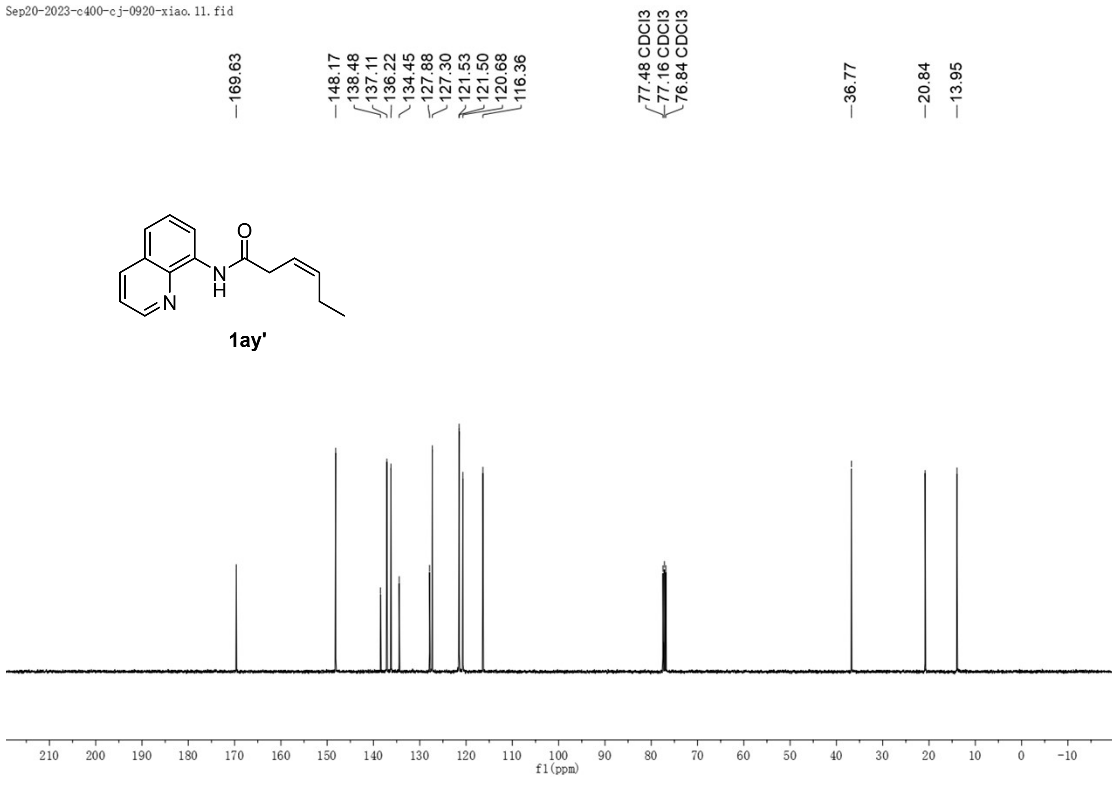

^{1}H NMR of 1bb ($CDCl_3$, 400 MHz)

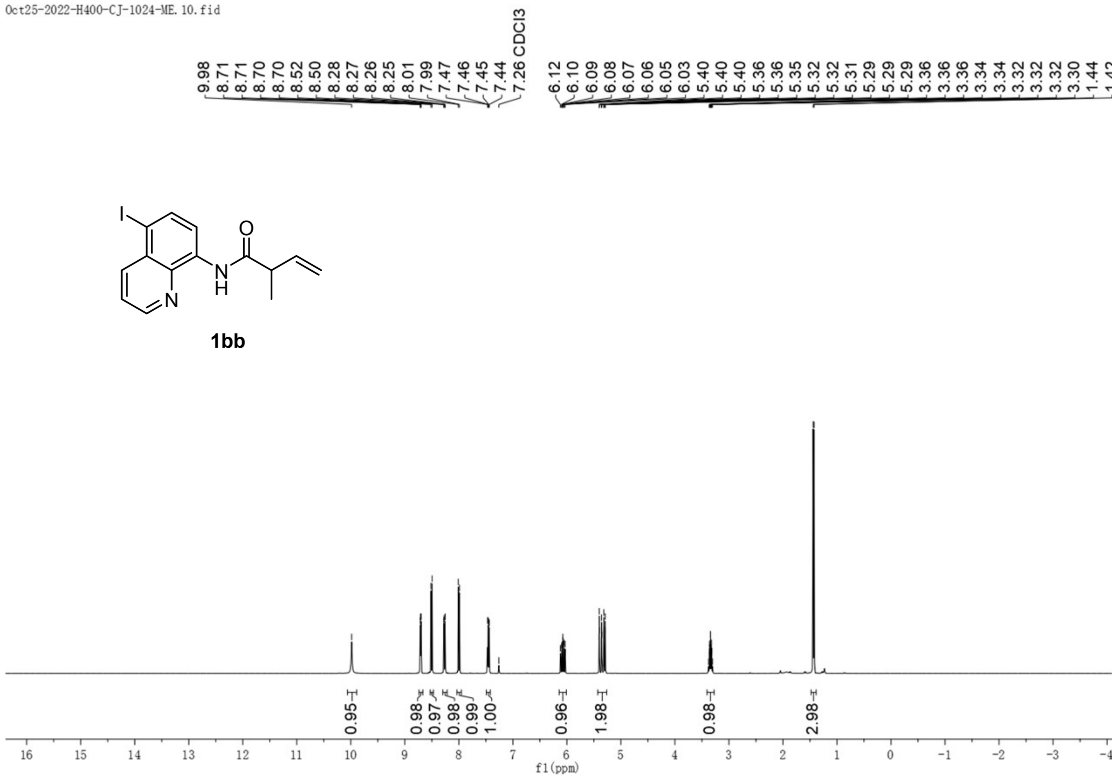

^{13}C NMR of 1bb ($CDCl_3$, 101 MHz)

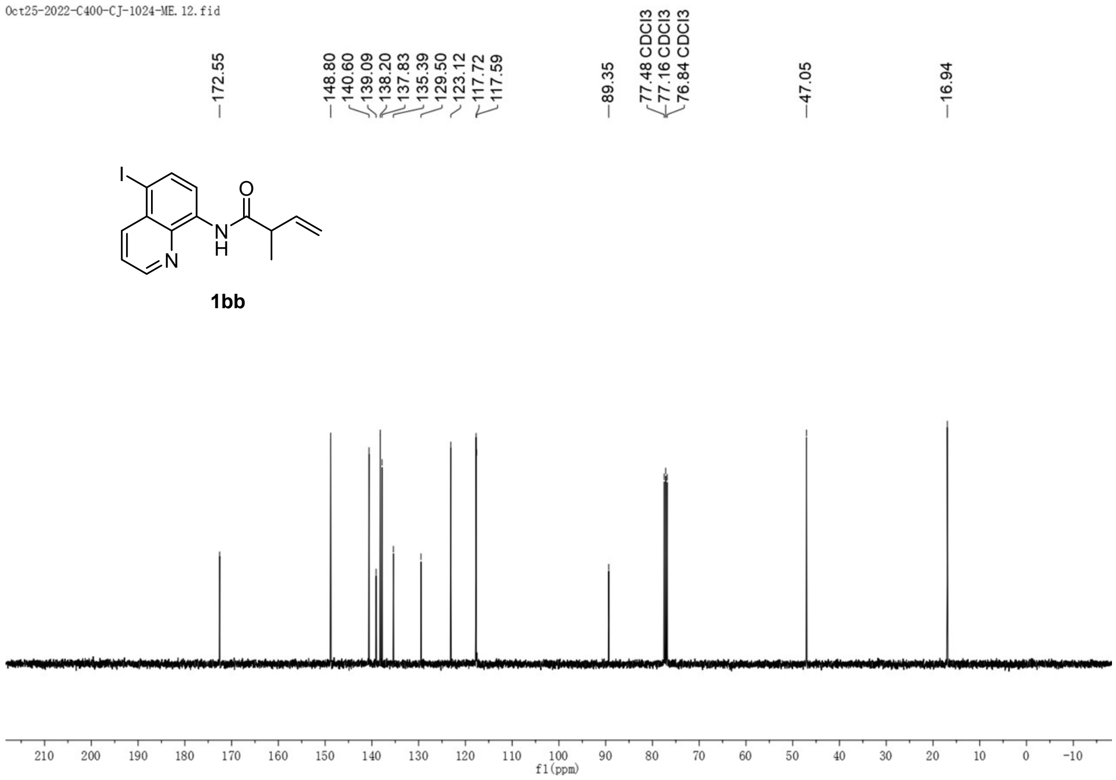

^{1}H NMR of 1bc ($CDCl_3$, 400 MHz)

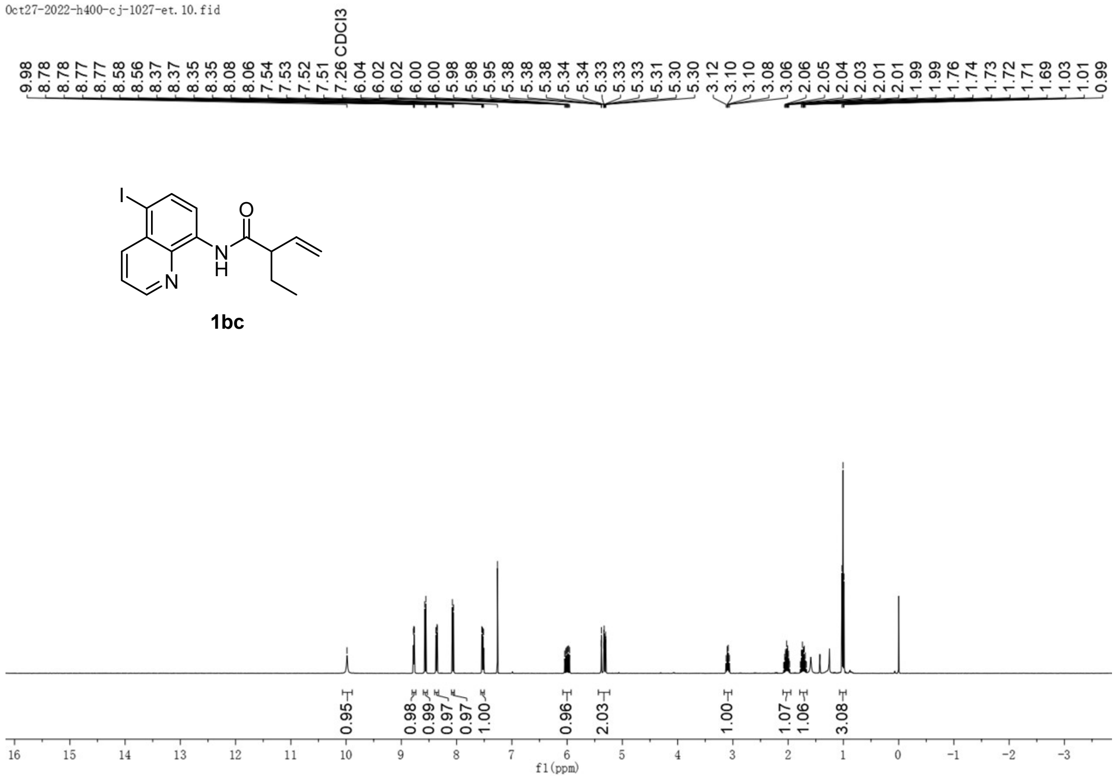

^{13}C NMR of 1bc ($CDCl_3$, 101 MHz)

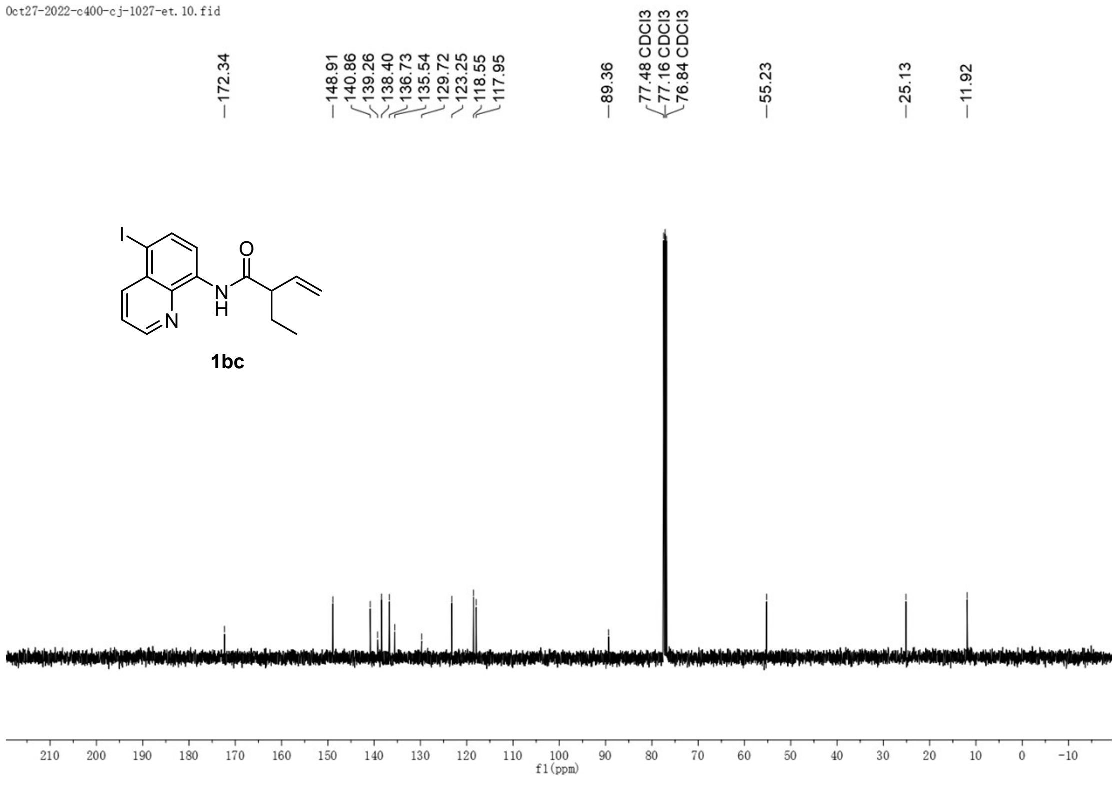

^{1}H NMR of 1bd ($CDCl_3$, 400 MHz)

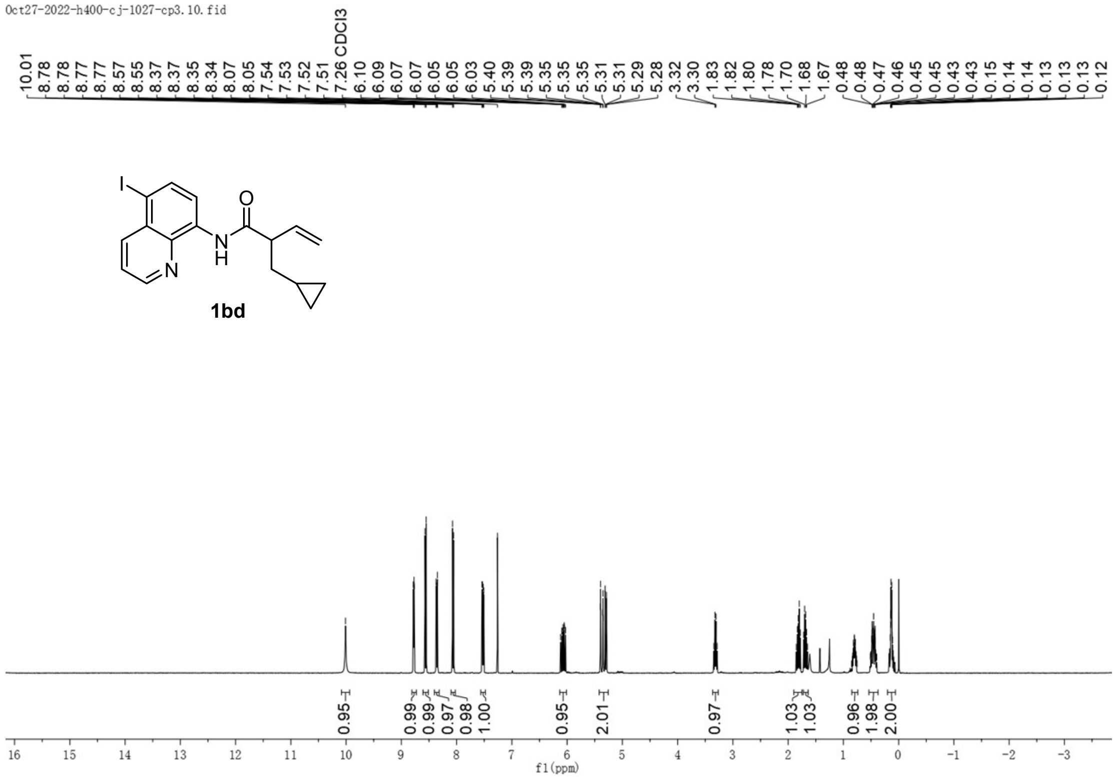

^{13}C NMR of 1bd ($CDCl_3$, 101 MHz)

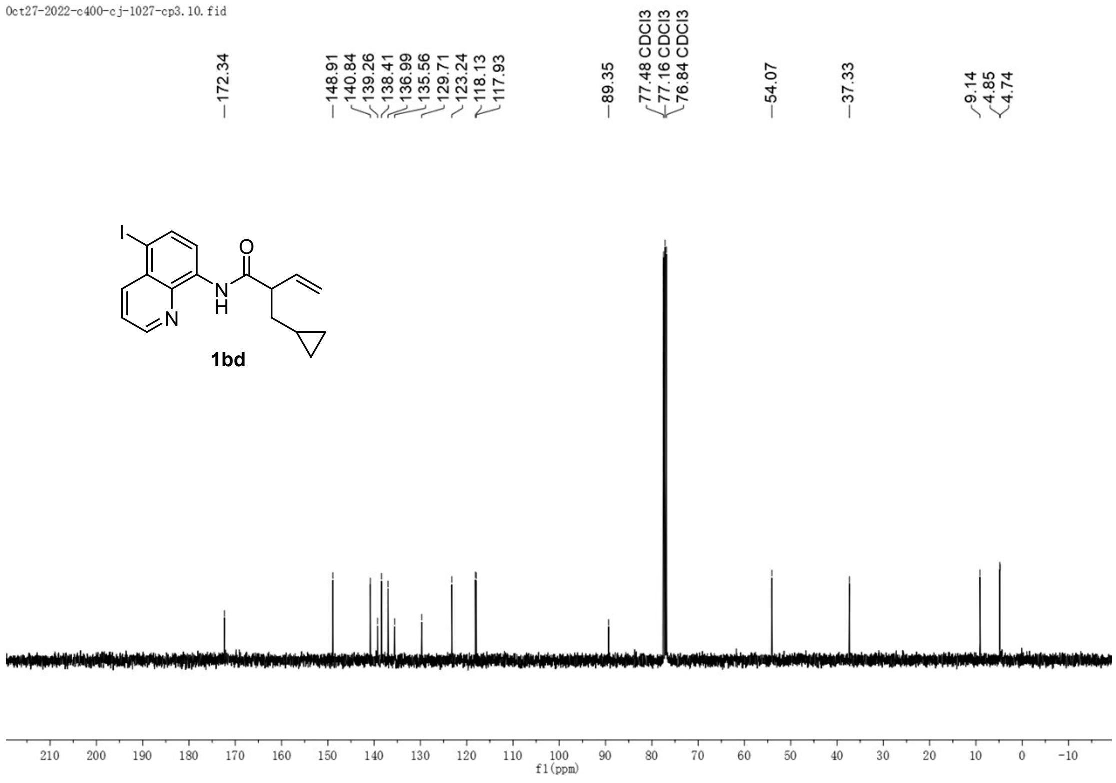

^{1}H NMR of 1be ($CDCl_3$, 400 MHz)

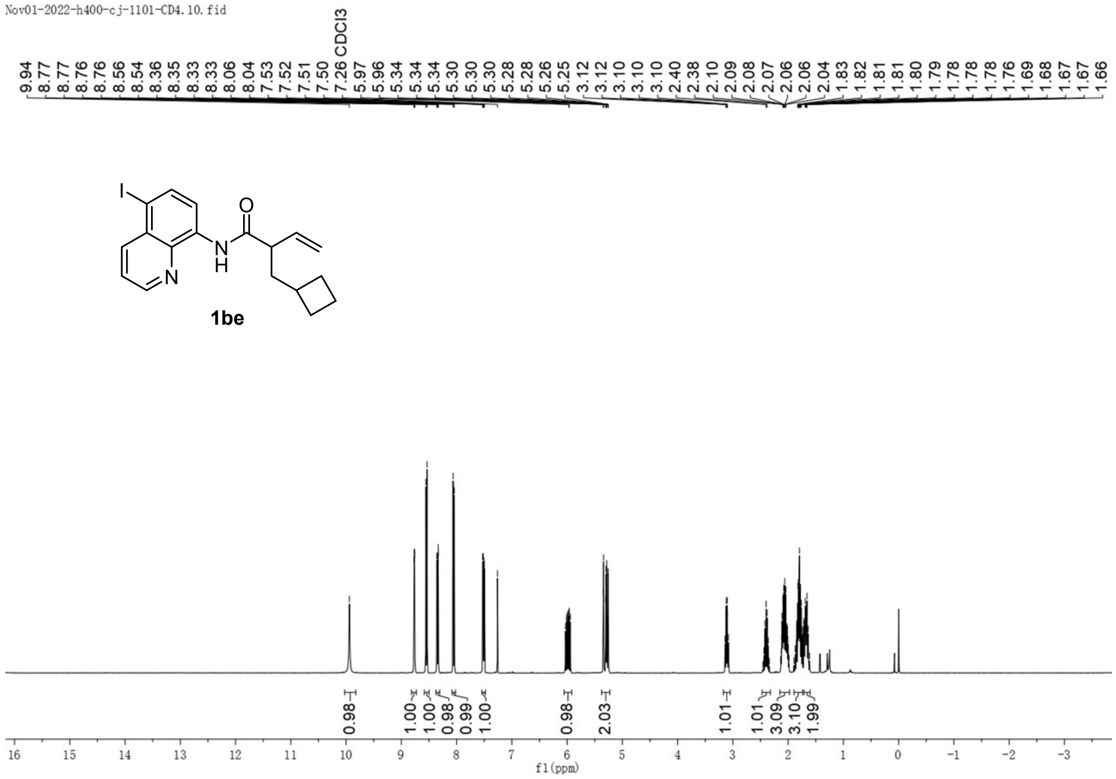

^{13}C NMR of 1be ($CDCl_3$, 101 MHz)

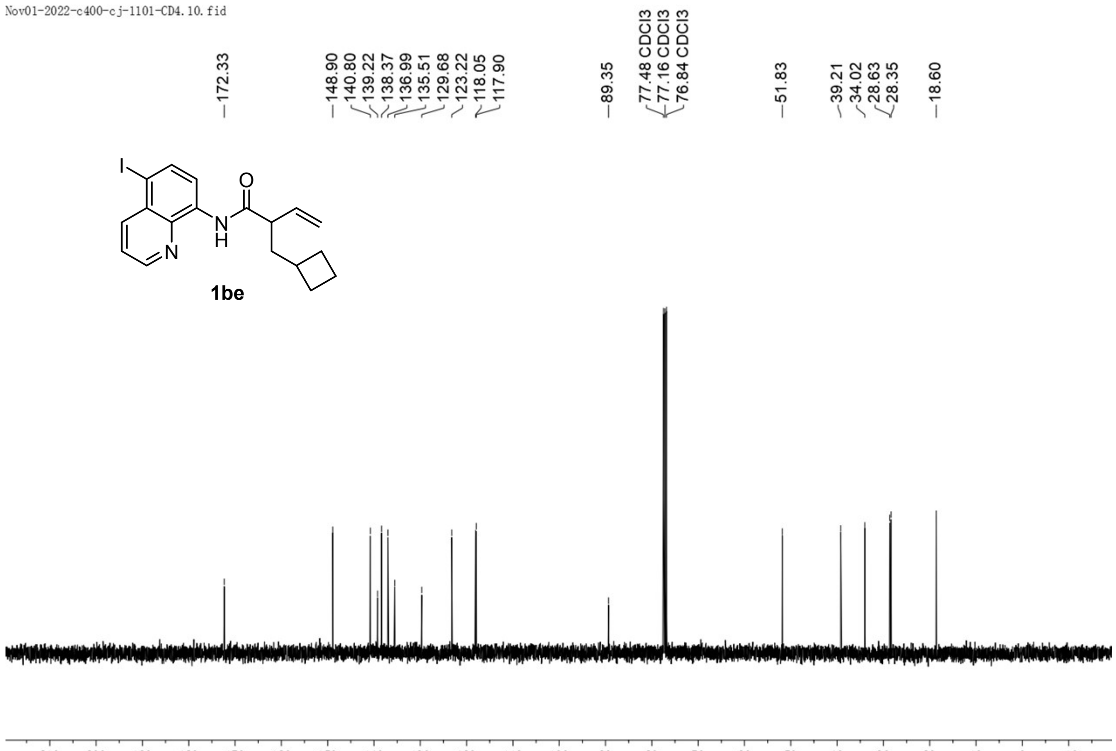

^{1}H NMR of 1bf ($CDCl_3$, 400 MHz)

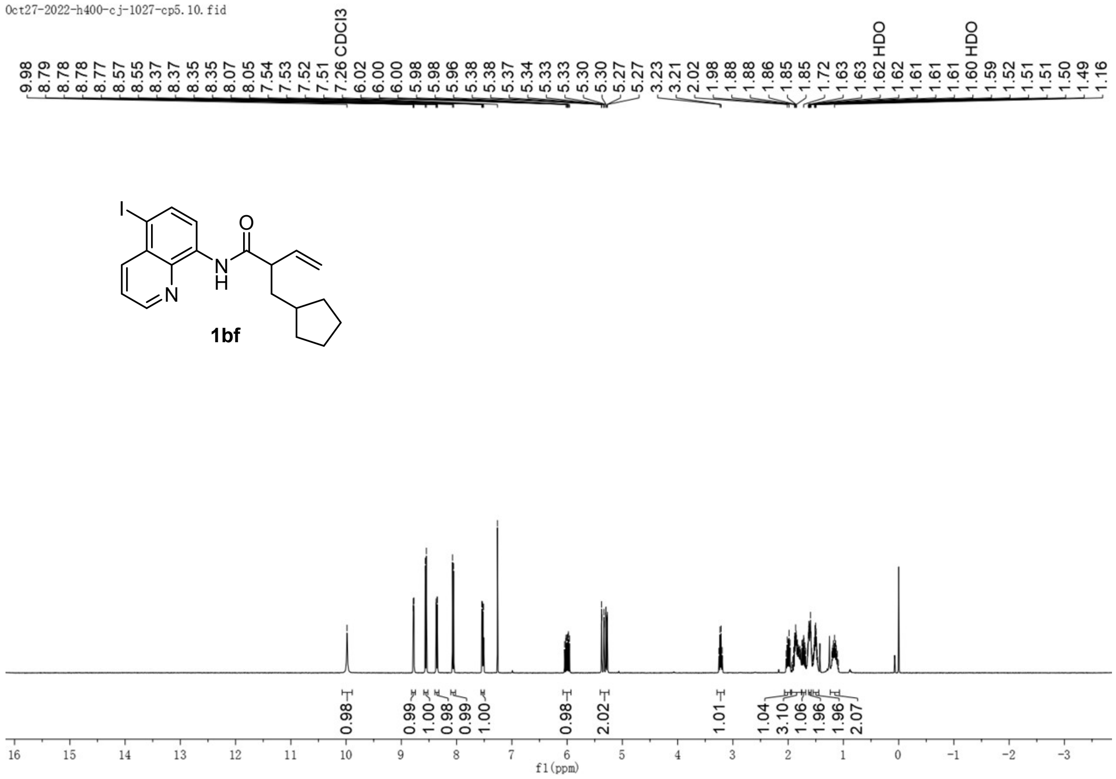

^{13}C NMR of 1bf ($CDCl_3$, 101 MHz)

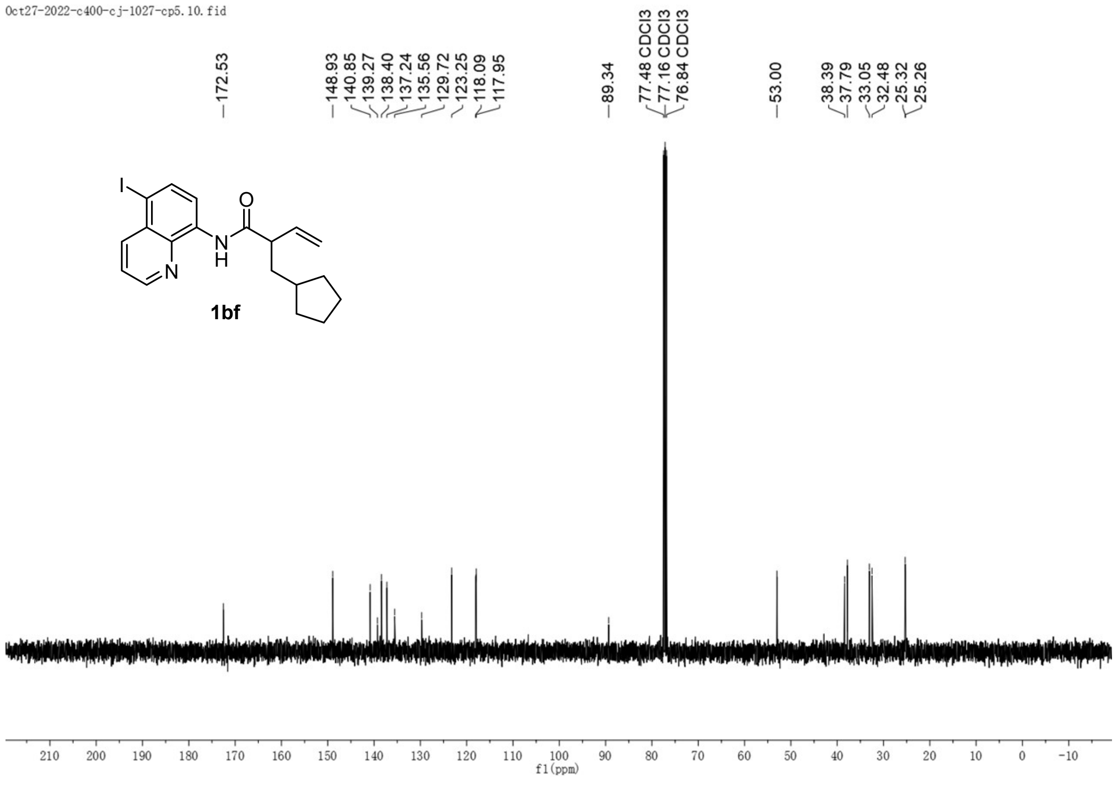

^{1}H NMR of 1bg ($CDCl_3$, 400 MHz)

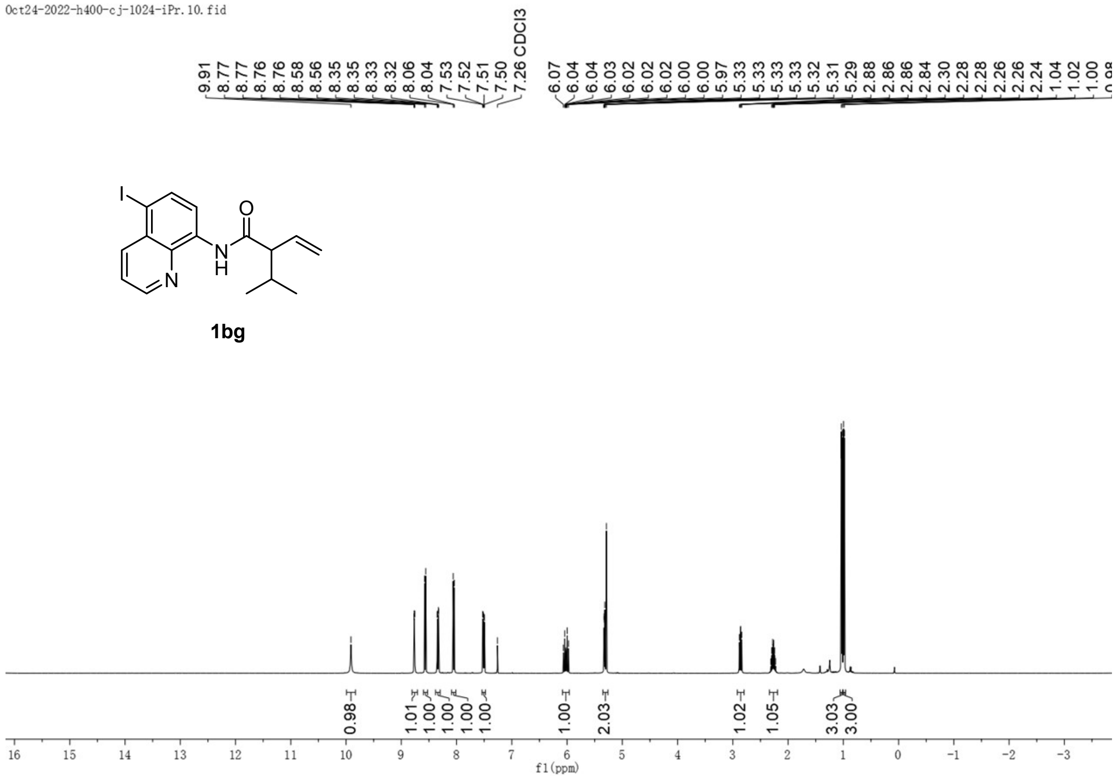

^{13}C NMR of 1bg ($CDCl_3$, 101 MHz)

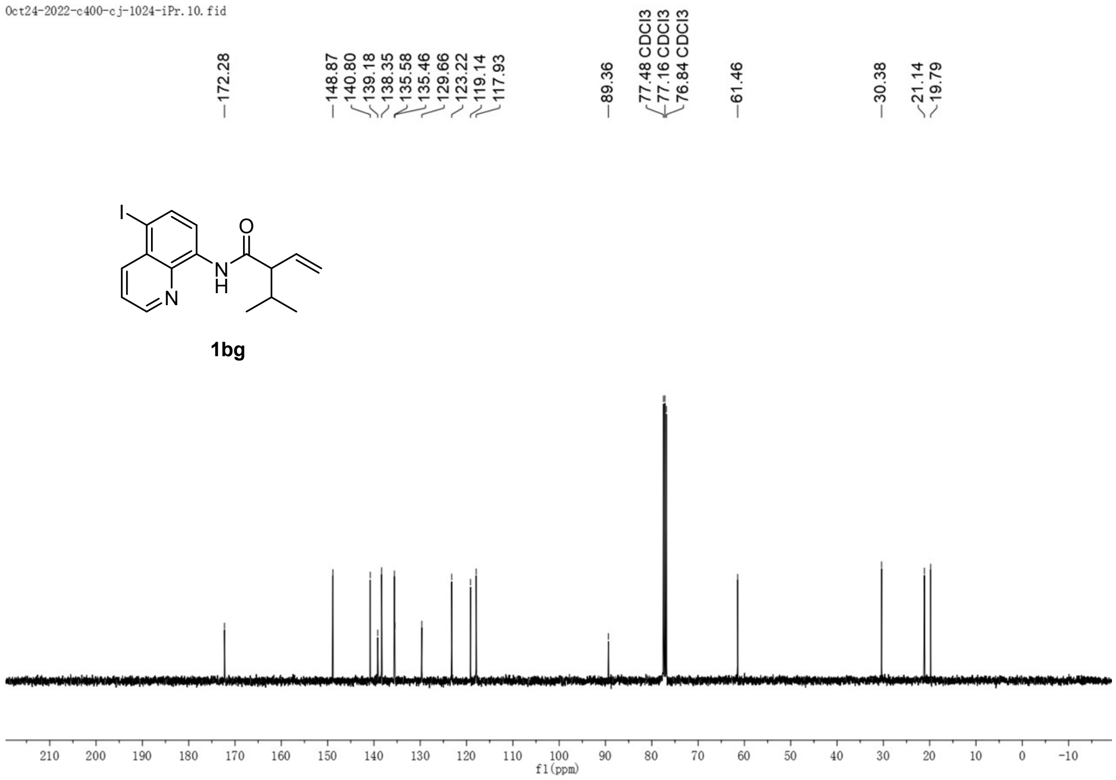

^{1}H NMR of 1bh ($CDCl_3$, 400 MHz)

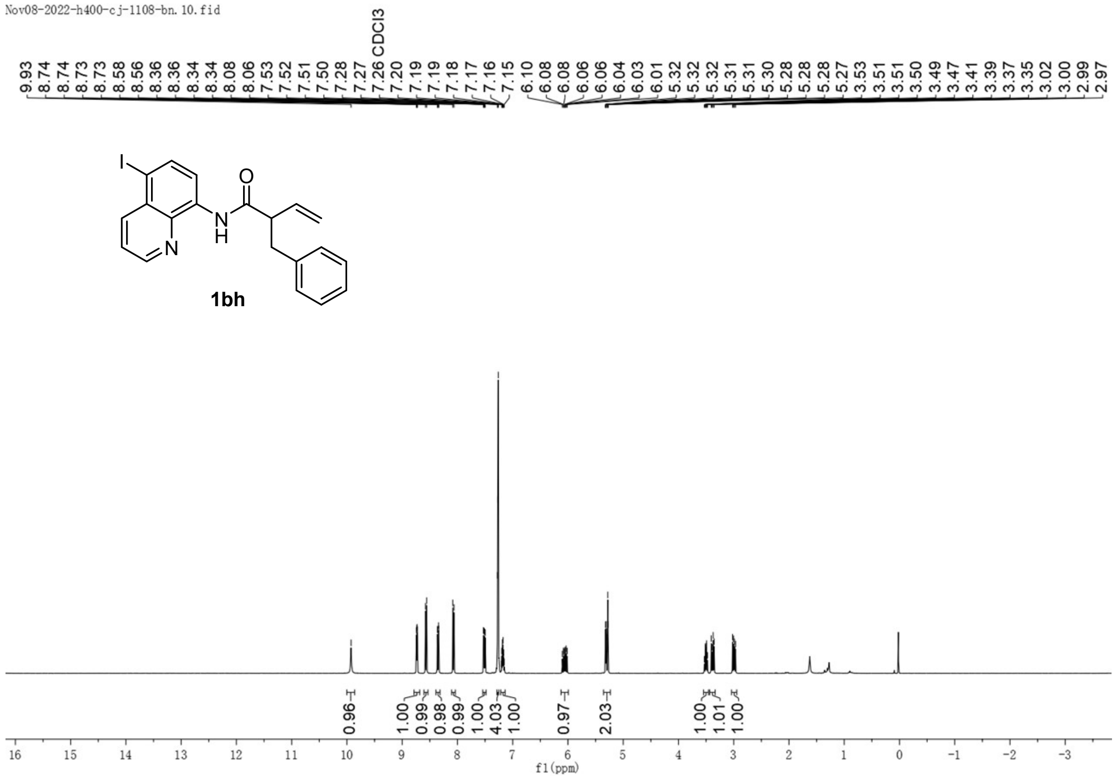

^{13}C NMR of 1bh ($CDCl_3$, 101 MHz)

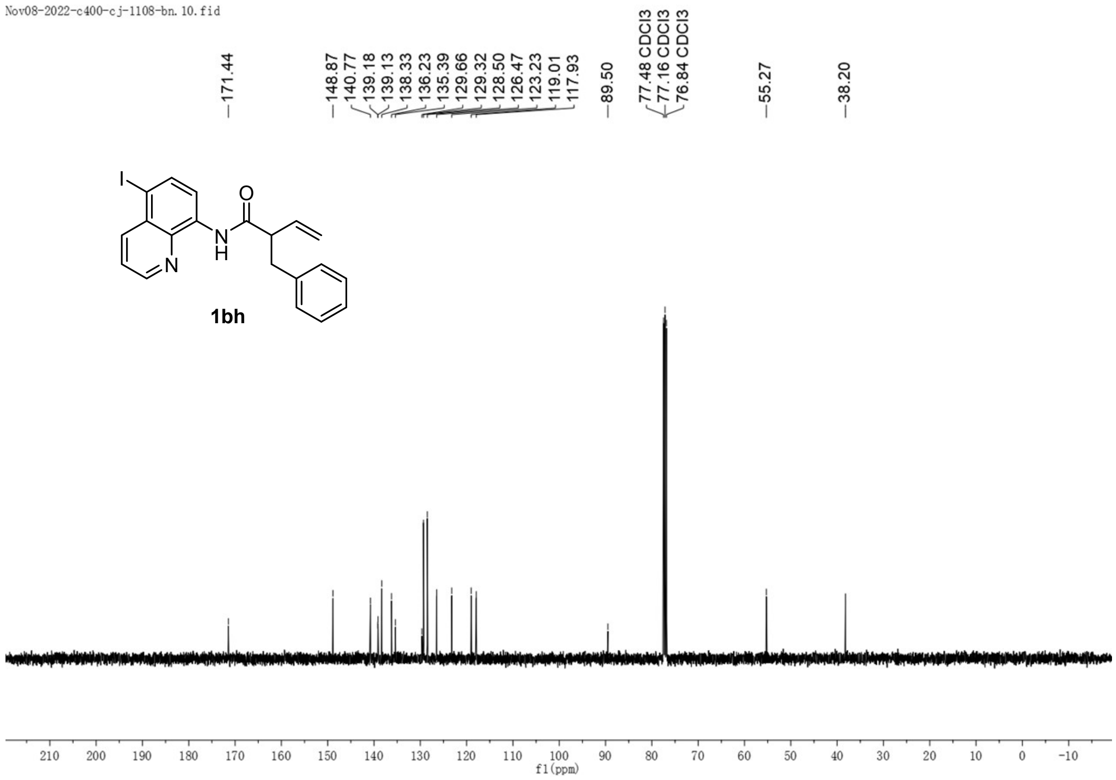

^{1}H NMR of 1bi ($CDCl_3$, 400 MHz)

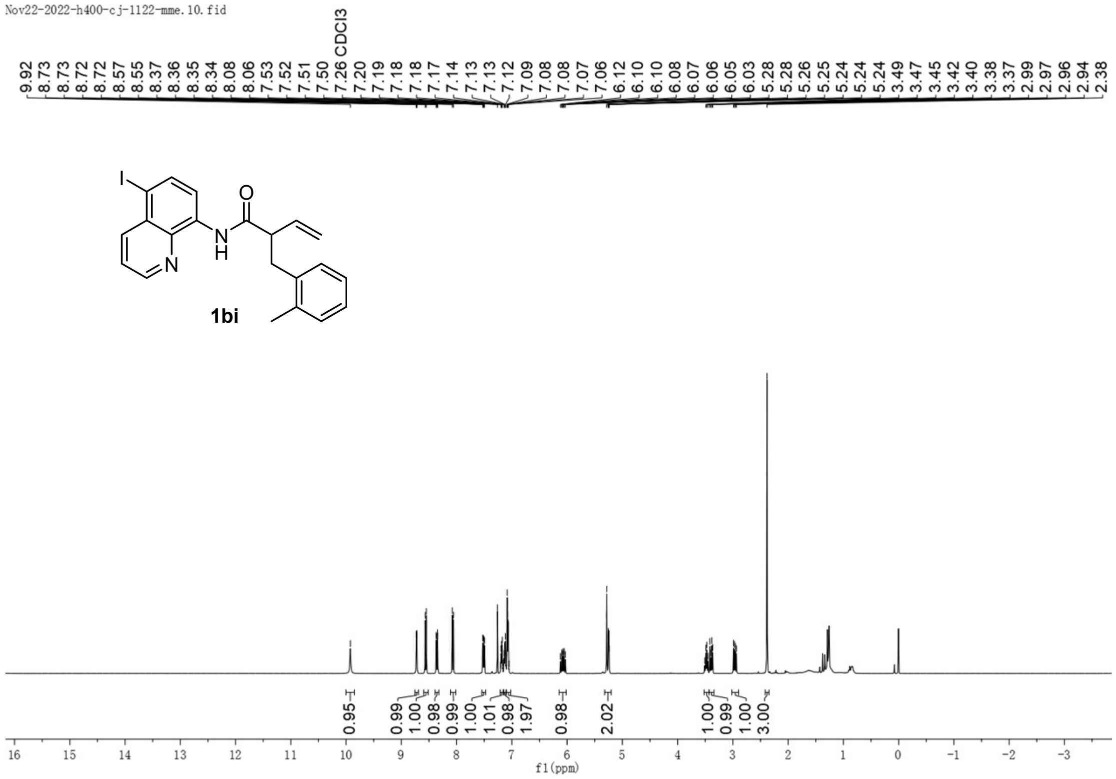

^{13}C NMR of 1bi ($CDCl_3$, 101 MHz)

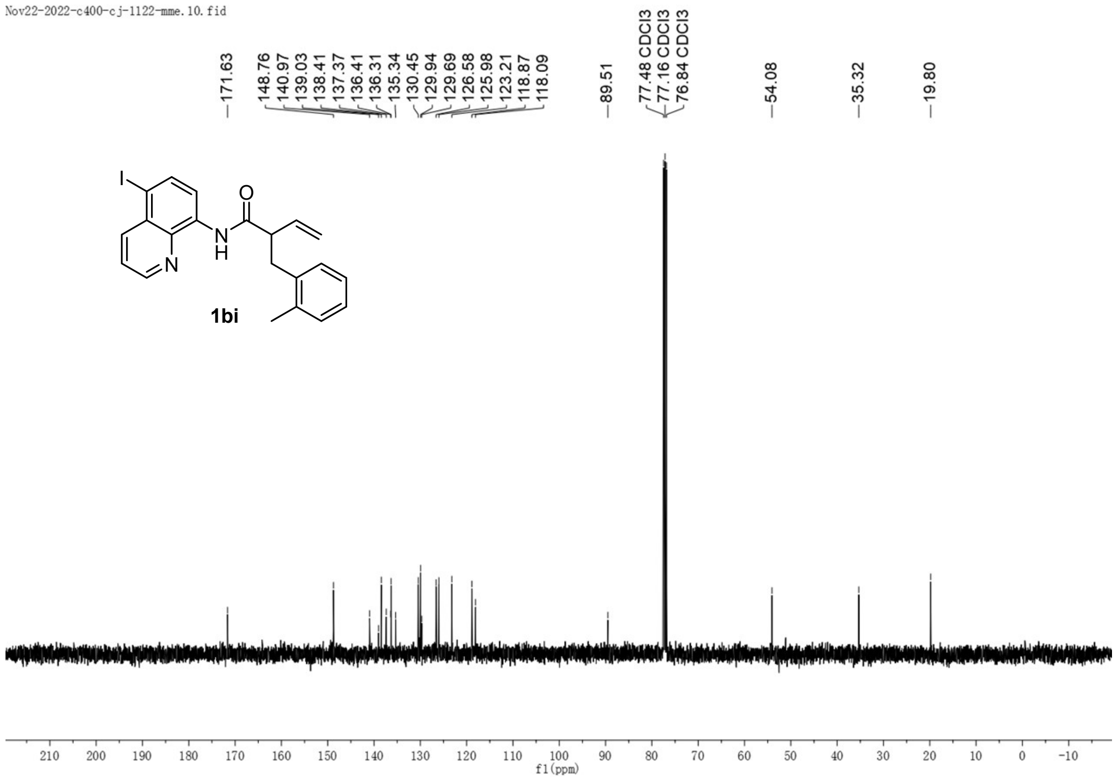

^{1}H NMR of 1bj($CDCl_3$, 400 MHz)

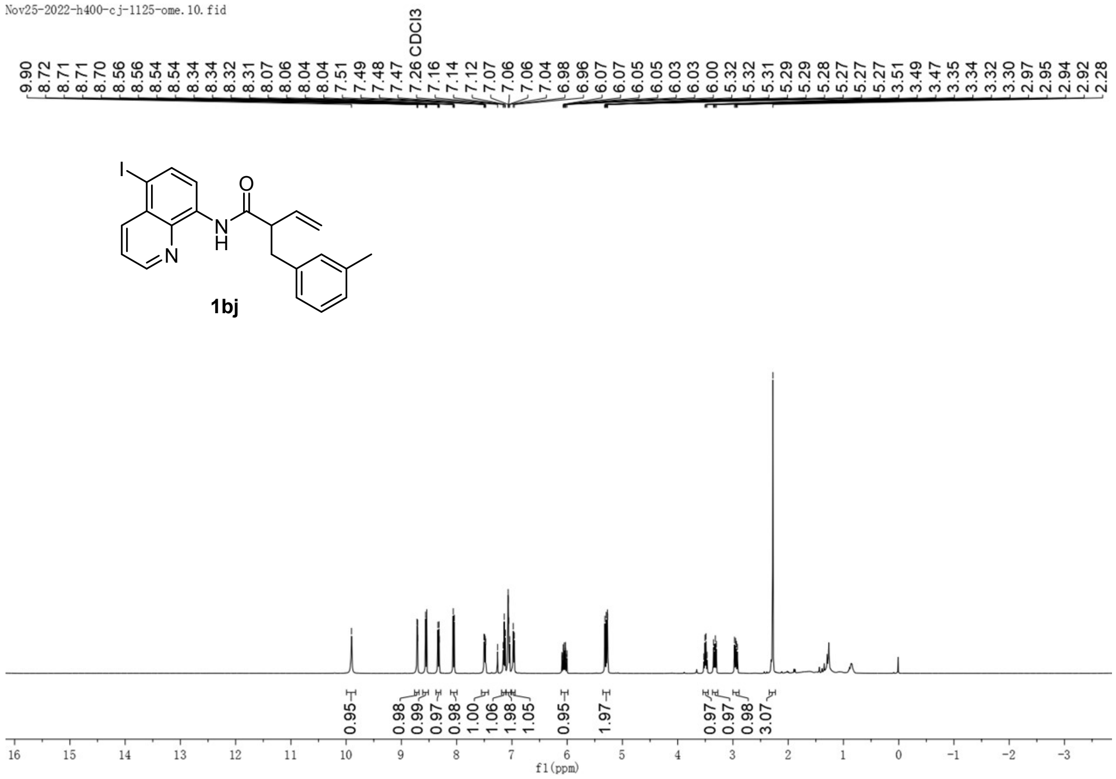

^{13}C NMR of 1bj ($CDCl_3$, 101 MHz)

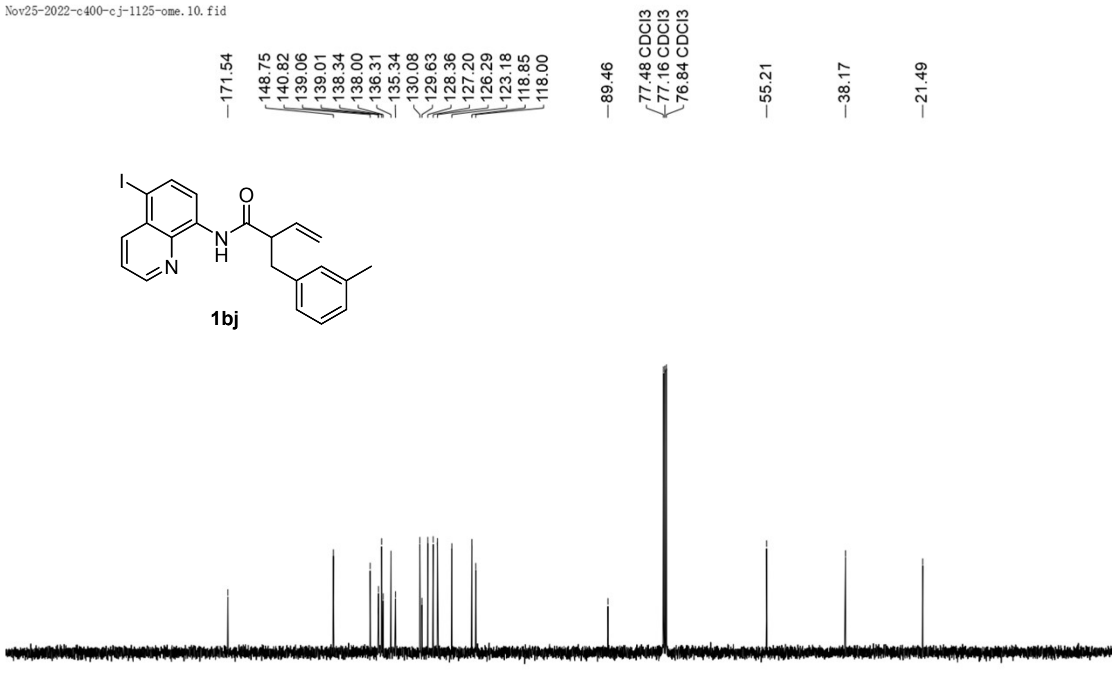

^{1}H NMR of 1bk ($CDCl_3$, 400 MHz)

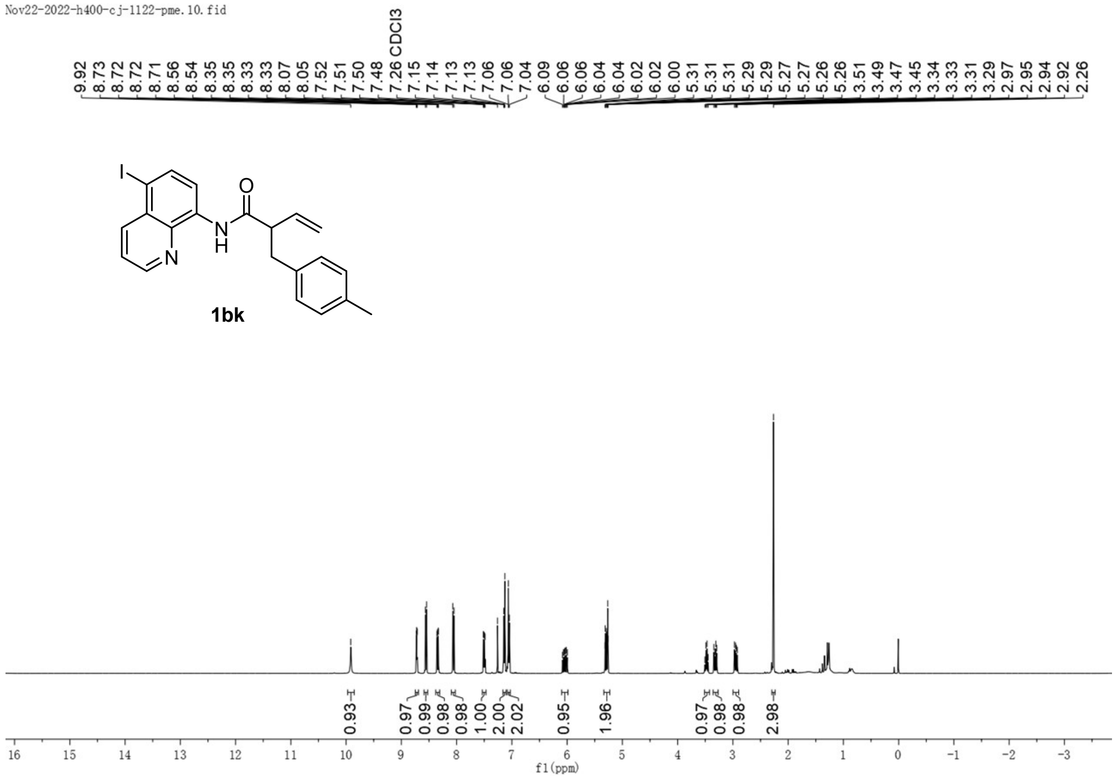

^{13}C NMR of 1bk ($CDCl_3$, 101 MHz)

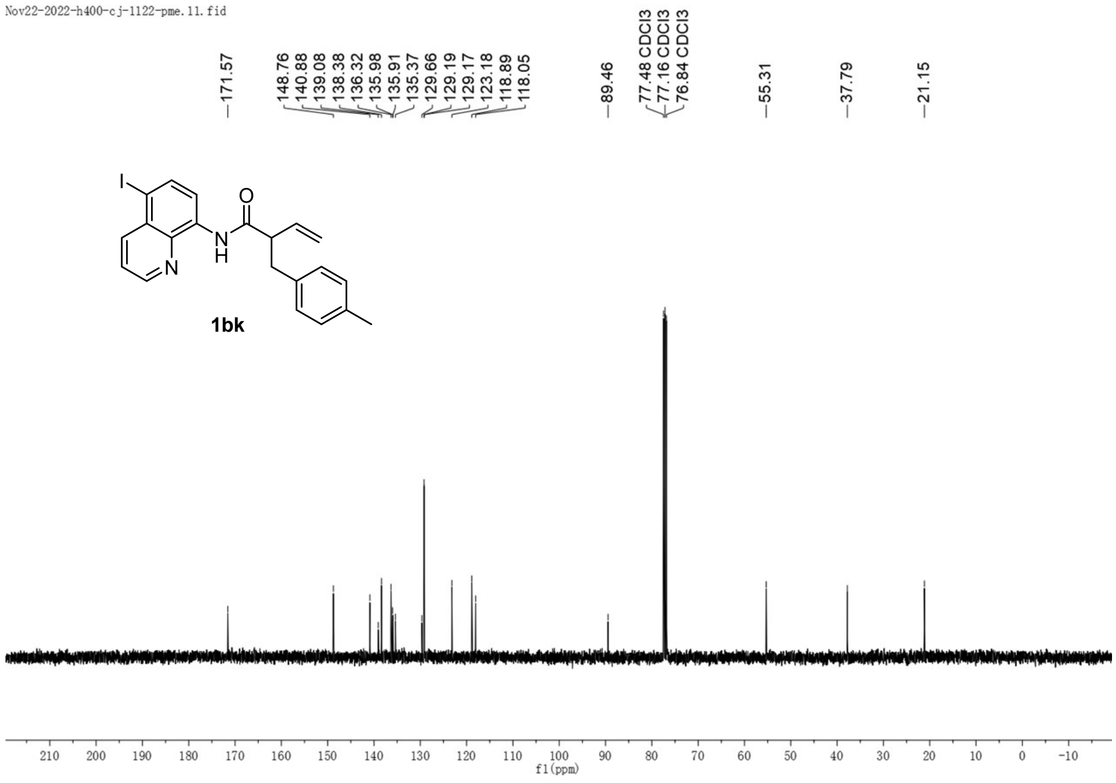

^{1}H NMR of 1bl (CDCl$_3$, 400 MHz)

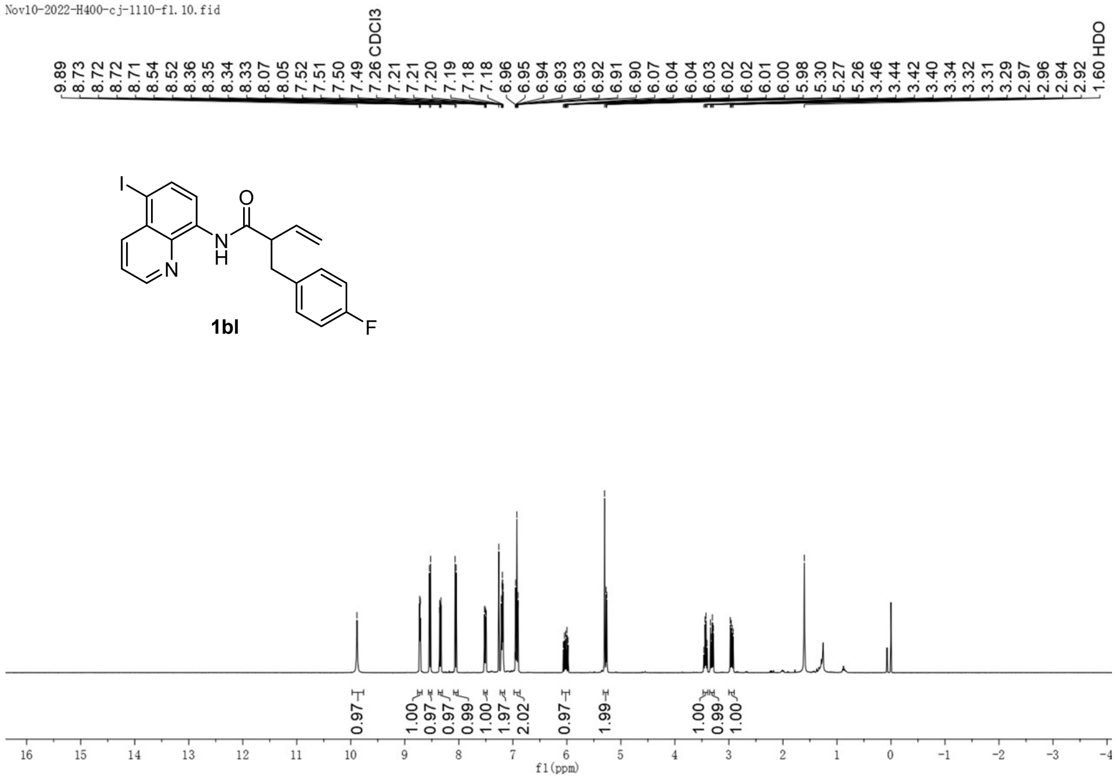

^{13}C NMR of 1bl (CDCl$_3$, 101 MHz)

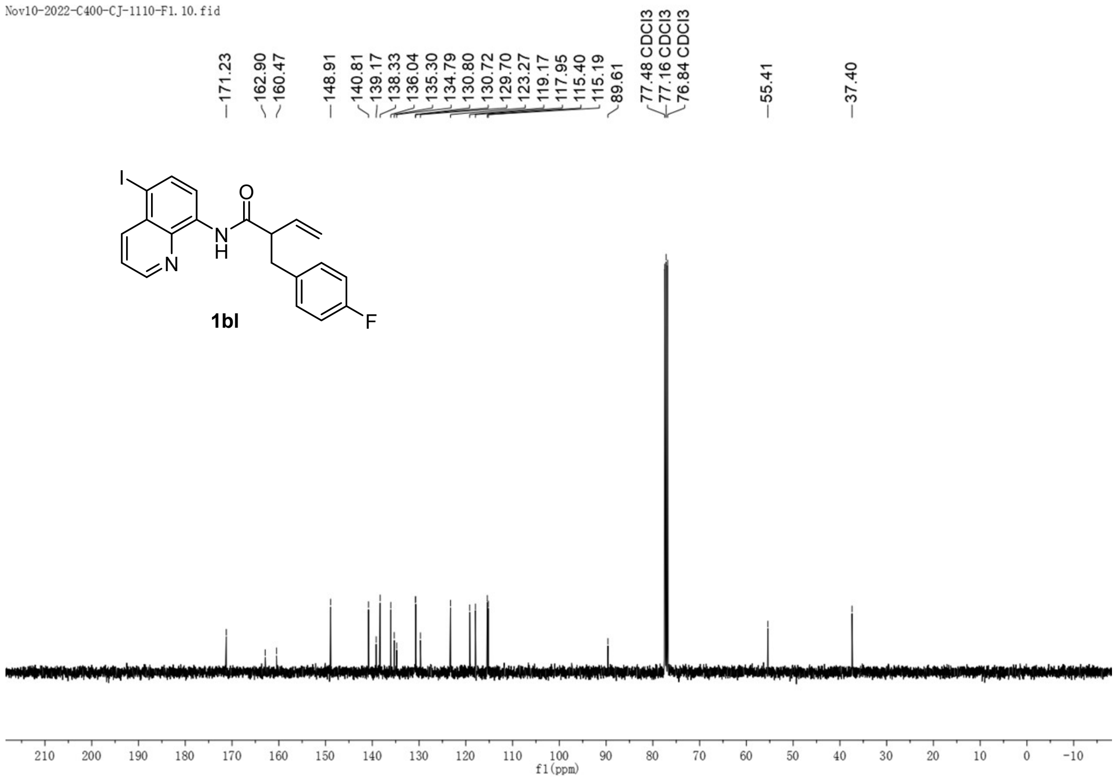

^{19}F NMR of 1bl ($CDCl_3$, 376 MHz)

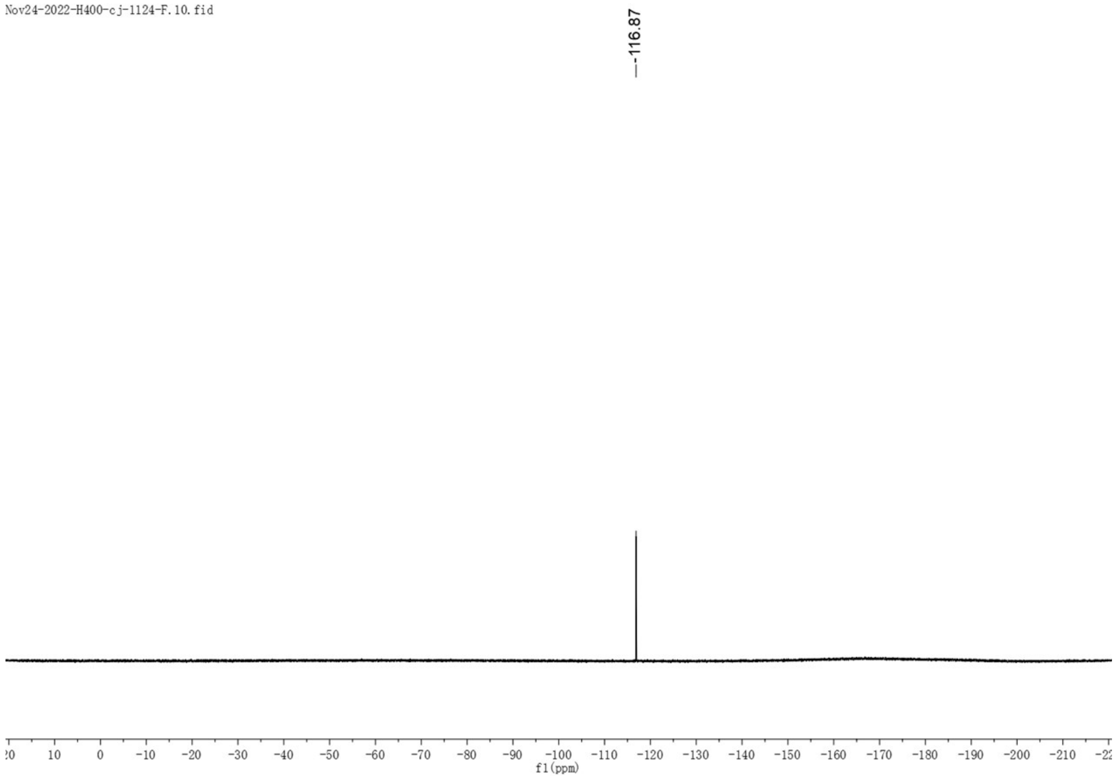

^{1}H NMR of 1bm ($CDCl_3$, 400 MHz)

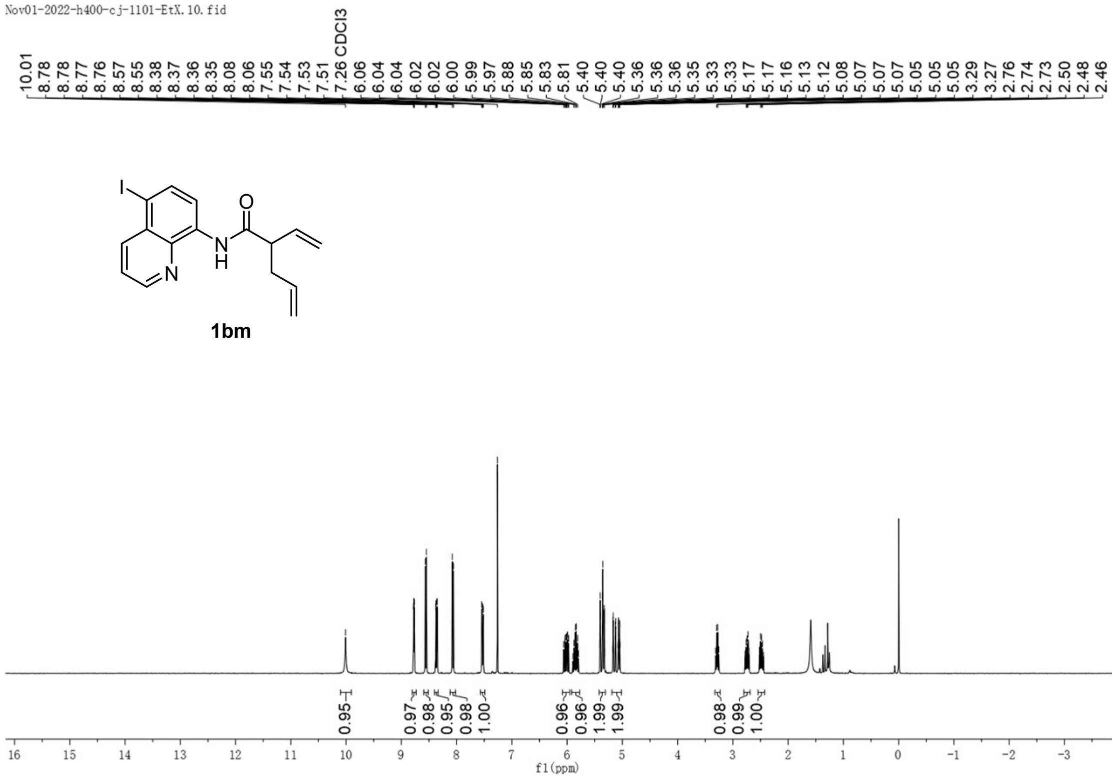

^{13}C NMR of 1bm ($CDCl_3$, 101 MHz)

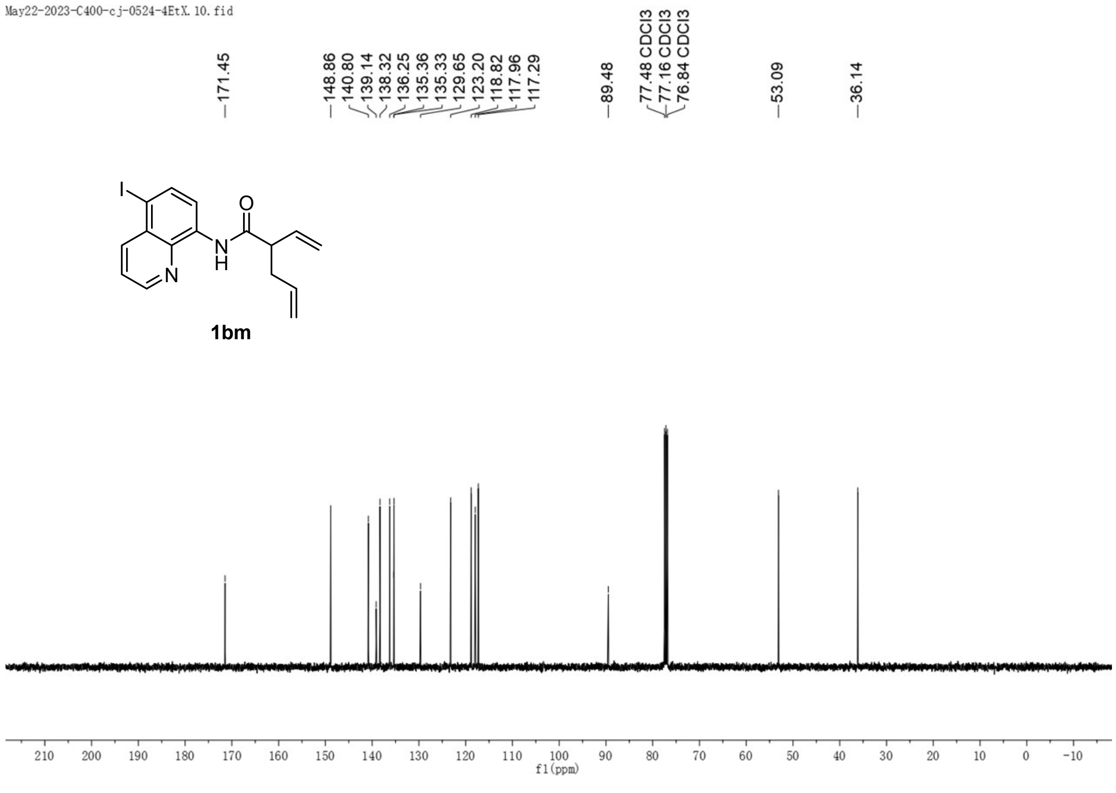

^{1}H NMR of 1bn ($CDCl_3$, 400 MHz)

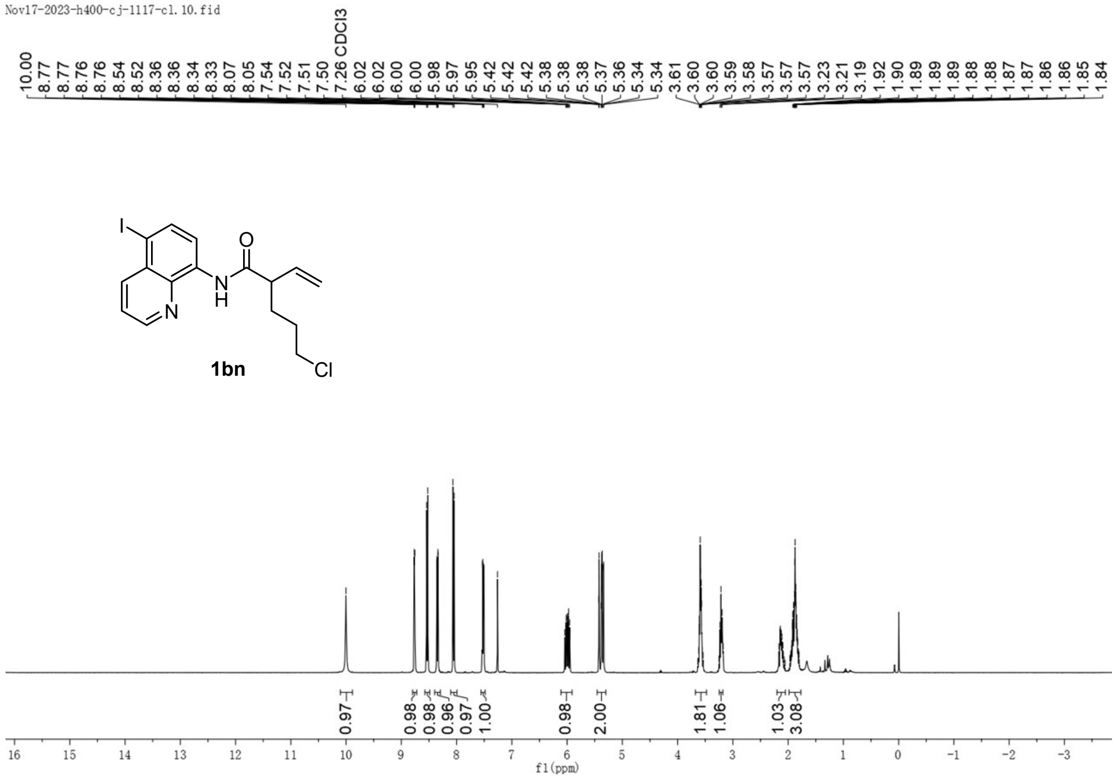

^{13}C NMR of 1bn ($CDCl_3$, 101 MHz)

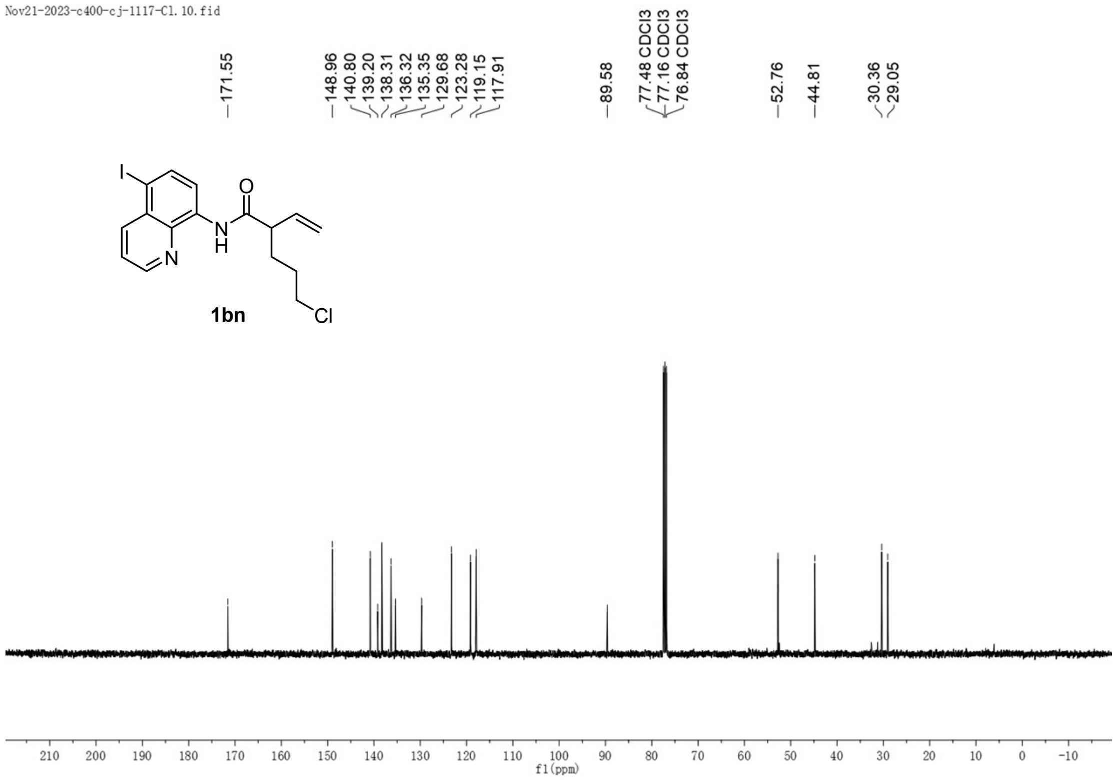

^{1}H NMR of 1bo ($CDCl_3$, 400 MHz)

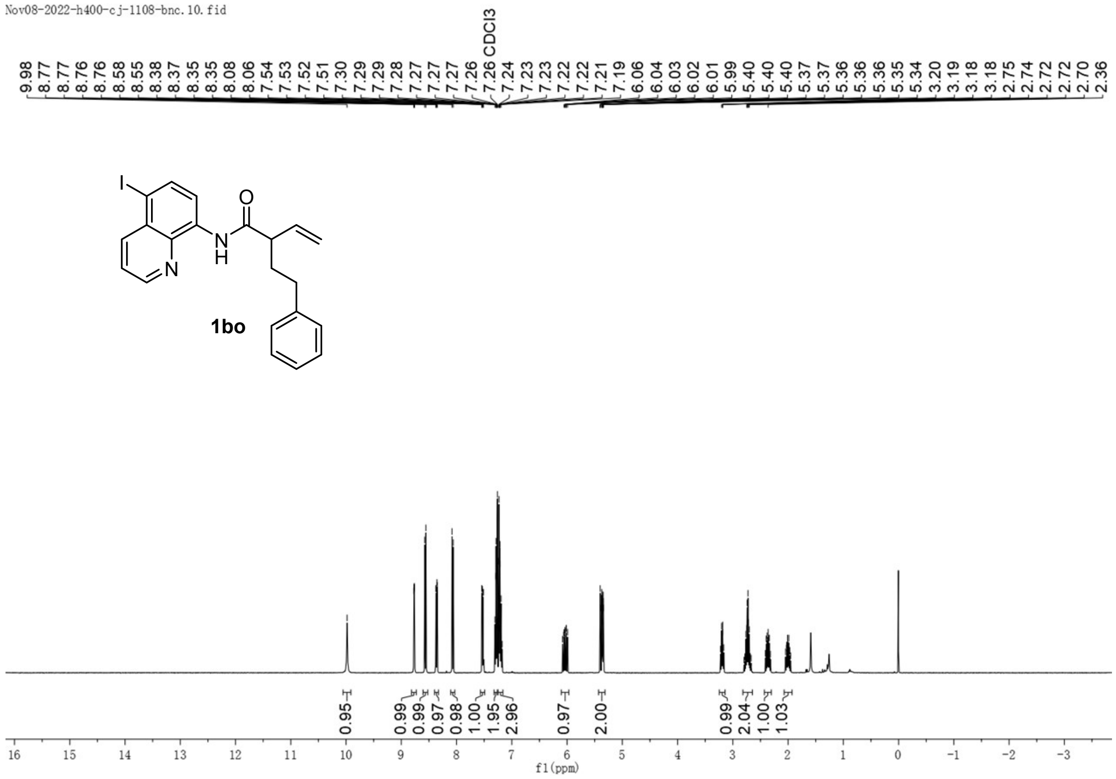

^{13}C NMR of 1bo ($CDCl_3$, 101 MHz)

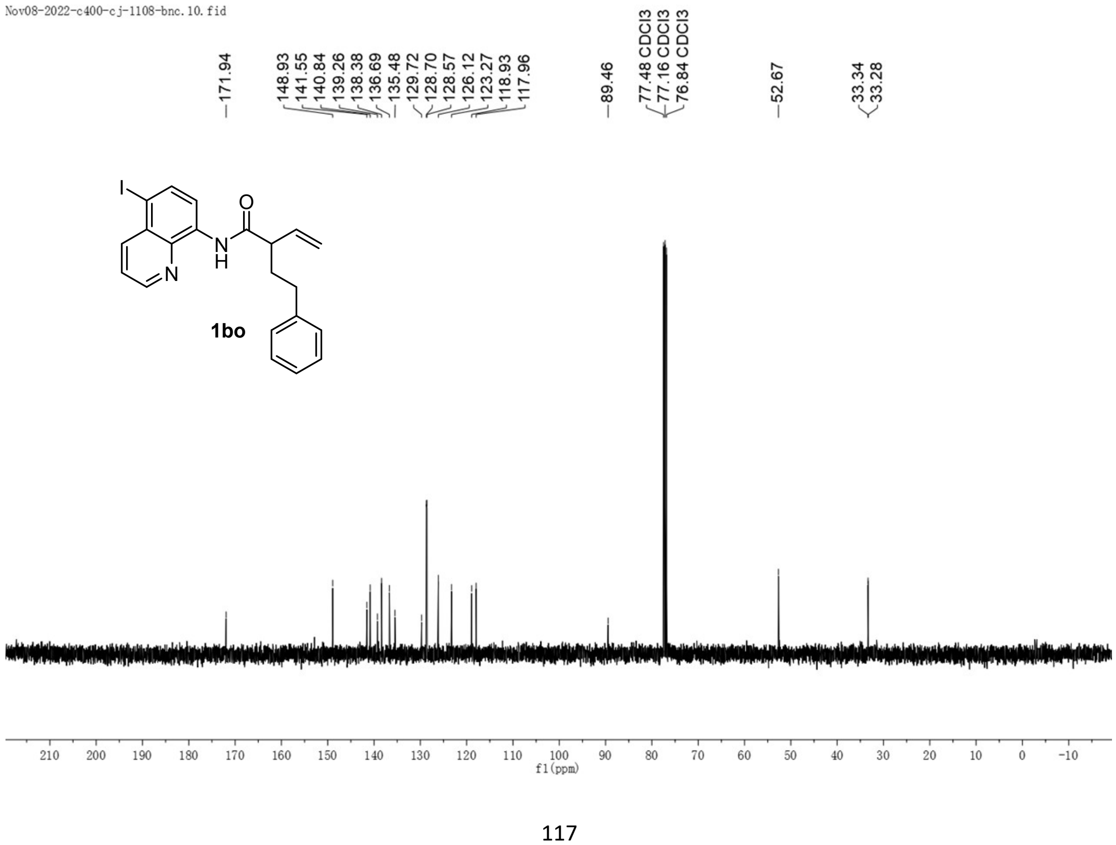

^{1}H NMR of 1bp ($CDCl_3$, 400 MHz)

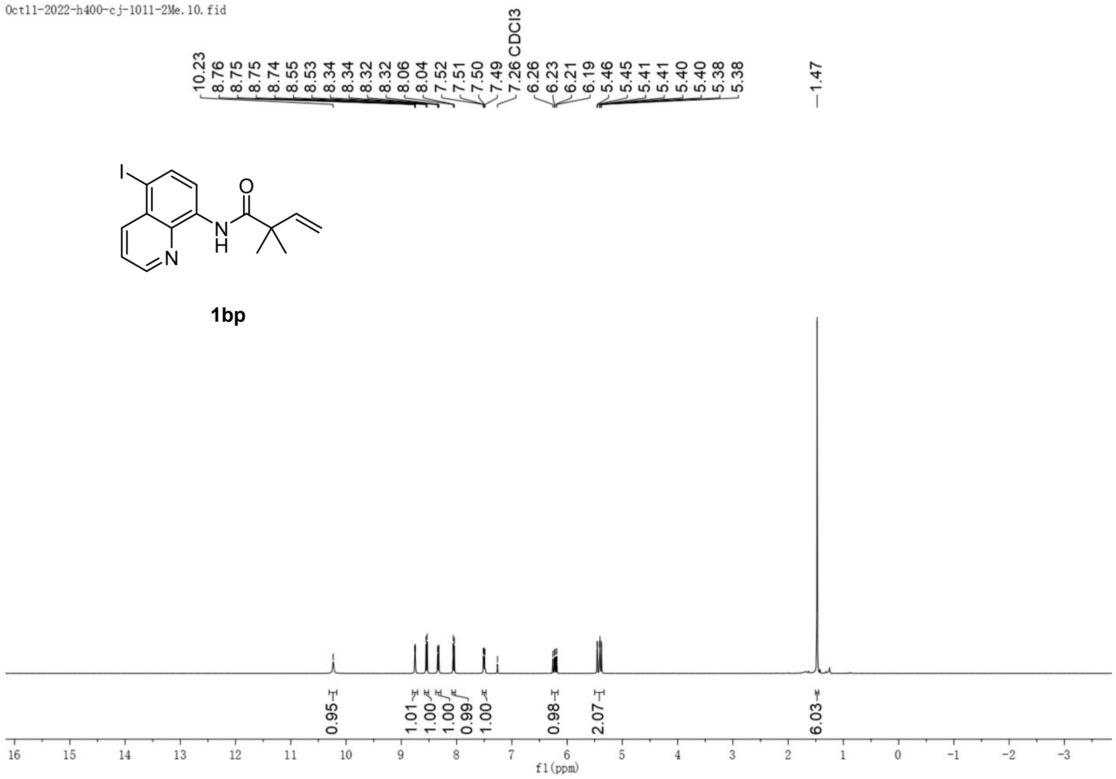

^{13}C NMR of 1bp ($CDCl_3$, 101 MHz)

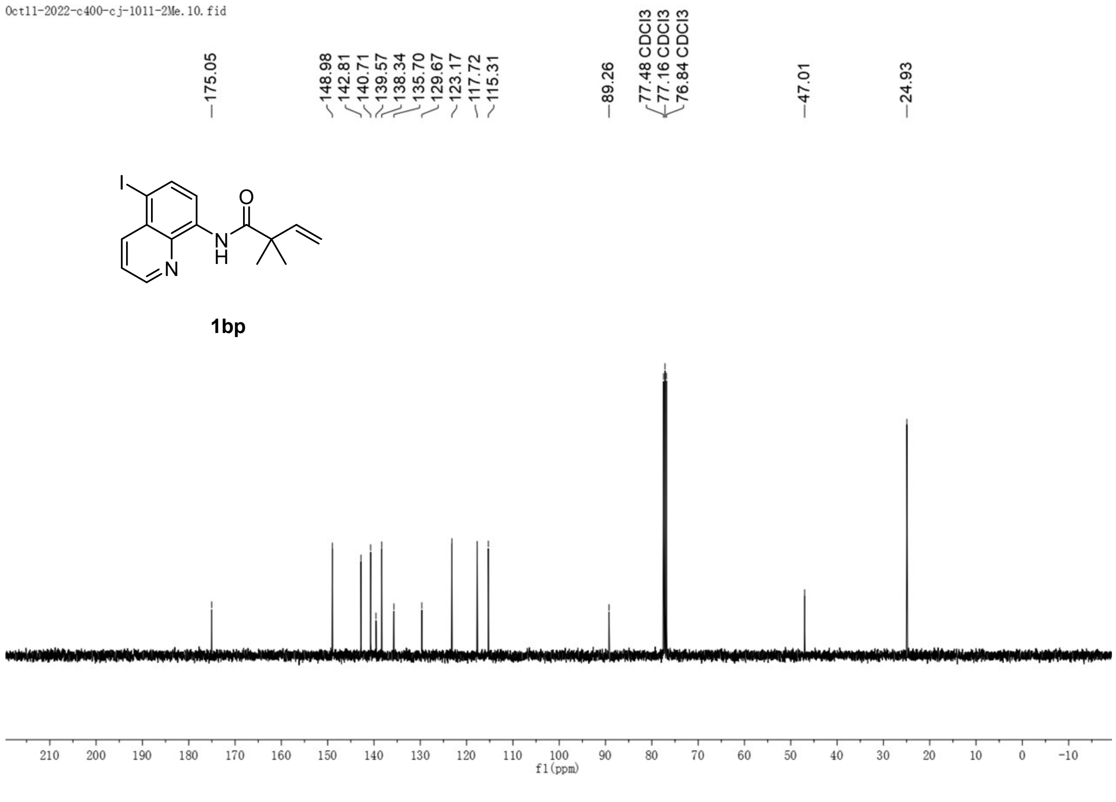

^{1}H NMR of 1bq ($CDCl_3$, 400 MHz)

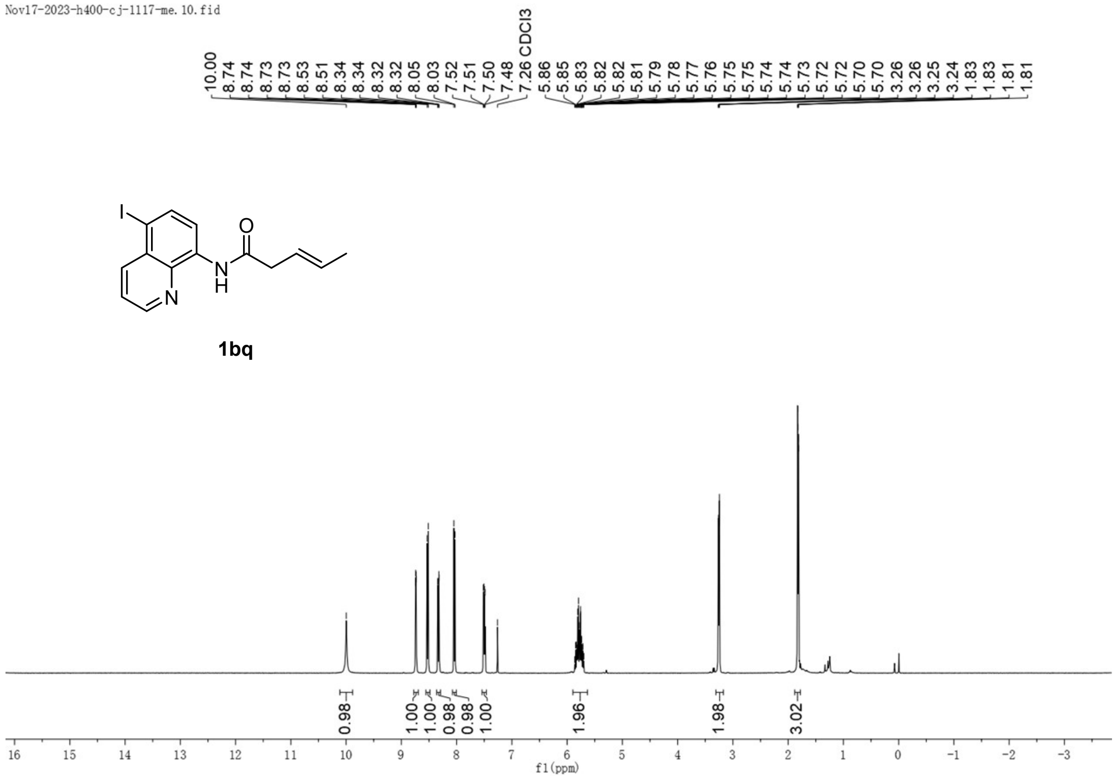

^{13}C NMR of 1bq ($CDCl_3$, 101 MHz)

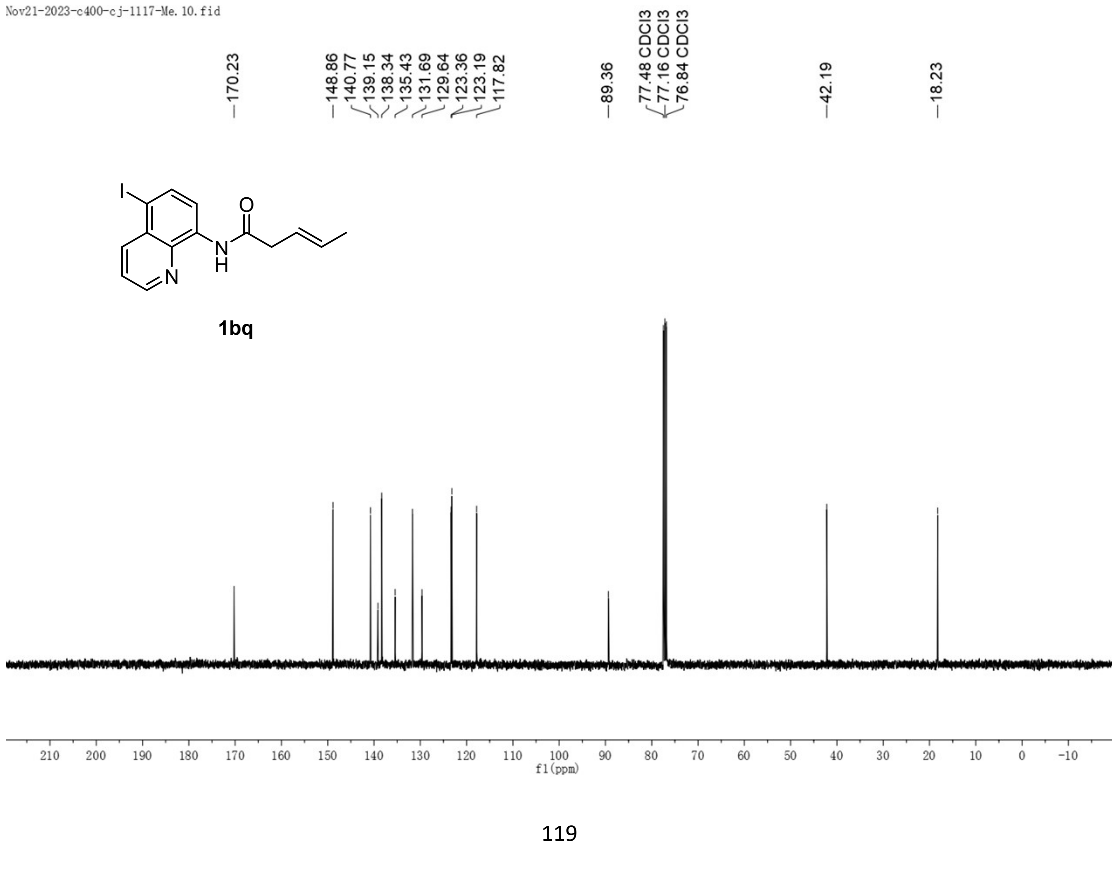

^{1}H NMR of 1br ($CDCl_3$, 400 MHz)

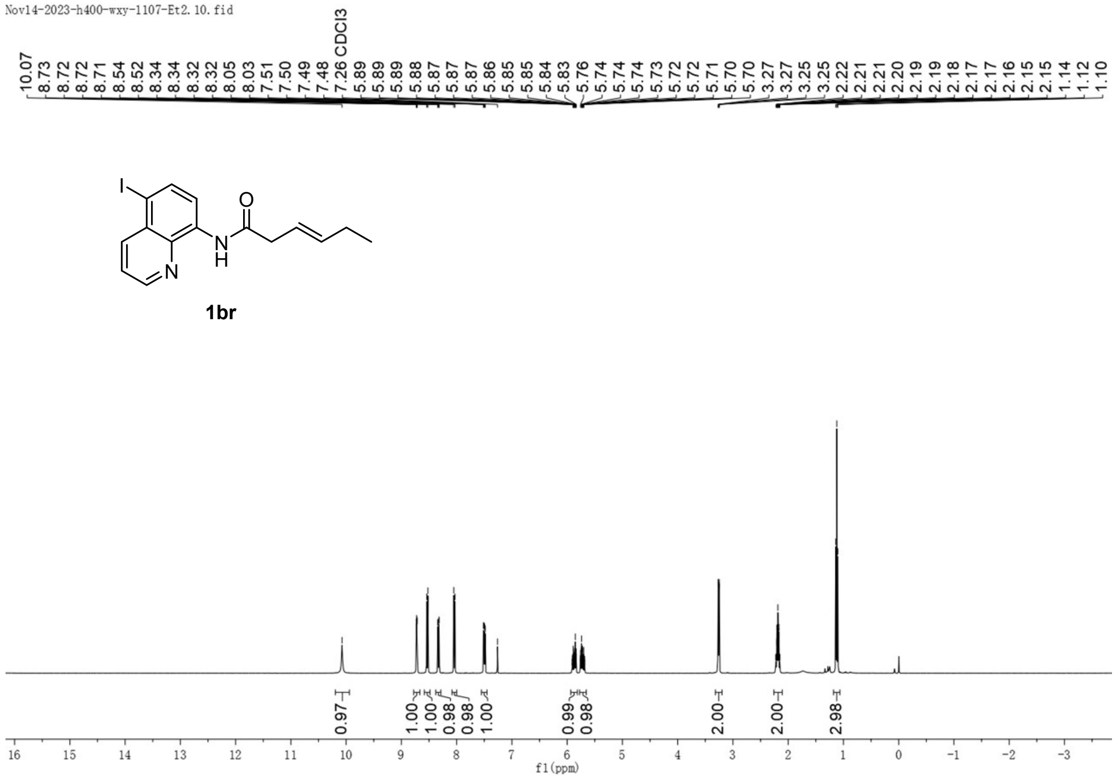

^{13}C NMR of 1br ($CDCl_3$, 101 MHz)

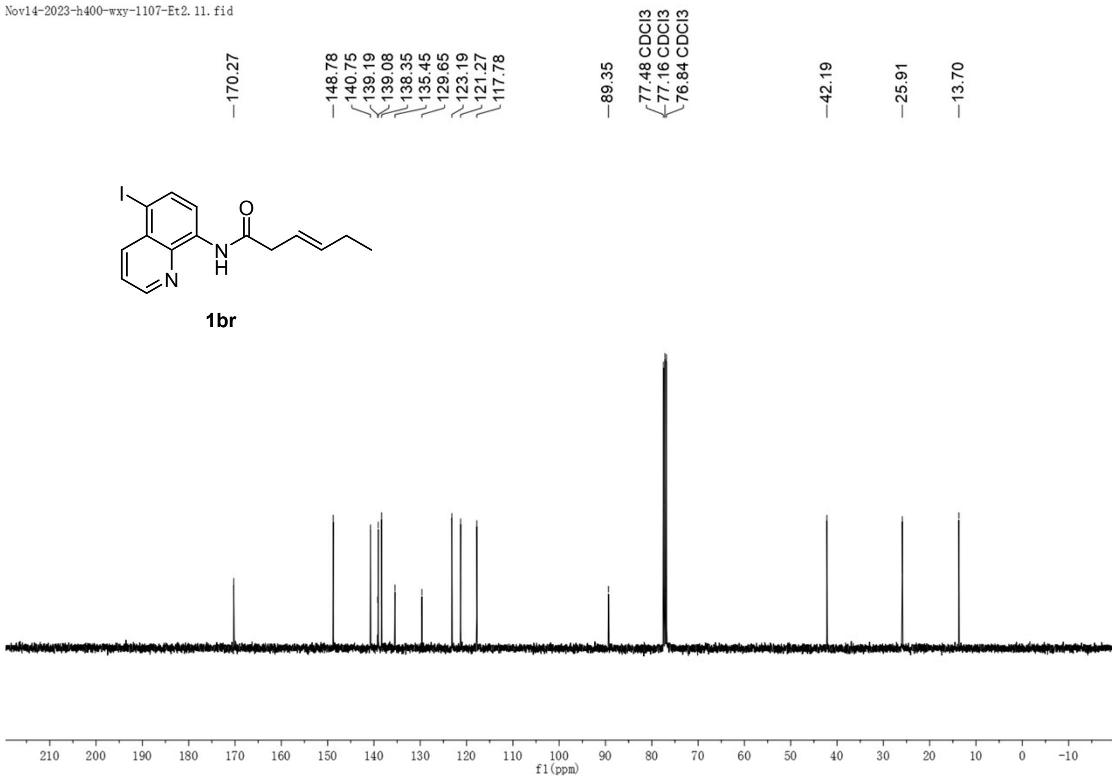

^{1}H NMR of 3'-AQ ($CDCl_3$, 400 MHz)

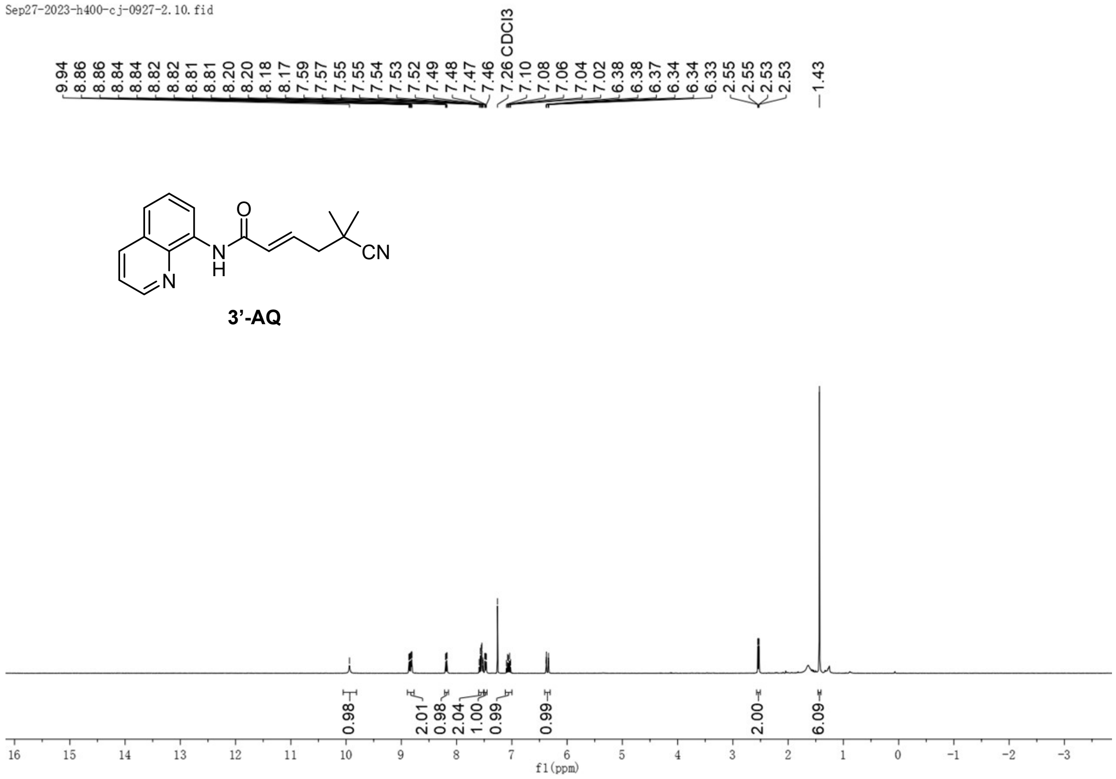

^{13}C NMR of 3'-AQ ($CDCl_3$, 101 MHz)

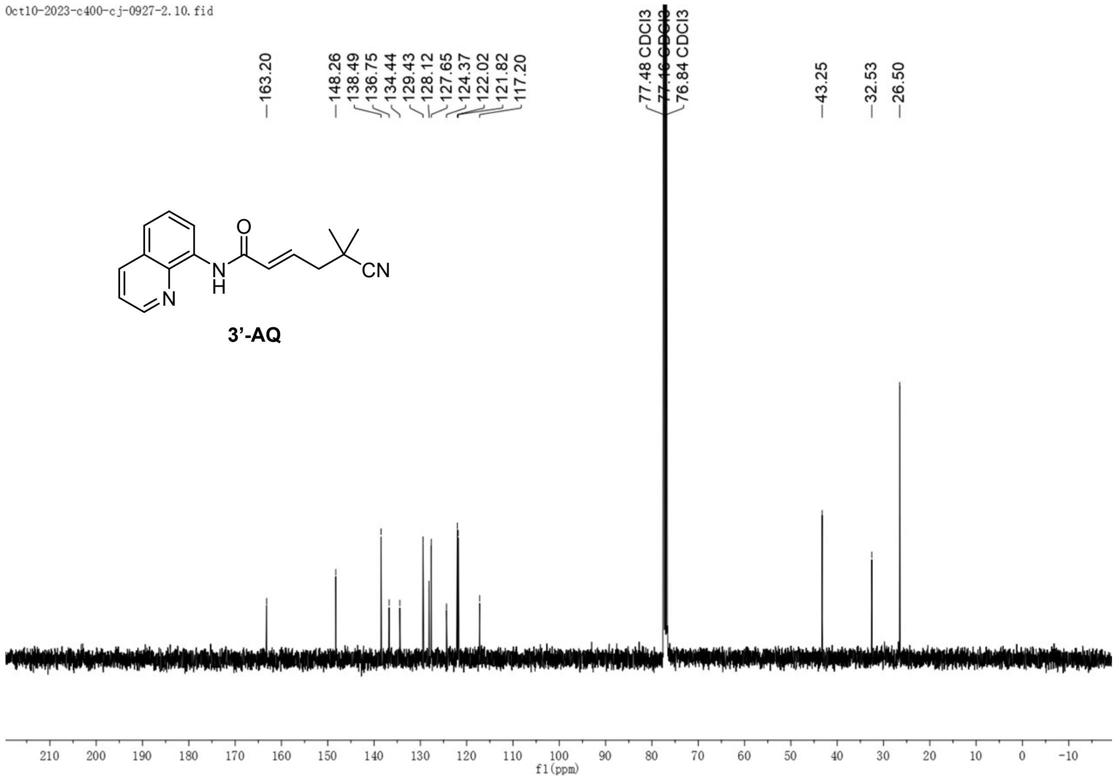

^{1}H NMR of 3aa ($CDCl_3$, 400 MHz)

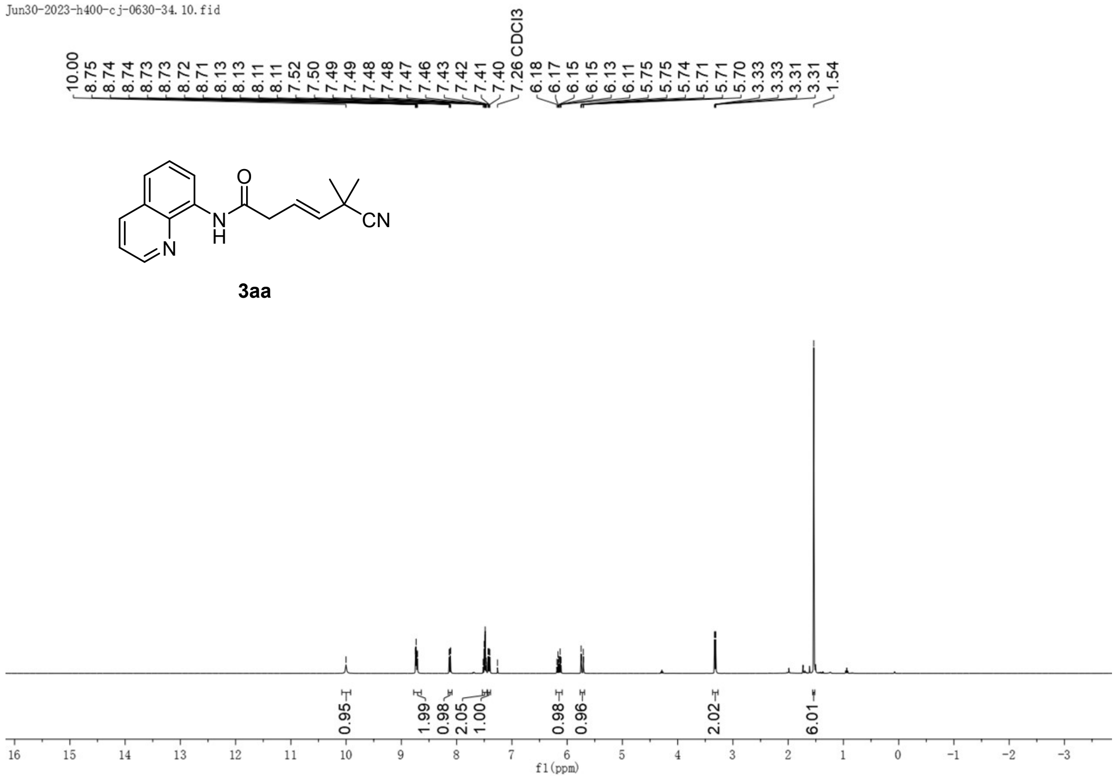

3aa

^{13}C NMR of 3aa ($CDCl_3$, 101 MHz)

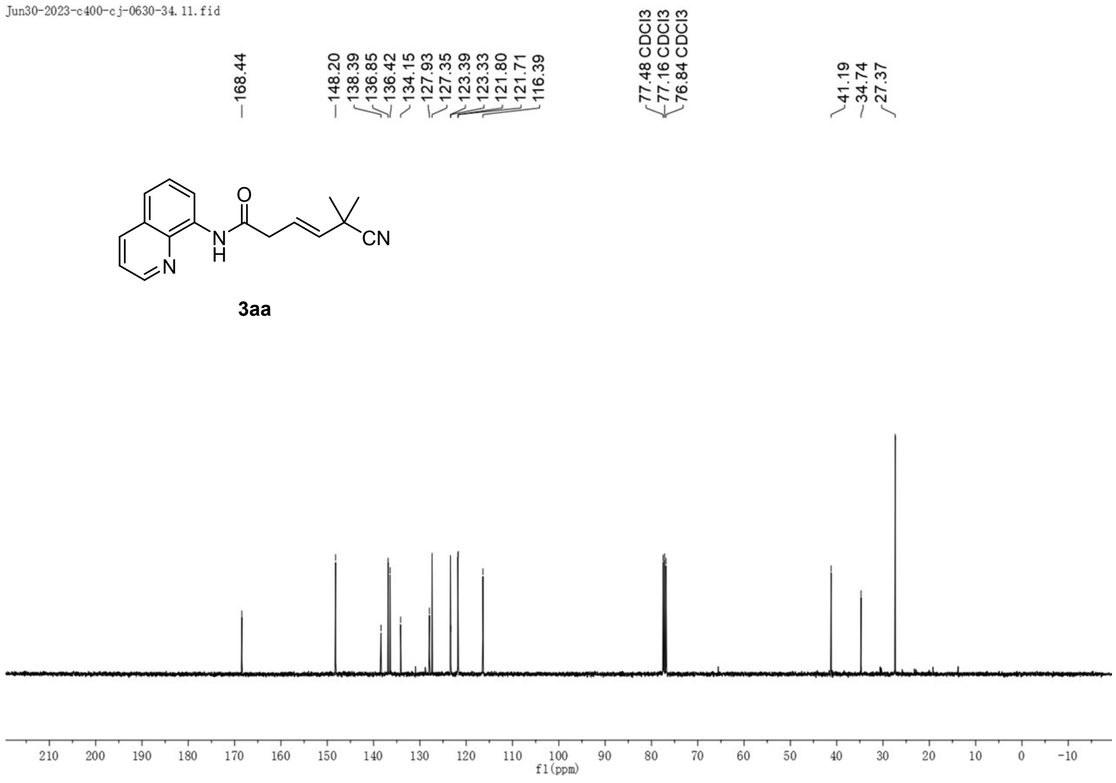

3aa

^{1}H NMR of 3ab ($CDCl_3$, 400 MHz)

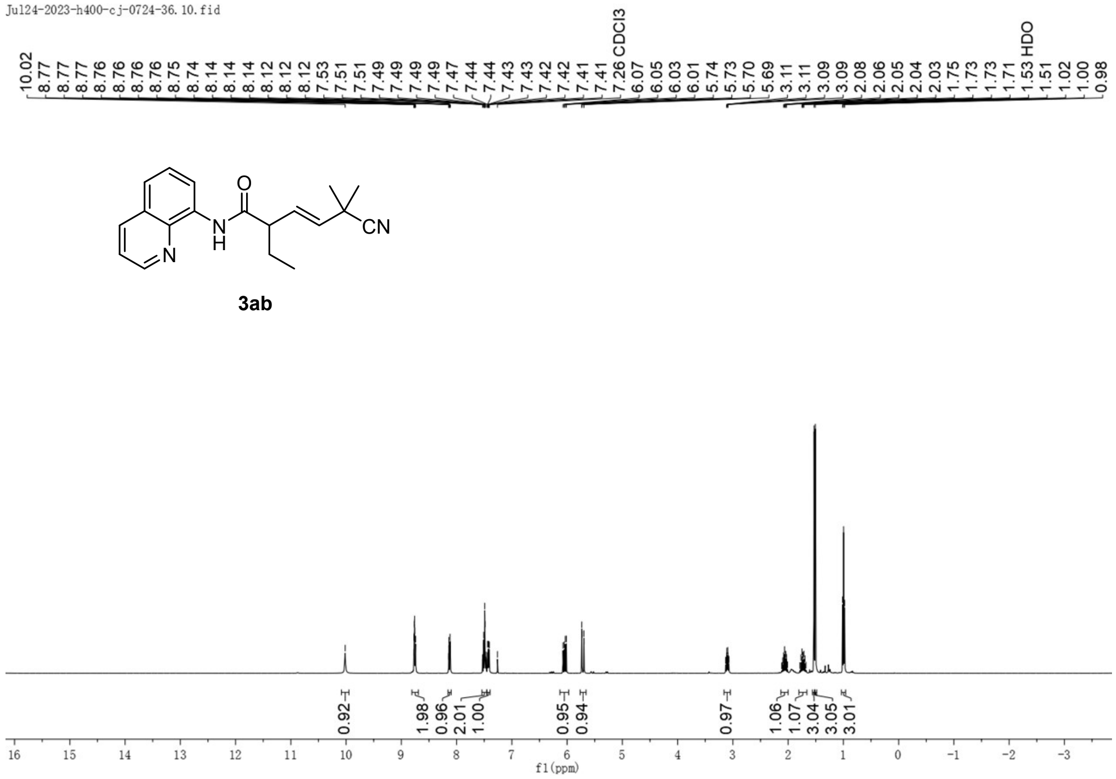

^{13}C NMR of 3ab ($CDCl_3$, 101 MHz)

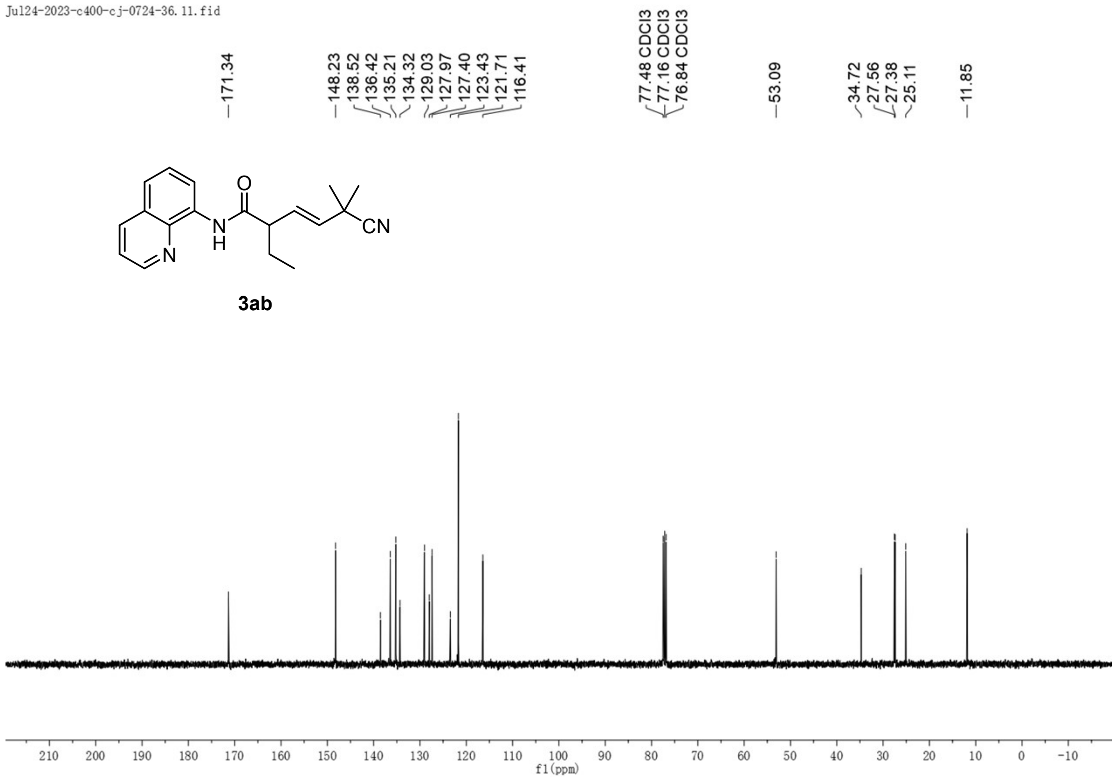

^{1}H NMR of 3ac ($CDCl_3$, 400 MHz)

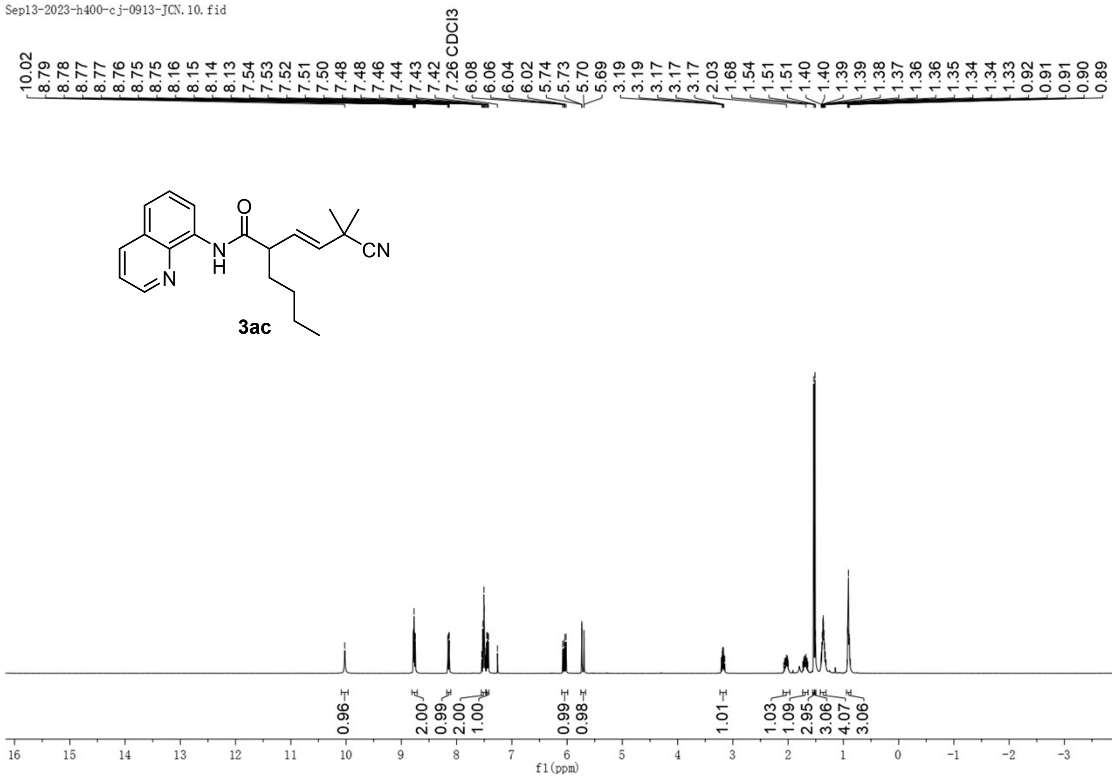

^{13}C NMR of 3ac ($CDCl_3$, 101 MHz)

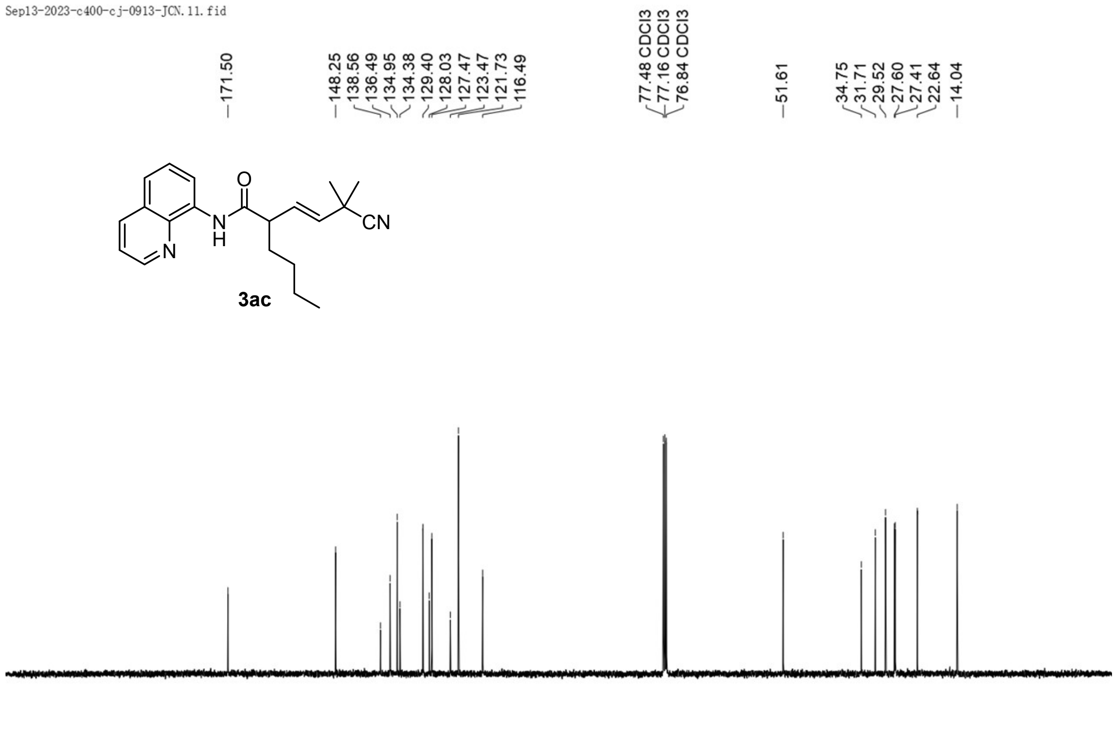

¹H NMR of 3ad ($CDCl_3$, 400 MHz)

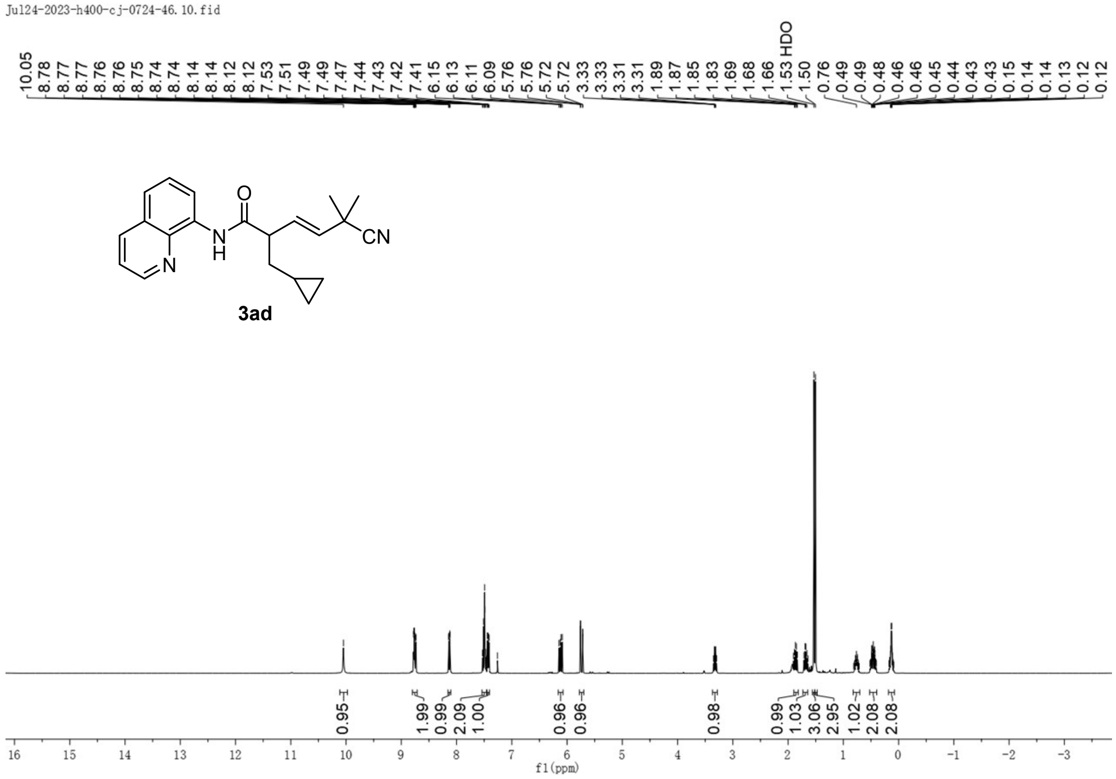

¹³C NMR of 3ad ($CDCl_3$, 101 MHz)

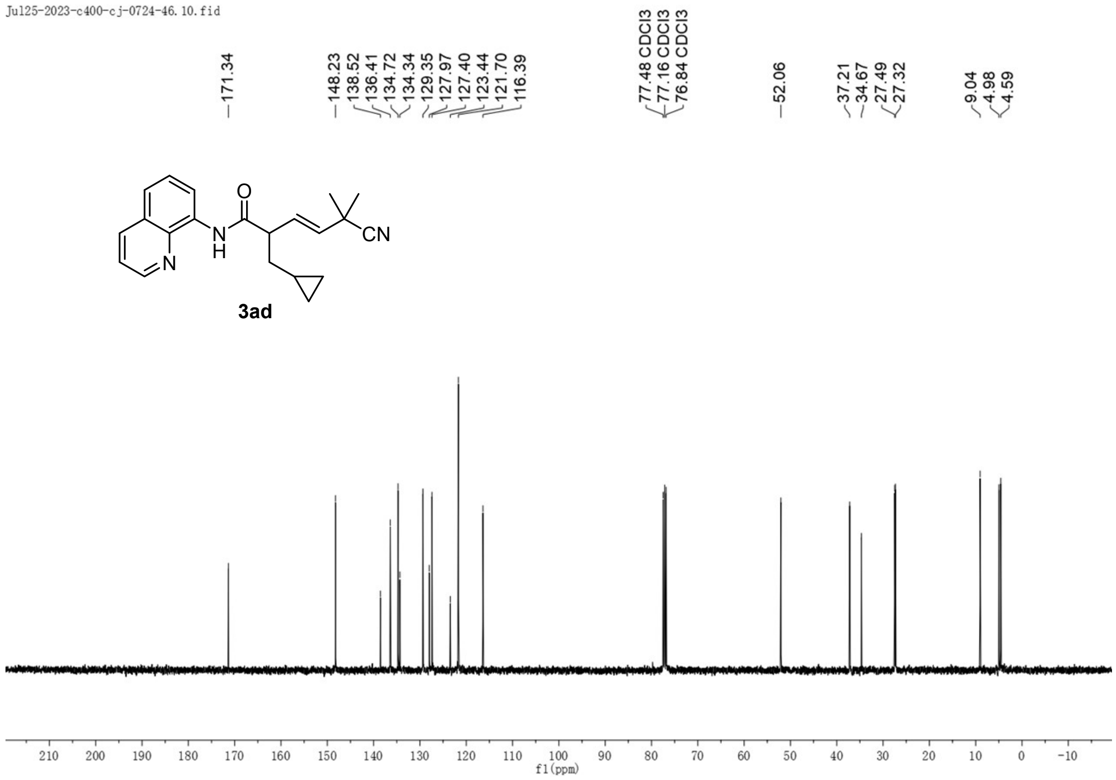

^{1}H NMR of 3ae ($CDCl_3$, 400 MHz)

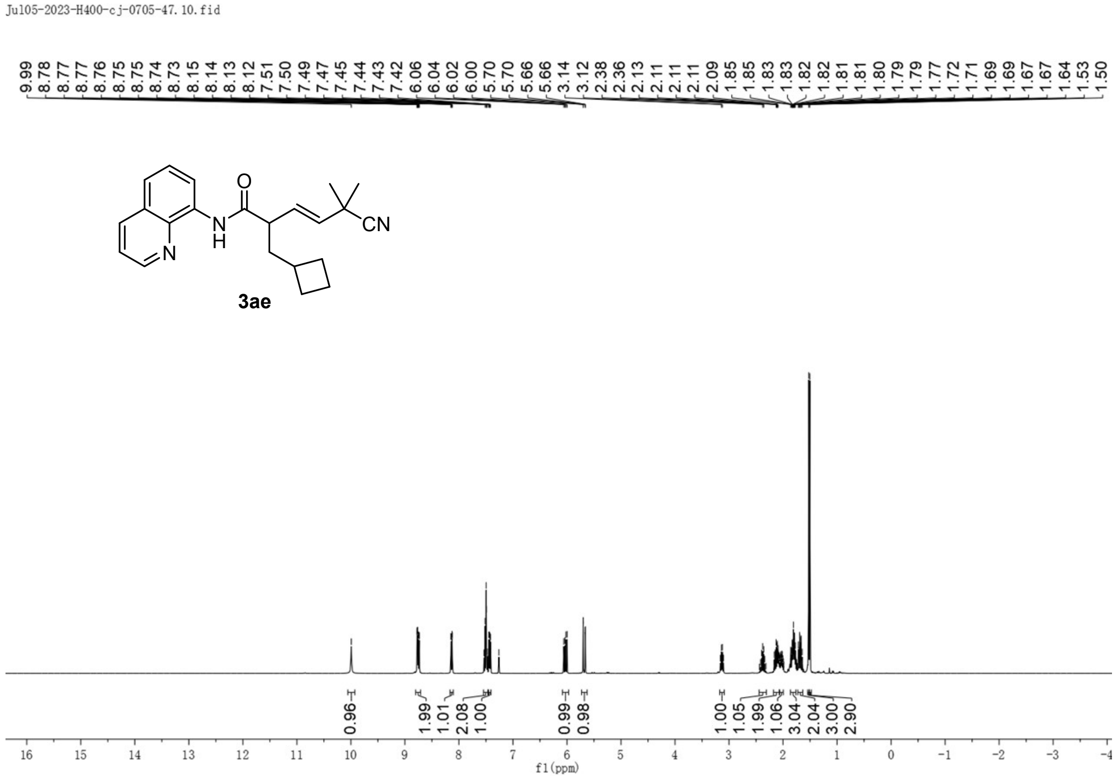

^{13}C NMR of 3ae ($CDCl_3$, 101 MHz)

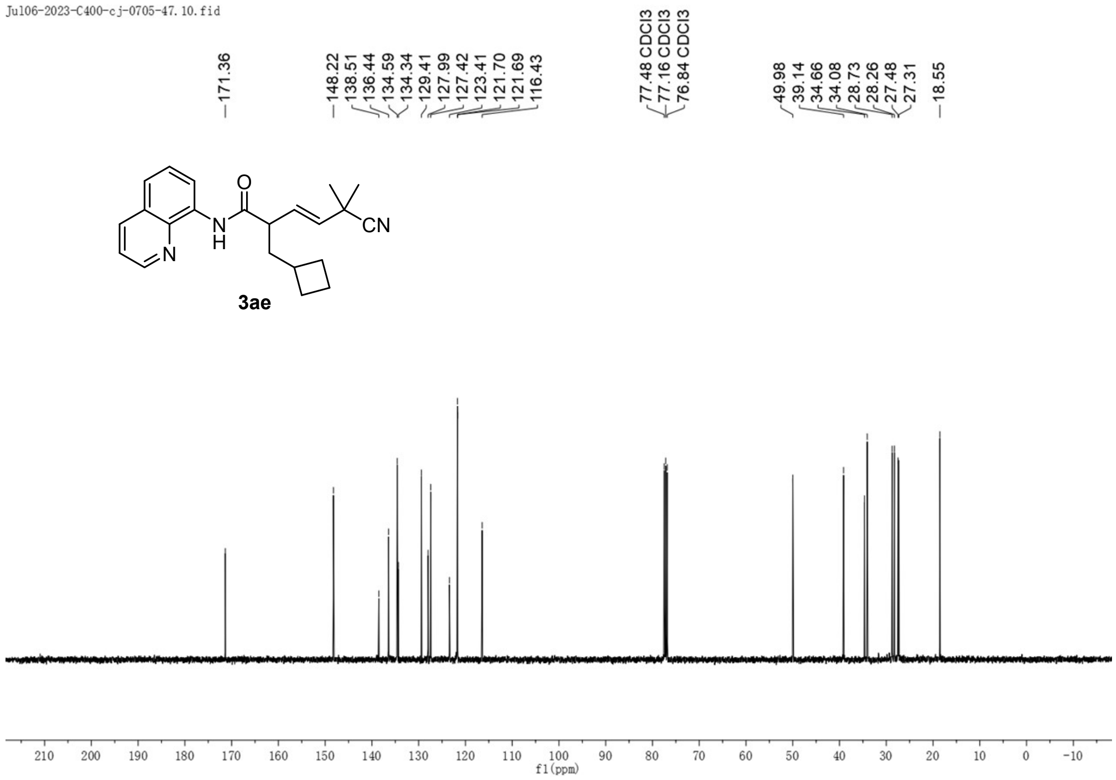

^{1}H NMR of 3af ($CDCl_3$, 400 MHz)

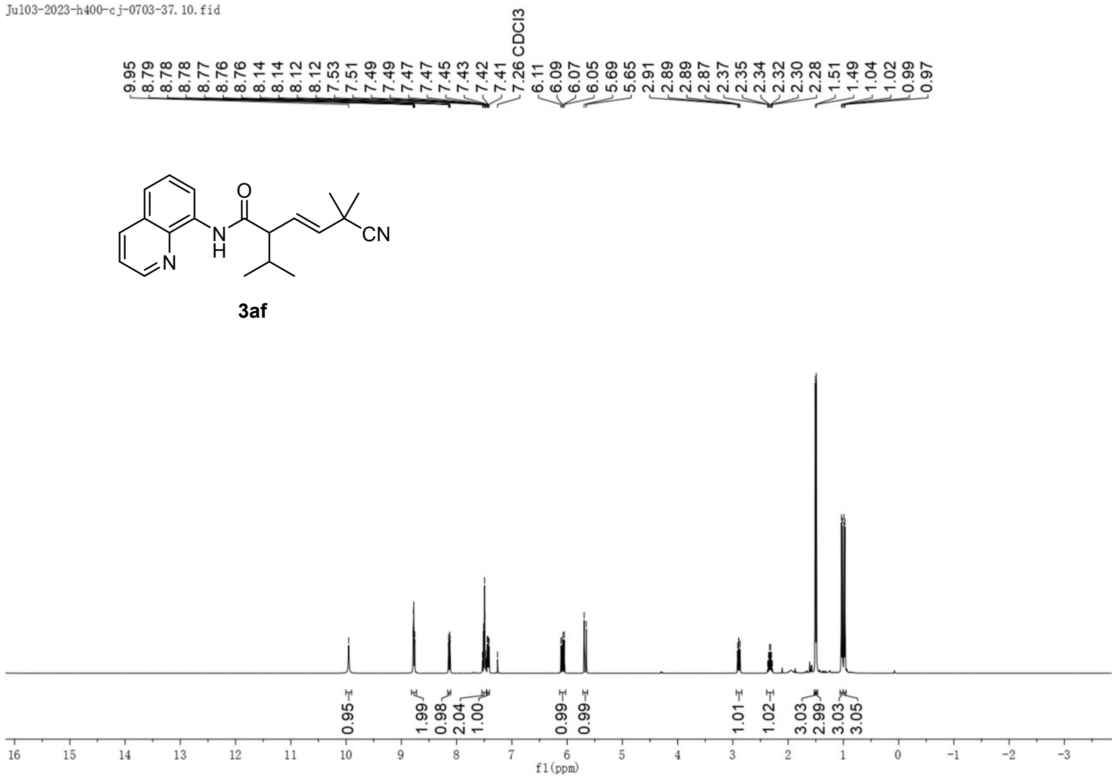

^{13}C NMR of 3af ($CDCl_3$, 101 MHz)

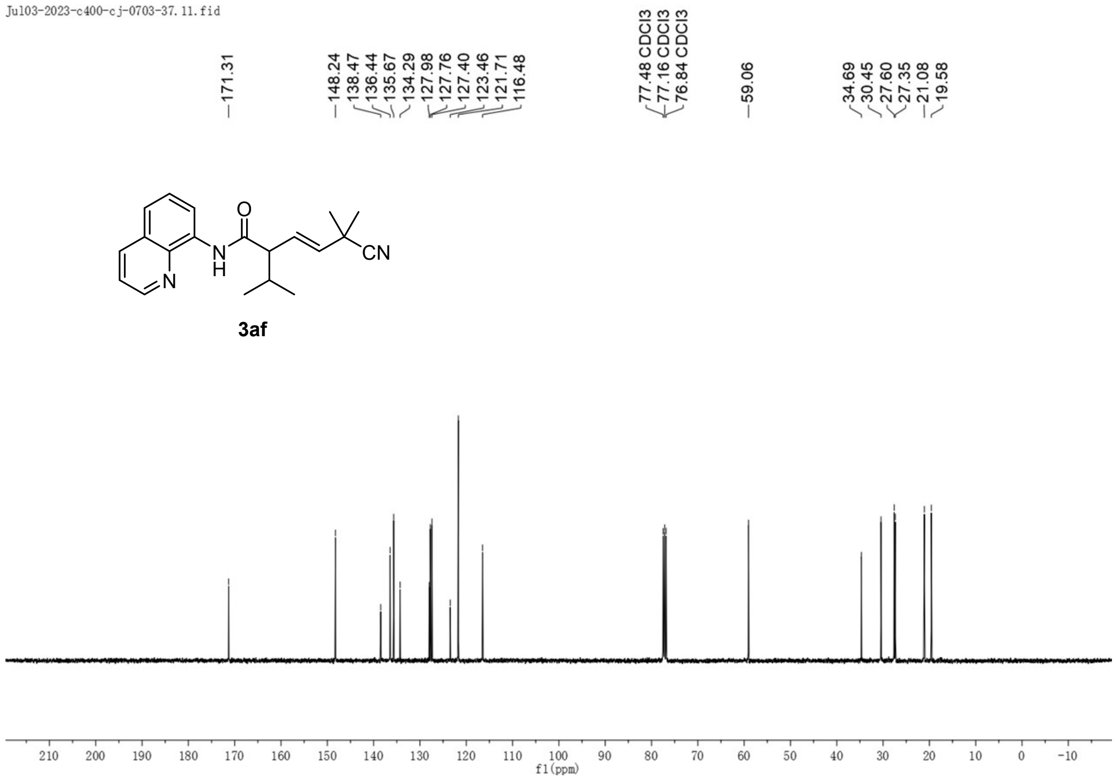

^{1}H NMR of 3ag ($CDCl_3$, 400 MHz)

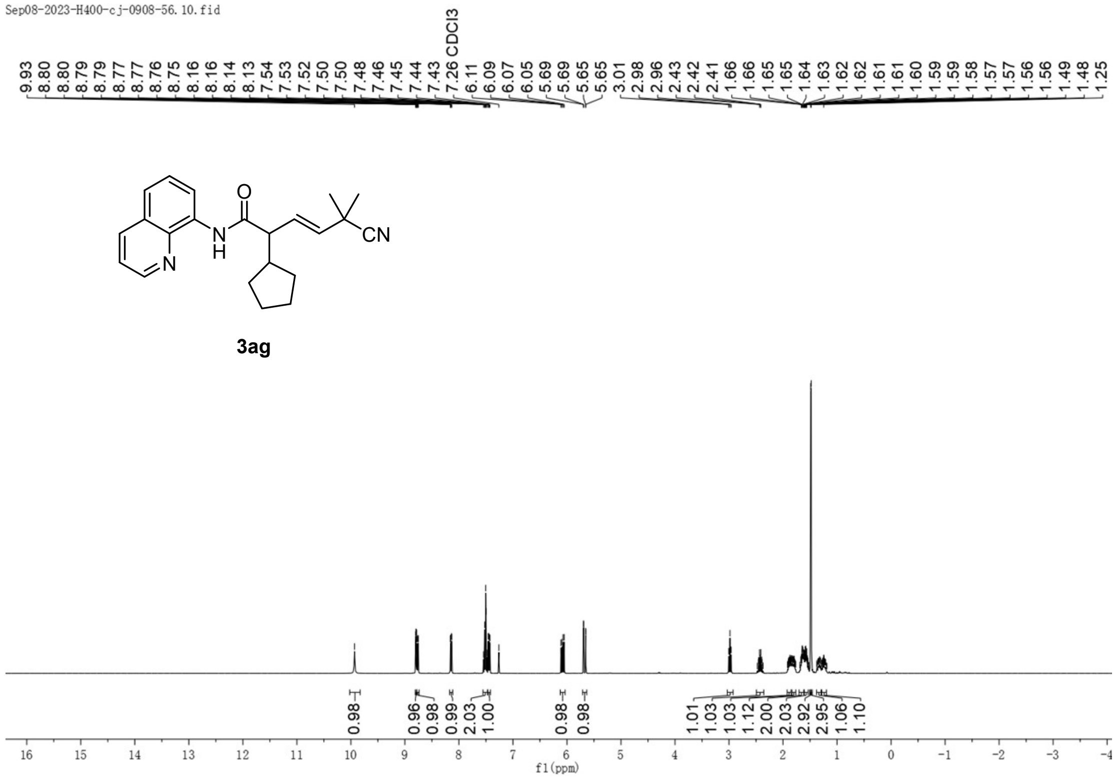

^{13}C NMR of 3ag ($CDCl_3$, 101 MHz)

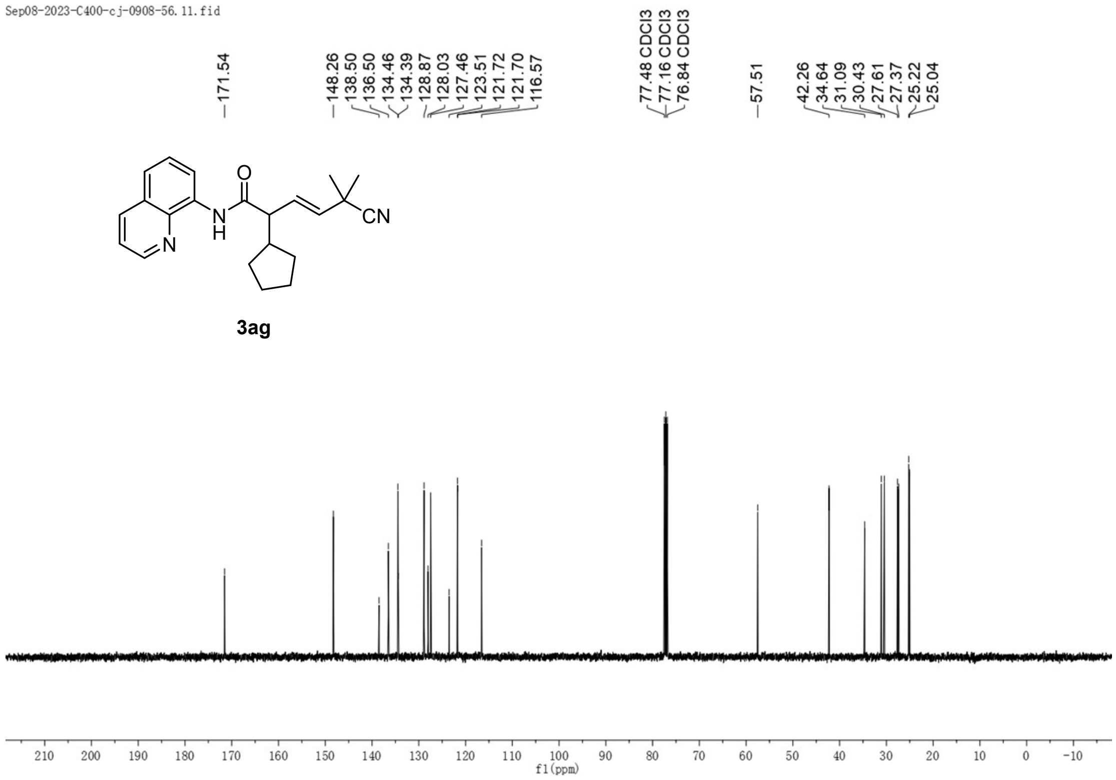

^{1}H NMR of 3ah ($CDCl_3$, 400 MHz)

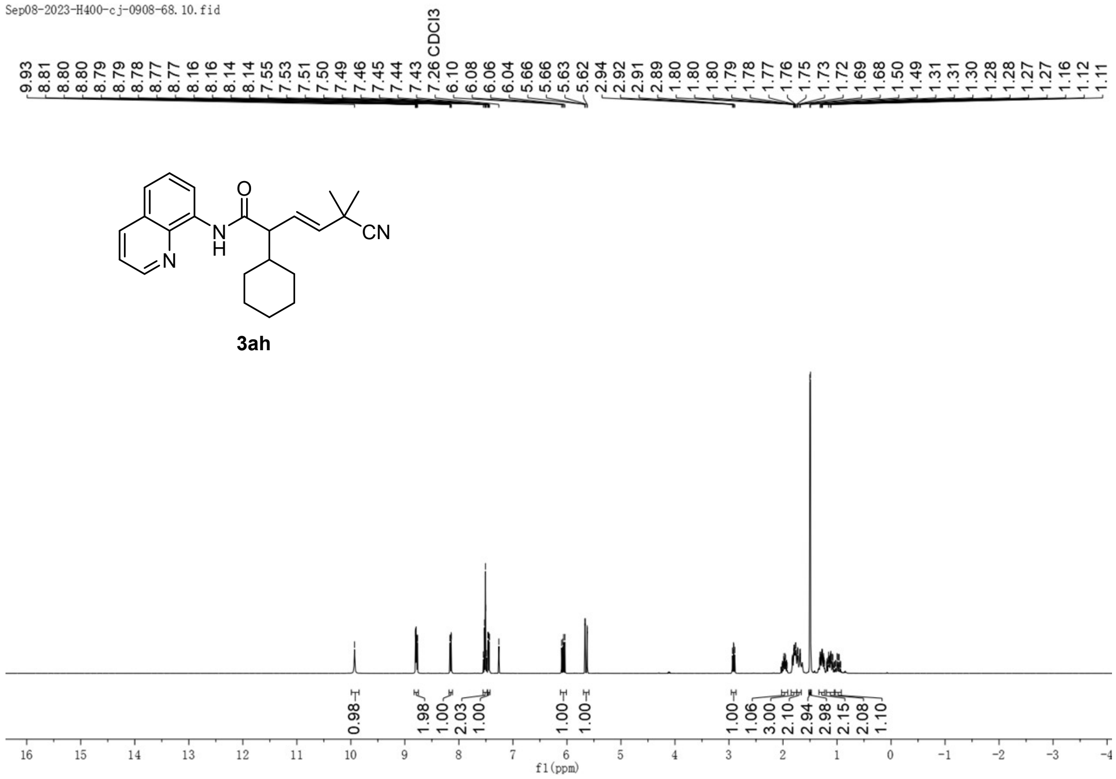

^{13}C NMR of 3ah ($CDCl_3$, 101 MHz)

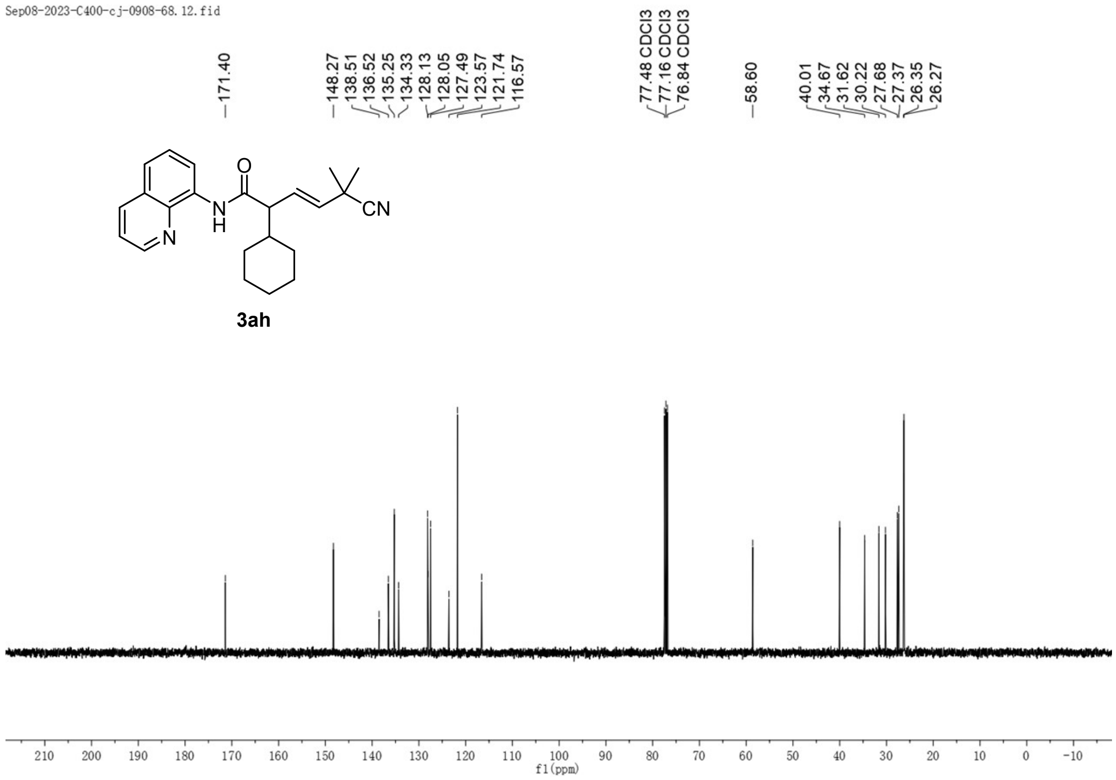

^{1}H NMR of 3ai ($CDCl_3$, 400 MHz)

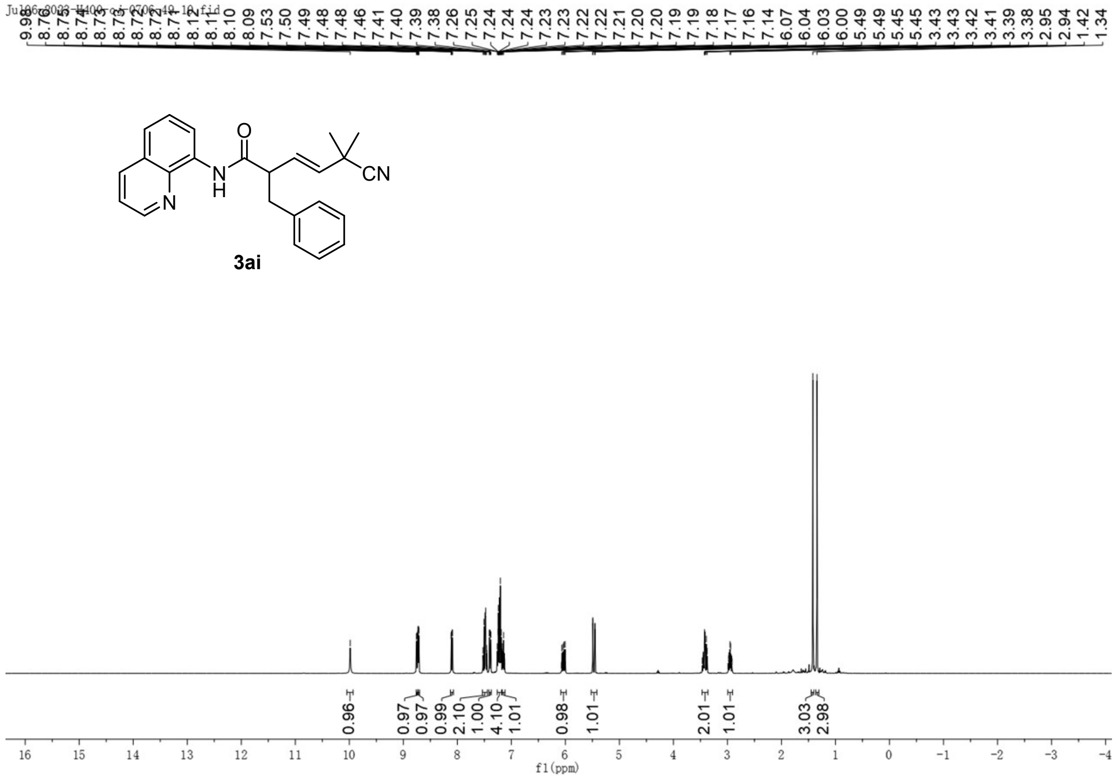

^{13}C NMR of 3ai ($CDCl_3$, 101 MHz)

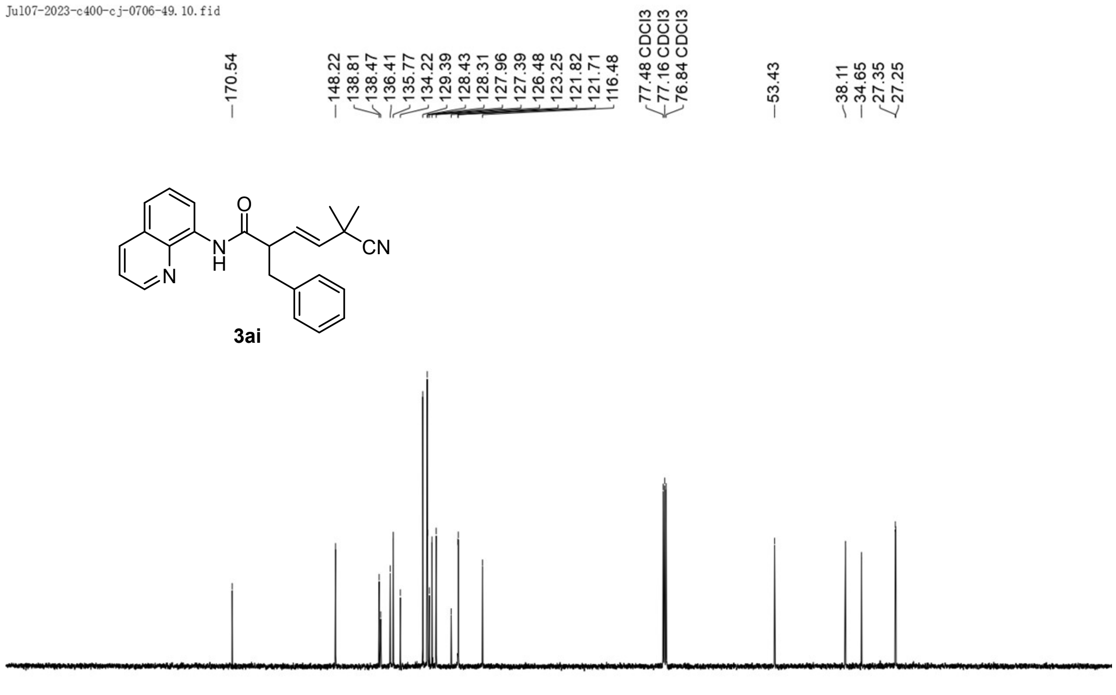

^{1}H NMR of 3aj ($CDCl_3$, 400 MHz)

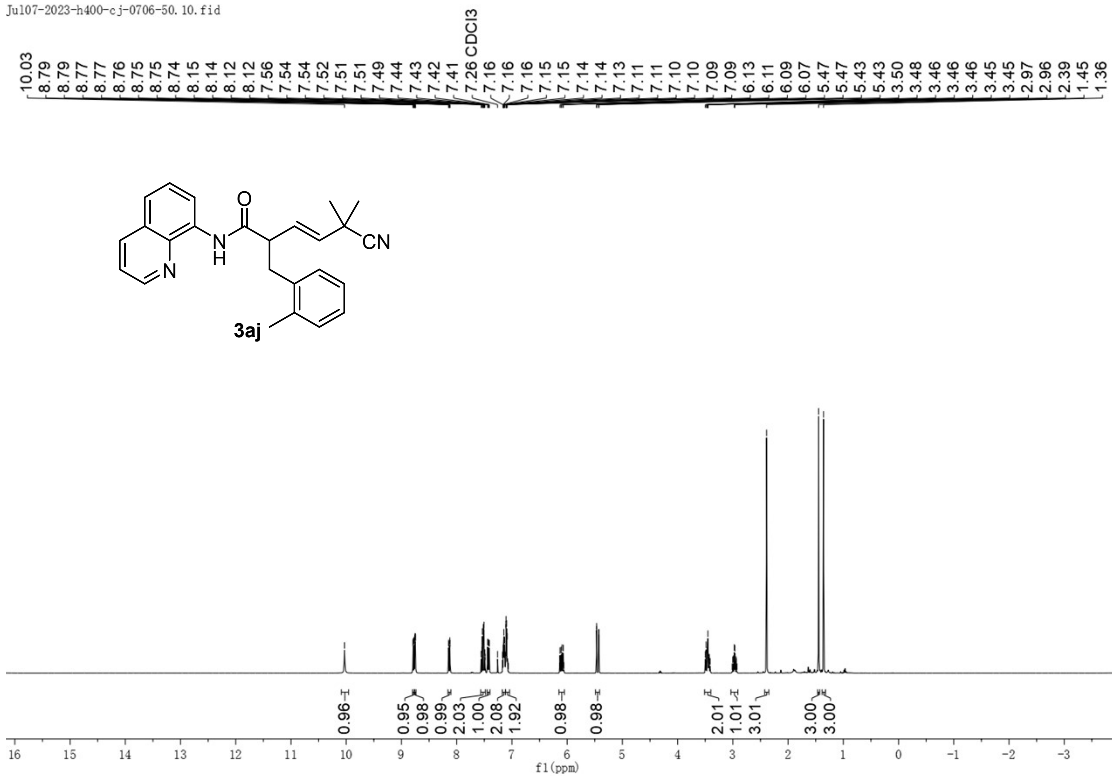

^{13}C NMR of 3aj ($CDCl_3$, 101 MHz)

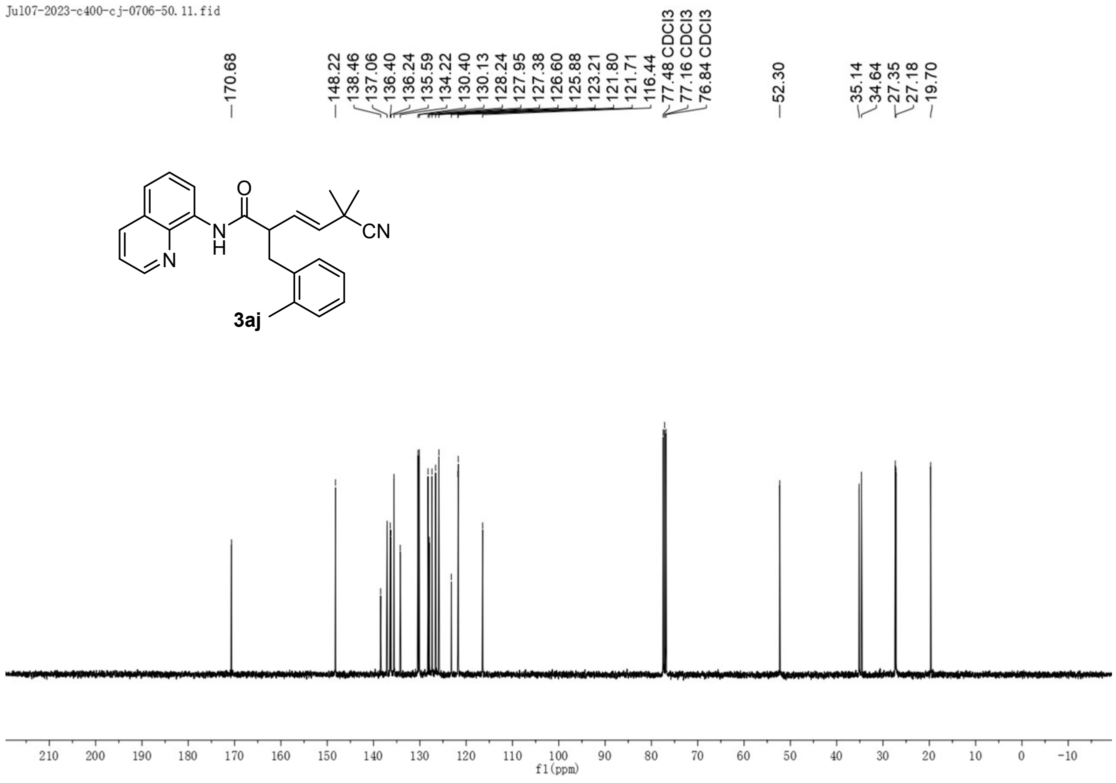

^{1}H NMR of 3ak ($CDCl_3$, 400 MHz)

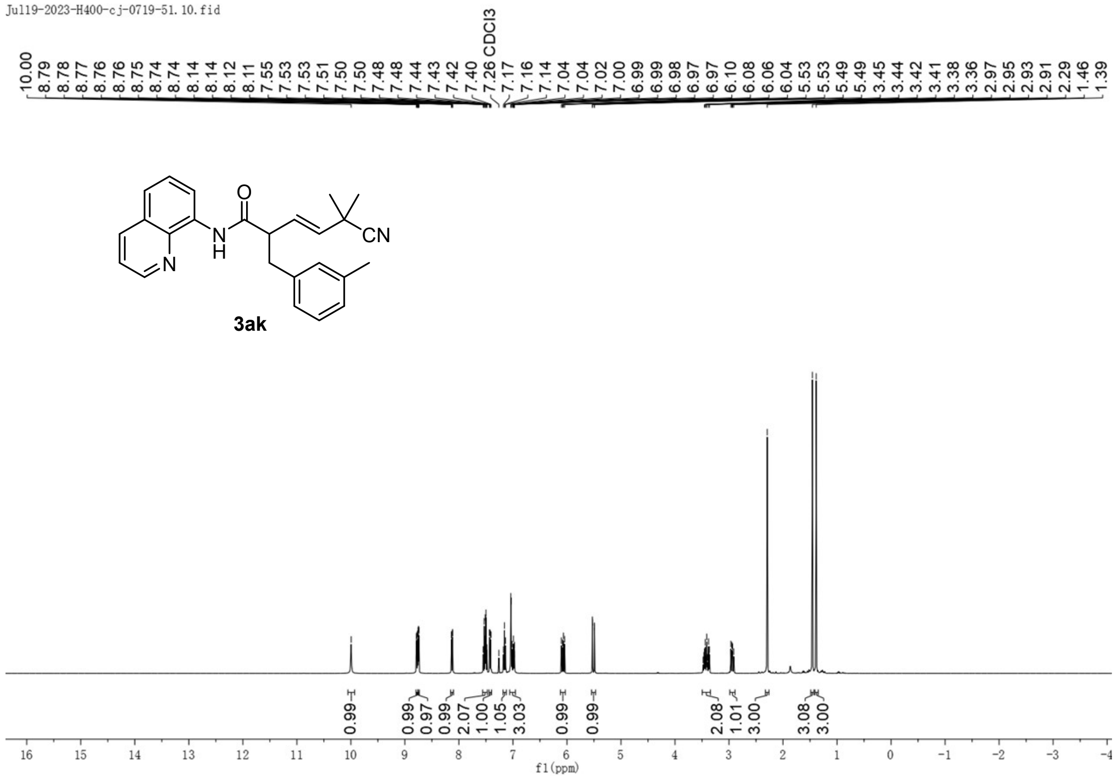

^{13}C NMR of 3ak ($CDCl_3$, 101 MHz)

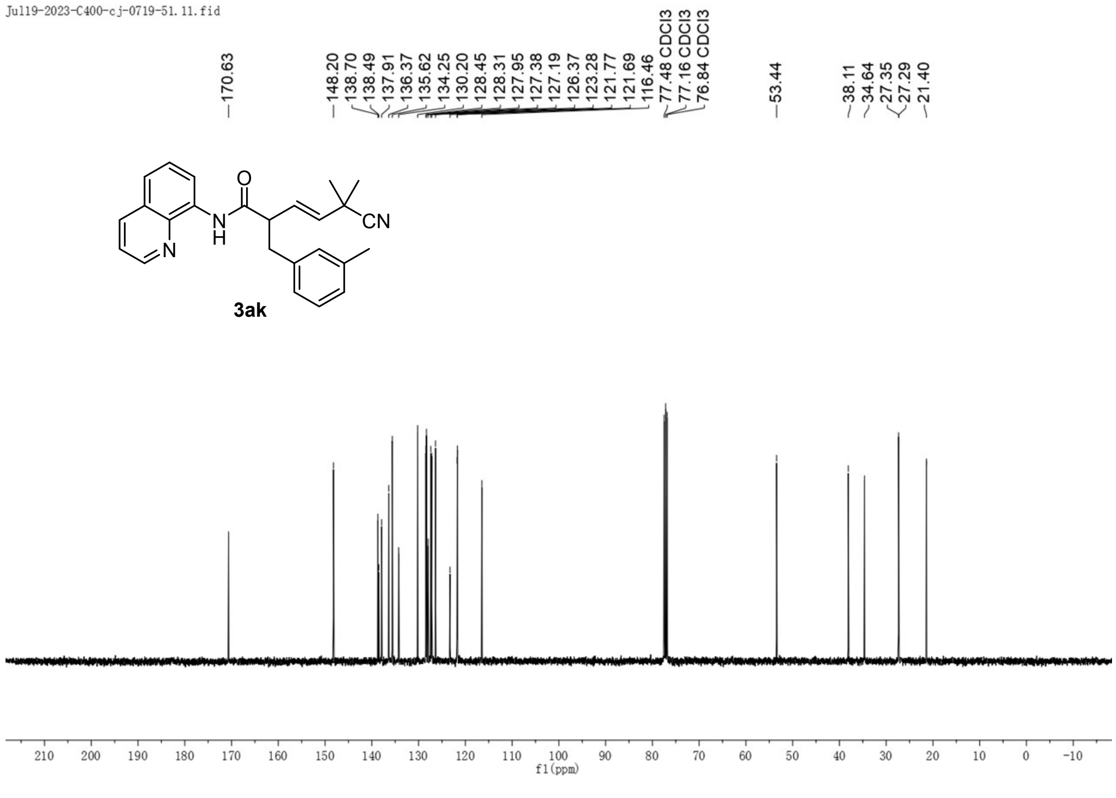

^{1}H NMR of 3al ($CDCl_3$, 400 MHz)

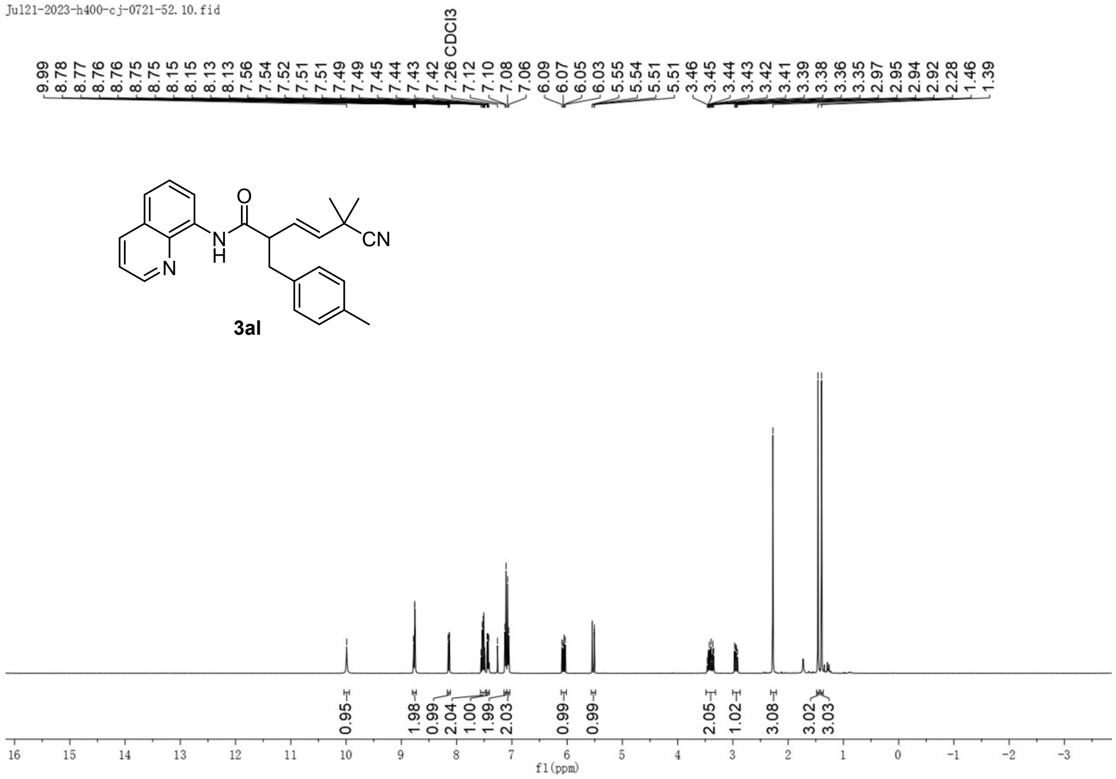

^{13}C NMR of 3al ($CDCl_3$, 101 MHz)

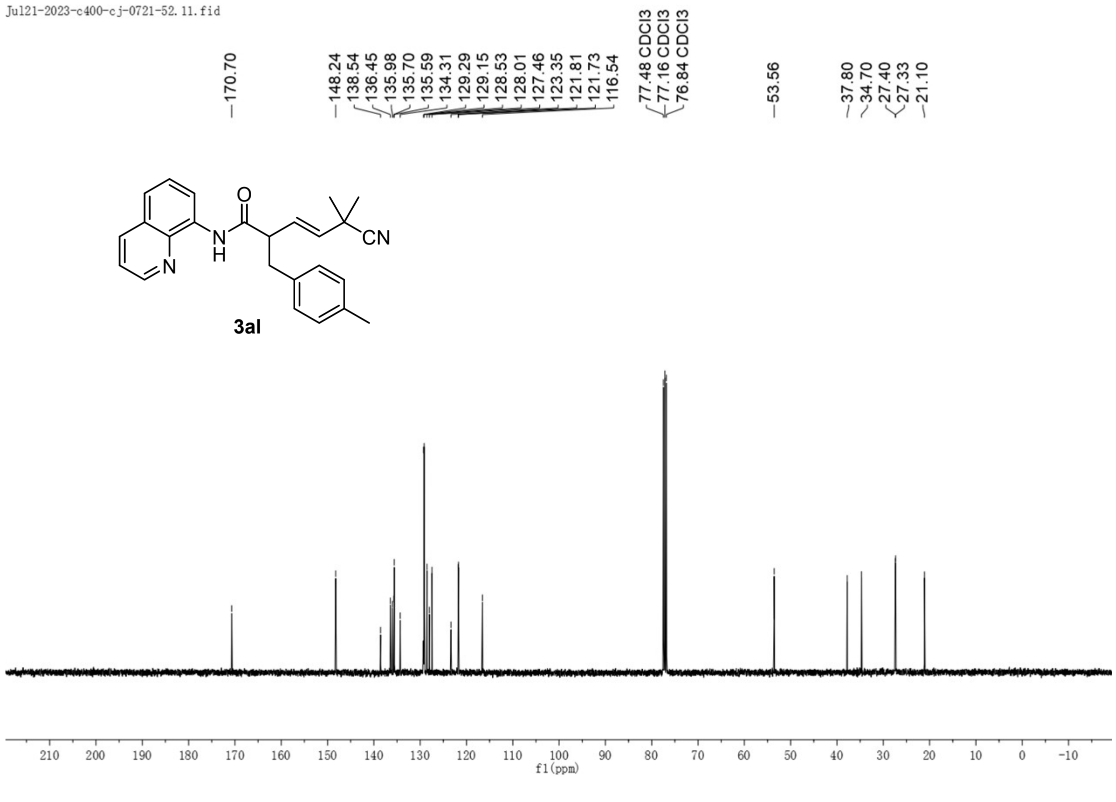

^{1}H NMR of 3am ($CDCl_3$, 400 MHz)

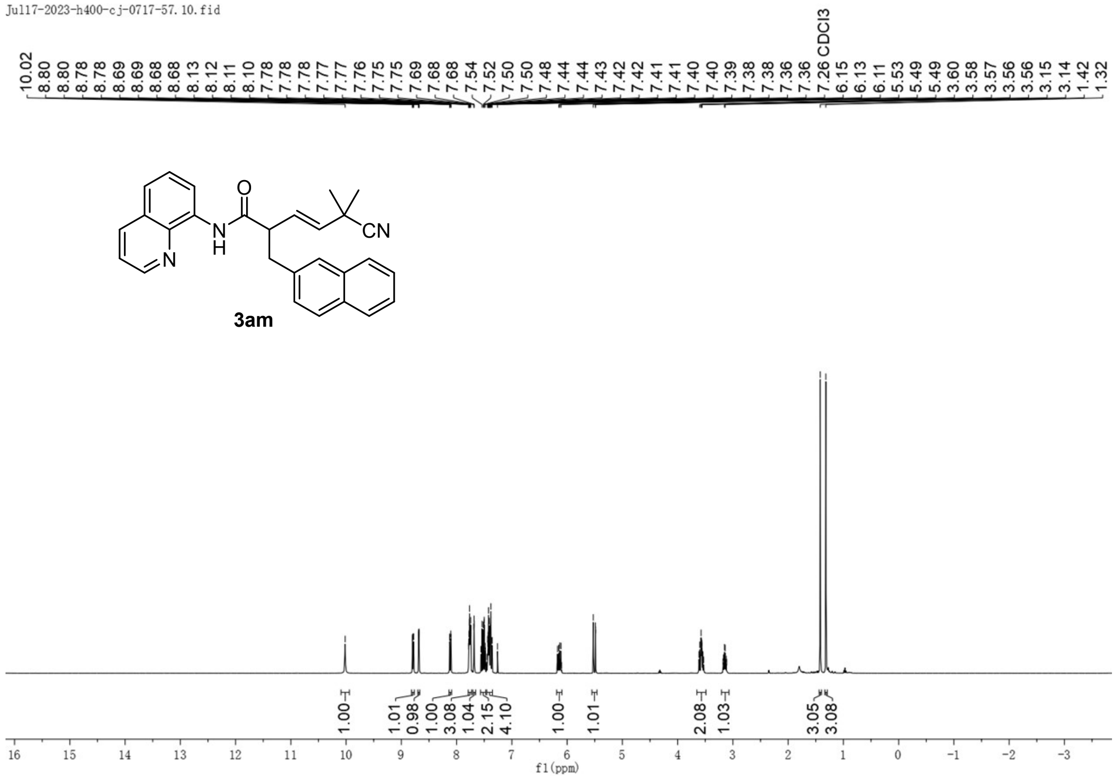

^{13}C NMR of 3am ($CDCl_3$, 101 MHz)

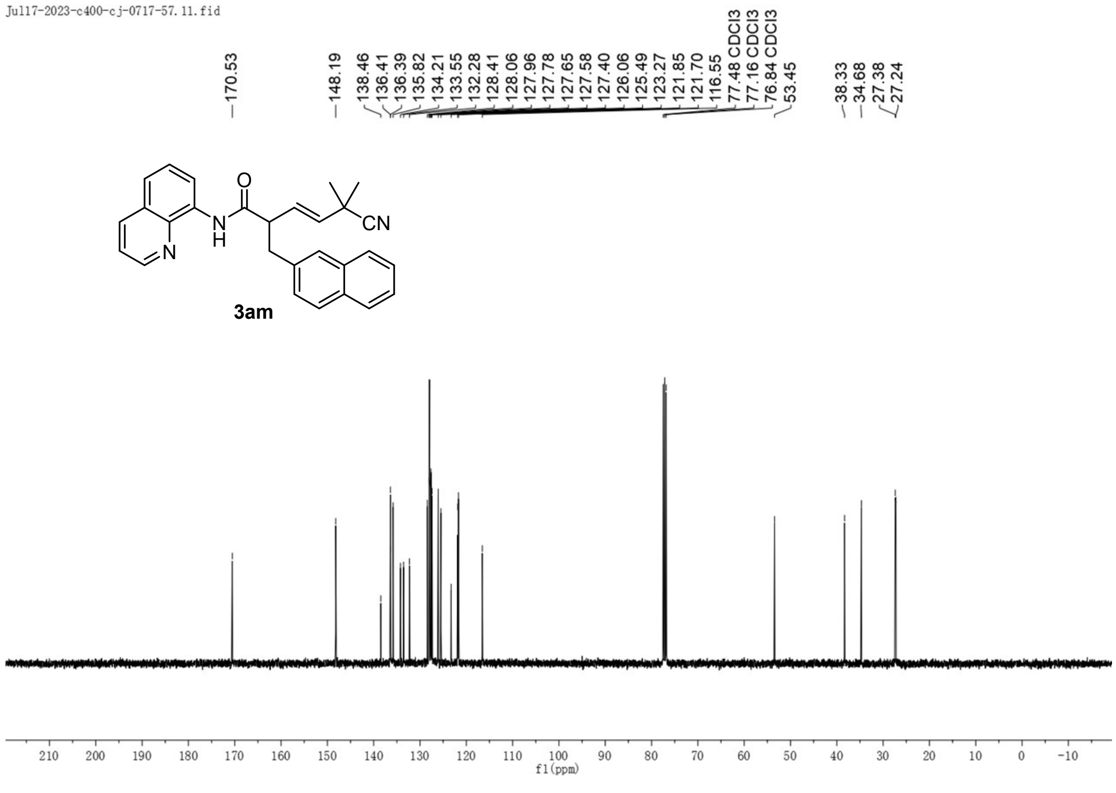

^{1}H NMR of 3an ($CDCl_3$, 400 MHz)

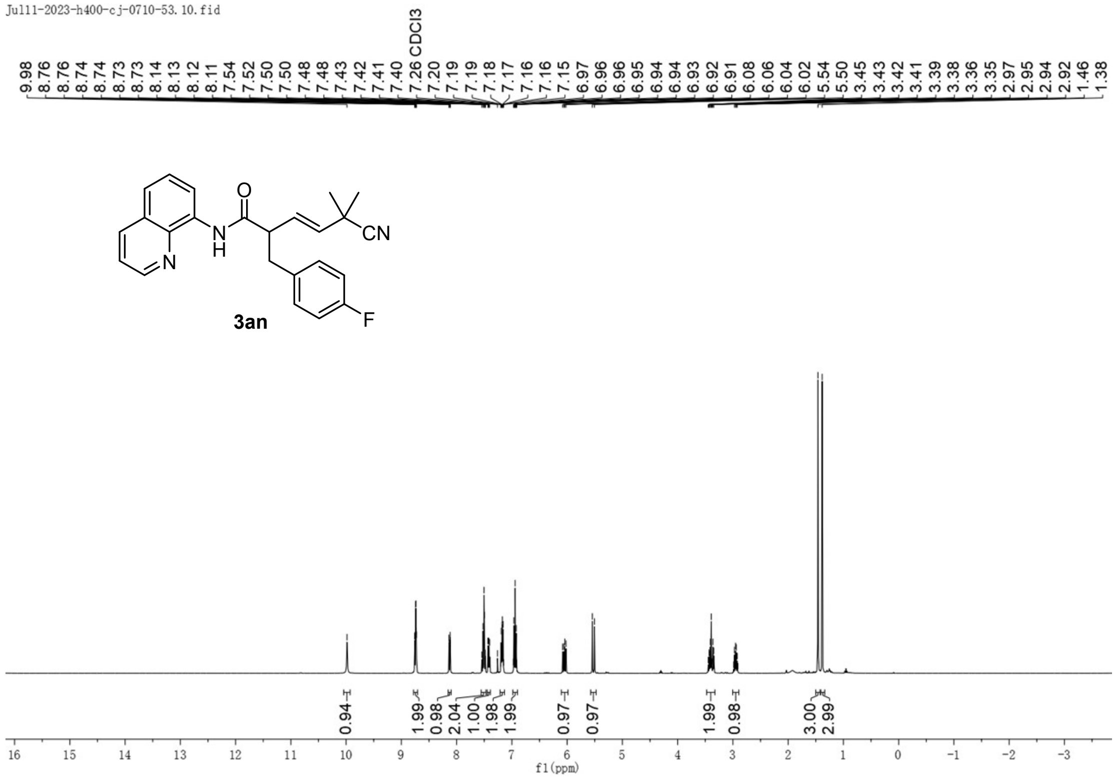

^{13}C NMR of 3an ($CDCl_3$, 101 MHz)

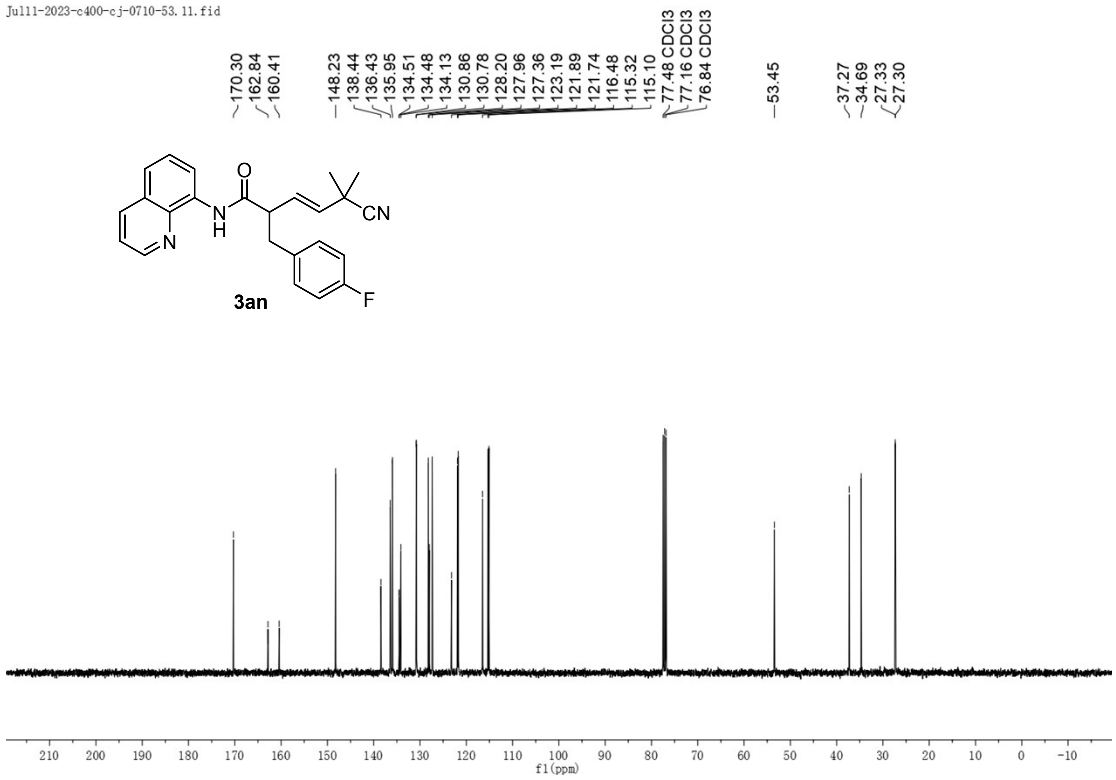

^{19}F NMR of 3an ($CDCl_3$, 376 MHz)

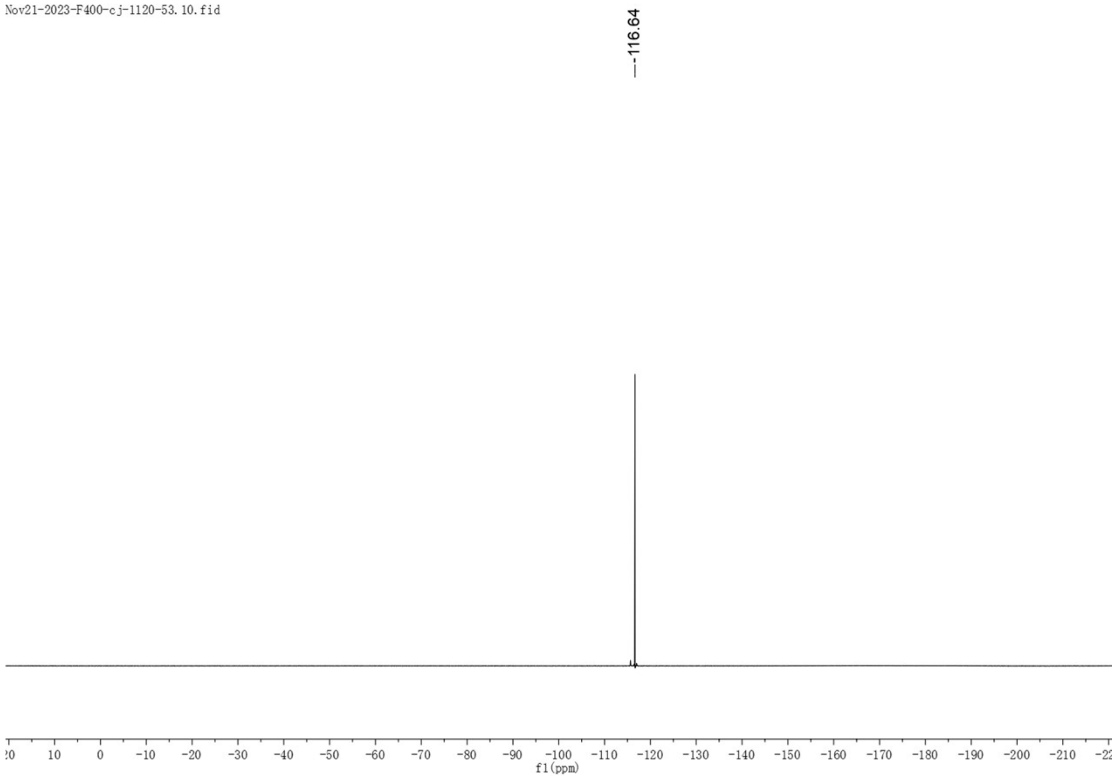

^{1}H NMR of 3ao ($CDCl_3$, 400 MHz)

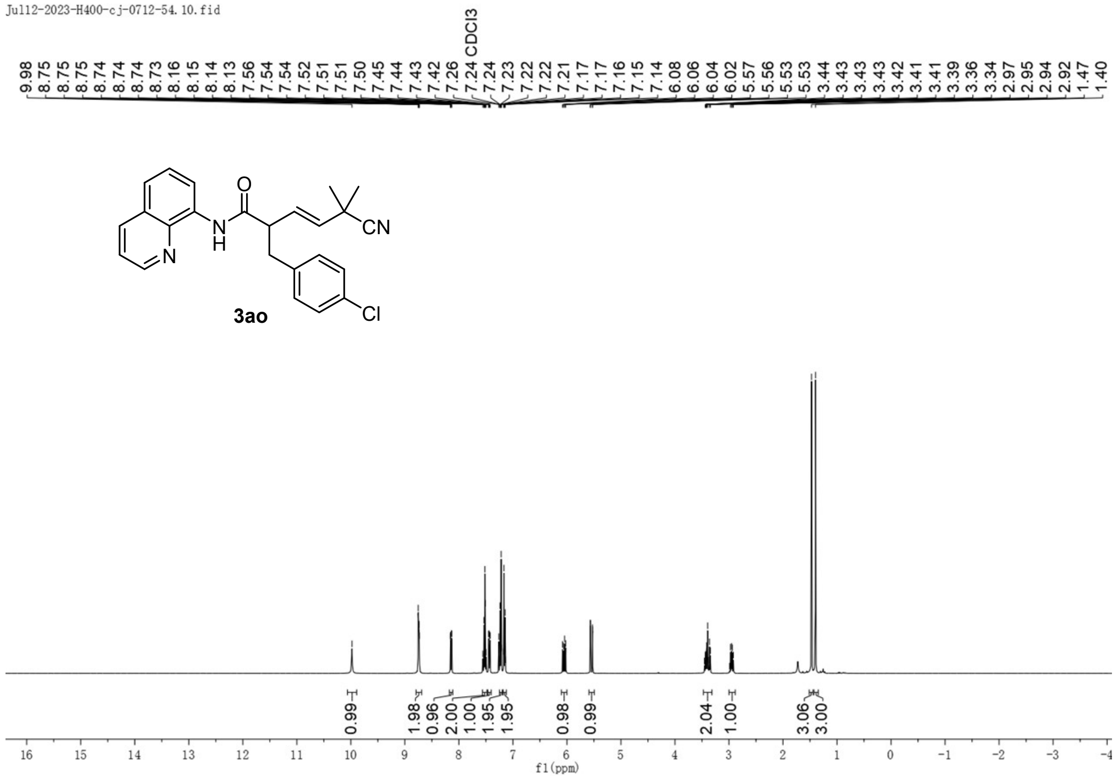

^{13}C NMR of 3ao ($CDCl_3$, 101 MHz)

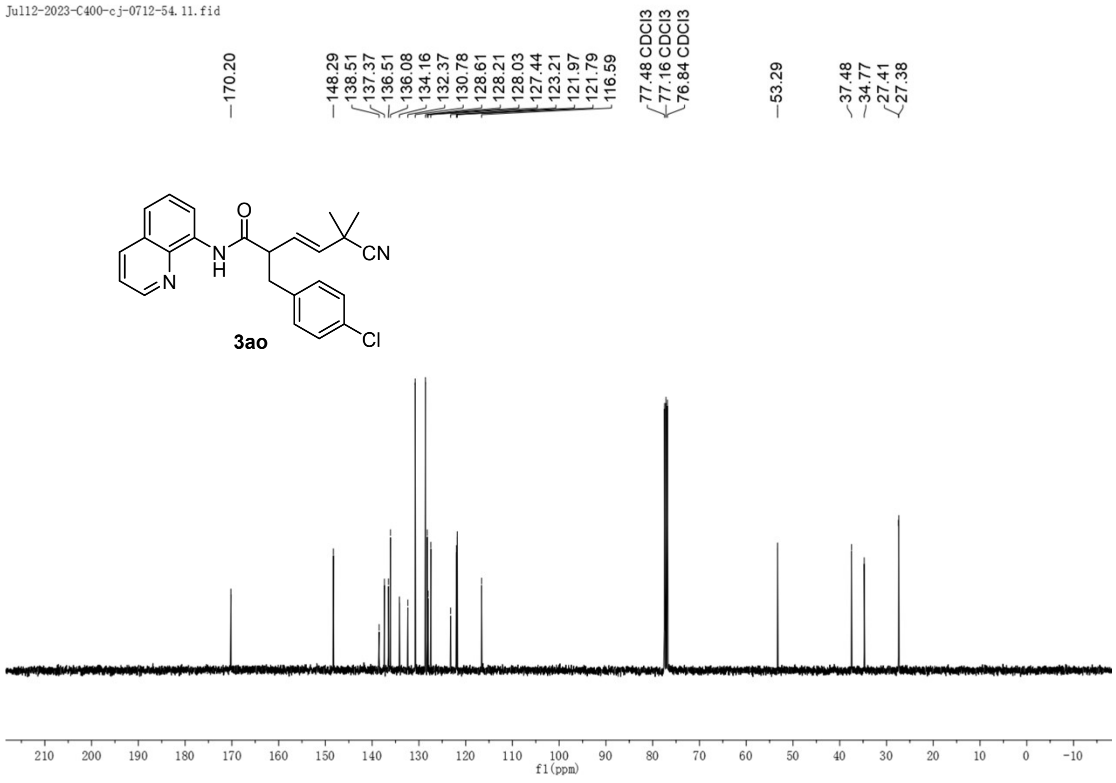

^{1}H NMR of 3ap ($CDCl_3$, 400 MHz)

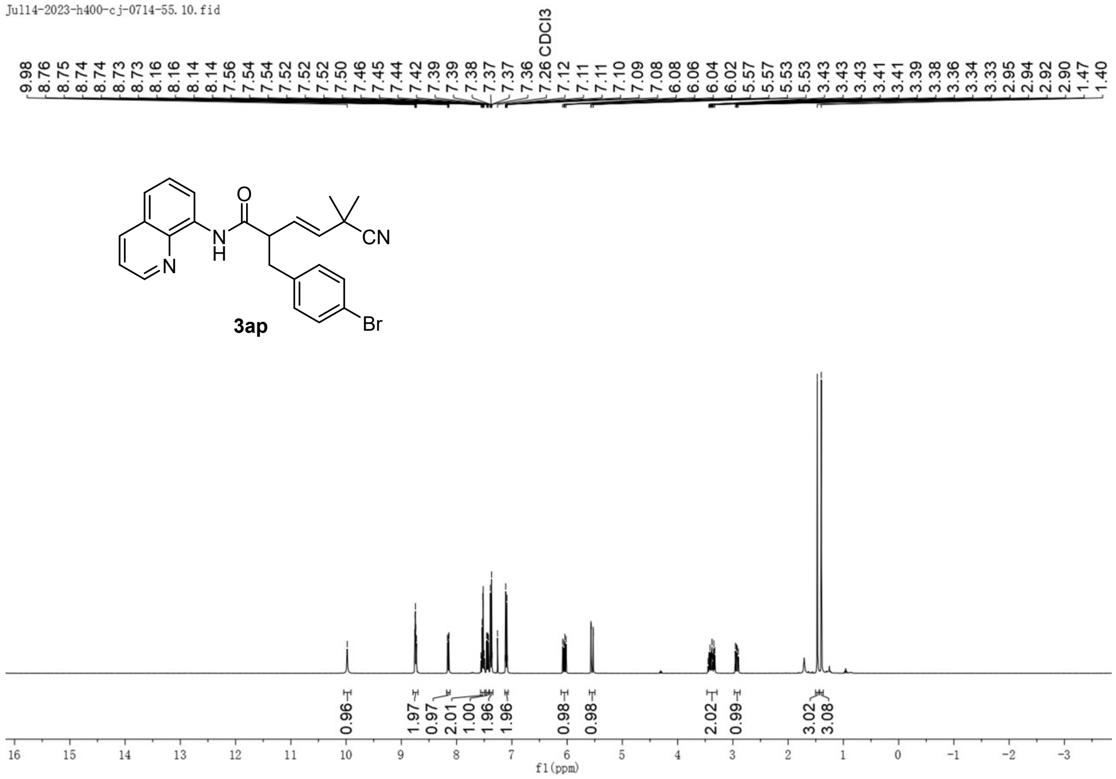

^{13}C NMR of 3ap ($CDCl_3$, 101 MHz)

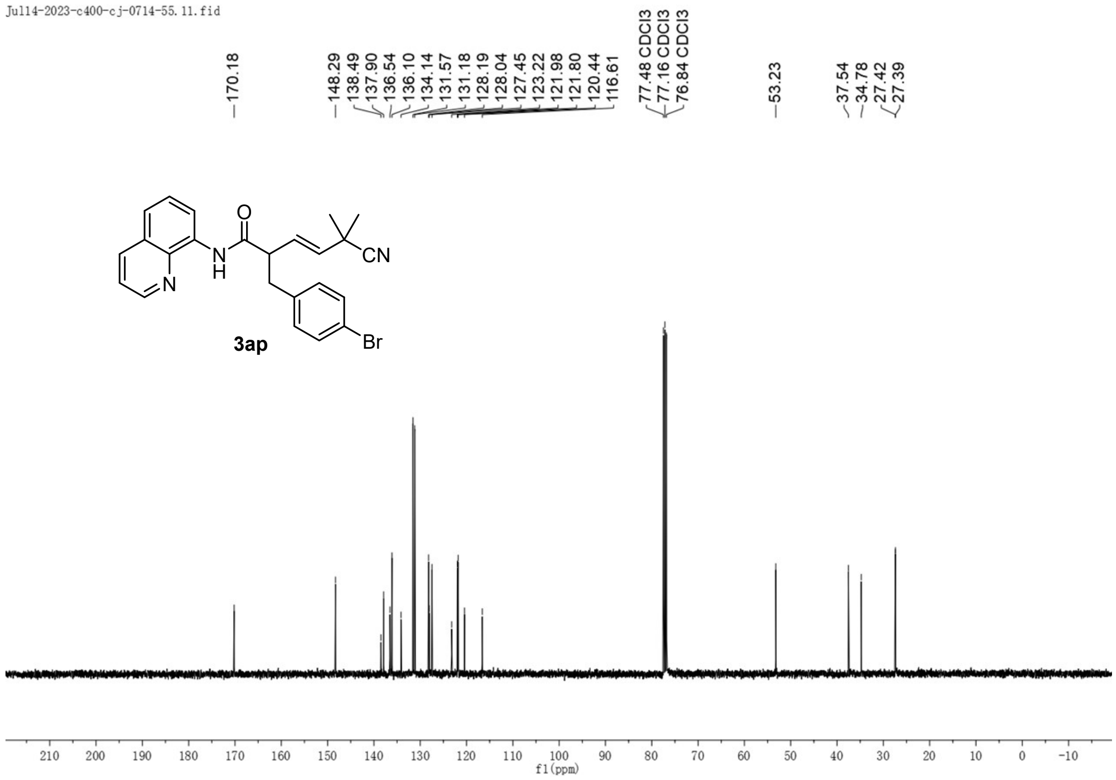

^{1}H NMR of 3aq ($CDCl_3$, 400 MHz)

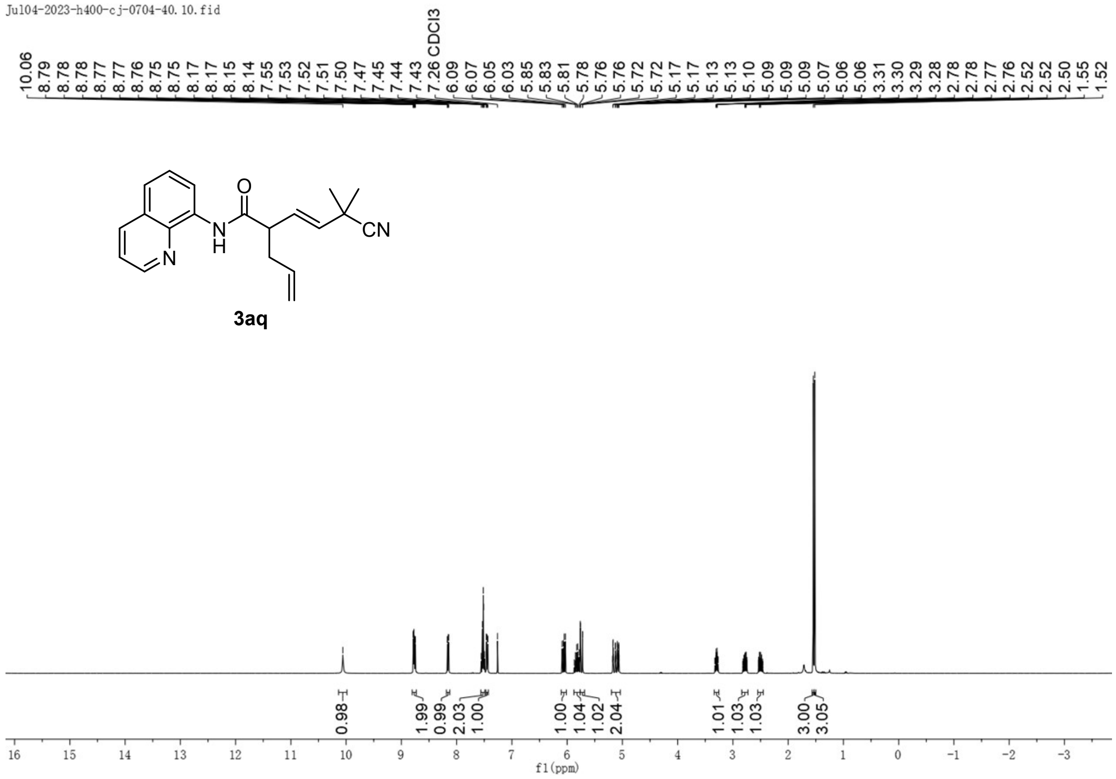

^{13}C NMR of 3aq ($CDCl_3$, 101 MHz)

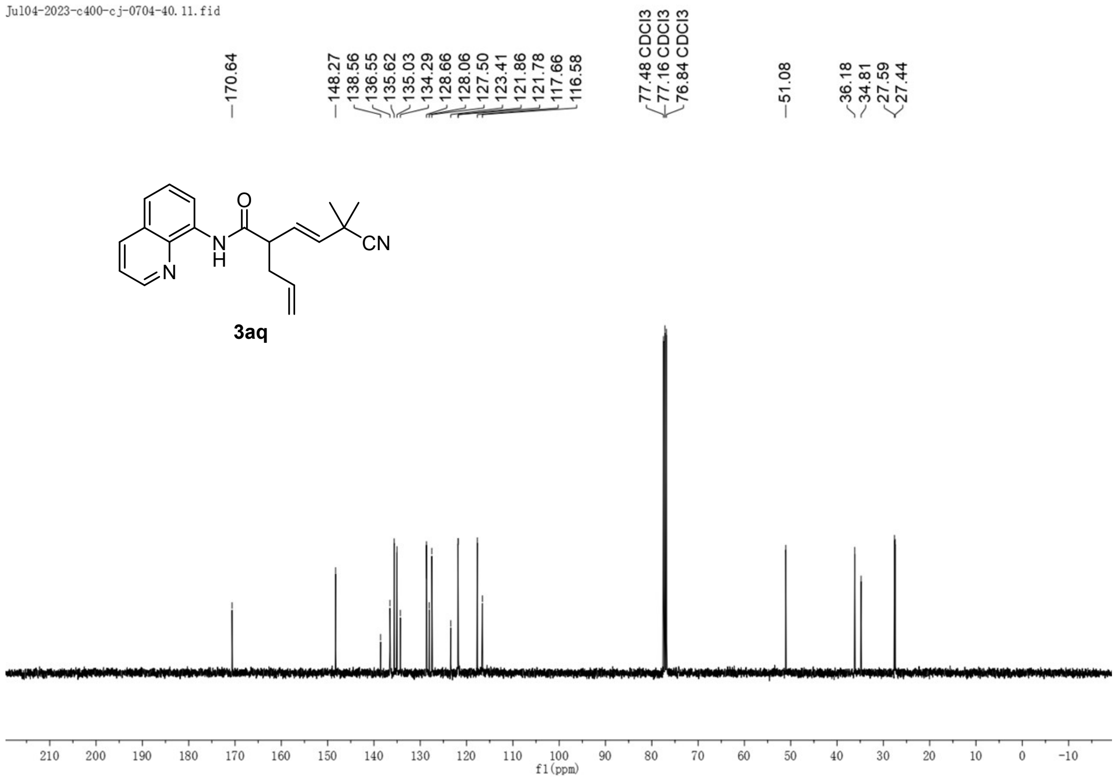

^{1}H NMR of 3ar ($CDCl_3$, 400 MHz)

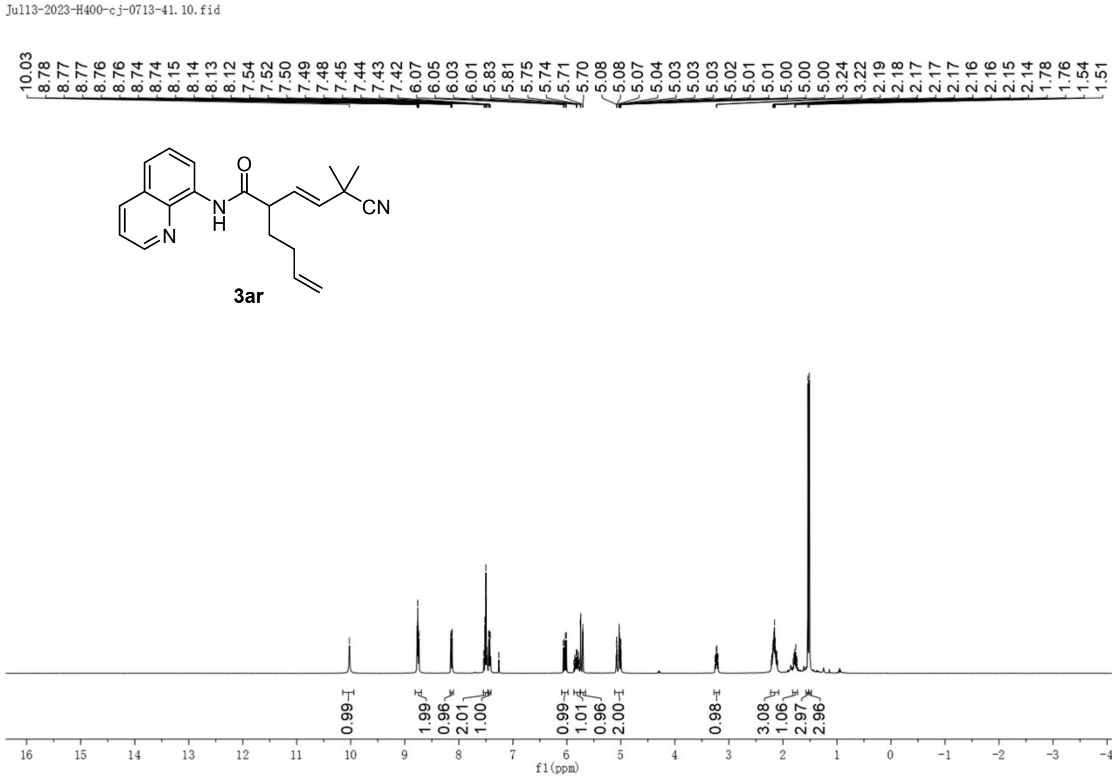

^{13}C NMR of 3ar ($CDCl_3$, 101 MHz)

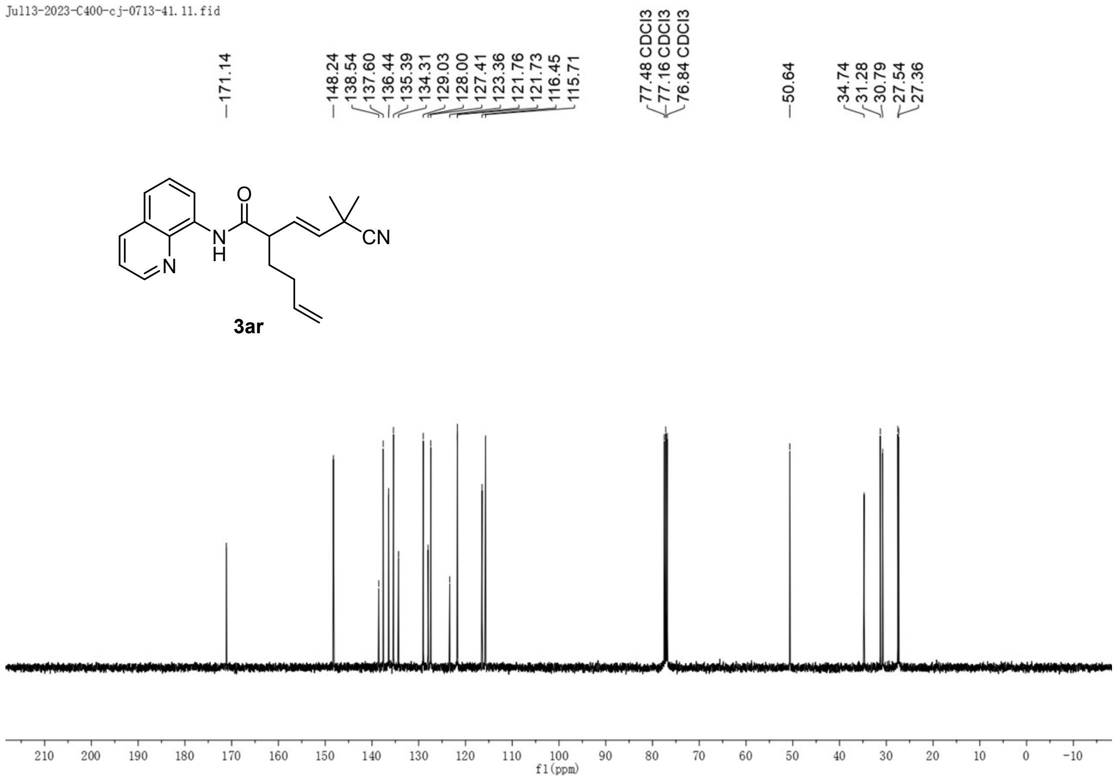

^{1}H NMR of 3as ($CDCl_3$, 400 MHz)

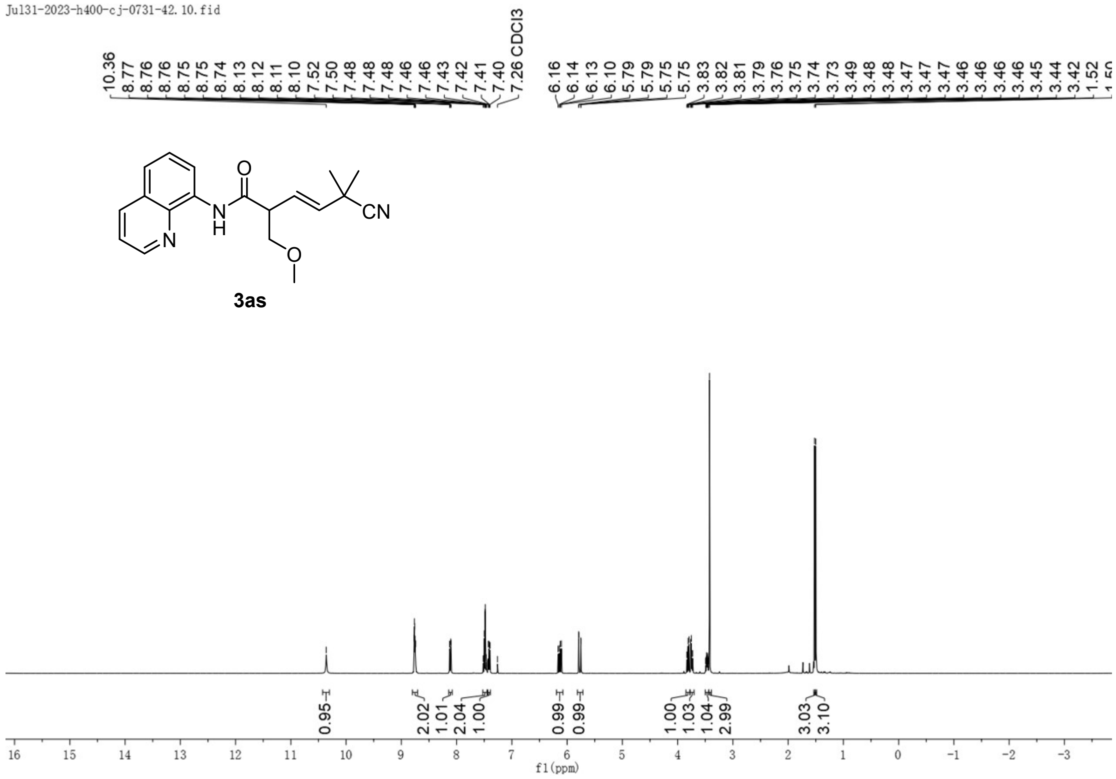

^{13}C NMR of 3as ($CDCl_3$, 101 MHz)

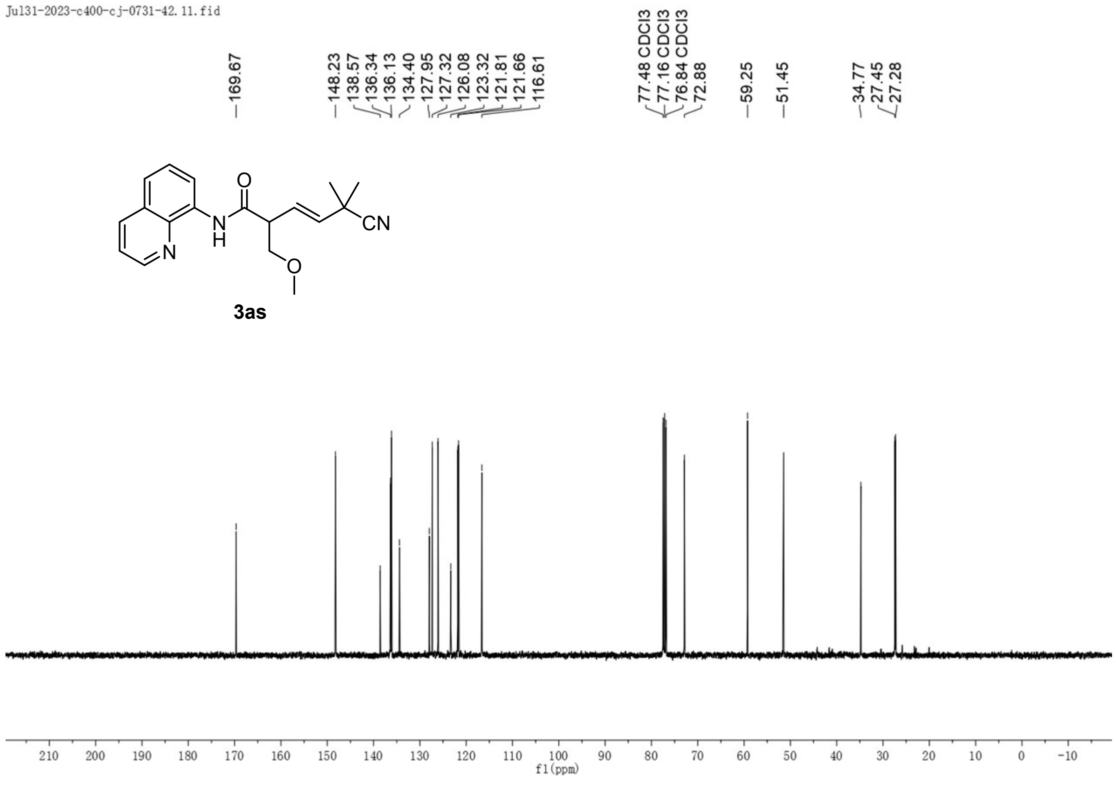

^{1}H NMR of 3at ($CDCl_3$, 400 MHz)

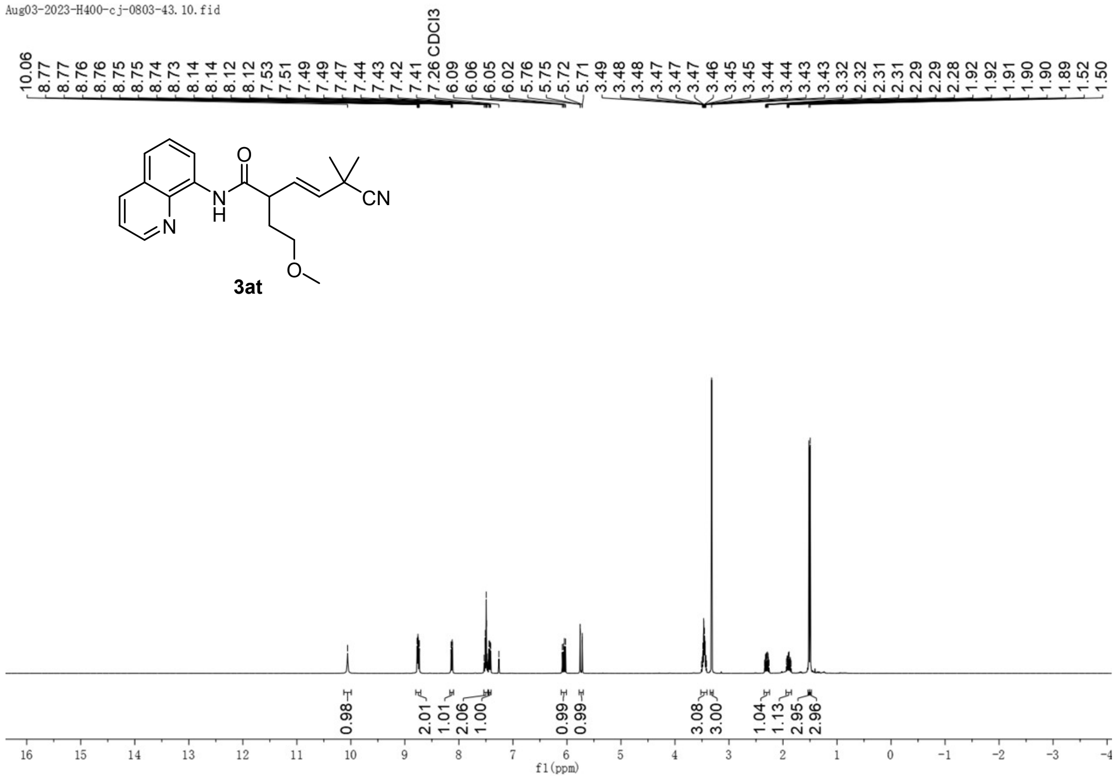

^{13}C NMR of 3at ($CDCl_3$, 101 MHz)

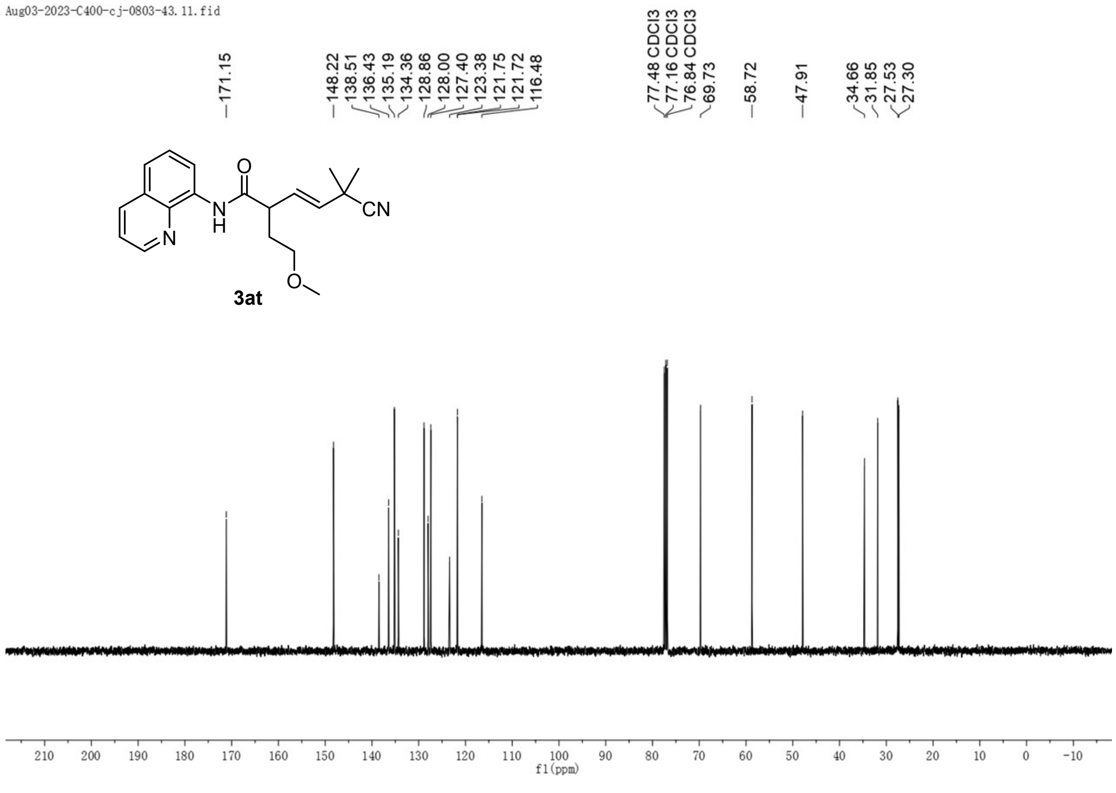

^{1}H NMR of 3au ($CDCl_3$, 400 MHz)

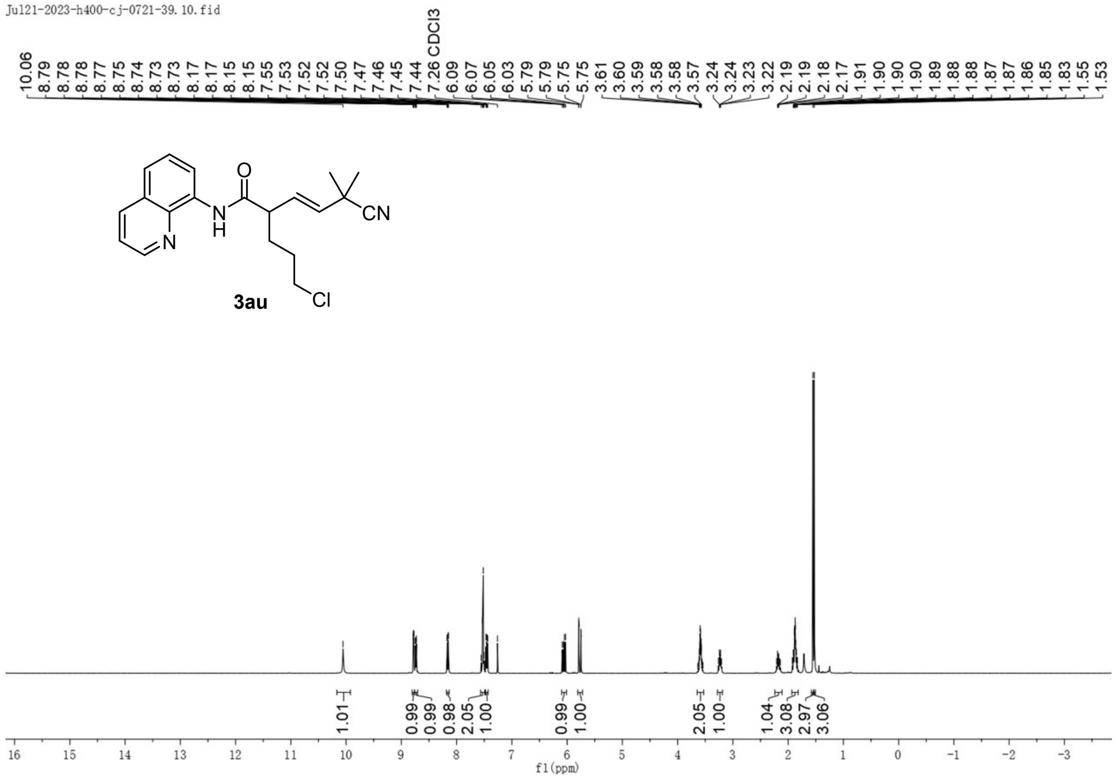

^{13}C NMR of 3au ($CDCl_3$, 101 MHz)

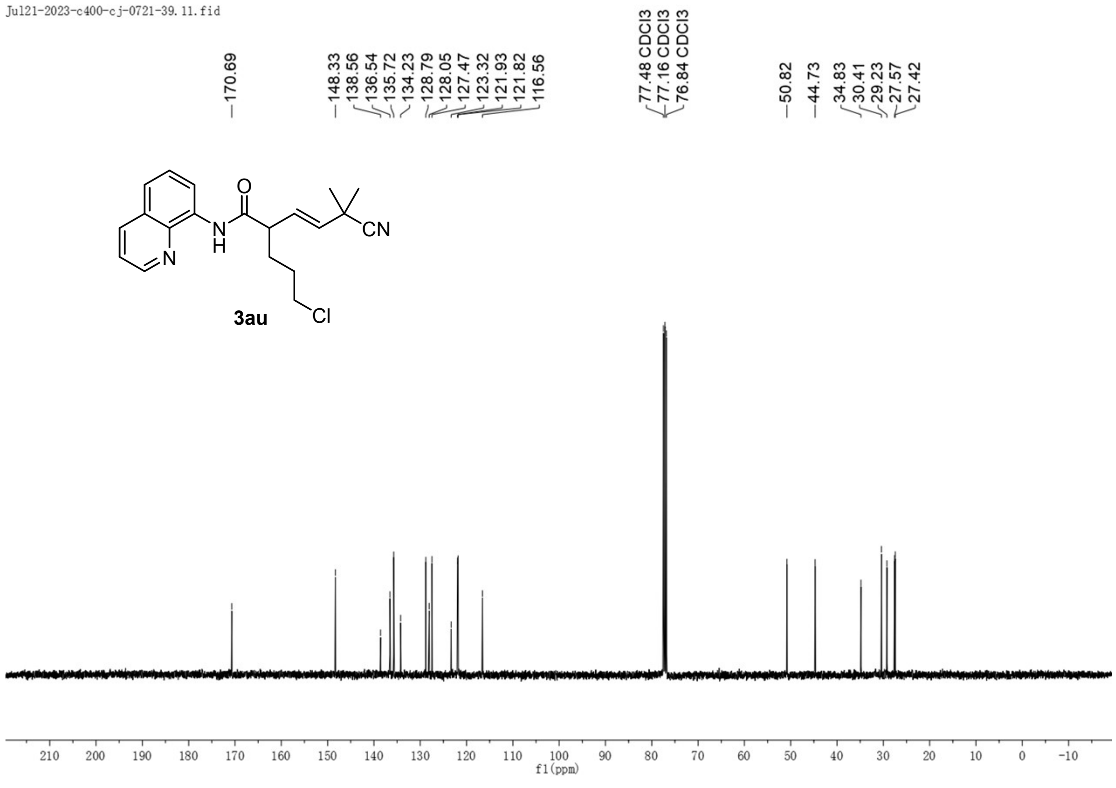

^{1}H NMR of 3av ($CDCl_3$, 400 MHz)

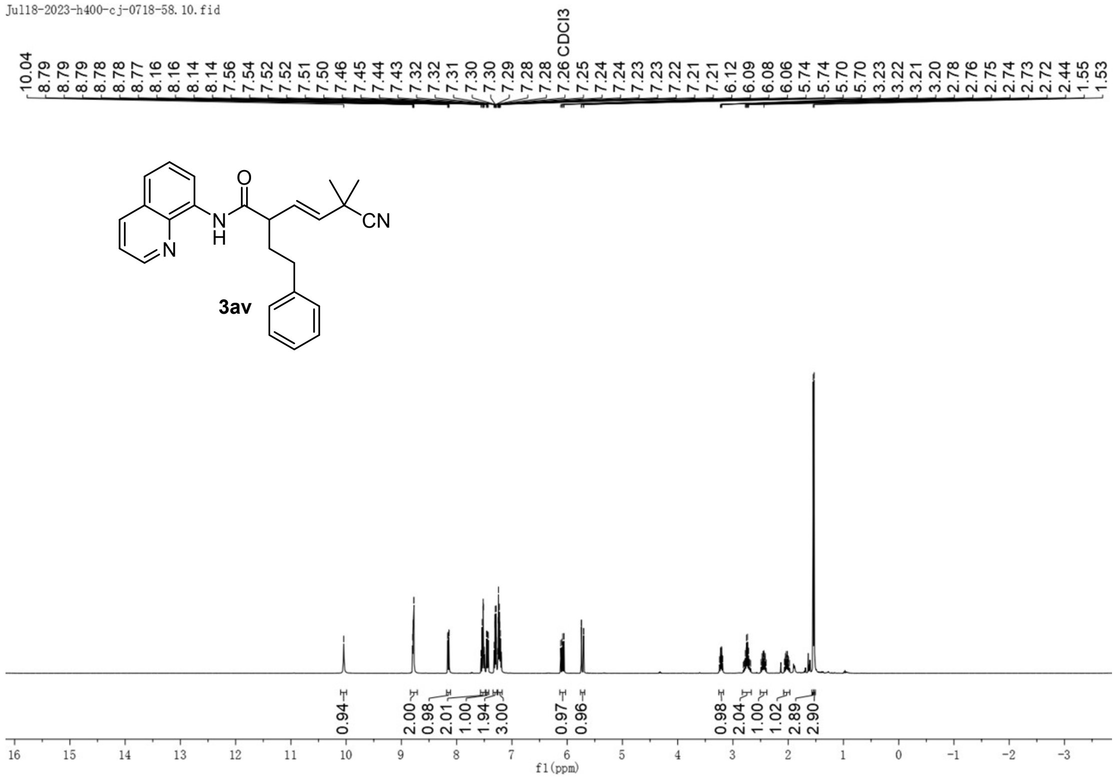

^{13}C NMR of 3av ($CDCl_3$, 101 MHz)

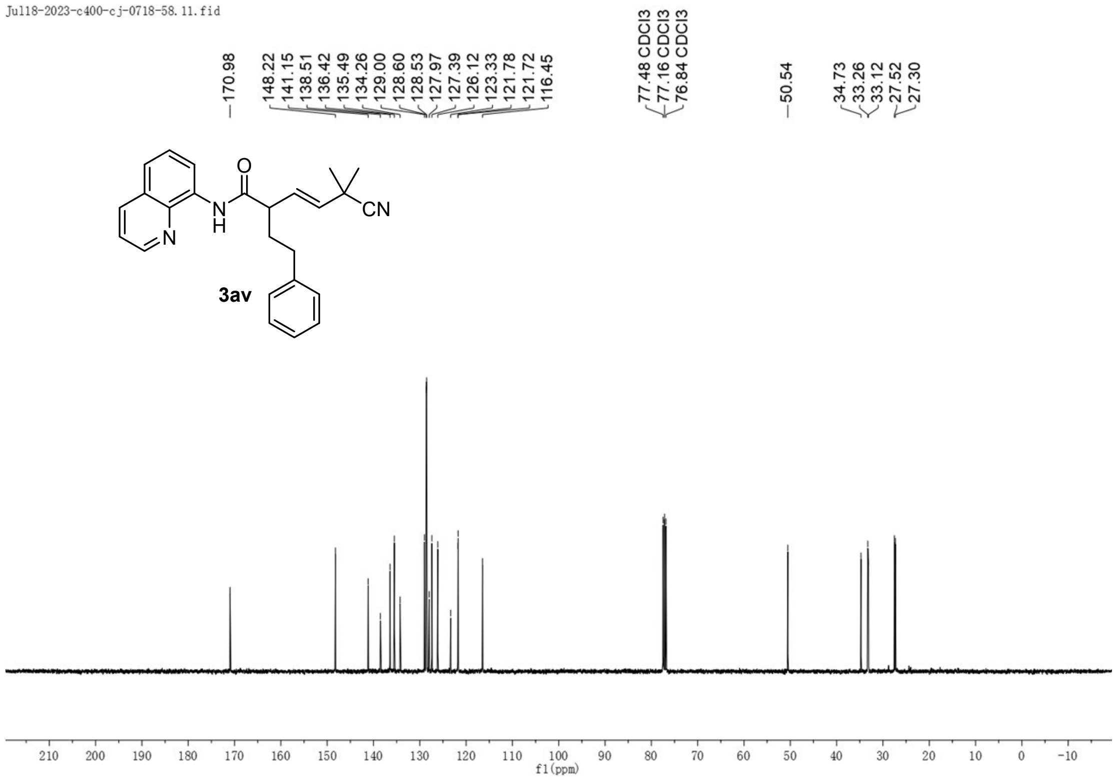

^1H NMR of 3aw ($CDCl_3$, 400 MHz)

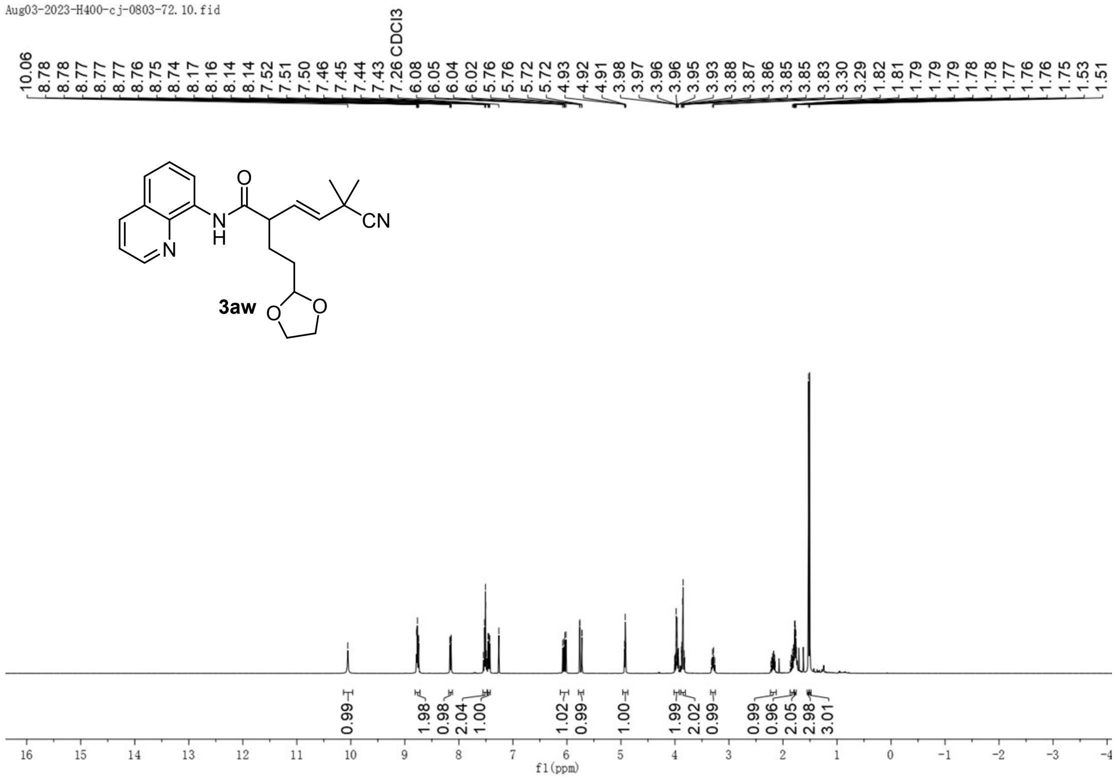

^{13}C NMR of 3aw ($CDCl_3$, 101 MHz)

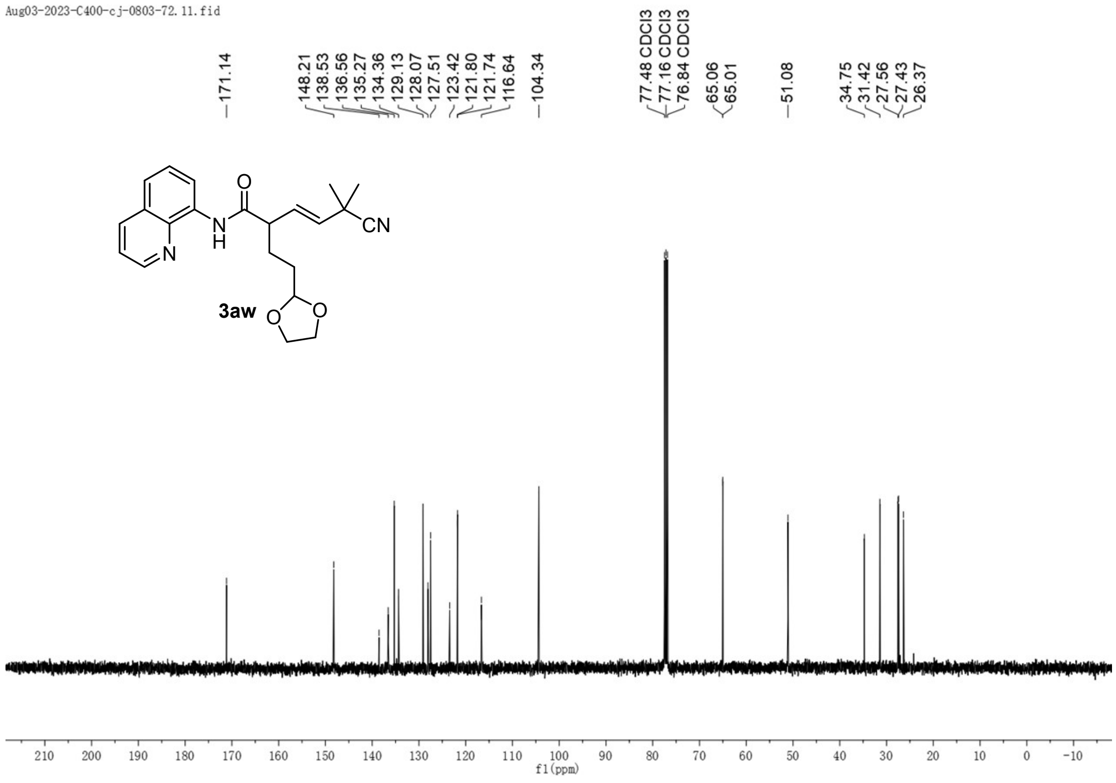

^{1}H NMR of 3ax ($CDCl_3$, 400 MHz)

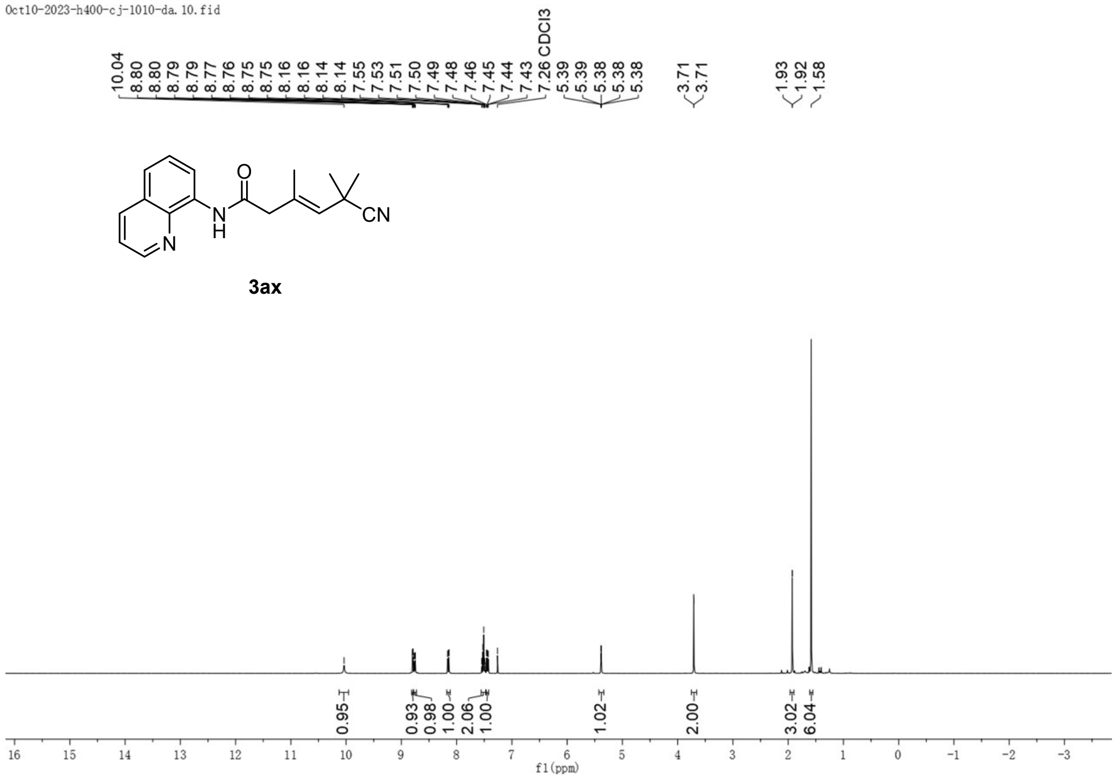

^{13}C NMR of 3ax ($CDCl_3$, 101 MHz)

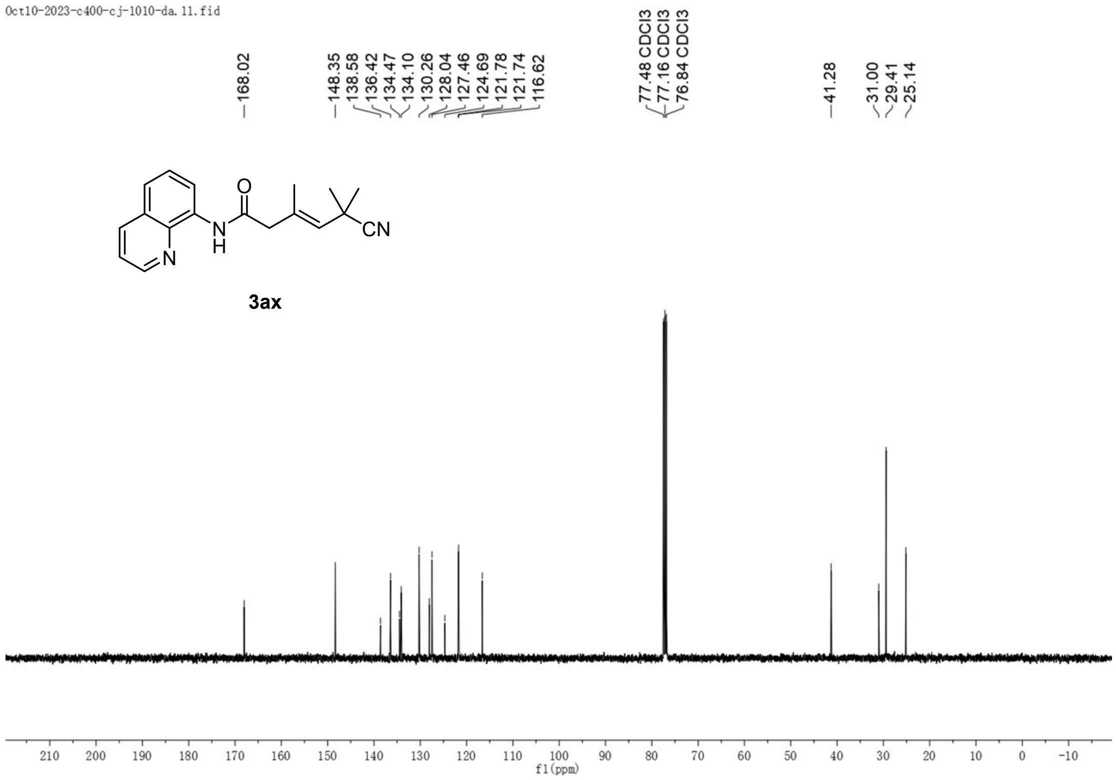

^{1}H NMR of 3ay' ($CDCl_3$, 400 MHz)

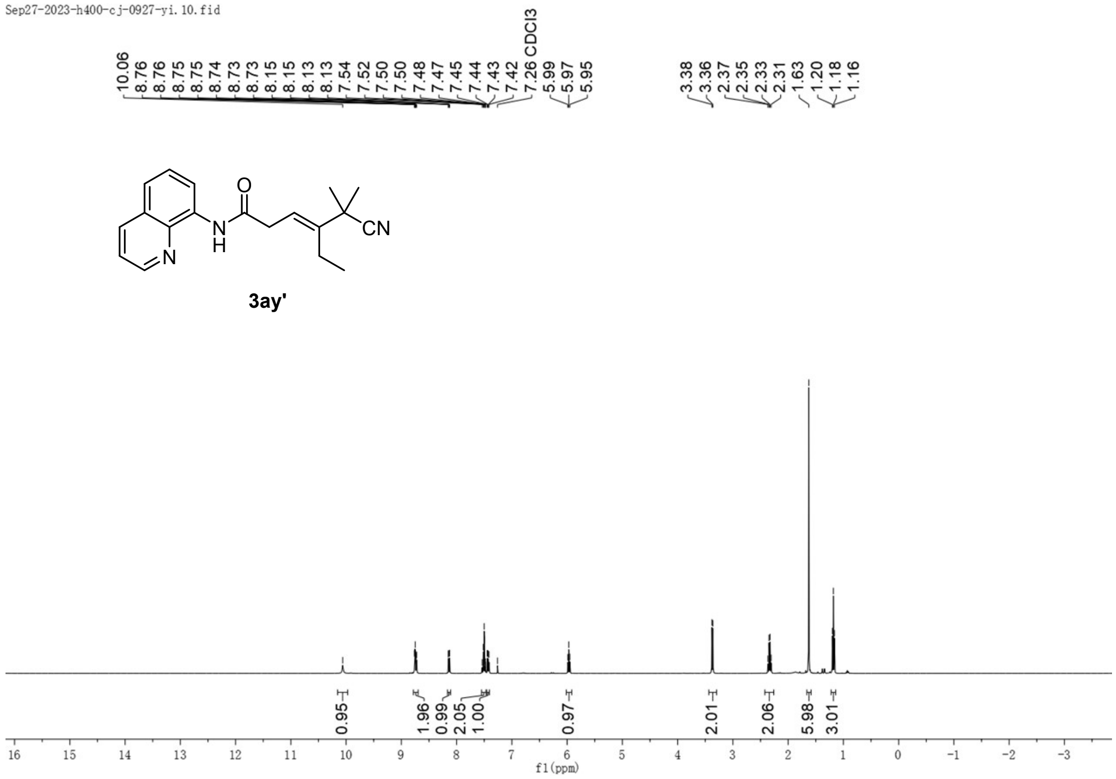

^{13}C NMR of 3ay' ($CDCl_3$, 101 MHz)

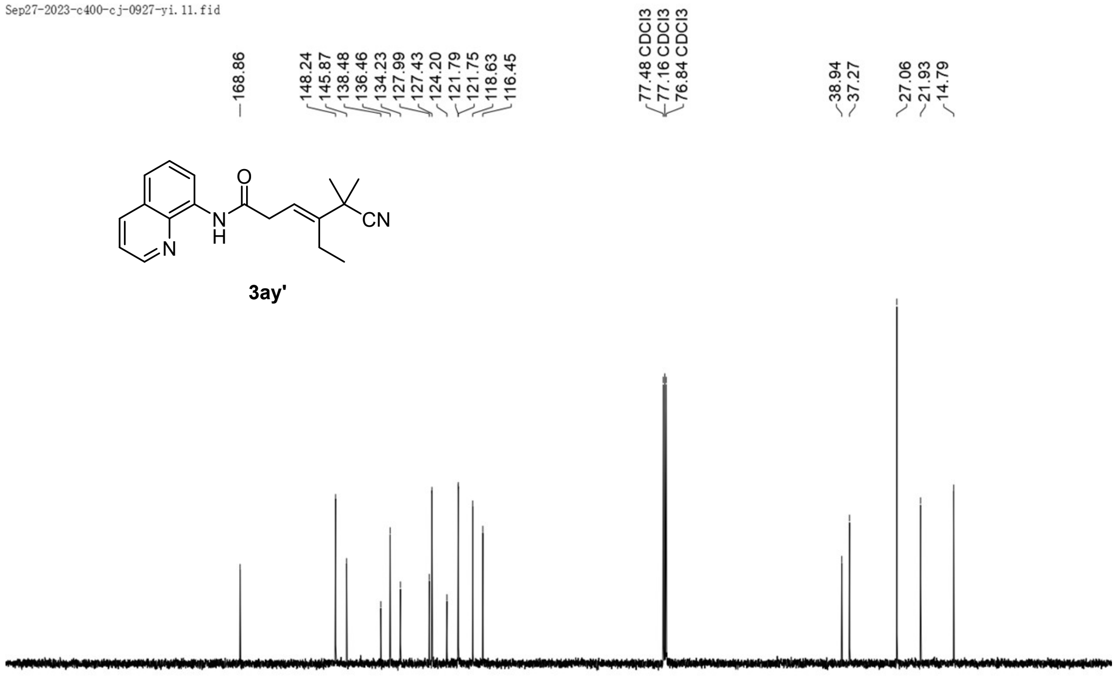

^{1}H NMR of 3az ($CDCl_3$, 400 MHz)

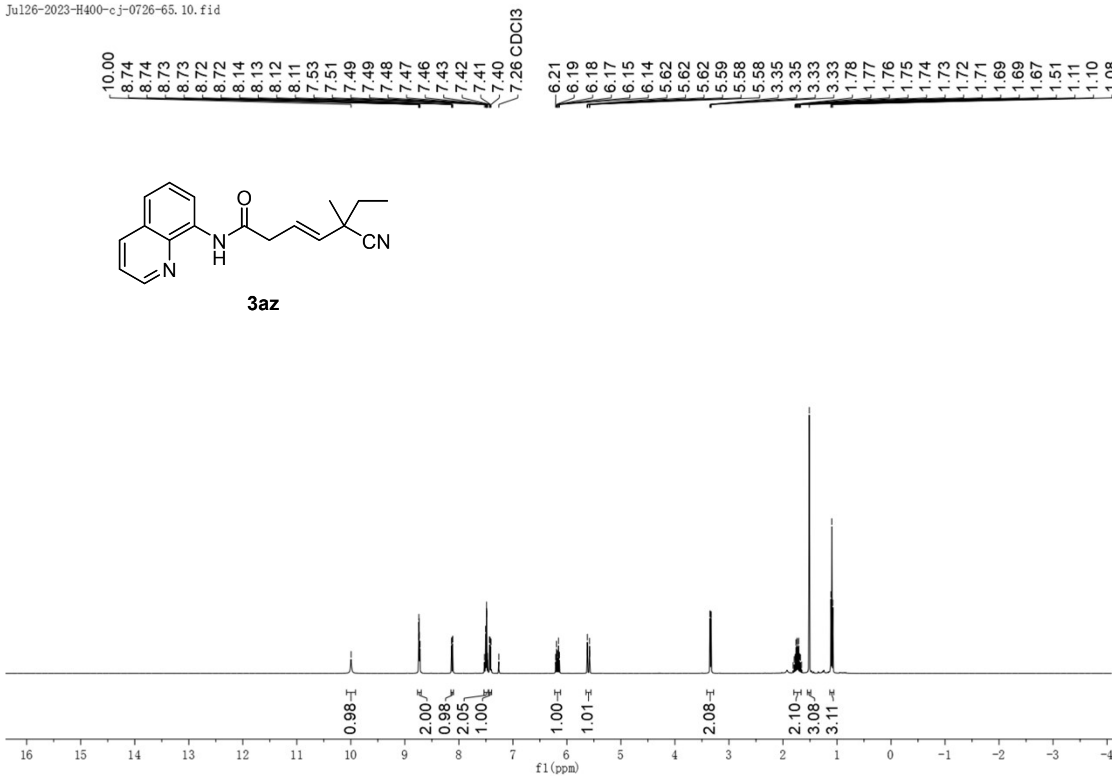

^{13}C NMR of 3az ($CDCl_3$, 101 MHz)

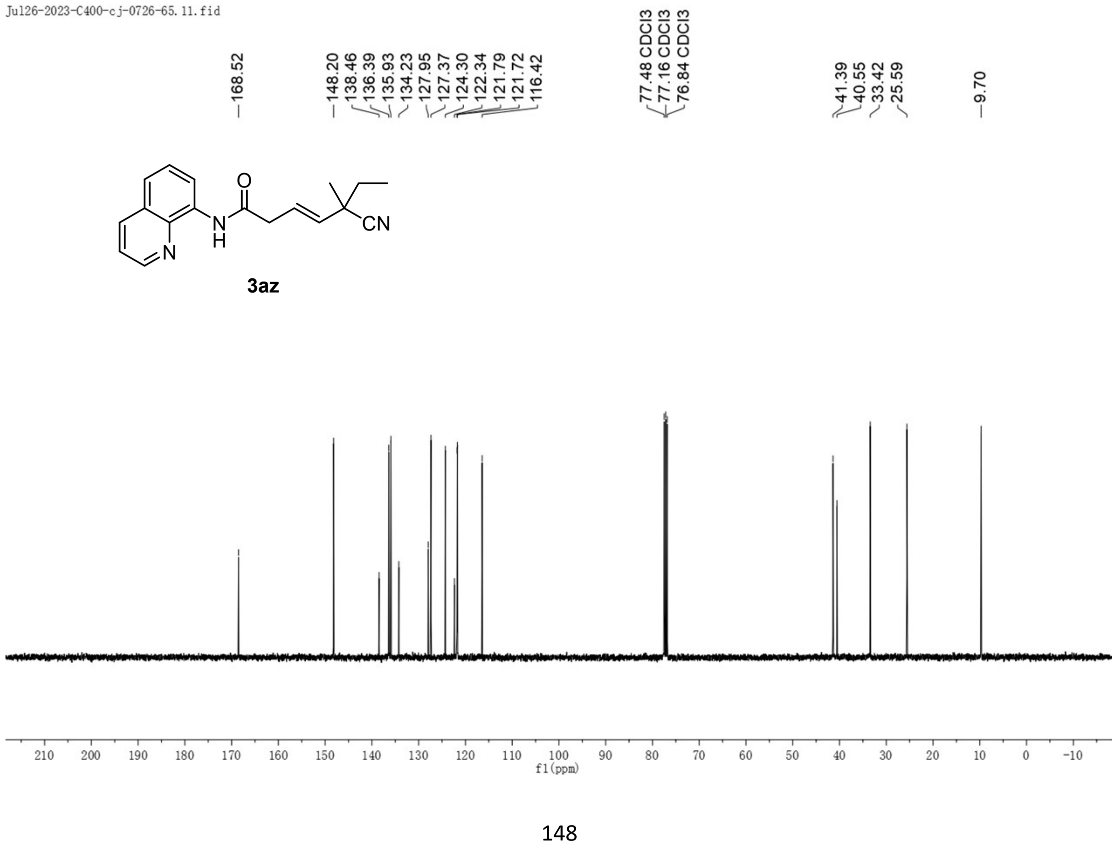

^1H NMR of 3az' ($CDCl_3$, 400 MHz)

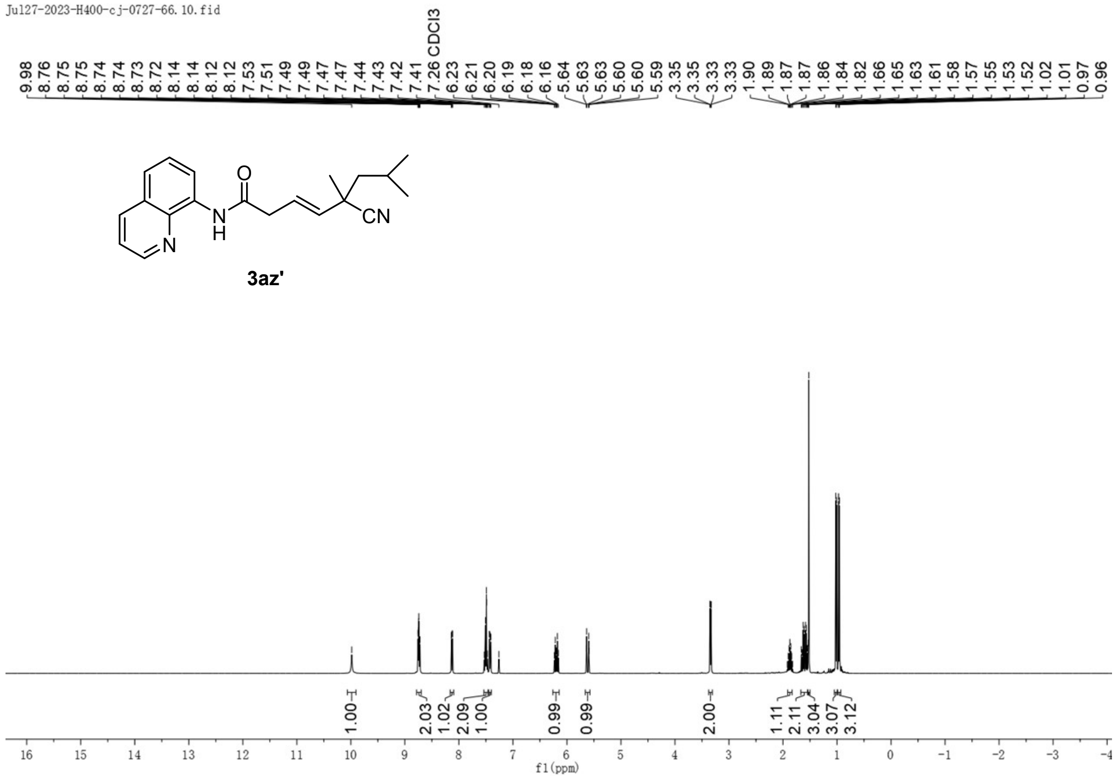

^{13}C NMR of 3az' ($CDCl_3$, 101 MHz)

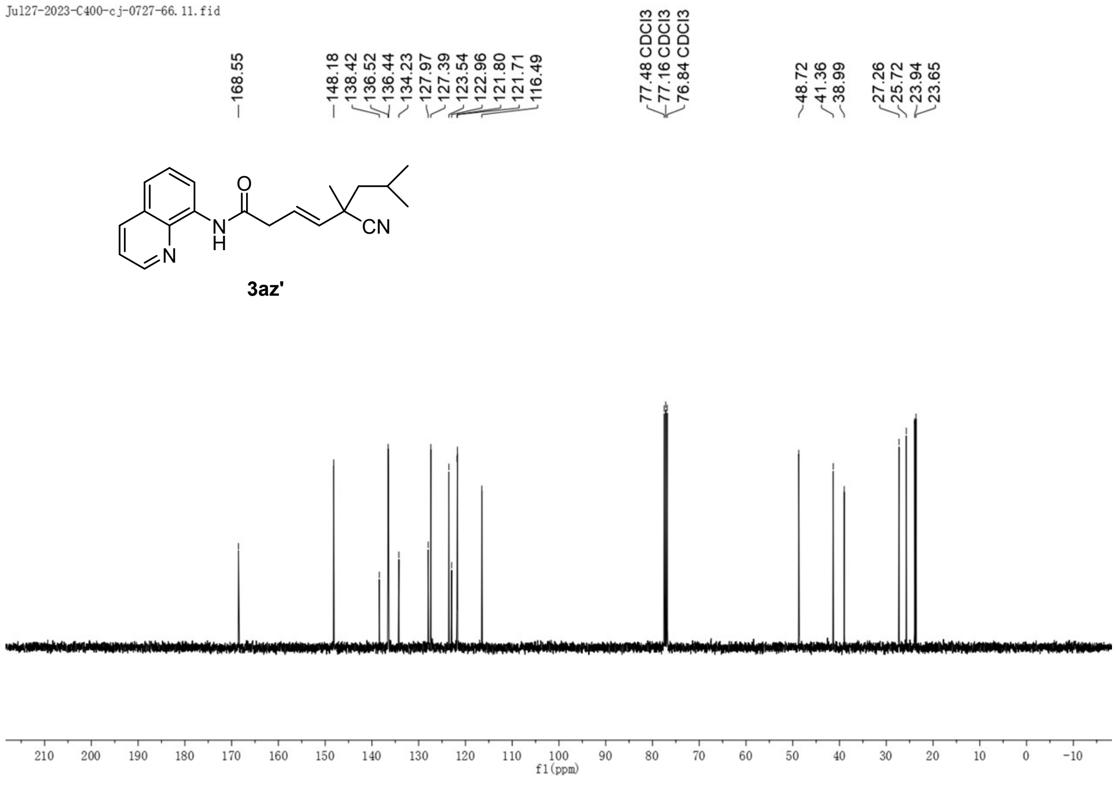

^{1}H NMR of 4aa ($CDCl_3$, 400 MHz)

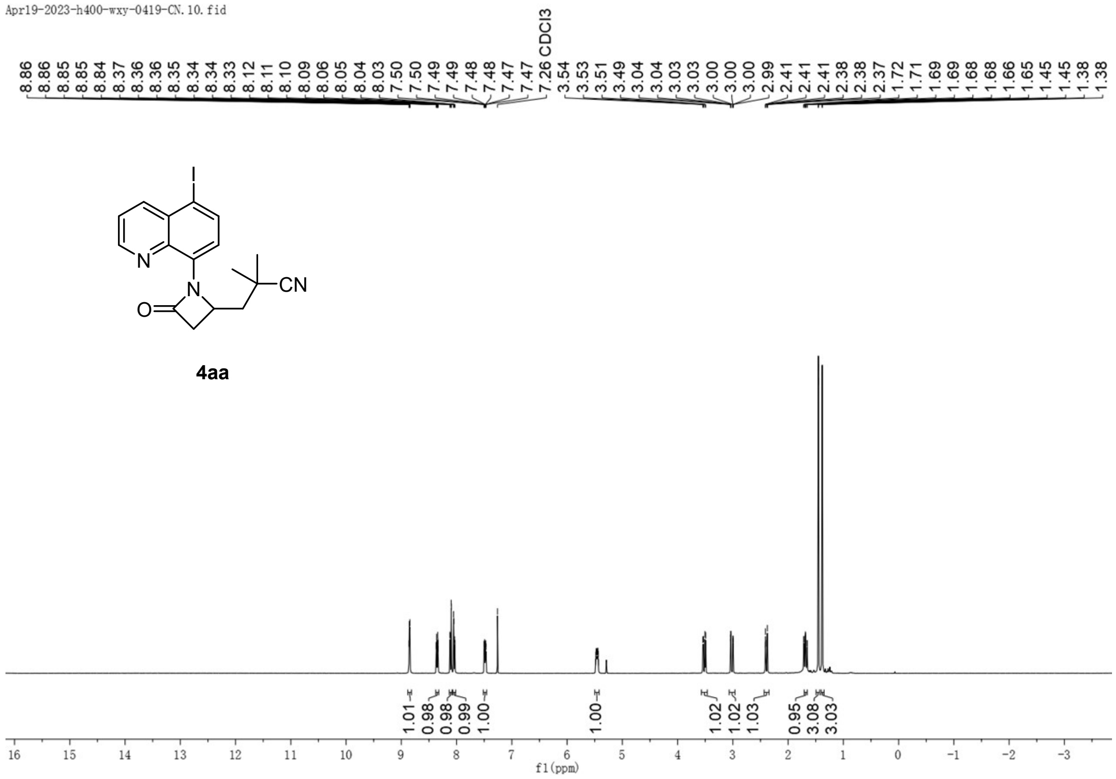

^{13}C NMR of 4aa ($CDCl_3$, 101 MHz)

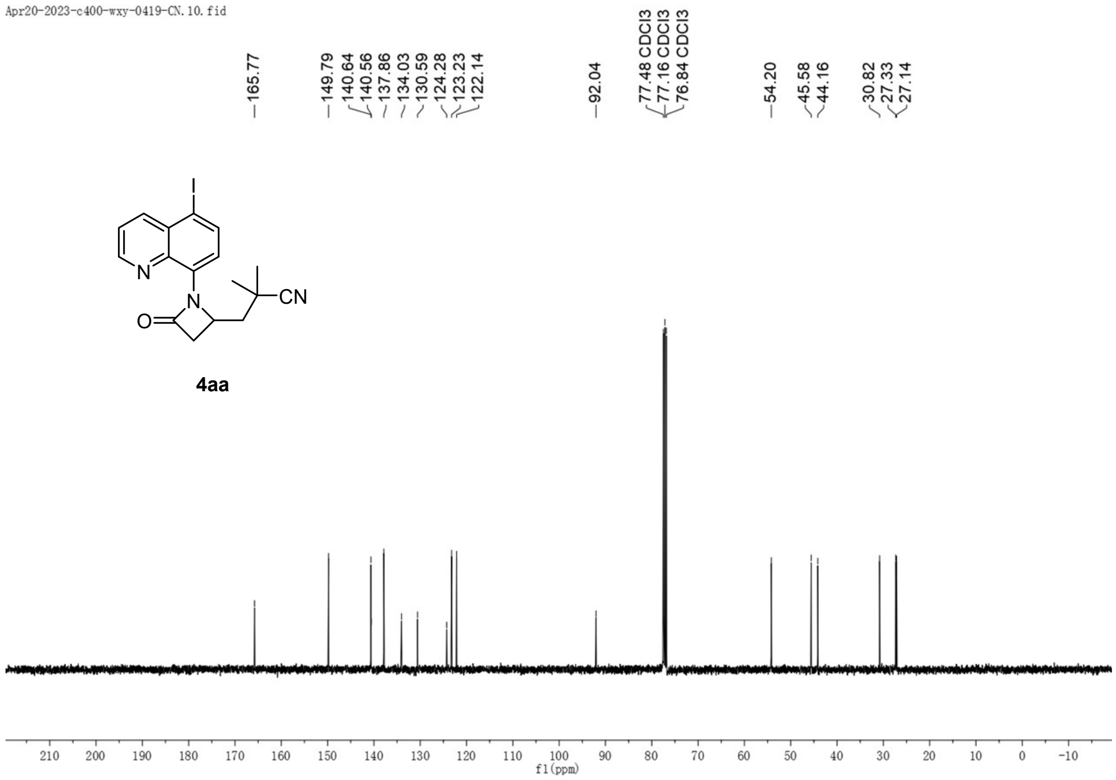

^{1}H NMR of 4ab ($CDCl_3$, 400 MHz)

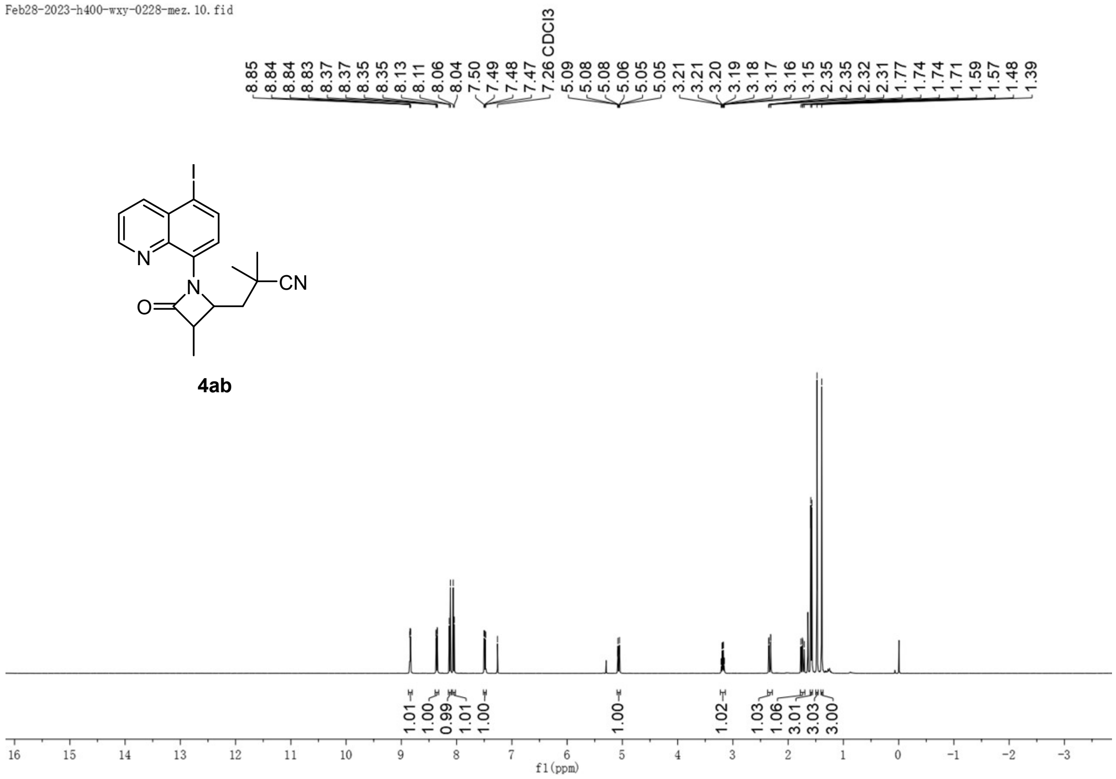

^{13}C NMR of 4ab ($CDCl_3$, 101 MHz)

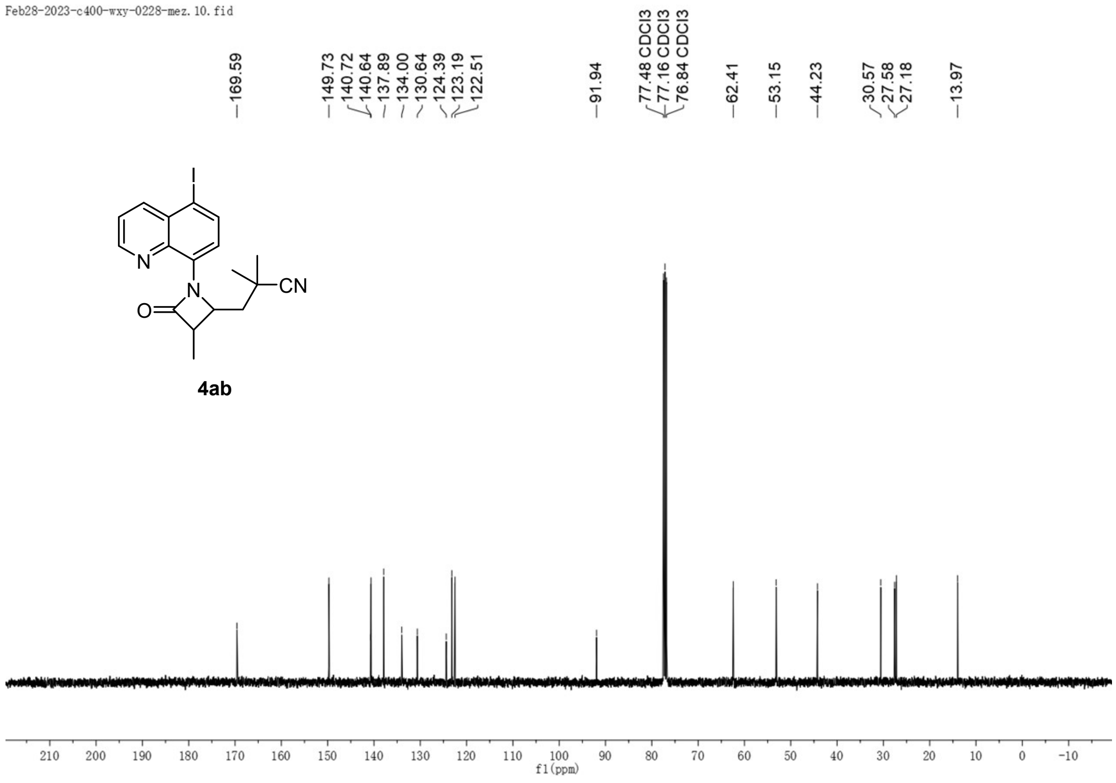

^{1}H NMR of 4ac ($CDCl_3$, 400 MHz)

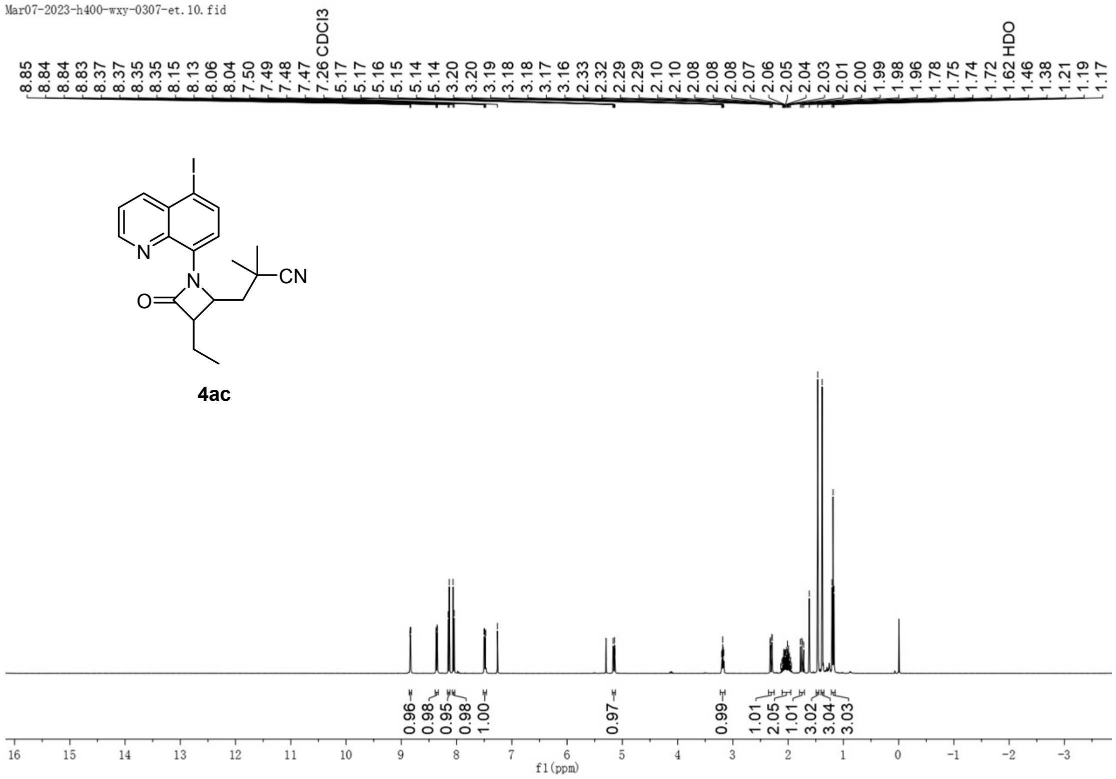

^{13}C NMR of 4ac ($CDCl_3$, 101 MHz)

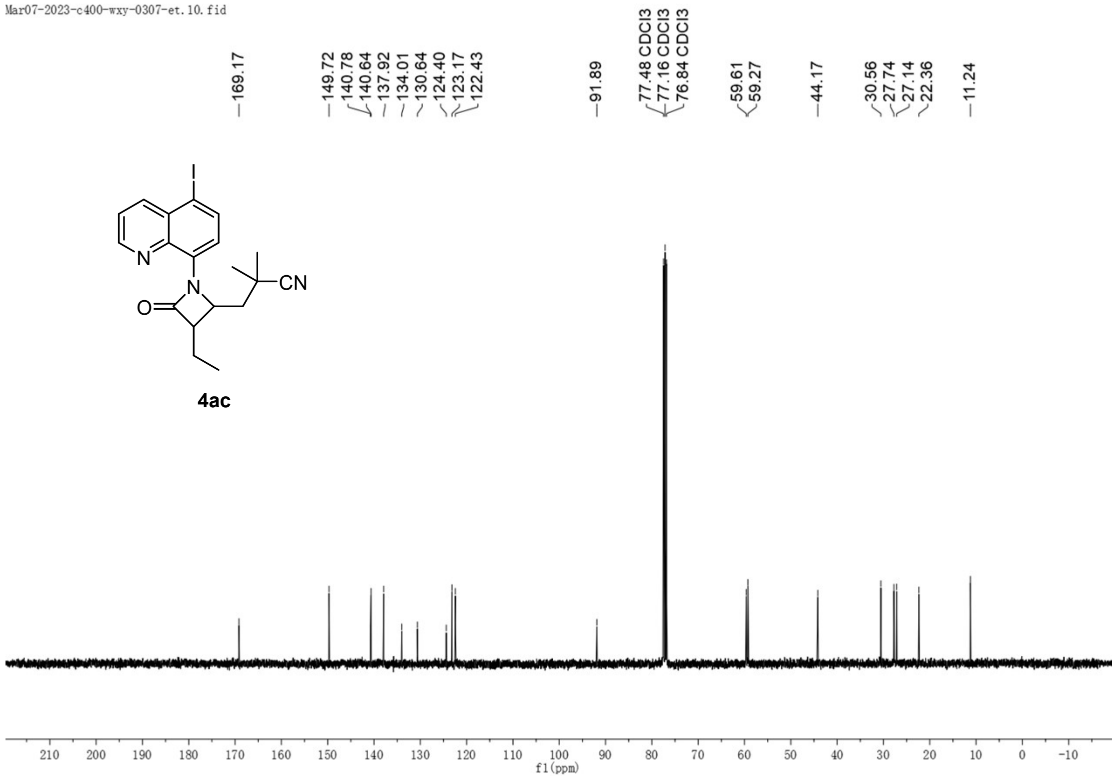

^{1}H NMR of 4ad ($CDCl_3$, 400 MHz)

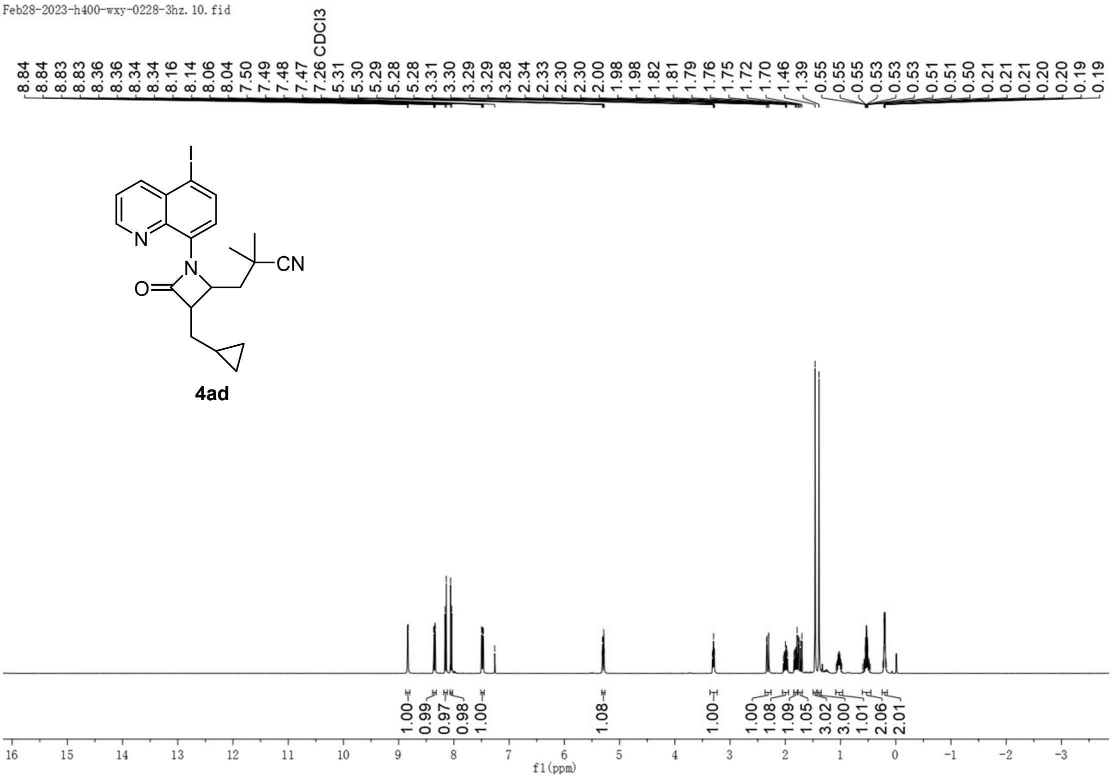

^{13}C NMR of 4ad ($CDCl_3$, 101 MHz)

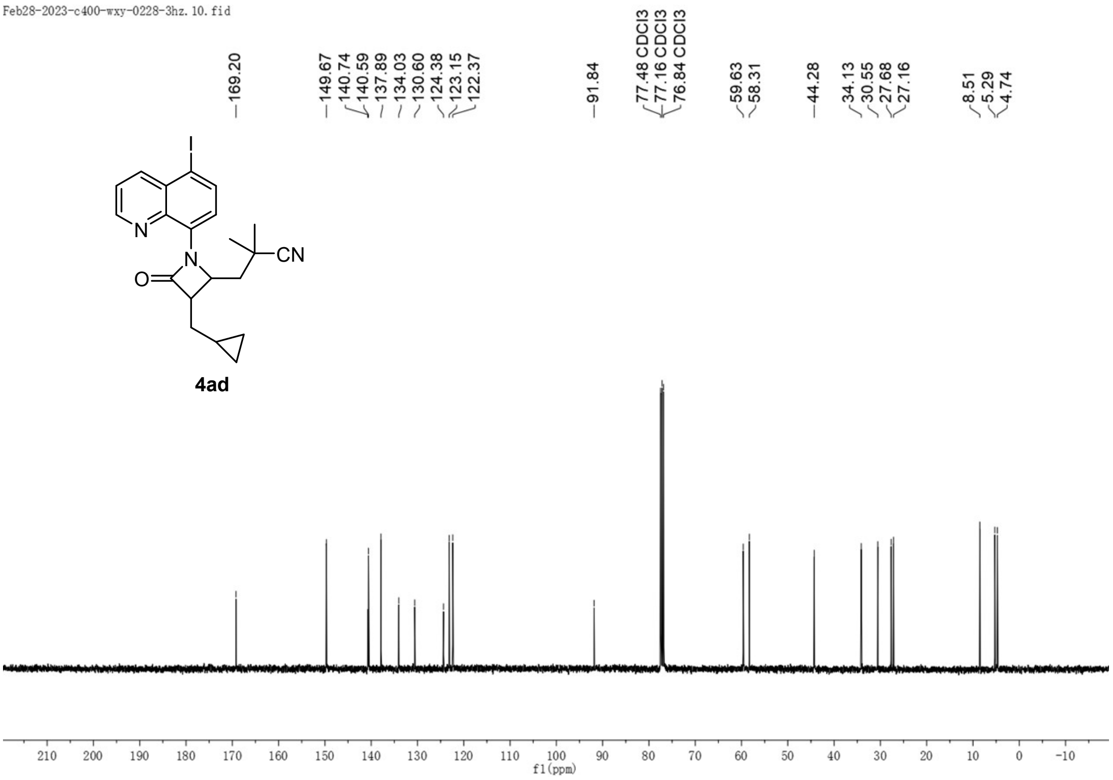

^{1}H NMR of 4ae ($CDCl_3$, 400 MHz)

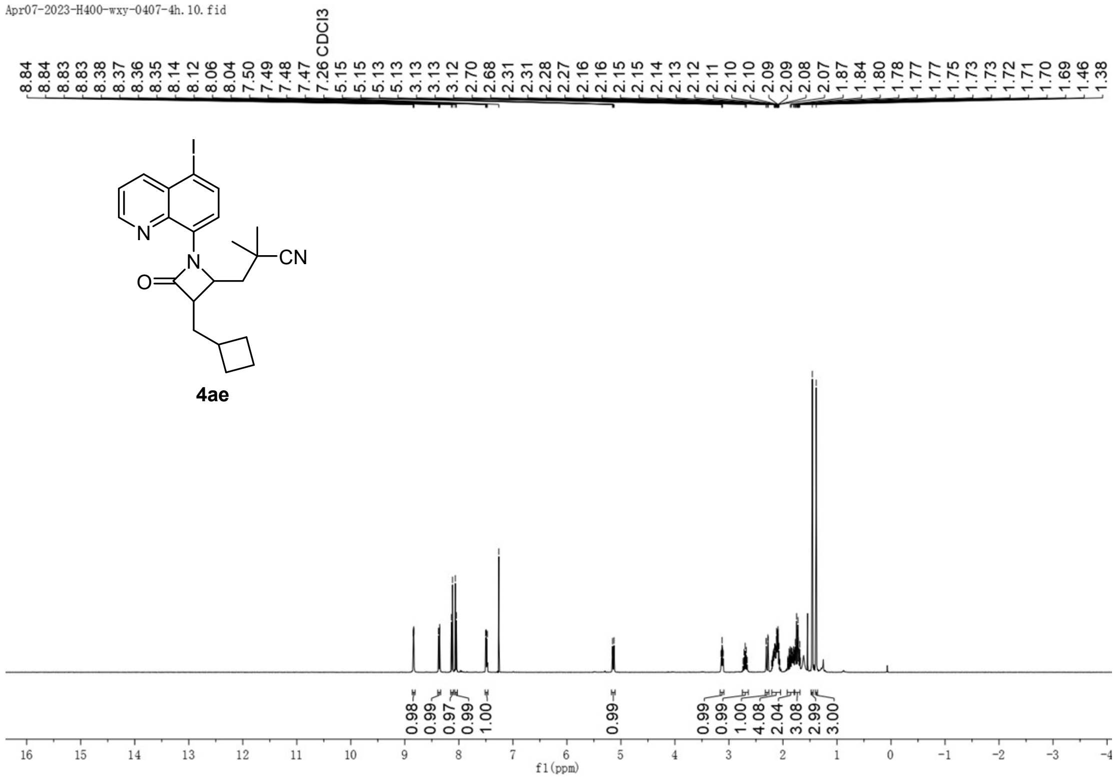

^{13}C NMR of 4ae ($CDCl_3$, 101 MHz)

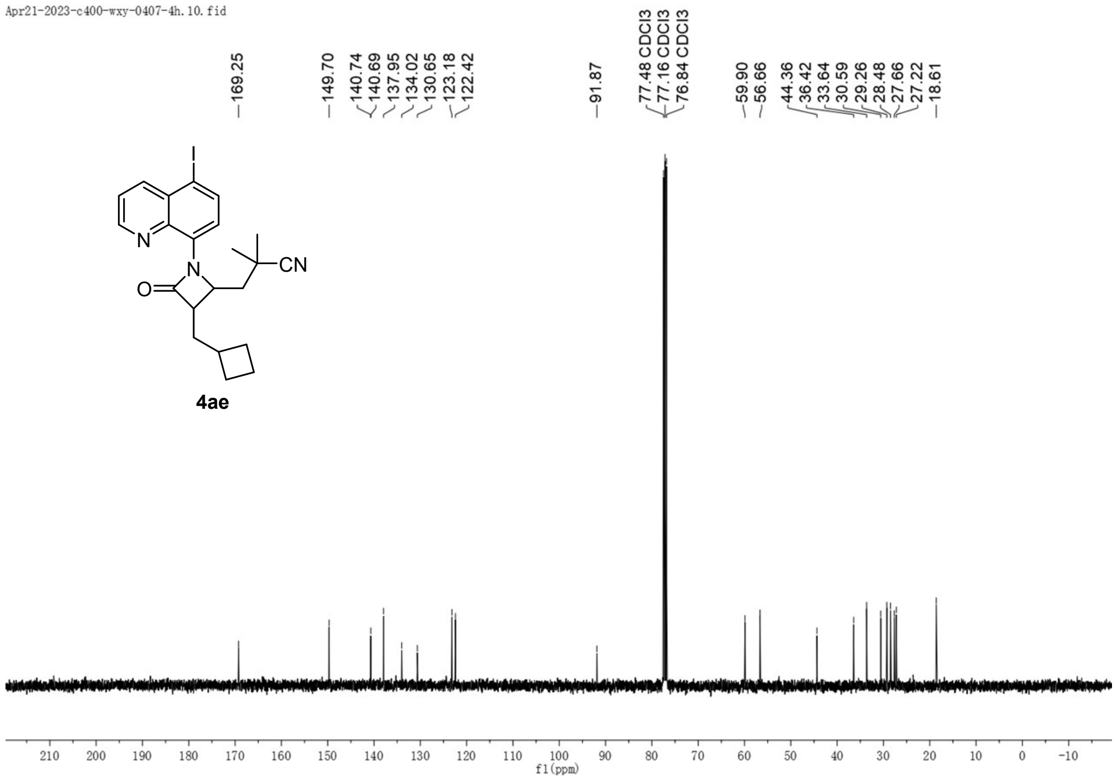

^{1}H NMR of 4af ($CDCl_3$, 400 MHz)

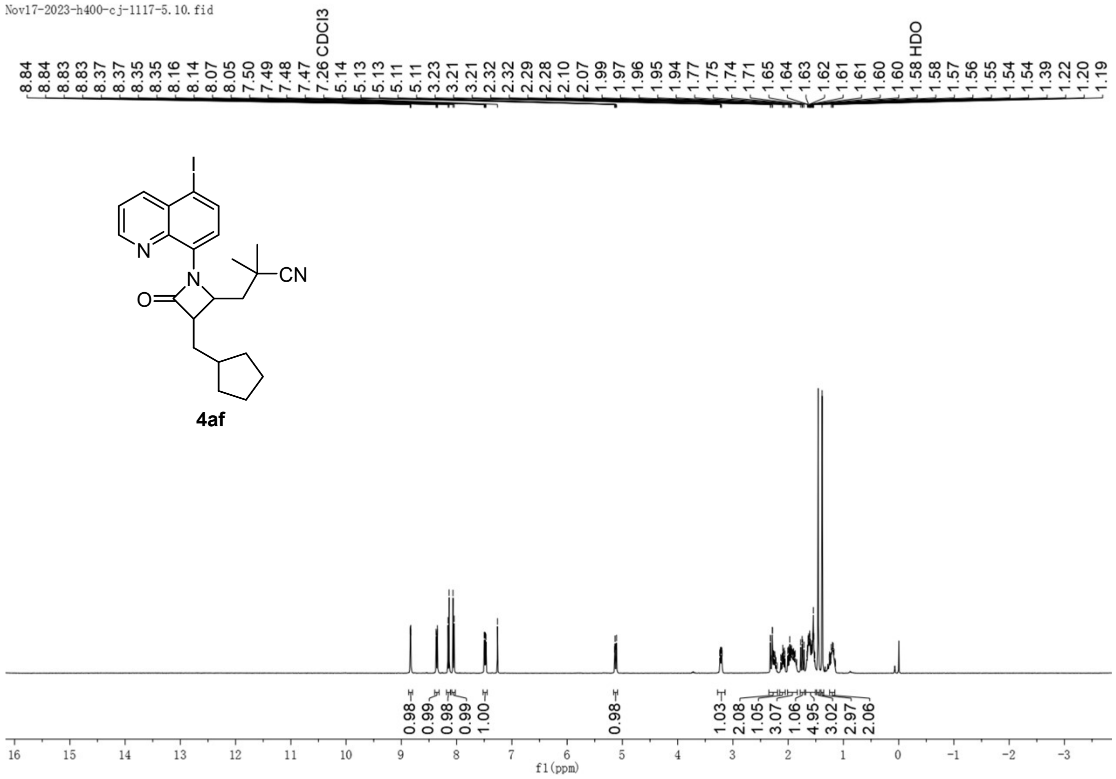

^{13}C NMR of 4af ($CDCl_3$, 101 MHz)

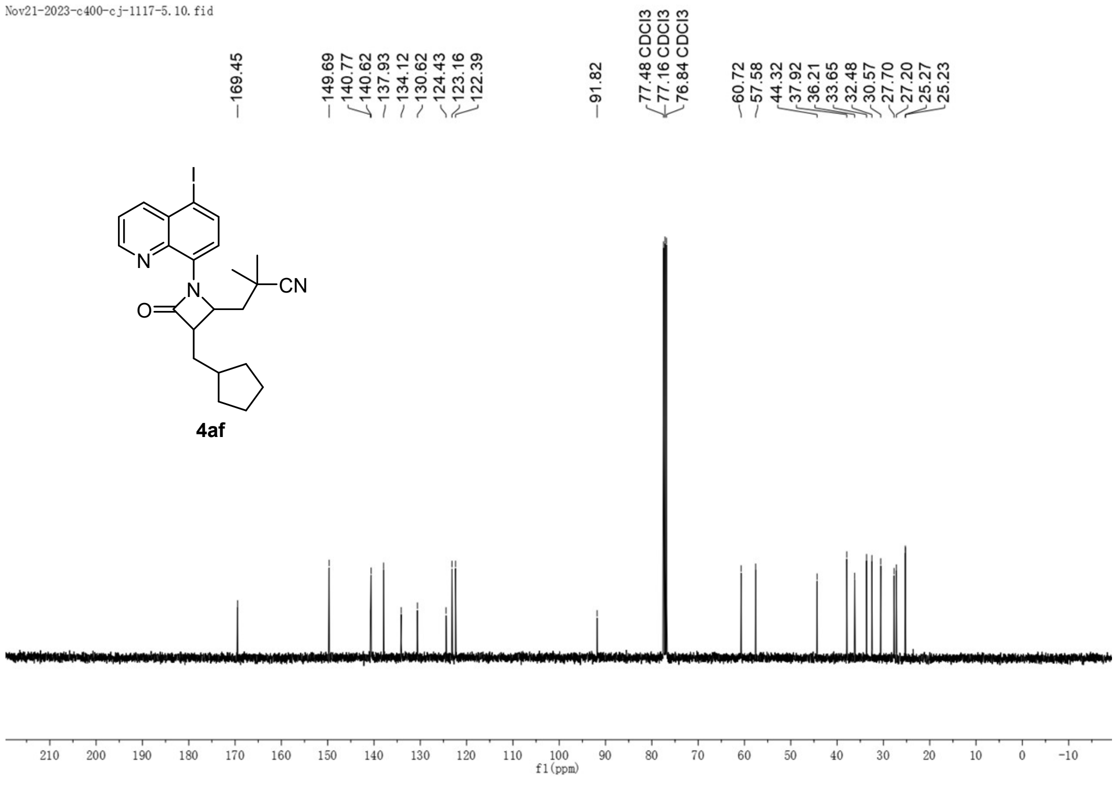

^{1}H NMR of 4ag ($CDCl_3$, 400 MHz)

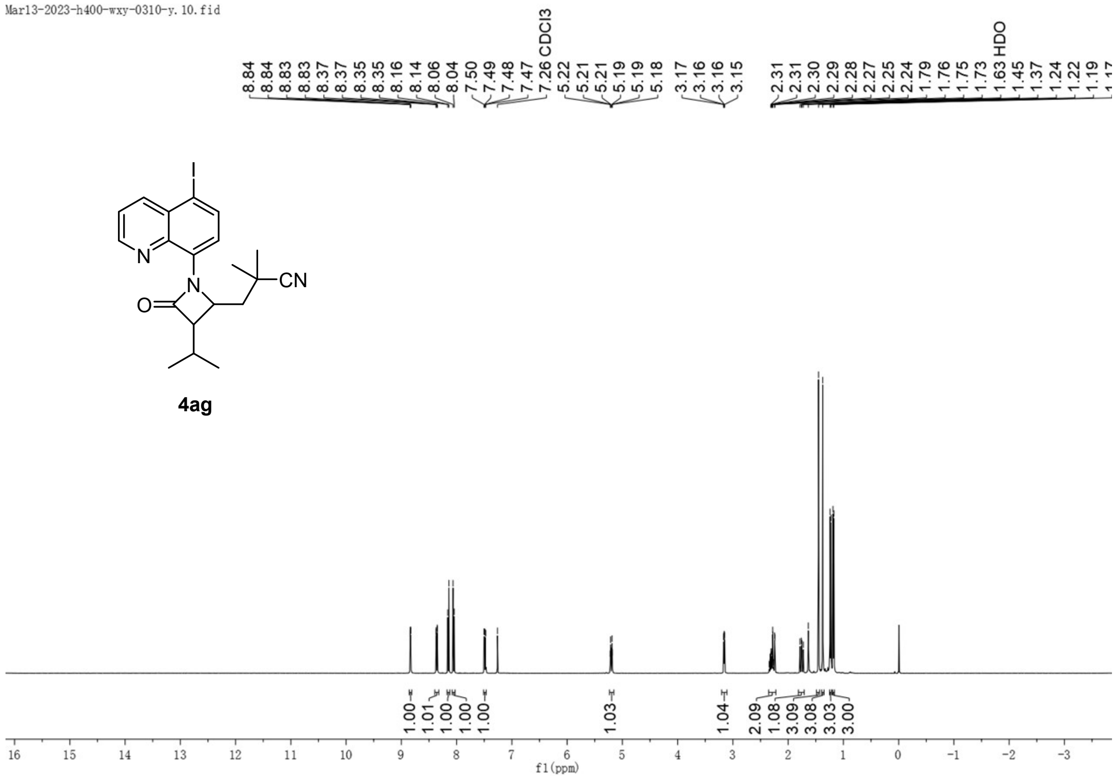

^{13}C NMR of 4ag ($CDCl_3$, 101 MHz)

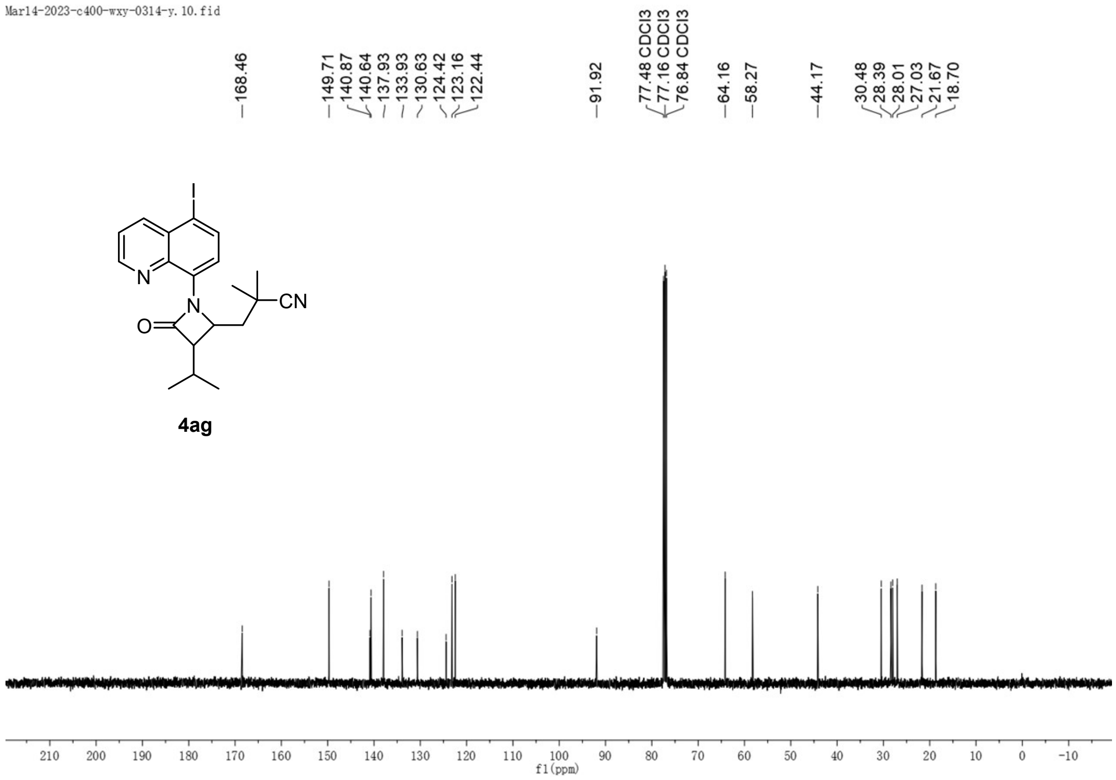

^{1}H NMR of 4ah ($CDCl_3$, 400 MHz)

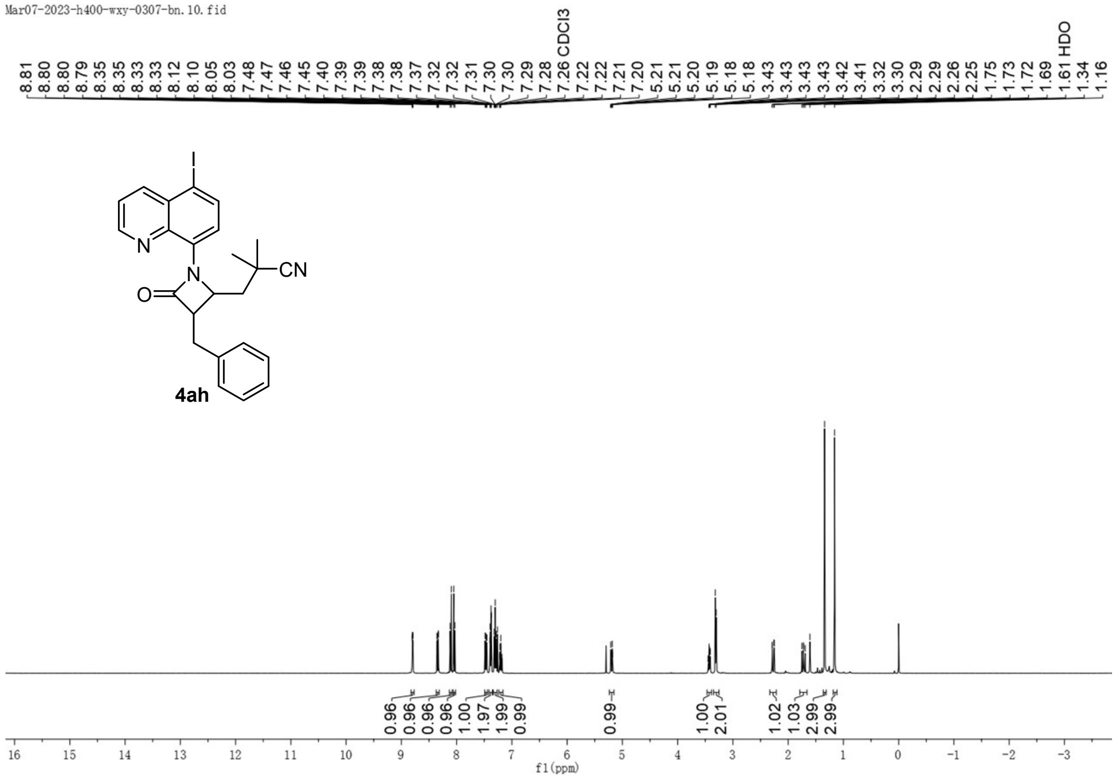

^{13}C NMR of 4ah ($CDCl_3$, 101 MHz)

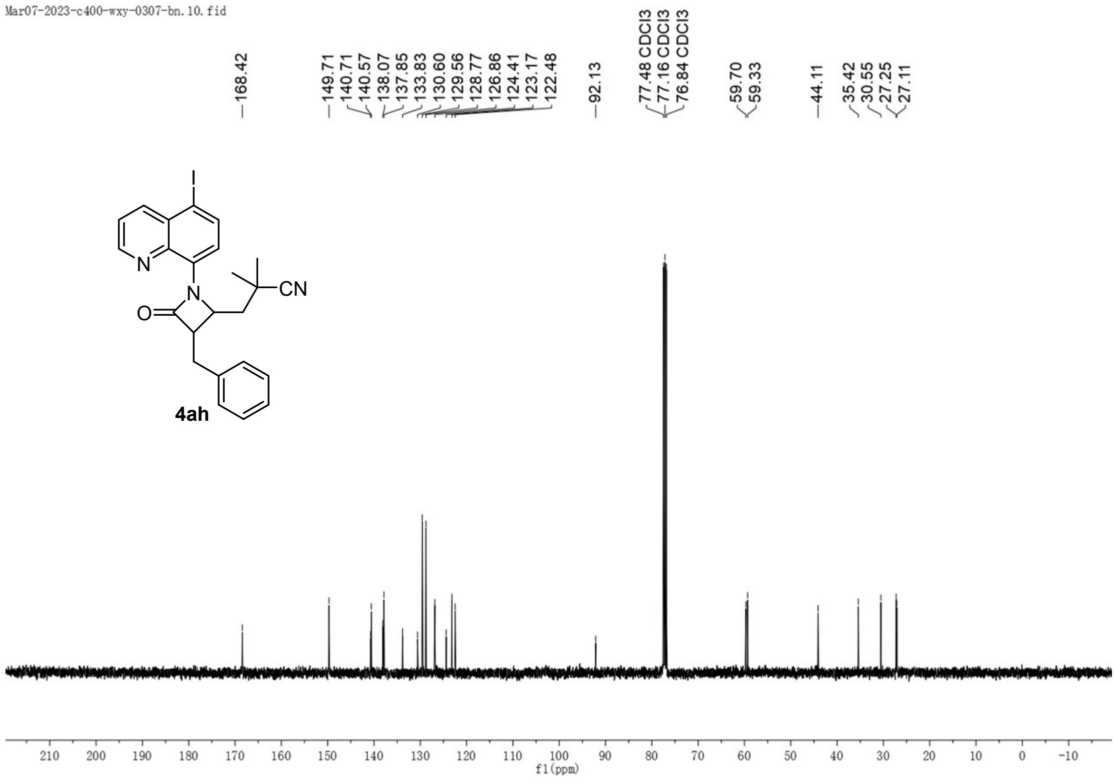

^{1}H NMR of 4ai ($CDCl_3$, 400 MHz)

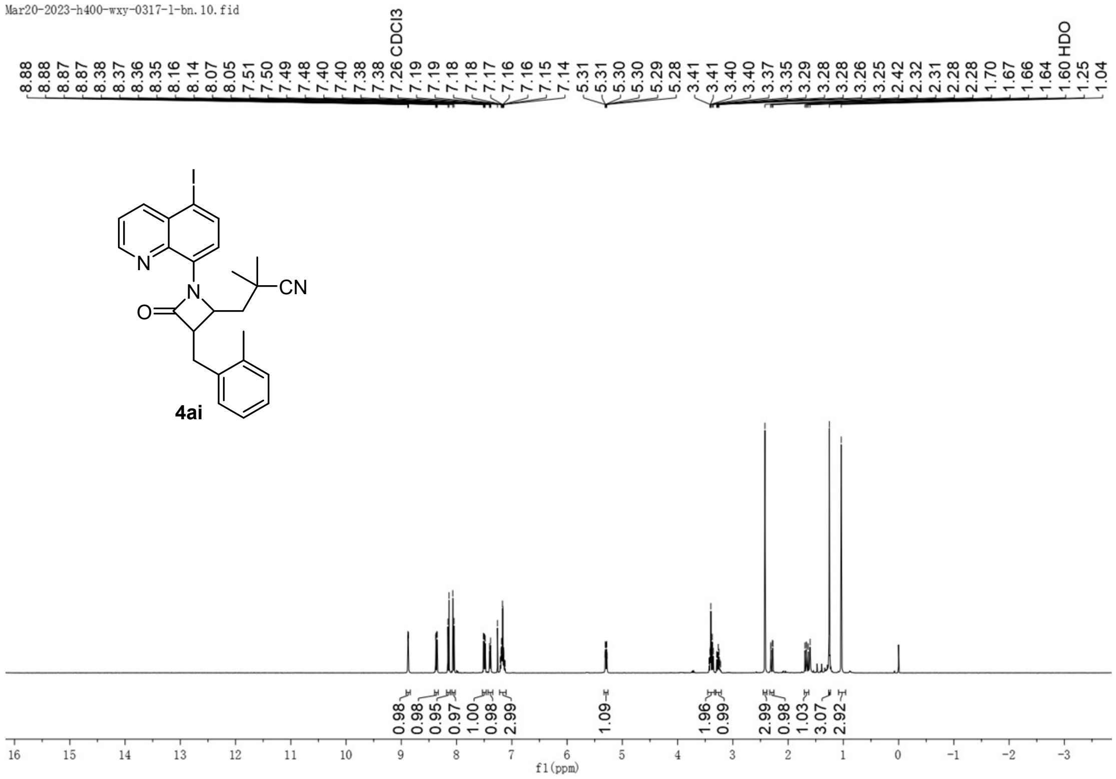

^{13}C NMR of 4ai ($CDCl_3$, 101 MHz)

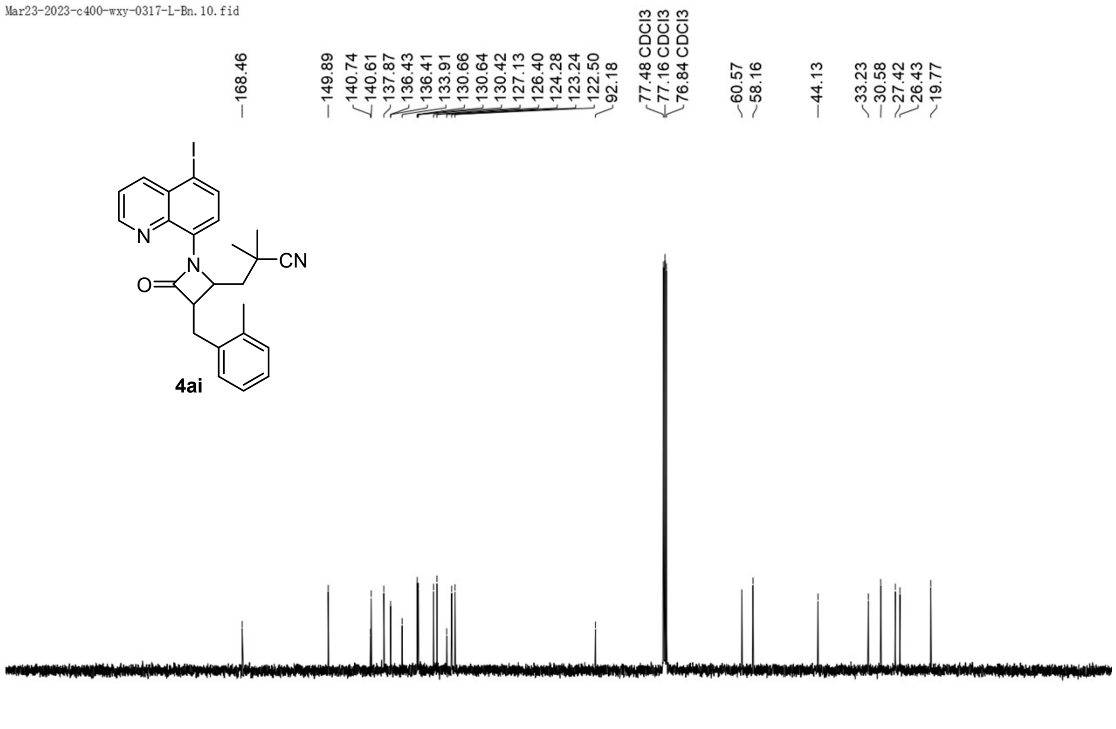

^{1}H NMR of 4aj ($CDCl_3$, 400 MHz)

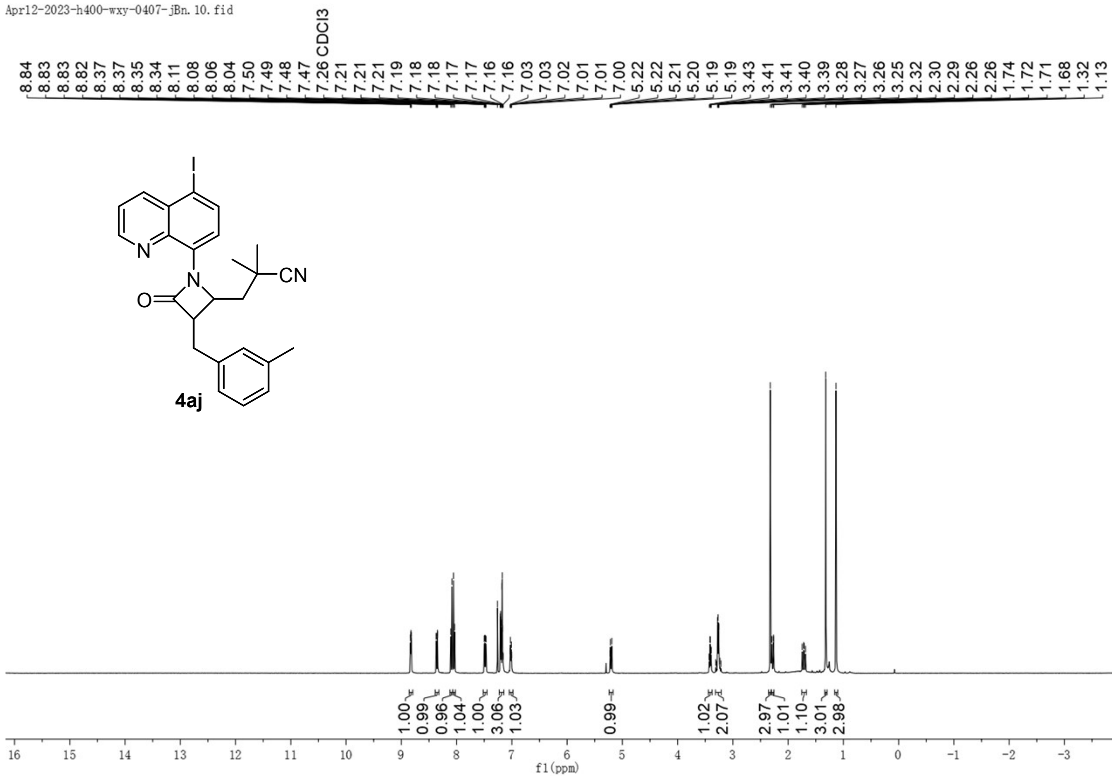

^{13}C NMR of 4aj ($CDCl_3$, 101 MHz)

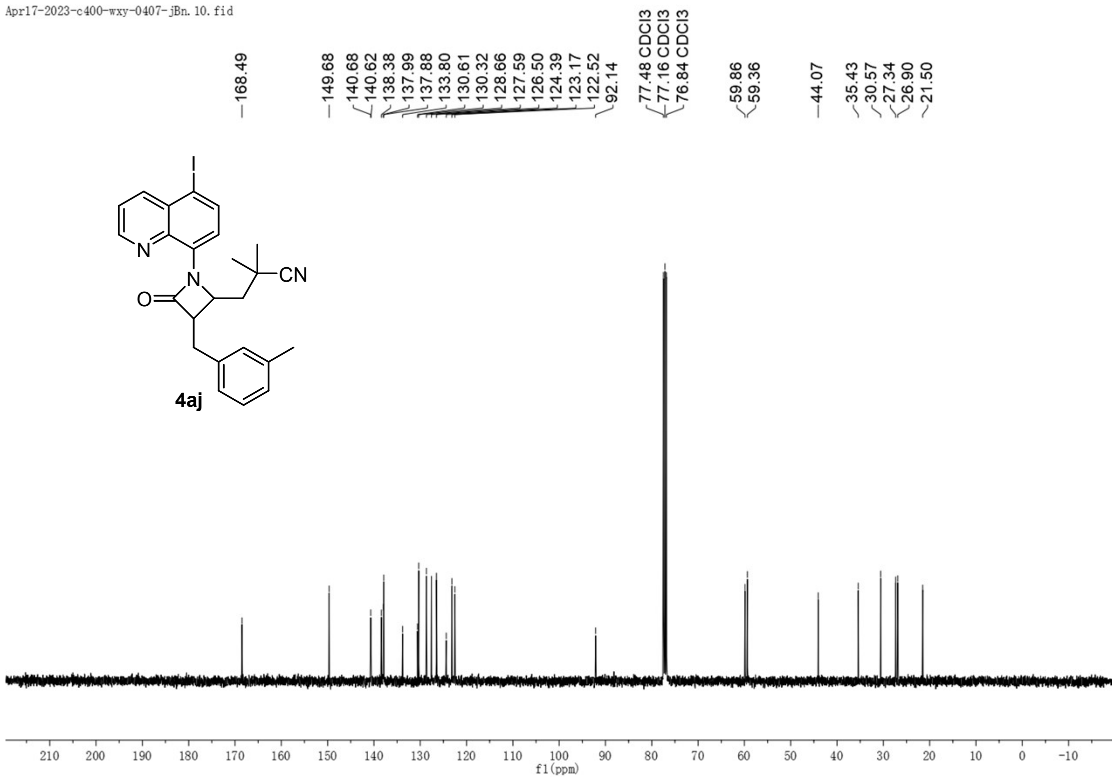

^{1}H NMR of 4ak ($CDCl_3$, 400 MHz)

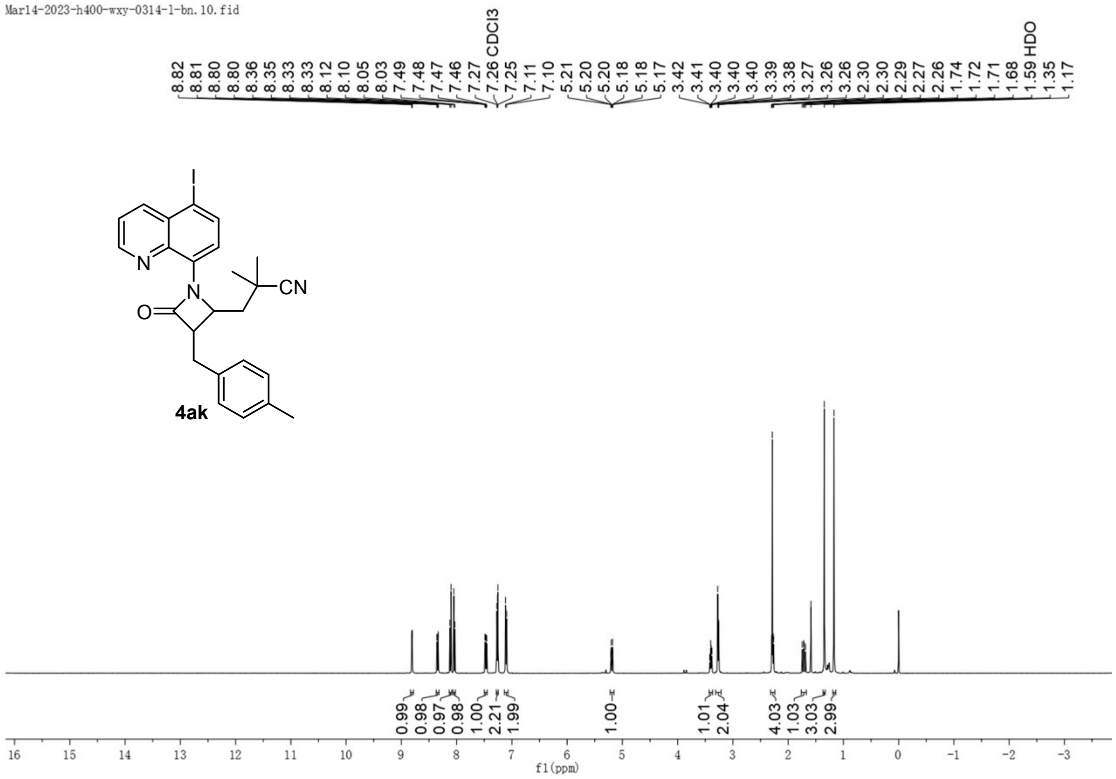

^{13}C NMR of 4ak ($CDCl_3$, 101 MHz)

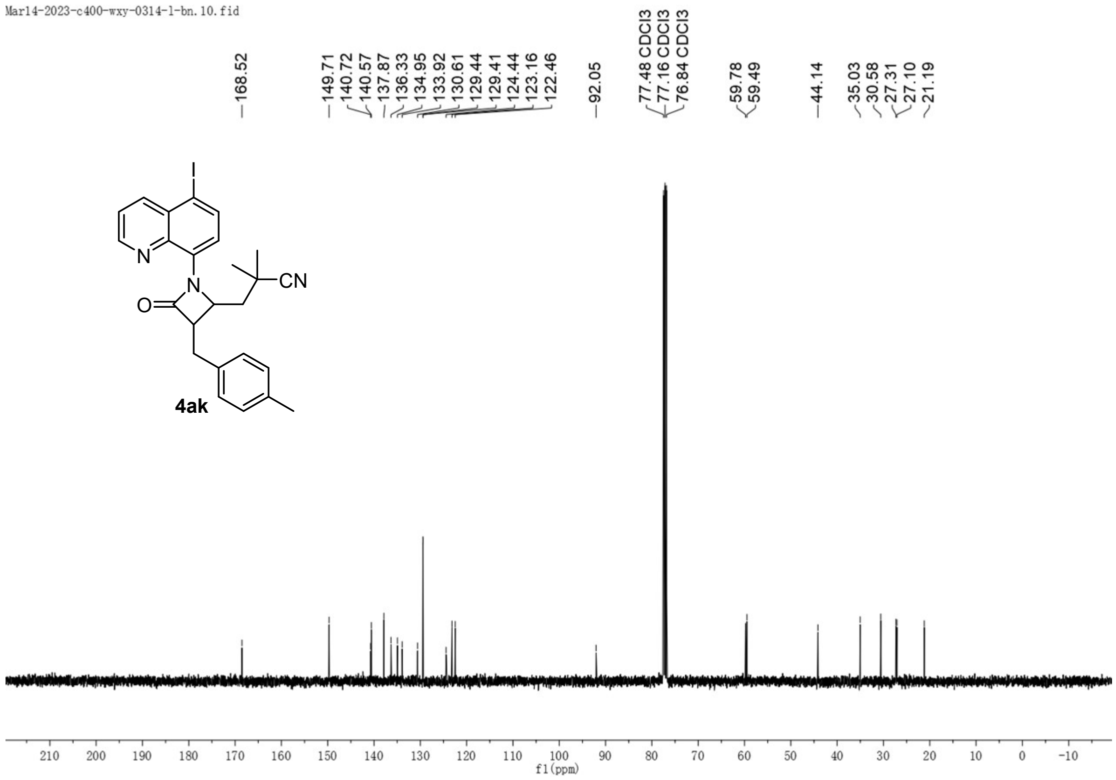

^{1}H NMR of 4al ($CDCl_3$, 400 MHz)

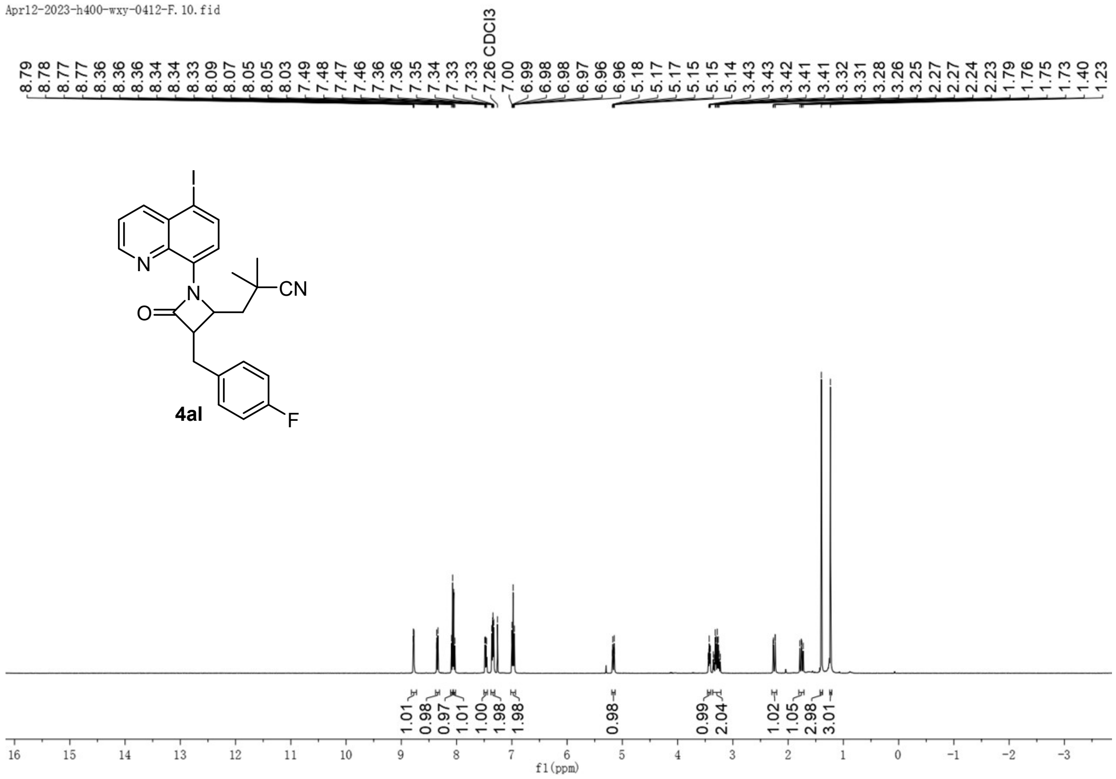

^{13}C NMR of 4al ($CDCl_3$, 101 MHz)

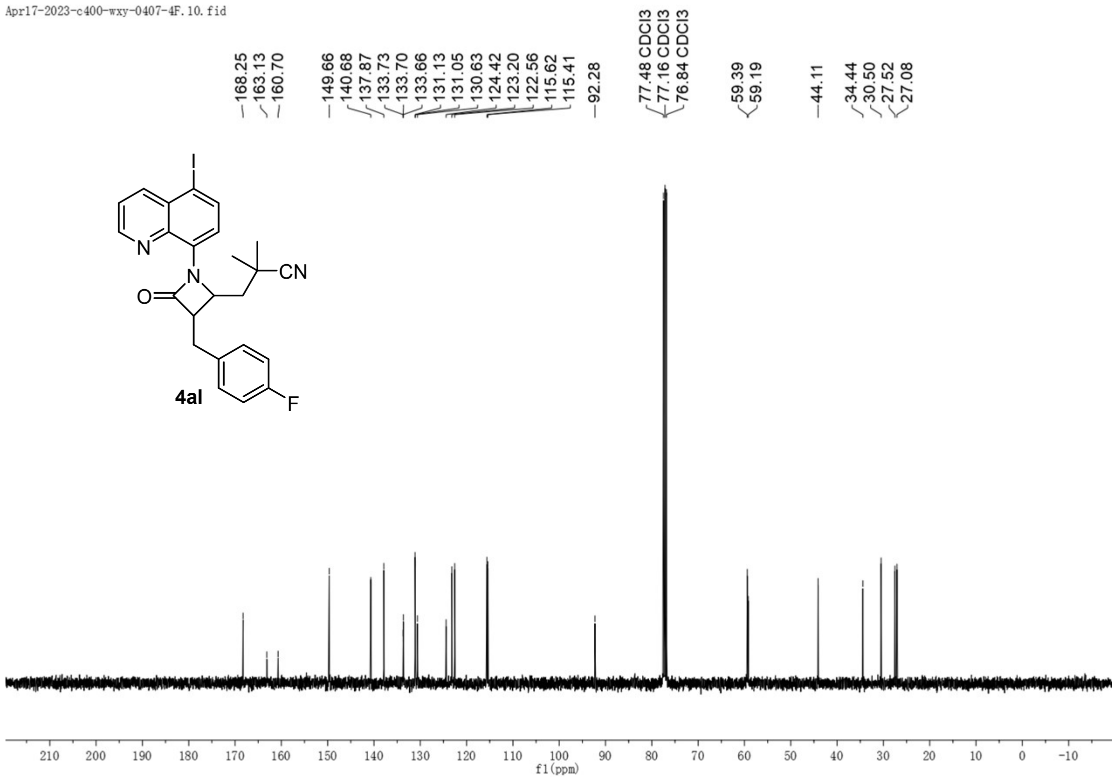

^{19}F NMR of 4al ($CDCl_3$, 376 MHz)

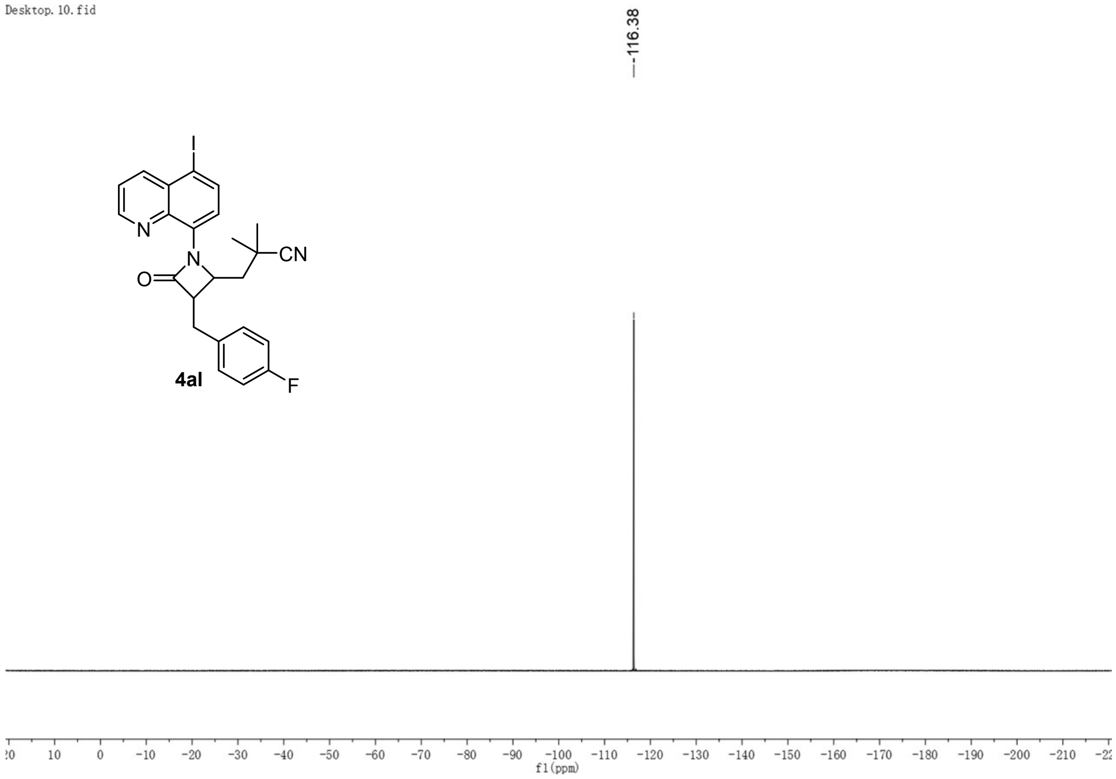

^{1}H NMR of 4am ($CDCl_3$, 400 MHz)

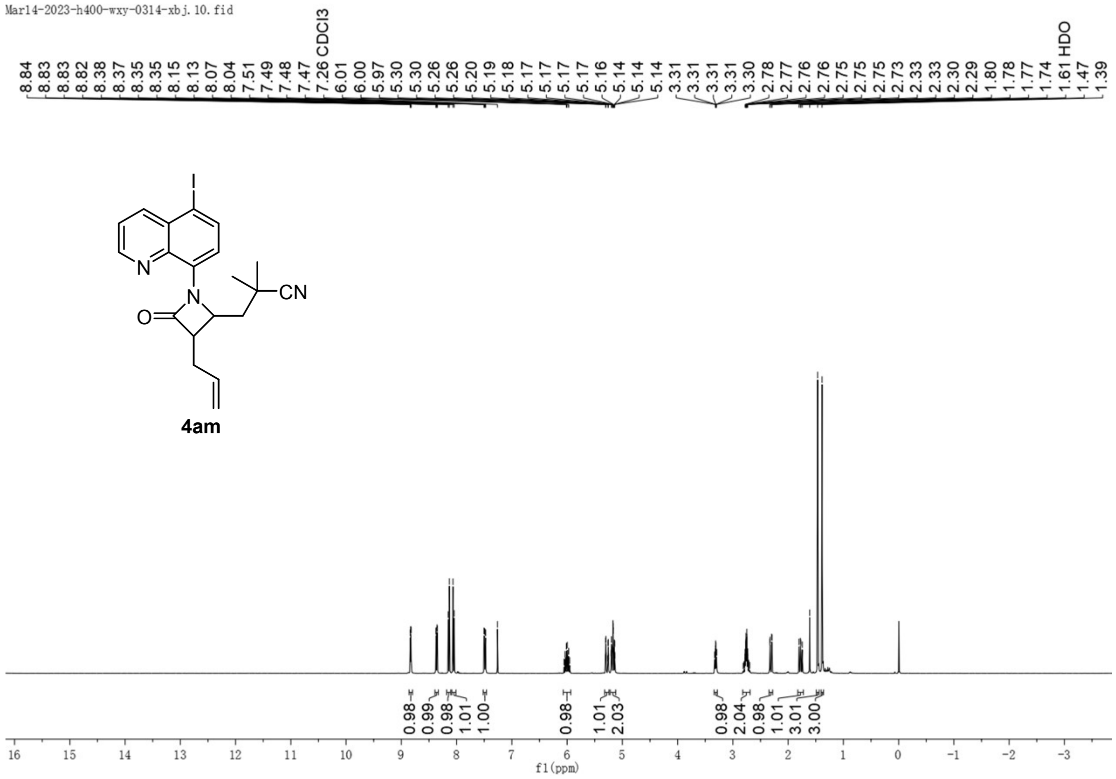

^{13}C NMR of 4am ($CDCl_3$, 101 MHz)

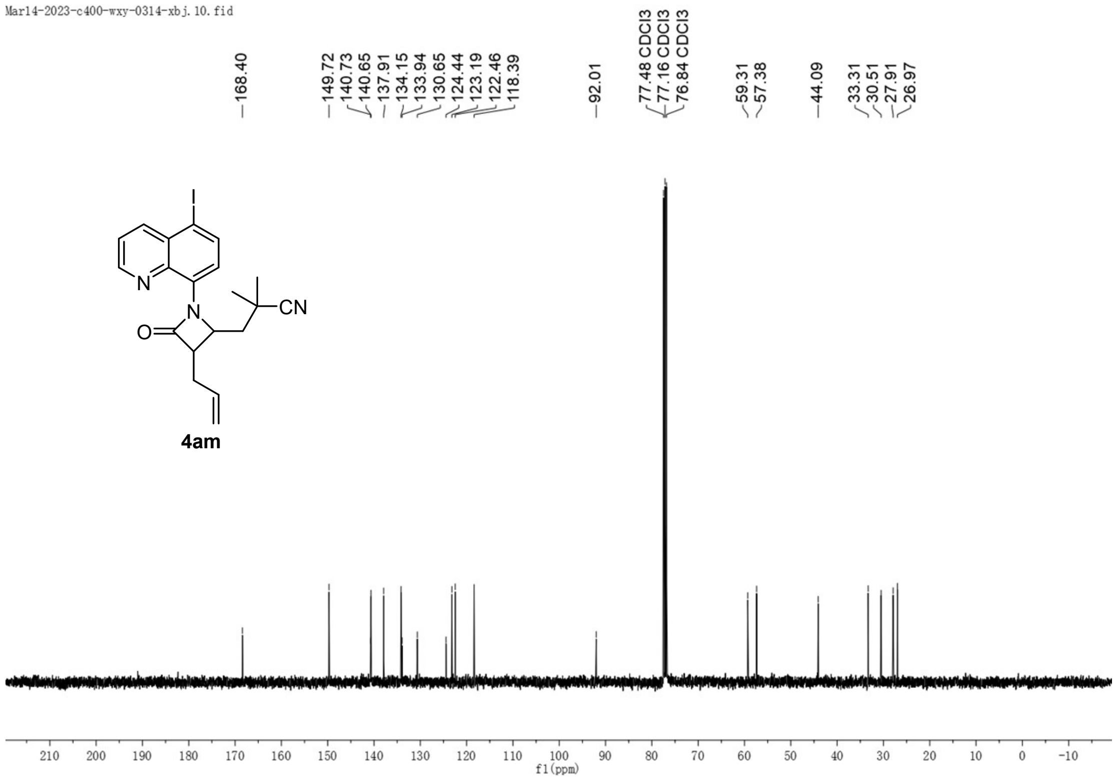

^{1}H NMR of 4an ($CDCl_3$, 400 MHz)

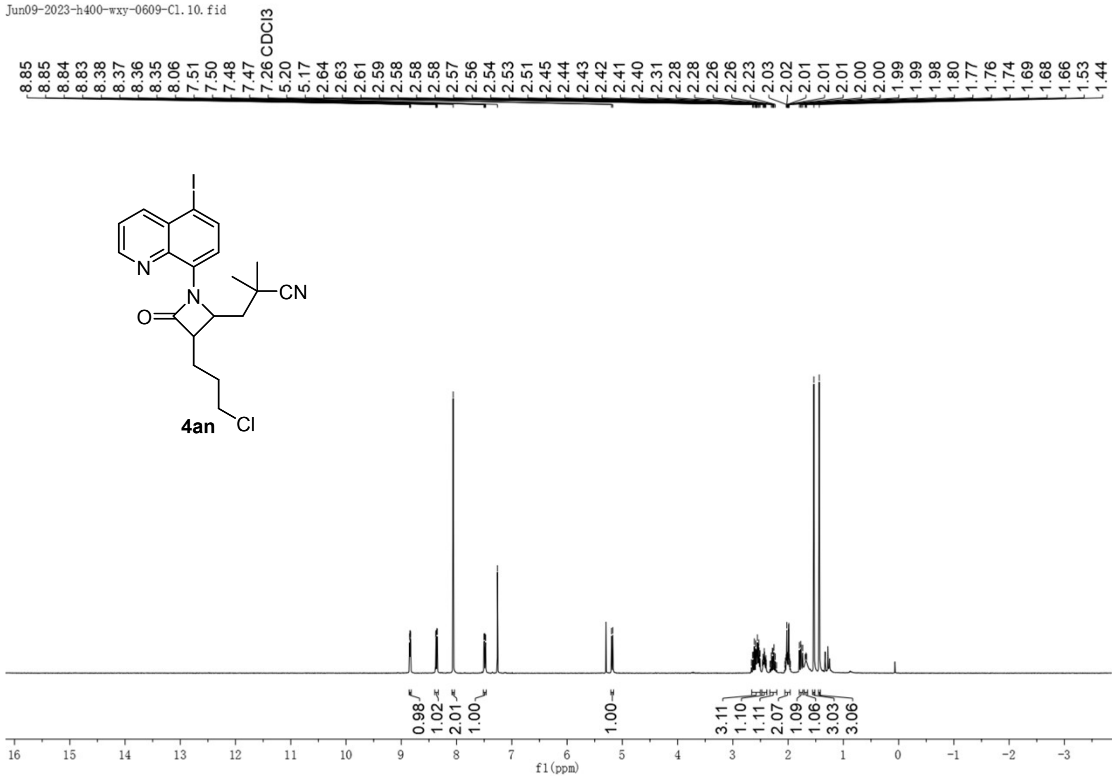

^{13}C NMR of 4an ($CDCl_3$, 101 MHz)

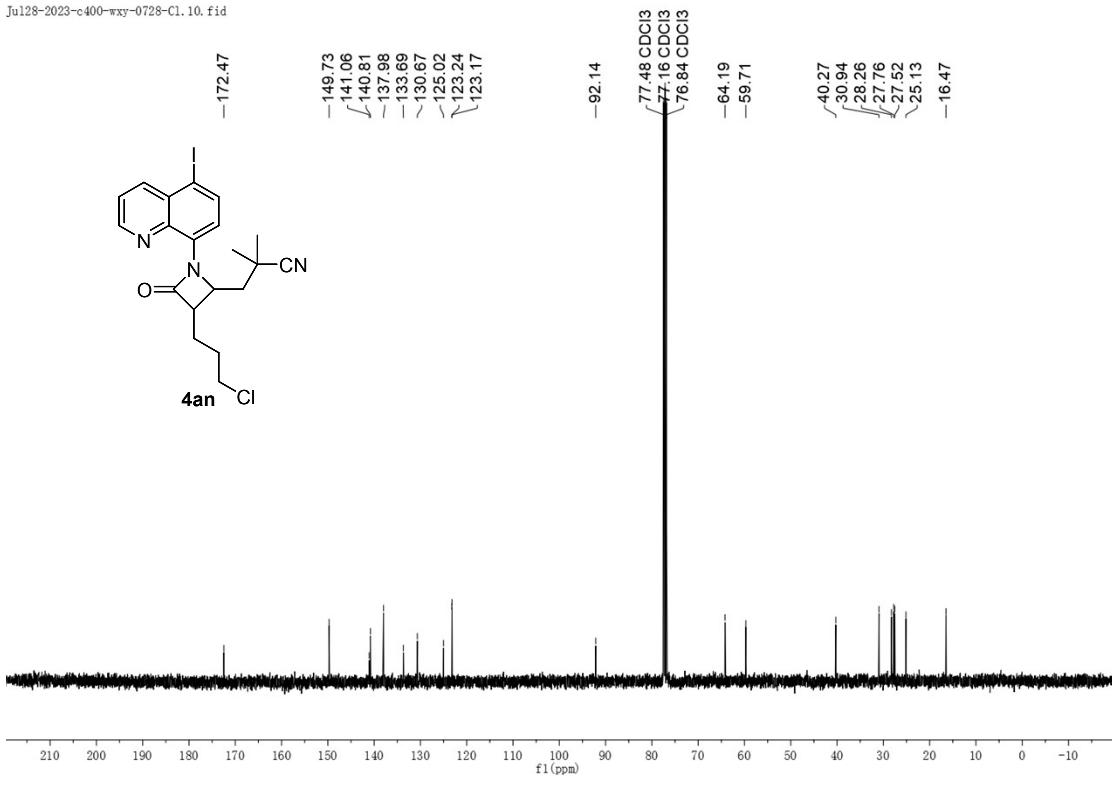

^{1}H NMR of 4ao ($CDCl_3$, 400 MHz)

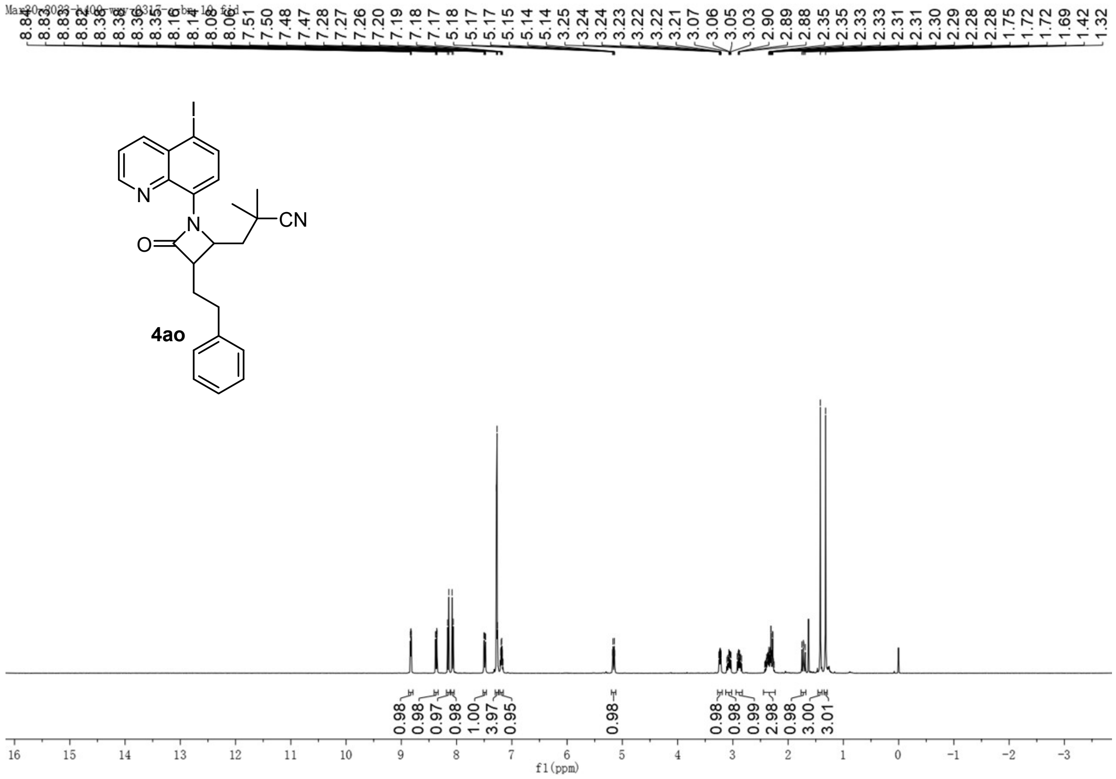

^{13}C NMR of 4ao ($CDCl_3$, 101 MHz)

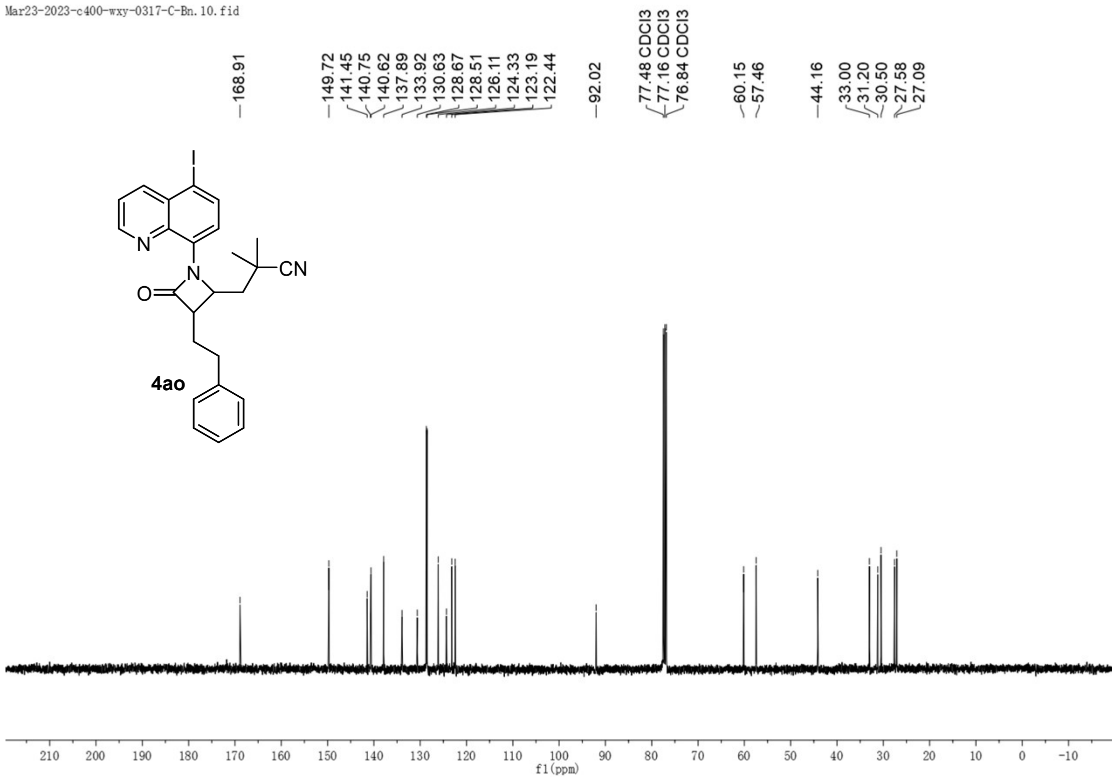

^{1}H NMR of 4ap ($CDCl_3$, 400 MHz)

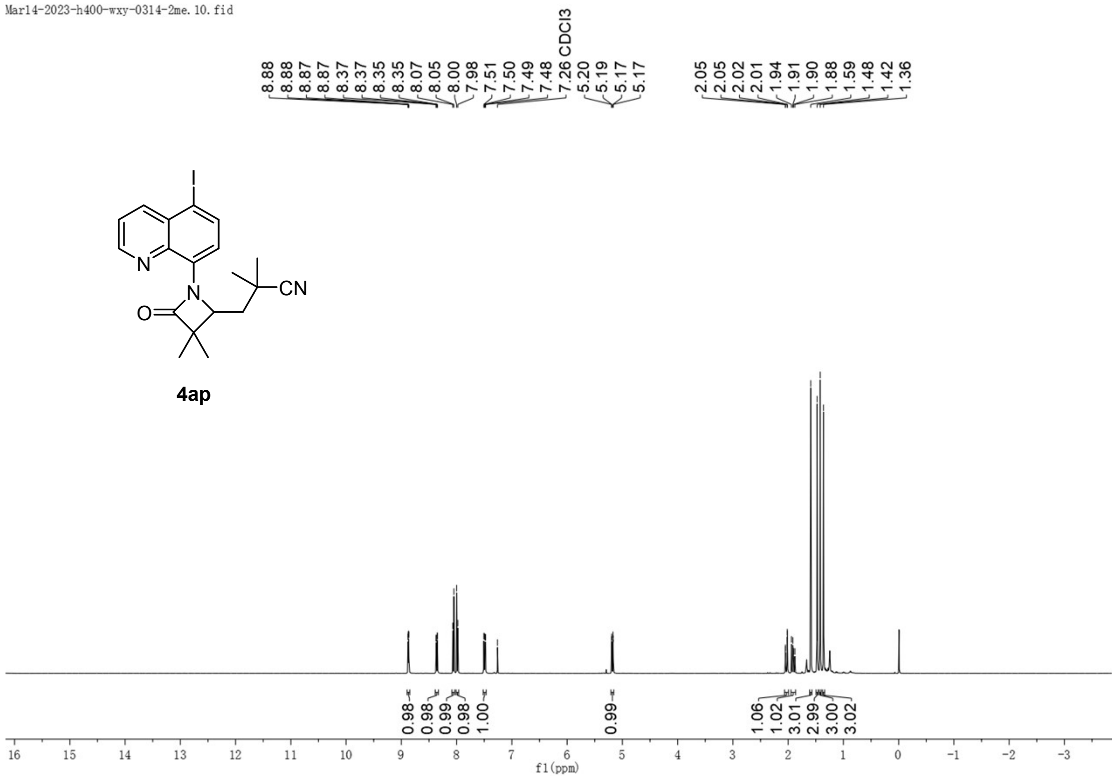

^{13}C NMR of 4ap ($CDCl_3$, 101 MHz)

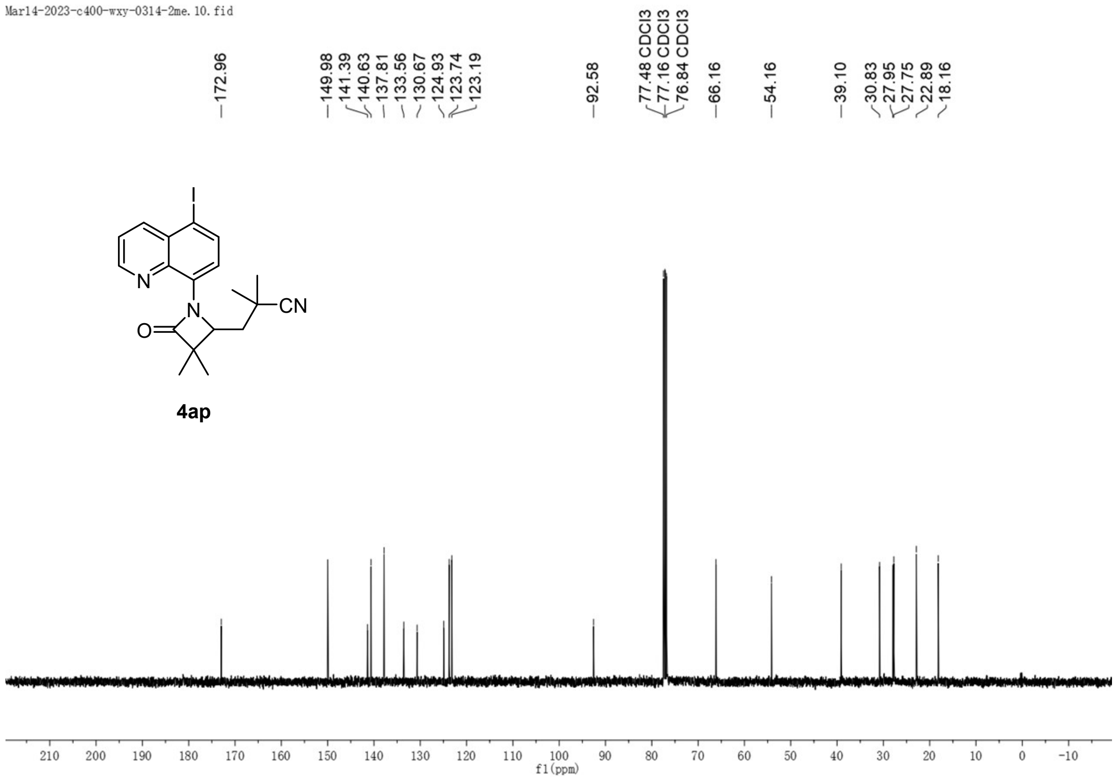

^{1}H NMR of 4as ($CDCl_3$, 400 MHz)

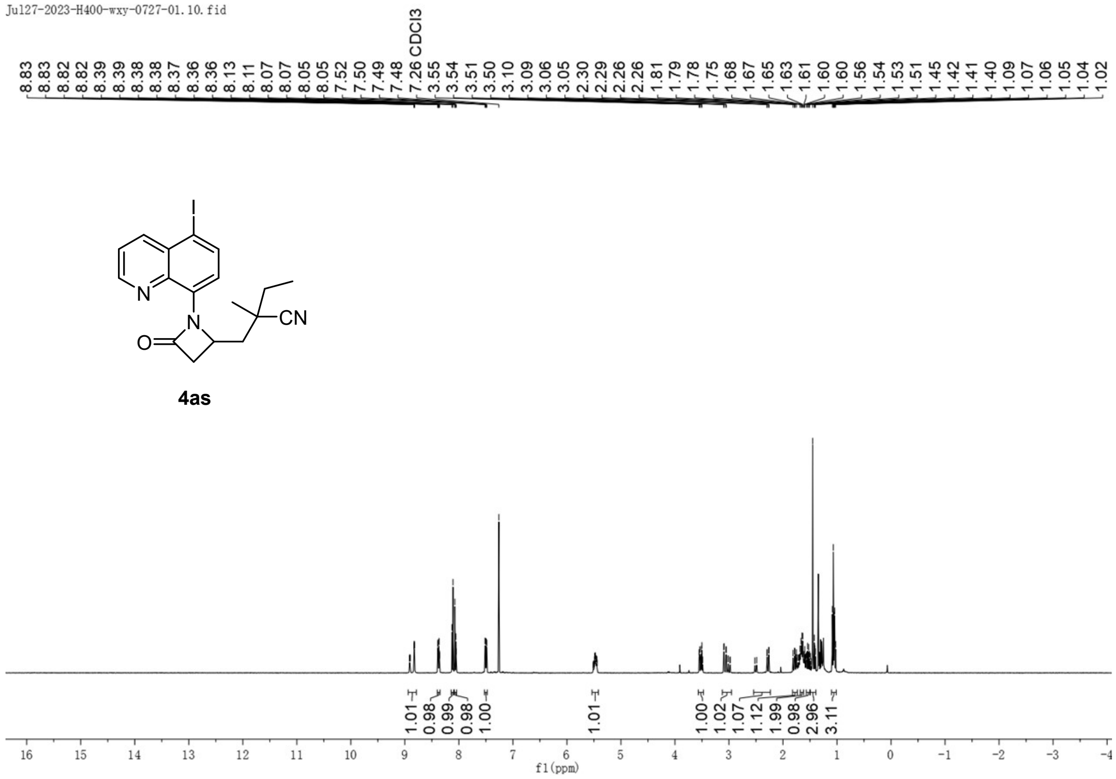

^{13}C NMR of 4as ($CDCl_3$, 101 MHz)

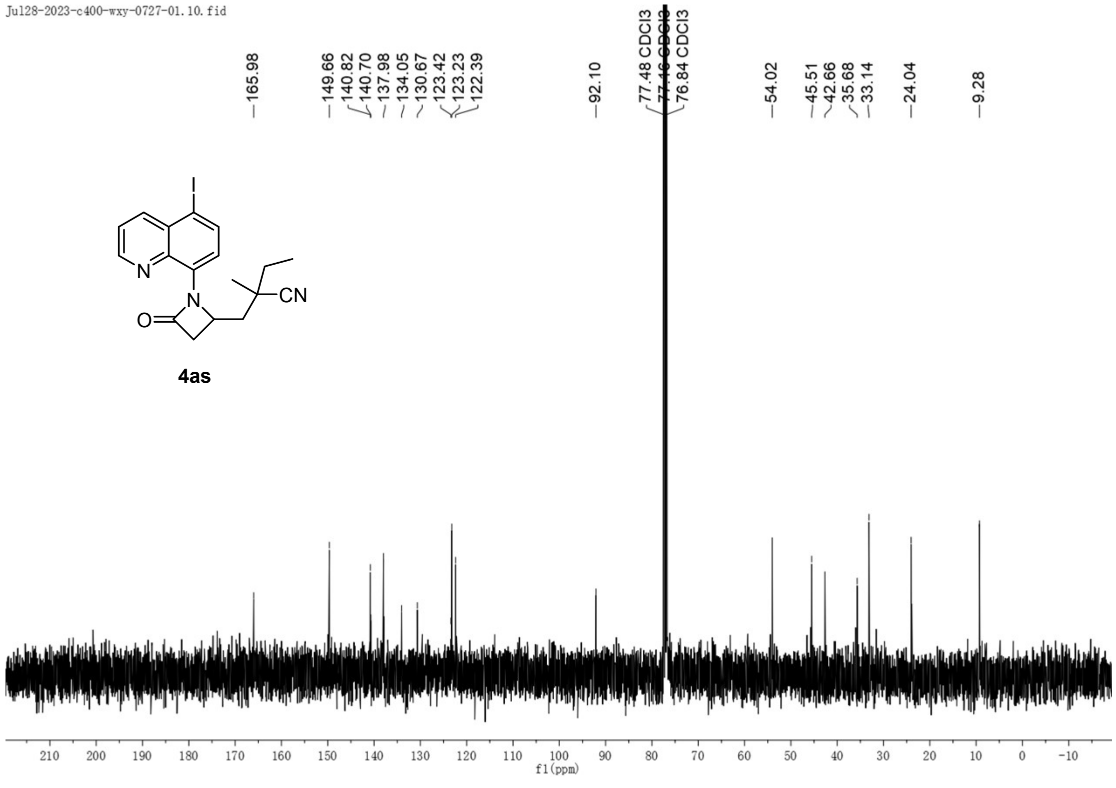

^{1}H NMR of 4at ($CDCl_3$, 400 MHz)

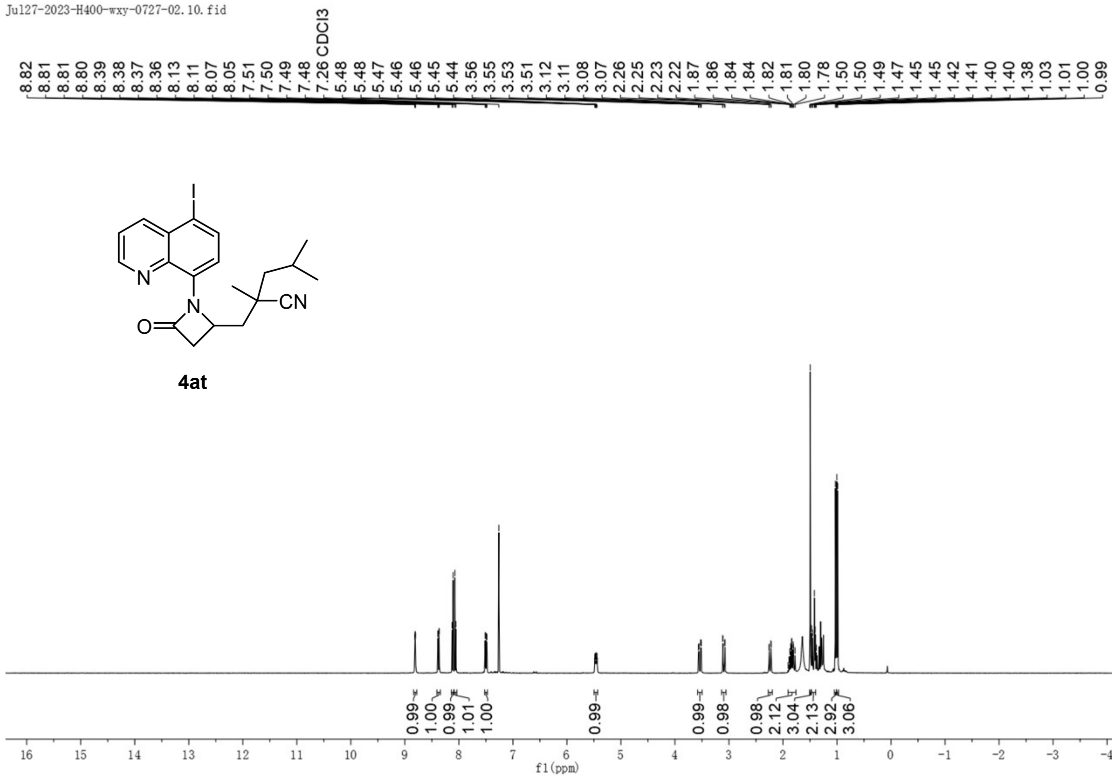

^{13}C NMR of 4at ($CDCl_3$, 101 MHz)

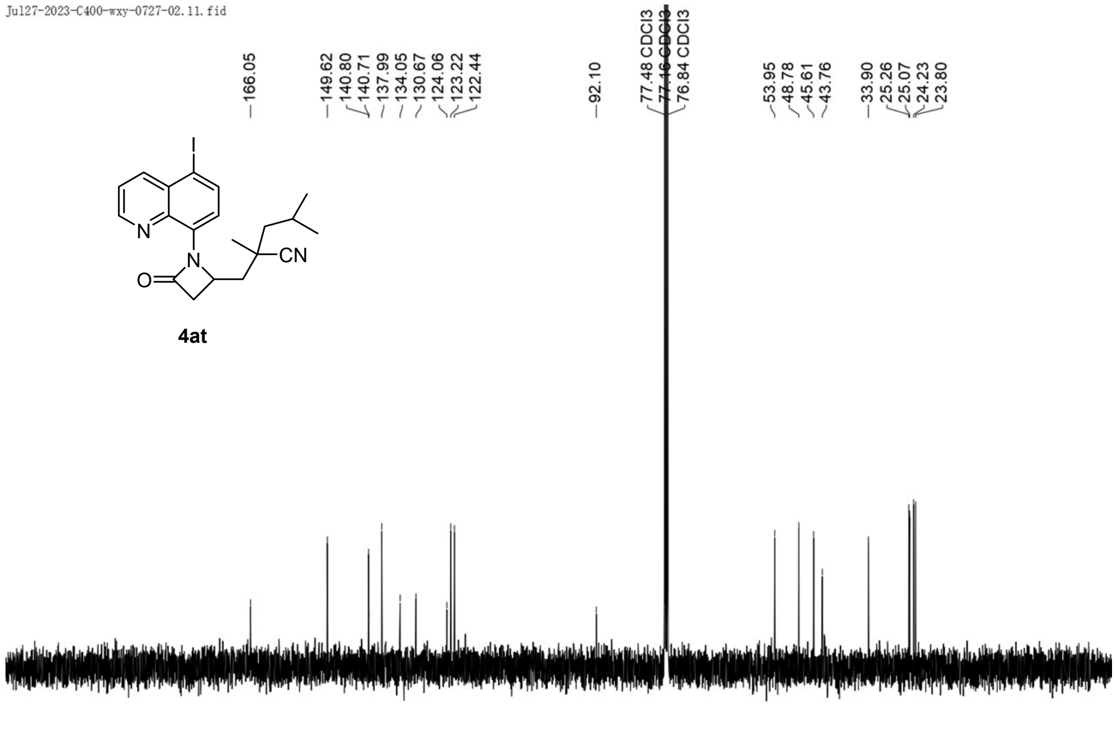

^{1}H NMR of 5 ($CDCl_3$, 400 MHz)

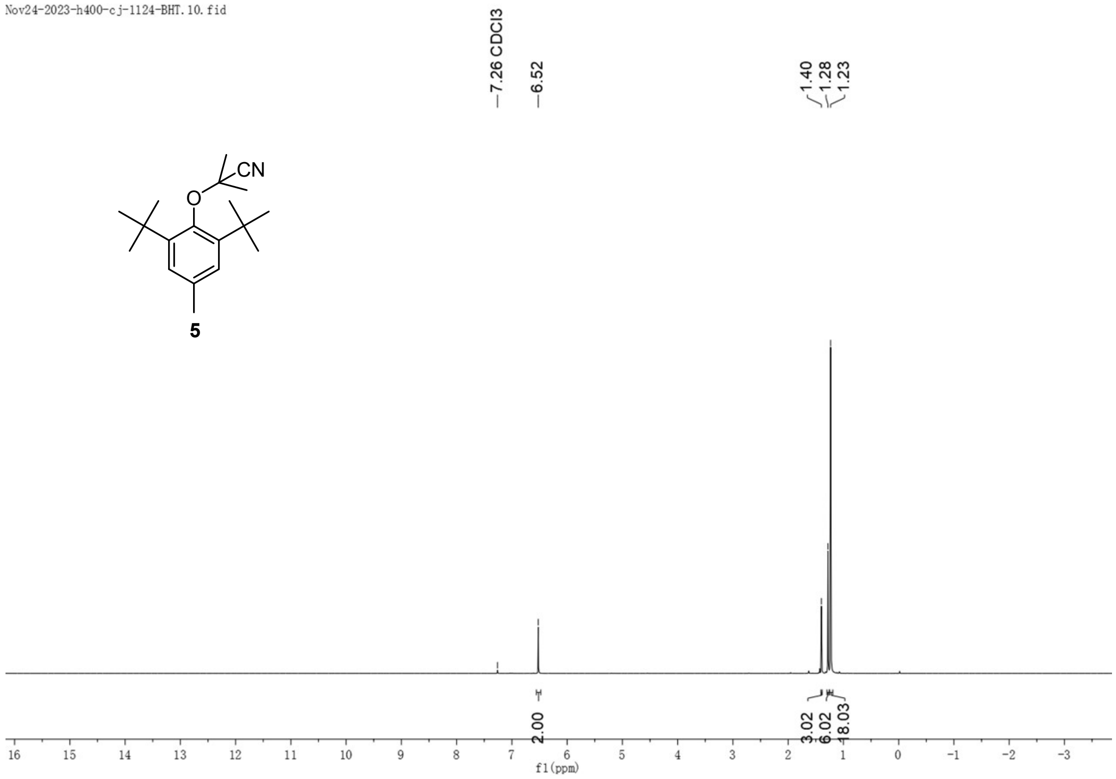

^{13}C NMR of 4at ($CDCl_3$, 101 MHz)

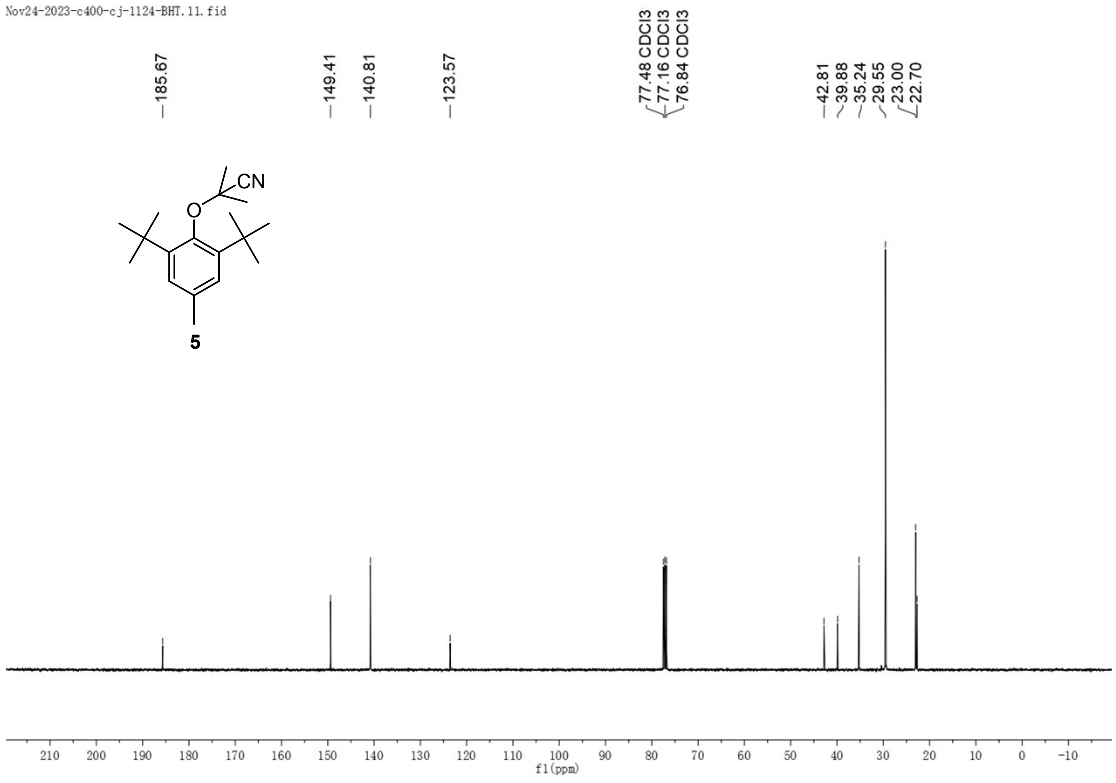

Supplement: RA-014-D4RA04113E-s001 [file RA-014-D4RA04113E-s001.pdf]
